# Stress and productivity patterns of interrupted, synergistic, and antagonistic office activities

**DOI:** 10.1038/s41597-019-0249-5

**Published:** 2019-11-08

**Authors:** Shaila Zaman, Amanveer Wesley, Dennis Rodrigo Da Cunha Silva, Pradeep Buddharaju, Fatema Akbar, Ge Gao, Gloria Mark, Ricardo Gutierrez-Osuna, Ioannis Pavlidis

**Affiliations:** 1 Computational Physiology Laboratory, University of Houston, Houston, USA; 2 Perception, Sensing, and Instrumentation Laboratory, Texas A & M University, College Station, USA; 3 Department of Informatics, University of California, Irvine, USA; 4 College of Information Studies, University of Maryland, College Park, USA

**Keywords:** Computer science, Business, Human behaviour

## Abstract

We describe a controlled experiment, aiming to study productivity and stress effects of email interruptions and activity interactions in the modern office. The measurement set includes multimodal data for *n* = 63 knowledge workers who volunteered for this experiment and were randomly assigned into four groups: (G1/G2) Batch email interruptions with/without exogenous stress. (G3/G4) Continual email interruptions with/without exogenous stress. To provide context, the experiment’s email treatments were surrounded by typical office tasks. The captured variables include physiological indicators of stress, measures of report writing quality and keystroke dynamics, as well as psychometric scores and biographic information detailing participants’ profiles. Investigations powered by this dataset are expected to lead to personalized recommendations for handling email interruptions and a deeper understanding of synergistic and antagonistic office activities. Given the centrality of email in the modern office, and the importance of office work to people’s lives and the economy, the present data have a valuable role to play.

## Abstract (summary)

**Table:** 

Measurement(s)	thermal facial data • heart rate • respiration rate • facial expression • hand activity • psychometric scores • keyboard data • biographic data
Technology Type(s)	Computerized Thermal Imaging • sensor • digital camera • questionnaire • computational modeling technique
Factor Type(s)	type of distraction • anticipatory stressor
Sample Characteristic - Organism	Homo sapiens
Sample Characteristic - Environment	office building

Machine-accessible metadata file describing the reported data: 10.6084/m9.figshare.9970304

## Background & Summary

Modern office work is the backbone of the economy^1^, defining the careers and lives of many people^2^. Naturally, productivity^3^ and well being^4^ at the office has received a lot of attention from the research community. In this context, interruptions of office tasks^5^ and their effects^6^ have been studied extensively both experimentally^7^ and *in-situ*^8^. Interruptions can arise in different forms, such as phone calls and face-to-face exchanges; for over a decade now social media and text messaging have been added into the mix. Email, however, remains a key source of interruptions in office work.

Email’s main effects and interactions with other office tasks, taking into account personality profiles, are under-explored. This is especially true when effects are not restricted only to productivity but extend to include workers’ stress levels measured in real-time. Here, we describe an experiment to provide comprehensive answers regarding the role of email interruptions. This experiment and the associated dataset have three unique aspects:

1. They afford the study of email use patterns that interact with ubiquitous office stressors, and how these interactions are modulated by individual characteristics. Most other studies treat email use in isolation and ignore profiles of office workers.
2. They afford second by second study of workers’ stress responses during the experimental manipulation. These stress responses are recorded through five different physiological sensing channels - all acquired unobtrusively via imaging or wearable devices.
3. They combine keystroke dynamics, written/oral content, and facial video. These information channels synergistically support investigations into participant emotions^9–12^, triggered by the experimental stressors.

Moreover, because the email treatments were designed in the midst of other representative office tasks to provide context, this study offers opportunities to investigate behaviors across a broader spectrum of information work activities. Examples of such investigations afforded by the data include:

1. How synergistic activities contrast with antagonistic activities. The experimental design provides for report writing free of exogenous considerations vs. report writing burdened by anticipation for an upcoming presentation.
2. How people cope with delivery of critical presentations when they are prepared vs. when they are unprepared.

Importantly, the dataset features a comprehensive array of measurement channels and ancillary media. Many of these channels and media are multidimensional (e.g., videos), constituting an interrelated collection of long data ripe for machine learning. Hence, it is a rich resource for the scientific community to perform not only hypothesis-driven but also exploratory research. The dataset is also expected to serve as a benchmark while communication forms continue to evolve - e.g., future office studies may replace email with a social network app, such as Slack (Slack Technologies, Inc., https://slack.com), referring back to the current study for comparison.

Analyzing a subset of the data presented in this paper, we reported in CHI’19^13^ that for neurotic individuals batched email is more stressful than continually incoming email. This is in contradistinction to commonly held views that batched email is uniformly preferable to instantaneous servicing of emails^14^. The CHI ’19 work gives a small flavor of the dataset’s potential for consequential behavioral and ergonomic research.

## Methods

### Ethics statement

The experimental procedures were approved by the Institutional Review Boards (IRB) of the Texas A&M University (protocol # IRB2017-0271D), the University of Houston (protocol # STUDY00000343), and the University of California, Irvine (protocol # IRB2017-3637). The authors performed these procedures in accordance with the approved guidelines, obtaining informed consent from each participant before conducting the experiments. In the consent form, the participants were given two explicit data use & release options, for which a separate signature was required:

#### OPTION A

Participants that selected this option consented both to the research use and public release of all their experimental data, including video data bearing identifying information.

#### OPTION B

Participants that selected this option consented to the in-house research use of all their experimental data, but did not consent to the public release of experimental data bearing identifying information. Hence, this excluded from the public version of the dataset the facial and operational theater videos of these participants.

### Participants

We recruited participants from the Texas A&M University (pop. 68,000), the University of Houston (pop. 45,000), and the University of California, Irvine (pop. 33,000) communities. Calls for participation were disseminated through email solicitations, portals, and flyer postings. We restricted admission to individuals whose native language was English or were bilingual, had at least high-school education, and were at the age of 18 or above. A total of 96 participants conforming to the inclusion-exclusion criteria volunteered for the study. Raw data for *n* = 33 participants were not properly recorded due to technical issues. Raw data for *n* = 63 participants were nearly complete, constituting our working set; 28 of these participants signed for OPTION A, while 35 for OPTION B.

### Experimental setup

**Fig. 1:**
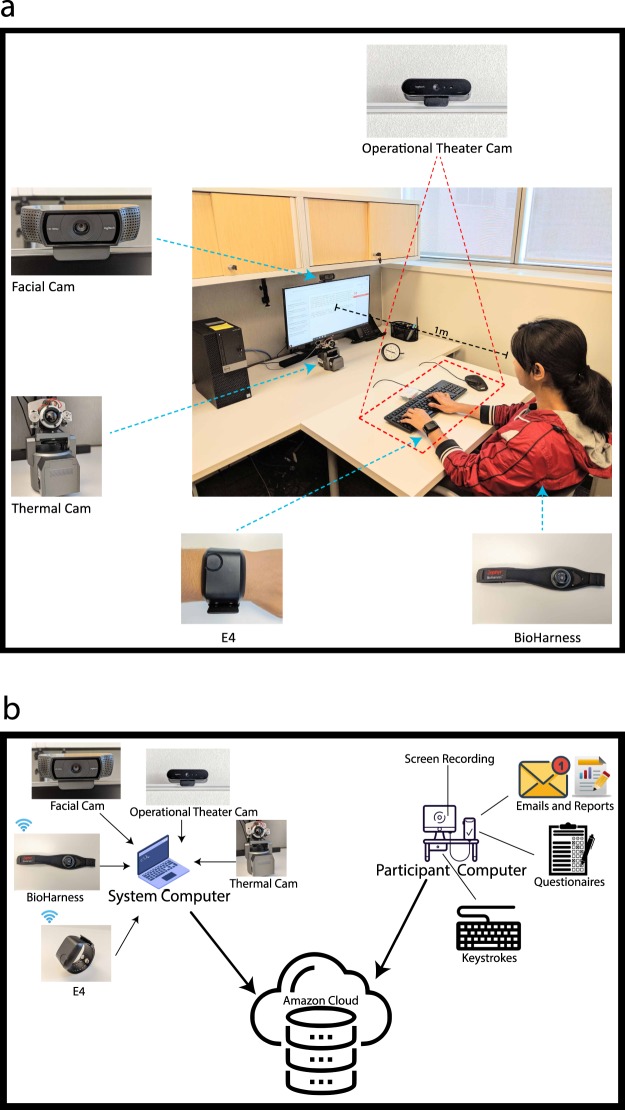
Experimental setup and system architecture. (**a**) Experimental setup. The specific setup is from the University of Houston site; mirror setups existed at Texas A&M University and the University of California, Irvine. (**b**) Experimental system architecture. Information channels acquired by the system and participant computers are archived in the Amazon cloud.

We carried out the study in three office rooms, each located in one of the participating campuses. The experiments in these offices were conducted by personnel trained the same, using identical systems and layouts (Fig. 1a). During the experimental sessions, the systems continuously imaged the participants’ faces with a thermal and visual camera. An additional visual camera angled down from the ceiling was imaging the participants’ desktop area. The systems were also capturing the screen and keystrokes of the participants’ computer, while two wearable devices were relaying the participants’ physiological signals. A detailed description of each system component follows.

#### Thermal facial camera

A Tau 640 long-wave infrared (LWIR) camera (FLIR Systems, Wilsonville, OR), featuring a small size (44 × 44 × 30 mm) and adequate thermal (50° mK) and spatial resolution (640 × 512 pixels). It is outfitted with a LWIR 35 mm lens f/1.2, controlled by a custom auto-focus mechanism. The camera is located under the participant’s computer screen (Fig. 1a), attached to Bescor MP-101 Motorized Pan & Tilt Head (Bescor, Farmingdale, NY) to facilitate face tracking. Thermal facial data are collected at a frame rate of 7.5 fps or higher. We use these thermal facial sequences to extract perinasal perspiration signals, known to commensurate with electrodermal (EDA) activity in the palm^15^.

#### Wrist EDA & heart rate sensors

An E4 wristband device (Empatica Inc., Milano, Italy) that collects EDA and heart rate measurements from participants’ non-dominant hand (Fig. 1a). EDA is measured via E4’s silver (Ag) electrode (valid range [0.01–100] μS), while heart rate is measured via E4’s photoplethysmographic (PPG) sensor. The E4 wristband is powered by a rechargeable lithium battery and transmits data to the system’s computer via Bluetooth (Fig. 1b).

#### Chest breathing & heart rate sensors

A Zephyr BioHarness 3.0 device (Zephyr Technology, Annapolis, MD) that measures participants’ breathing rate and heart rate. The device has the form of a chest strap that is worn underneath the participant’s clothing (Fig. 1a). BioHarness is powered by a rechargeable lithium polymer battery (up to 26 h per charge) and transmits data to the system’s computer via Bluetooth (Fig. 1b). It can reliably measure breathing rate in the range [4–40] BPM, while heart rate in the range [40–140] BPM.

#### Visual facial camera

A Logitech HD Pro - C920 camera (Logitech, Newark, CA) with spatial resolution 1920 × 1080 pixels and a frame rate of 30 fps. It is located 1 m from participants, tucked atop their computer screen (Fig. 1a). Appropriate lens zooming for the said distance ensures participants’ faces fit well in the field of view. Such tight fit provides sufficient resolution for analysis of facial expressions.

#### Visual operational theater camera

A Logitech Brio camera (Logitech, Newark, CA) with spatial resolution 1280 × 720 pixels and a frame rate of 30 fps. It is located 2 m from the participant’s desktop, tucked into the ceiling and angled downwards (Fig. 1a), in order to record hand activity.

#### System computer

A Dell Lattitude E5570 laptop computer, featuring an Intel DualCore i7–6600U 2.6 GHz processor, 16 GB RAM, and 500 GB hard disk. This laptop hosts the main thread of S-Interface, that is, the software that performs synced data acquisition from sensors (Fig. 1b). The acquired data are curated at the end of each experiment and archived in the Amazon cloud.

#### Participant computer

A Dell OptiPlex 7050 desktop computer, featuring an Intel QuadCore i7–7700 3.6 GHz processor, 16 GB RAM, and 1 TB solid state disk. The computer is connected to a Dell U2417H-Ultrasharp 24 in display. Participants use this computer to perform the assigned tasks per the experimental design (Fig. 1a). In fact, an automated interface guides participants step by step through the experimental process to enforce precise protocol execution. Another piece of software, a clone of S-Interface, acquires and curates screen capture recordings and participant input (Fig. 1b). Screen capture recordings are effected through S-Interface calls on VisioForge’s Software Development Kit (SDK). Participant input includes biographic/psychometric questionnaires, emails/reports, and keystroke dynamics. All curated data are archived in the Amazon cloud.

### Experimental design

To establish individual profiles, all participants complete a biographic questionnaire, the Big Five Inventory, the Emotion Regulation Questionnaire, and the Perceived Stress Scale. In the core experiment, participants undergo five treatments. Treatments and information given to participants differ in parts depending on the group they are assigned. Specifically, participants are randomly assigned to four groups in a 2 × 2 factorial design. There are two factors: Email Mode and Anticipatory Stressor. Each factor has two levels: Email Mode {**B**atch, **C**ontinual} × Anticipatory Stressor {**H**igh, **L**ow} = {**BH** (*n* = 15), **BL** (*n* = 14), **CH** (*n* = 17), **CL** (*n* = 17)}. The Continual level is characterized by a pseudo-periodic arrival of emails throughout the main session, involving a writing task, while the Batch level is characterized by simultaneous delivery of all the emails towards the beginning of the session. The Anticipatory High Stressor is implemented by forewarning participants about an upcoming presentation. The Low Stressor group is spared such anticipation. The treatments are applied in the following order:

#### Resting baseline (RB)

Participants take a deep breath, close their eyes, and think of something relaxing for 4 min. The purpose of this session is to bring participants’ arousal close to their tonic levels, so it could be used as a normalizing anchor for the physiological measures taken during the treatments. The tonic level of arousal differs significantly among people, something that is reflected in the physiological readings. Hence, absolute physiological measurements during treatments are not reliable stress indicators for participants; what matters is how much their arousal has increased with respect to their characteristic tonic level.

#### Single task (ST)

Participants have 5 min to write a short report expressing their opinions on the subject of competition vs. collaboration. This session allows participants to warm up for the subsequent writing session, and provides a baseline of writing skills for each participant.

#### Priming (PM): Stroop OR relaxing video (RV)

This session occurs directly before the main writing session in order to reinforce arousal for the Anticipatory High Stressor group, while subduing arousal for the Low Anticipatory Stressor group. Priming for the Anticipatory High Stressor group is implemented via 5 min of the Stroop color word test, while priming for the Anticipatory Low Stressor group is implemented via 5 min of viewing a relaxing natural landscape video.

#### Dual task (DT)

This is the main writing session. Participants are asked to write a report on the topic of technological singularity (i.e., when machines overtake human intelligence). Participants in the two Anticipatory High Stressor groups are told that they have to present their report to a panel of judges at the end of the session. Participants are given 50 min to compose the report, during which they also have to respond to eight emails (secondary task). In the Batch group, the eight emails arrive 10 minutes after the start of the DT, and participants have 5 min to start replying to them. In the Continual group, individual emails arrive every 4 min (on average), and participants have 10 s to start replying to each email. If participants do not start their reply within the transitional time allotted, the interface shifts into the email page in order to ensure consistency across participants of the same email group (Batch or Continual). Five emails ask for opinion/advice and three are scheduling tasks. The order of the emails is randomized. At the end of DT, participants complete the NASA TLX questionnaire to gauge psychometrically the loading induced by the experiment’s main treatment.

The emails, the DT report prompt, and the ST report prompt are available in the Supplementary Information file. We chose the DT and ST report topics so that they are of interest to knowledge workers and require careful thought. The five opinion/advice email prompts were chosen from a pilot study on MTurk, where an original selection of 30 emails were presented to 270 workers on the platform. Each email was presented to 9 different MTurk workers who were asked to compose a reply as if they worked for a company. Then, we selected the five emails that generated the highest mean word count in replies.

#### Presentation (PR)

At the end of DT, all participants are asked to deliver a 5-minute oral presentation in front of a panel of three judges, who attend remotely (via Skype). In fact, this is a pre-recorded video with actors that gives the impression of a live session. As a result, both realism and stimulus consistency are maintained across the participant sample. Public speaking is an example of a Trier Social Stress^16^ - a stimulus of stronger intensity with respect to the milder report stressors designed in this experiment. Hence, in addition to serving as an anticipatory stressor for the BH and CH groups, the presentation session also acts as an upper bound of stress for all groups, facilitating additional validation of our measurement methods.

### Computation

Algorithmic processing of the thermal imagery yields a signal that quantifies perinasal perspiration. The algorithm includes a virtual tissue tracker that keeps track of the region of interest, despite participants’ small head motions. This ensures that the thermophysiological signal extractor operates on consistent and valid sets of data over the clip’s timeline.

#### Thermal imaging - tissue tracking

We use the tissue tracker reported by Zhou *et al*.^17^. On the initial frame, the experimenter initiates the tracking algorithm by selecting the participant’s perinasal region. The tracker estimates the best matching block in every next frame of the thermal clip via spatio-temporal smoothing. A visual example of the tissue tracking operation is shown in the TOP row of Supplemental Fig. S1.

#### Thermal imaging - perinasal perspiration signal extraction

In facial thermal imagery, activated perspiration pores appear as ‘cold’ (dark) spots, amidst ‘hot’ surrounding tissue. A morphology-based algorithm is applied on the measurement region of interest (MROI) to compute the perspiration signal^15^; MROI refers to the upper orbicularis oris portion of the tracked perinasal tissue. The MIDDLE row of Supplemental Fig. S1 shows the evolving thermal signature of perspiration spots in the MROI of participant T005, as she undergoes moments of low and high arousal. The said algorithm quantifies the manifested spatial frequency pattern, extracting an energy signal **E**(*k*, *j*), indicative of perspiration activity in the perinasal MROI of participant *k*, for treatment *j* (BOTTOM panel of Supplemental Fig. S1). Any high-frequency noise in this signal is suppressed by a Fast Fourier Transformation (FFT) filter.

## Data Records

The data are freely available on the Open Science Framework (OSF)^18^. The OSF repository has quantitative data, textual data, and ancillary media organized participant-wise; it also holds the custom software tools and other material we used to acquire these data.

### Quantitative data folder

The quantitative data folder holds four comma separated value (csv) files, as well as the voluminous thermal imaging data: (1) Questionnaire Data - 5 KB. (2) Physiological Data - 21.8 MB. (3) Keyboard Data - 42.7 MB. (4) Report Data - 13.9 KB. (5) Thermal Imaging Data - 1.86 TB.

The first two columns of the csv files hold the participant ID and group, respectively. The participant ID is coded as T_xyz_, while the group assignment takes values from the set [BH, BL, CH, CL]. The remaining csv columns are specific to the corresponding type of data; their description follows.

### Questionnaire data file

In the Questionnaire Data file, in addition to the columns holding the participant ID (***Column*** **A**) and group information (***Column*** **B**), there are columns holding biographic data (***Columns*** **C - K**) and other columns holding scores from psychometric inventories (***Columns*** **L - Y**). Specifically:

***Column*** **C: Age:** Age of participants in years.

***Column*** **D: Gender:** Gender of participants [1 ≡ Male, 2 ≡ female].

***Column*** **E: Nationality:** Nationality of participants [1 ≡ United States, 2 ≡ Others].

***Column*** **F: Other_Nationality:** Nationality of non-U.S. participants.

***Column*** **G: Native_Language:** Mother tongue of participants [1 ≡ English, 2 ≡ Others].

***Column*** **H: Other_Native_Language:** Mother tongue of bilingual participants.

***Column*** **I: Education:** Educational level of participants [1 ≡ High School, 2 ≡ Undergraduate, 3 ≡ Master or equivalent, 4 ≡ PhD, JD, or equivalent].

***Column*** **J: Writing_Proficiency:** Self-reported writing proficiency of participants in a seven-point Likert scale, where 1 ≡ Not fluent at all and 7 ≡ Very fluent.

***Column*** **K: Daily_Email_Frequency:** Self-reported daily use of email in a seven-point Likert scale, where 1 ≡ Never and 7 ≡ Very often.

**Big Five Inventory (BFI)** - A trait psychometric related to the participant’s key personality factors^19^. It has five sub-scales.

- ***Column***n **L: BFI_Agreeableness:** The level of participant’s friendliness with score range [9–45].
- ***Column*** **M: BFI_Conscientiousness:** The level of participant’s organized nature with score range [9–45]
- ***Column***n **N: BFI_Extraversion:** The level of participant’s outgoing nature with score range [8–40].
- ***Column*** **O: BFI_Neuroticism:** The level of participant’s nervousness with score range [8–40].
- ***Column*** **P: BFI_Openness:** The level of participant’s curiosity with score range [10–50].
- **Emotion Regulation Questionnaire (ERQ)** - A trait psychometric related to the participant’s ability to regulate emotions^20^. It has two sub-scales.
- ***Column*** **Q: ERQ_Cognitive_Reappraisal:** The degree to which a participant can change the way s/he thinks about emotion-eliciting events with score range [6–42].
- ***Column*** **R: ERQ_Expressive_Suppression:** The degree to which a participant can change the way s/he responds to emotion-eliciting events with score range [4–28].
  ***Column*** **S: Perceived Stress Scale (PSS):** Level of non-specific perceived stress of participants with score range [0–40]. This is a trait psychometric that predicts health-related outcomes associated with appraised stress^21^.
  **NASA TLX** - A state psychometric administered upon completion of DT to gauge the perceived loading this task induced to participants. NASA TLX^22^ features six sub-scales with common rating [1 = Strongly disagree, 2 = Disagree, 3 = Somewhat disagree, 4 = Neither agree or disagree, 5 = Somewhat agree, 6 = Agree, 7 = Strongly agree].
- ***Column*** **T: NASA_Mental_Demand:** Perceived mental load induced by DT.
- ***Column*** **U: NASA_Physical_Demand:** Perceived physical activity induced by DT.
- ***Column*** **V: NASA_Temporal_Demand:** Perceived time pressure induced by DT.
- ***Column*** **W: NASA_Performance:** Perceived success in executing DT.
- ***Column*** **X: NASA_Effort:** Perceived amount of work expended to achieve the said level of DT performance.
- ***Column*** **Y: NASA_Frustration:** Perceived level of irritation in performing DT.

### Physiological data file

In the Physiological Data file, in addition to the columns holding the participant ID (*Column* **A**) and group information (*Column* **B**), there are columns holding treatment information, task information, timing, and signal data from the various sensing modalities used in the experiment. The recordings of the physiological sensors were synced. Hence, each row from left to right holds the time and the synced set of modal signal values recorded at that time. The temporal resolution is fixed at 1 s across the board to match the slowest physiological channels Chest BR and Chest HR. The main data repository^18^ holds the quality controlled values of the physiological variables (**_QC**). The raw variable values and the R code that operates upon them to implement the processes described herein, reside in GitHub (Zaman, S. & Pavlidis, I. Office-Tasks-2019-Methods. *GitHub* https://github.com/UH-CPL/Office-Tasks-2019-Methods). In more detail:

***Column*** **C: Treatment:** The treatment during which each set of modal signal values was recorded.

***Column*** **D: Time:** The recorded date and time for each set of modal signal values.

***Column*** **E: Treatment_Time:** The time elapsed in seconds since the start of the present treatment.

***Column*** **F: Task:** Labeling of email vs. report writing activity during DT.

***Column*** **G: PP_QC:** Values of the perinasal perspiration signal in °C^2^.

***Column*** **H: EDA_QC:** Values of the EDA signal in μS, measured with E4 in the wrist of the participant’s non-dominant hand.

***Column*** **I: BR_QC:** Values of the breathing rate signal in BPM, measured with the BioHarness in the participant’s chest.

***Column*J: Chest_HR_QC:** Values of the heart rate signal in BPM, measured with the BioHarness in the participant’s chest.

***Column*** **K: Wrist_HR_QC:** Values of the heart rate signal in BPM, measured with E4 in the wrist of the participant’s non-dominant hand.

### Keyboard data file

In the Keyboard Data file, in addition to the Columns holding the participant ID (***Column*** **A**), group information (***Column*** **B**), treatment information (***Column*** **C**), time information (***Column*** **D**), and task information (***Column*** **E**), there are columns holding keystroke information. Specifically:

***Column*** **F: Is_Key_Up:** 0 stands for key depressed, while 1 stands for key released.

***Column*** **G: Key:** Alphanumeric code of the key that is either released or depressed.

### Report data file

In the Report Data file, in addition to the columns holding the participant ID (***Column*** **A**), group information (***Column*** **B**), and treatment information (***Column*** **C**), there are ***Column***s holding report length measures, writing quality measures by the *e-rater* scoring engine of the Educational Testing Service (ETS)^23^, and usage measures for the delete keys.

***Column*** **D: Word_Count:** The number of words in the report.

***Column*** **E: Character_Count:** The number of characters in the report.

***Column*** **F: Criterion_Score:** The overall report quality score given by the *e-rater*.

***Column*** **G: Mechanics_Errors:** Number of mechanics errors in the report, such as spelling errors; it is provided by the *e-rater*.

***Column*** **H: Grammar_Errors:** Number of grammar errors in the report, such as subject-verb agreement errors; it is provided by the *e-rater*.

***Column*** **I: Usage_Errors:** Number of usage errors in the report, such as article errors; it is provided by the *e-rater*.

***Column*** **J: Style_Errors:** Number of style errors in the report, such as repetition of words and very short or very long sentences; it is provided by the *e-rater*.

***Column*** **K: Delete_Key_Count:** The number of times the backwards and forward delete keys were depressed during the writing of the report. This information is extracted from the Keyboard Data file.

***Column*** **L: Mechanics_Errors/WC:** The number of mechanics errors divided by the number of words in the report.

***Column*** **M: Grammar_Errors/WC:** The number of grammar errors divided by the number of words in the report.

***Column*** **N: Usage_Errors/WC:** The number of usage errors divided by the number of words in the report.

***Column*** **O: Style_Errors/WC:** The number of style errors divided by the number of words in the report.

***Column*** **P: Delete_Key/CC:** The number of times the backwards and forward delete keys were depressed during the writing of the report, normalized per the report length in characters.

### Thermal imaging data subfolder

This subfolder contains the facial thermal imaging sequences acquired during experimentation via the S-Interface^24^. These sequences can be used for extraction of additional physiological indicators, such as breathing signals^25^, the re-extraction of perinasal perspiration signals, or other computer vision research. The files holding the thermal imaging sequences are in a binary format called .dat. Each .dat file is accompanied by a text file .inf. The header of each .inf file has three numbers: (1) The number of thermal frames contained in the corresponding .dat file. (2) The width of each thermal frame. (3) The height of each thermal frame. The body of each .inf file contains the timestamps of all thermal frames contained in the corresponding .dat file. The S-Interface uses.inf files to properly open the corresponding .dat files and process them.

### Textual data folder

#### Reports and emails file

This Excel file holds the ST (***Column*** **C**) and DT (***Column*** **D**) reports of participants, as well as the eight emails they wrote (***Column*** **E** to ***Column*** **L**); its size is 124.8 KB.

### Ancillary media folder

#### Facial videos

Visual videos of participants’ faces during experimentation. They are in mp4 format named as T_xyz_-FV.mp4; their total size is 53.6 GB.

#### Operational theater videos

Visual videos of participants’ desktop area during experimentation. They are in mp4 format named as T_xyz_-OTV.mp4; their total size is 53.6 GB.

#### Computer screen videos

Visual videos of participants’ computer screen during experimentation. They are in mp4 format named as T_xyz_-CSV.mp4; their total size is 69.1 GB.

#### Thermal MROI videos

Videos of participants’ perinasal MROI extracted through the S-Interface. The PP signals are computed upon these MROIs. The said videos are in mp4 format named as T_xyz_-MROI.mp4; their total size is 3.05 GB.

#### Tools folder

This folder contains the interfaces, applications, and videos needed to reproduce the present experiment and collect additional data. Specifically: (a) p-Interface for executing the experimental protocol; (b) Stroop application for stress priming in the BH and CH groups; (c) natural landscape video for relaxation priming in the BL and CL groups; (d) panel of judges video delivered to participants during the presentation treatment; (e) Survey Gizmo links for delivery of the experiment’s questionnaires. The only experimental tool that is missing is the S-Interface, which is held on a general purpose repository^24^, as it is software with broader applicability.

## Technical Validation

To ensure the soundness of the data, we carried out the following operations: (1) Quality control of study variables. (2) Experimental validation of study variables. We conducted hypothesis testing against a one tail-alternative in the NASA-TLX sub-scales and a two-tail alternative in all other cases; levels of significance were set at *α* = 0.05 designated by *, *α* = 0.01 designated by **, or *α* = 0.001 designated by ***. In physiological and performance variables we applied paired t-tests, with logarithmic corrections if the corresponding distributions had an exponential structure. In a few cases where normality assumptions were violated but variables did not have an exponential structure, we applied paired Wilcoxon signed-rank tests.

### Quality control of study variables

#### Biographic variables

Supplemental Fig. S2 shows distributions of biographic variables for the study participants. Supplemental Fig. S2a indicates that the great majority of the participants were young adults (23.75  ±  8.76 yr). This dovetails with Supplemental Fig. S2e, which reveals that the great majority of the participants were undergraduate students. Supplemental Fig. S2b shows that among the participants 45 were female (F) and 18 were male (M). We did not control for gender and thus the sample is not balanced, but still features a reasonable mix (70% F vs. 30% M). As an educated cohort of individuals, the participants appeared highly confident about their writing proficiency (Supplemental Fig. S2c). In addition, the overwhelming majority of the participants declared either frequent or very frequent use of daily email (Supplemental Fig. S2d). Since the experiment calls for report writing interspersed with email use, writing proficiency and email use are important participant attributes for a meaningful study. Hence, the biographic makeup of the participants is highly consistent with the intent of the experimental design.

#### Psychometric variables

We screened participants psychometrically via three inventories. Across the sub-scales of these inventories participant distributions feature a healthy spread (Supplemental Fig. S3), suggesting the presence of useful variability. Specifically:

- **Big Five Inventory or Big 5 - Supplemental Fig. S3a.** It measures five personality traits: agreeableness (detached vs. friendly), conscientiousness (careless vs. organized), extraversion (reserved vs. outgoing), neuroticism (confident vs. nervous), and openness (cautious vs. curious). Of particular interest in this experiment are conscientiousness, extraversion, and neuroticism traits. Indeed, organizational skills may play some role in the way people manage multi-tasking, while different extraversion and neuroticism levels may affect participant responses in the presentation task. The agreeableness distribution is at 35.39  ±  5.01 in a sub-scale that can range from 9 to 45; thus, it is shifted towards the high-end of the sub-scale, indicating that the majority of participants were agreeable individuals. The conscientiousness distribution is at 31.28  ±  5.41 in a sub-scale that can range from 9 to 45. The extraversion distribution is at 26.08  ±  6.53 in a sub-scale that can range from 8 to 40. The neuroticism distribution is at 22.11  ±  4.71 in a sub-scale that can range from 8 to 40; thus, it is centered near the middle of the sub-scale and characterized by absence of extremes, which would have confounded physiological responses. The openness distribution is at 37.41  ±  5.94 in a sub-scale that can range from 10 to 50; thus, it is shifted towards the high-end of the sub-scale, indicating that the majority of participants were open individuals.
- **Emotion Regulation Questionnaire or ERQ - Supplemental Fig. S3b.** It measures cognitive reappraisal and expressive suppression. The experimental design provides for four groups: Two groups (BH, CH) are told from the beginning they need to give a presentation after DT, while the other two groups (BL, CL) are advised about the presentation directly before the PR phase of the experiment. Hence, the ability to reframe an anticipated stressful situation in the first case, or deal with its surprising announcement in the second case, may affect participant responses and performance. Cognitive Reappraisal is at 32  ±  5.46 in a sub-scale that can range from 6 to 42; thus, it is shifted towards the high-end of the sub-scale, indicating a cohort of participants capable of controlling emotions via reframing. Expressive Suppression is at 14.34  ±  5.08 in a sub-scale that can range from 4 to 28.
- **Perceived Stress Scale or PSS - Supplemental Fig. S3c.** It measures how stressful the respondents find their lives. The PSS distribution is at 17.13 ± 5.68 in a scale that can range from 0 to 40; thus, it is centered toward the middle of the scale and characterized by absence of extremes that would have confounded physiological responses.

At the end of DT and prior to PR, we asked participants to complete the NASA Task Load Index (TLX) to measure the loading induced by the experiment’s main treatment. For the sub-scales of the NASA-TLX index, the score ranges are as follows: Mental Demand [1–7]; Physical Demand [1–7]; Temporal Demand [1–7]; Performance [1–7]; Effort [1–7]; Frustration [1–7]. Ranks from 1 to 4 indicate various degrees of trivial perceived loading, while ranks from 5 to 7 indicate various degrees of substantial perceived loading. For Performance, loading refers to sense of success. In Supplemental Fig. S4 the left column of plots shows the distributions of the responses in the seven-point Likert scales, while the right column shows the distributions of these responses when clustered in trivial vs. substantial loading subgroups. Testing if substantial loading are significantly larger than trivial loading subgroups suggests that the great majority of participants perceived DT as mentally challenging (*p* < 0.001, test of proportions in Mental Demand), for which they expended significant effort (*p* < 0.001, test of proportions in Effort), under time pressure (*p* < 0.001, test of proportions in Temporal Demand). These results confirm the success of the experimental design, which meant for DT to simulate a consuming office task. At the same time, the participants felt that did not expend any significant physical effort (*p* > 0.05, test of proportions in Physical Demand) - it was sedentary work after all. The participants also found the task to be non-frustrating (*p* > 0.05, test of proportions in Frustration).

#### Physiological variables

These variables constitute a set of peripheral physiological indicators of arousal that track levels of stress. The set includes perinasal perspiration, wrist EDA, breathing rate, and chest/wrist heart rate signals. We use up to two levels of quality control in curating data from physiological channels. Quality control level 1 (QC1) indicates the application of a specification filter. In the case of signals from physiological probes, their values are checked to ascertain they are within the specification range given by the sensor manufacturer. Signals that are found to have values out of range are discarded from the set. In the case of signals extracted algorithmically from physiological imagery, the image quality is examined to ascertain conformance to algorithmic assumptions.

In certain physiological channels, where additional information empowers a more detailed screening, quality control level 2 (QC2) follows QC1. In some instances, QC2 involves removal of signals plagued by excessive high frequency noise, which escaped algorithmic or electronic filtering. For the most part, however, QC2 takes place when there is a redundant channel modality, enabling congruency tests. A case in point is the presence of two synchronized heart rate channels in the dataset, one associated with a chest sensor and the other with a wrist sensor. Where temporally matched measurement pairs from such redundant channels are found to be incongruent, the most plausible values are kept, while their cross-modal paired measurements are discarded. Plausibility is assessed on the basis of background physiological knowledge and experimental context.

#### Perinasal perspiration signals

The left column of Supplemental Fig. S5 depicts the algorithmically extracted perinasal perspiration (PP) signal sets (see Methods section), while the right column depicts the down-selected PP signal sets after the application of quality control. Perspiration signal extraction from thermophysiological imagery is morphological in nature, quantifying the presence of active perspiration pores, which are tiny in size; thus, it critically depends on sharp focusing. Focus quality in facial thermal imaging sequences is assessed on the basis of edge contrast between the participants’ (cold) eyebrows and the surrounding (hot) tissue. We found that only two of the 314 PP signals were extracted from improperly focused thermal imagery; these signals were removed from the dataset.

#### EDA signals on wrist

Supplemental Fig. S6 shows the participants’ wrist EDA signals per treatment. The left column of this figure depicts the original signal sets, while the right column depicts the down-selected signal sets after the application of two levels of quality control. The first level of quality control (QC1) is based on valid range checking - it removes signals featuring at least one value outside the range [0.01,100] μS. Values outside this range violate the E4’s EDA sensor specification, suggesting weak electrodermal signal^26^ or other abnormality. We found 5.19% of the wrist EDA data to be below 0.01 μS; we found no EDA data to be above 100 μS. The said invalid values were spread over 54 signals, which were removed from further consideration. The second level of quality control (QC2) scans for patterns of excessive high frequency noise, suggestive of poor wristband fitting. We found five EDA signals to exhibit such patterns and removed them from the dataset (Supplementary Information: EDA graphs for T011-PM, T011-ST, T011-DT, T064-ST, and T096-DT).

#### Breathing rate signals on chest

Supplemental Fig. S7 shows the participants’ breathing rate signals per treatment. The left column of this figure depicts the original signal sets, while the right column depicts the down-selected signal sets after the application of quality control. Quality control, in this case, is based on valid range checking (QC1) - it removes signals featuring at least one value outside the range [4, 40] bpm. The Zephyr BioHarness 3.0 device cannot measure reliably breathing rates outside this range. Moreover, extreme breathing rates are unlikely for sitting subjects that are under mild to moderate stress. We found 0.22% of the breathing rate data to be below 4 bpm, while we did not find any data to exceed 40 bpm. These invalid values were spread over 12 signals, which were removed from further consideration. Breathing rate measurements in Zephyr BioHarness 3.0 are more resilient to disruptions with respect to heart rate measurements by the same device. This is due to the piezoelectric nature of the breathing sensor, where breathing signal formation tracks the expansion and contraction of the chest’s circumference. This operational principle ameliorates the effect of poor fitting and posturing, as measurement failures require significant strap deformation or detachment.

#### Heart rate signals on chest and wrist

Supplemental Figs S8 and S9 show the heart rate signals measured on the participants’ chest (via BioHarness) and non-dominant wrist (via E4), respectively. The left columns of these figures depict the original signal sets, while the right columns depict the down-selected signal sets after the application of quality control. Quality control in these cases proceeds in two stages - range checking (QC1) followed by cross-modality checking (QC2).

**QC1** The first stage of quality control is based on valid range checking (QC 1) - it removes signals featuring at least one value outside the range [40, 140] bpm. The Zephyr BioHarness 3.0 device cannot measure reliably heart rates outside this range. Moreover, extreme heart rates are unlikely for sitting subjects that are under mild to moderate stress. In Zephyr BioHarness 3.0 we found 9.90% of the heart rate data to be below 40 bpm and 0.01% of the heart rate data to exceed 140 bpm. These invalid data values were spread over 33 signals, which were removed from further consideration. One additional signal was removed because of a preponderance of missing values. In E4 we found no heart rate data to be below 40 bpm, while we found 0.11% of the data to exceed 140 bpm. These suspect data values were spread over seven signals, which were removed.

Overall, problems were of small to moderate scale and consistent with wearability limitations for BioHarness and E4^27^. Interestingly, violations of the lower bound were found exclusively in the chest heart rate signals, while violations of the upper bound were found only in the wrist heart rate signals. The distribution of the failure modes can be explained by the different measurement principles and body locations associated with the BioHarness and E4 heart sensors. The BioHarness performs heart rate measurements based on electrophysiological sensing and is sensitive to electrode detachment, which is likely in the thoracic area when participants crouch; it leads to catastrophic failures (see zero drops in Supplemental Fig. S8). E4 performs heart rate measurements based on plethysmography and is sensitive to probe contact force; overtight or loose wristbands lead to unreliable measurements. Even when the wristbands are loose, however, they do not result into loss of signal (no zero drops in Supplemental Fig. S9), because the heart rate measurements are effected via light beams - not electrodes.

**QC2** The redundancy in heart rate measurements affords the opportunity of a second stage quality control. Mean heart rate measurements on the chest and wrist should not be significantly different within participant and treatment - after all, people have only one heart! Hence, cross-modality comparisons can detect incongruent measurement pairs. A study of these incongruent pairs can identify the modality at fault in each case, leading to the removal of erroneous signals.

**Fig. 2:**
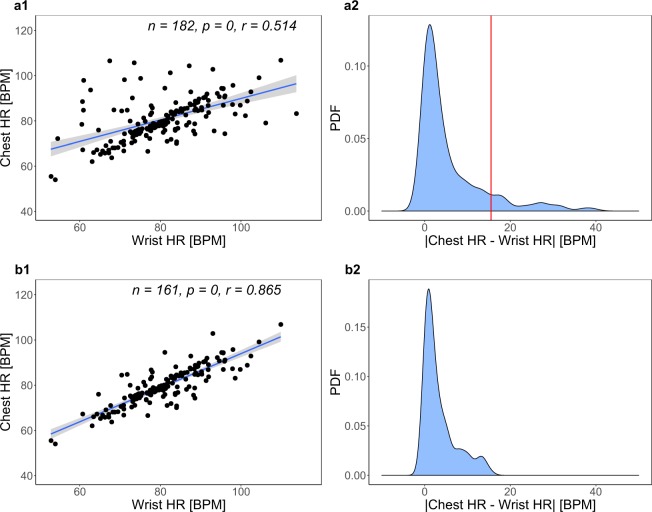
BioHarness (Chest HR) vs. E4 (Wrist HR) heart rate measurements. (**a1**) Regression of Chest HR on Wrist HR after QC1 but before QC2. (**a2**) Probability density function (PDF) of modality agreement/disagreement after QC1 but before QC2. The red line at 15.5 BPM demarcates the three normal modes constituting the extreme tail end of the distribution. (**b1**) Regression of Chest HR on Wrist HR after QC2. (**b2**) Probability density function (PDF) of modality agreement/disagreement after QC2.

In this direction, we studied how chest heart rate data regress on the corresponding wrist heart rate data. In the QC1 sets, we found significant association but no high linear correlation between the two modalities (*p* = 0, *r* = 0.514, Pearson correlation) - Fig. 2a1. Next, we studied the multinormal distribution of |ChestHR−WristHR| (Fig. 2a2) that quantifies the modes of agreement/disagreement between the two modalities. All secondary normal modes in the region |ChestHR−WristHR| > 10 contain noisy measurements that reduce the strength of the linear relationship between chest and wrist heart rate. We focused our attention on the normal modes occupying the tail region |ChestHR−WristHR| > 15.5, inspecting the 21 outliers contained therein. We determined that in all 21 cases the wrist heart rate was in error. The prevalent pattern was apparent overestimation by the wrist heart sensor in resting baseline (RB) and priming (PM) sessions. In these non-stressful treatments, E4 was reporting for certain participants heart rate measurements in excess of 100 BPM, featuring waveforms with aberrant variance - all telltale signs of an unsettled wristband. Examples of erroneous wrist heart rate signals that fall under the said pattern include T019-RB, T031-RB, T031-PM, T035-RB, and T035-PM (see Supplementary Information). We removed all such faulty measurements and recomputed the regression, revealing this time high linear correlation between the chest and wrist modalities (*p* = 0, *r* = 0.865, Pearson correlation) - Fig. 2b1.

Unavoidably, setting the cutoff threshold at 15.5 (instead of 10) in the probability distribution function (PDF) left a small amount of noise in the heart rate data (Fig. 2b2), which is hard to remove without introducing other errors. Indeed, as the absolute difference of the paired mean heart rate measurements gets smaller, entering the region 10 < |ChestHR−WristHR| ≤ 15.5, it becomes difficult to discern which modality is at fault.

#### Overview of quality control outcomes on physiological variables

**Fig. 3:**
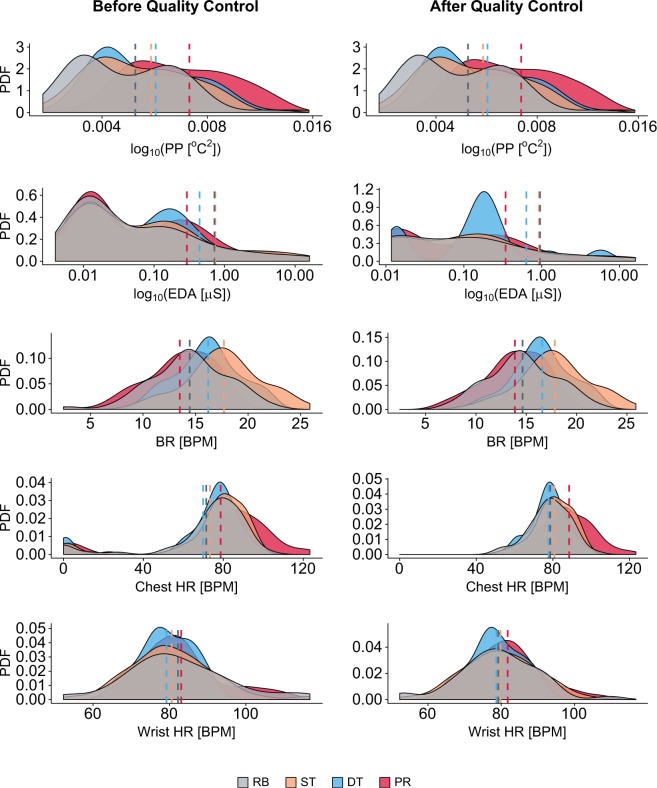
Probability density functions (PDF) of physiological signals before and after quality control for the key treatments. Signal data in the perinasal perspiration channel and in the breathing rate channel have passed QC1. Signal data in the EDA, Chest HR, and Wrist HR channels have passed QC 1 and QC2. Hyphenated lines indicate the corresponding distribution means.

Figure 3 depicts the probability density functions (PDF) for each physiological channel before and after quality control; the key treatments are included in each graph. In certain cases, the beneficial effect of quality control is evident. For instance, in Chest HR, the long left tails of the distributions in the original signal sets (left Chest HR panel) were removed (right Chest HR panel). At the same time, the stressful effect of presentation came into sharper focus, as its mean Chest HR moved further away from the means of the other treatments (right vs. left Chest HR panel).

Although prior to precise analysis, physiological measurements need to be normalized within participants to factor out inter-individual variability, the non-normalized PDF trends in the right column panels of Fig. 3 can provide soundness cues. Indeed, these trends need to conform to common-sense expectations with respect to the effects of the experimental design. In the current experiment, the mean arousal level in the resting baseline (RB) is expected to be the lowest, the mean arousal level in the presentation (PR) is expected to be the highest, and the mean arousal levels in the two writing treatments (ST and DT) are expected to be in-between. In this sense, the PP trends fully conform to expectations, suggesting a high quality physiological channel. In contradistinction, the wrist EDA channel presents non-sensical trends, which is worrisome but consistent with other reports in the literature^26^. The breathing rate channel captures well the stress effect of the writing treatments with respect to the resting baseline, but gives a non-sensical result for the presentation. In contrast, the two heart rate channels capture well the high stress effect of the presentation, but give an unclear picture regarding differences between the writing treatments and the resting baseline.

The complementarity between the heart rate and breathing rate variables could be abstracted in terms of task characteristics. Heart rate performs well in moderate office stressors with an emotional component, such as office presentations. The latter is fortunate, because tasks that involve speech are outside the operational envelope of the breathing rate channel, due to interference of speech respiratory patterns^28^. By contrast, heart rate performs poorly in mild office stressors of a cognitive nature (e.g., report writing), where thankfully breathing rate excels. This success of the breathing rate channel can be explained as follows: Cognitive tasks at the office are bereft of physical activity, and thus metabolic rate of office workers during report writing is defined by energy consumption in the brain. Respiration tracks metabolic rate^29^ and in turn breathing rate tracks respiration.

An additional cardiac variable that is considered a good tracker of sympathetic activation is heart rate variability (HRV)^30^. In this experiment HRV is measured via the BioHarness. HRV is expressed in various metrics^31^. Quality control and validity analysis of RR - the most fundamental HRV metric - is presented in Supplementary Information, suggesting that HRV mirrors the information provided by the Chest HR channel.

#### Performance variables - report scores and keystrokes

Two key office tasks in this experiment are the reports required of the participants in ST and DT. Hence, performance in these reports constitutes an important set of response variables. We scored the participant reports using the Educational Testing Service (ETS) *e-rater* scoring engine^23^. For each report, the *e-rater* produces a holistic score, called criterion score, which rates overall quality of writing. The *e-rater* also provides scores for four key writing traits - mechanics, grammar, usage, and style errors. Moreover, report content is entered using the keyboard, with keystroke information being captured in the Keyboard Data file. We can use this dynamic information to cross-check the static information in the report text, as manifested by the *e-rater* scores.

**Fig. 4:**
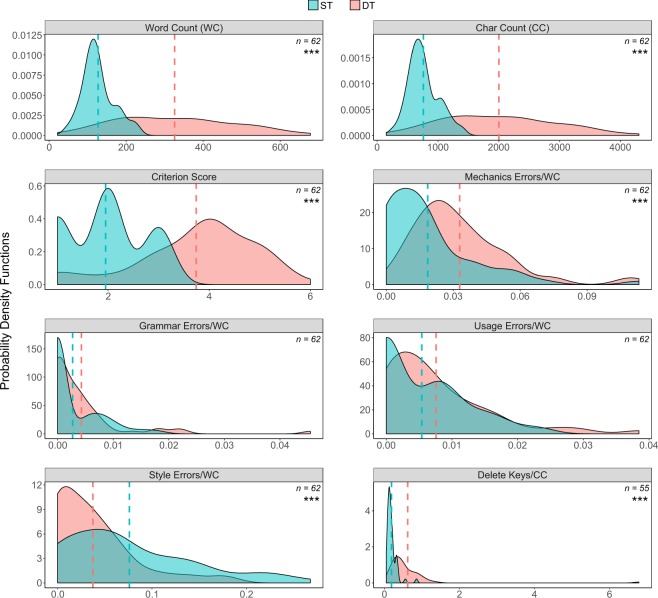
Report length, *e-rater* scoring distributions, and use of delete keys in ST and DT. Hyphenated lines indicate the corresponding distribution means. Star annotations or absence thereof in the figure’s panels refer to the results of the corresponding paired Wilcoxon signed-rank tests.

In Fig. 4, the distributional differences between ST and DT in word counts (WC), character counts (CC), criterion scores, mechanics errors, and style errors are visually apparent. To confirm these visual impressions we resort mostly to rank tests instead of t-tests because nearly all the distributions violate normality assumptions, as is also evident by their skewed or multinormal shape. In fact, we perform paired Wilcoxon signed-rank tests (rather than pooled Wilcoxon signed-rank tests), to safely account for inter-individual differences. Such hypothesis testing suggests the DT reports of participants have significantly higher quality than their ST reports (*p* < 0.001, paired Wilcoxon signed-rank test).

The two key differences between ST and DT lie in the absence vs. presence of multi-tasking and the limited vs. comfortable amount of report time, respectively. In ST, participants had only report writing to contend with, but they had to finish in 5 minutes. In DT, participants had to respond to emails while working on the report. Nevertheless, the session was longer, leaving them at least 20 minutes for report composition. These significantly different times resulted in significantly different report lengths. As Fig. 4 shows, DT reports have significantly larger mean word and character counts than ST reports (*p* < 0.001, paired Wilcoxon signed-rank test and t-test, respectively). The variances of word and character counts also differ significantly between ST and DT. There is only so much one can write in 5 minutes, resulting in short reports with similar lengths for ST (low variance). Given extra time in DT, several people take advantage of it writing significantly lengthier reports, while others do not (high variance).

For mechanics, grammar, usage, and style errors the *e-rater* reports absolute counts. To put these counts into perspective, we normalize them with respect to the corresponding report length, measured in number of words (Fig. 4). In this context, the participants perform significantly more mechanics errors in DT reports (*p* < 0.001, paired Wilcoxon signed-rank test), while they commit significantly more style errors in ST reports (*p* < 0.001, paired Wilcoxon signed-rank test). Given that we normalize for report length, one would expect that mechanics errors remain invariant within participants, as is the case for grammar and usage errors (*p* > 0.05, paired Wilcoxon signed-rank test in both cases). This would likely have been the case if the participants used a standard modern editor, such as Microsoft Word. However, because the participants used a basic editor without spell-checking capability, the mechanics errors result makes sense, adding to the validity of the data.

The error-ridden style in ST also makes sense, as participants did not have the time in that short session to thoughtfully style their sentences. This assertion is nicely supported by the dynamic information contained in the Keyboard Data file. As the last panel in Fig. 4 indicates, participants made far more extensive use of the delete keys in DT vs. ST (*p* < 0.001, paired Wilcoxon signed-rank test). Apparently, this extensive editing activity was funneled in restyling sentences, which dovetails with *e-rater’s* style scores.

### Experimental validation of study variables

Initial soundness indications for each physiological channel suggested by aggregate and non-normalized PDF trends (Fig. 3), need to be rigorously checked through validity testing. We choose to perform such validation within group and within treatment to account for the nuances of the experimental design.

#### Validity of PP as a stress tracker

Let **PP**(*S*_*i*_, *G*_*j*_, *T*_*k*_) represent the perinasal perspiration signal of participant *S*_*i*_, in group *G*_*j*_, for treatment *T*_*k*_, where *i* ∈ {1, …, 63}, *j* ∈ {BH, BL, CH, CL}, and *k* ∈ {ST, Stroop, RV, DT, PR}, respectively. In each group *G*_*j*_, we normalize within participants the expected PP values by computing the distributions of paired differences between the participants’ mean **PP** in *T*_*k*_ and *T*_RB_:

1 $$\Delta \bar{{\bf{P}}{\bf{P}}}(\cdot ,{G}_{j},{T}_{k})=\bar{{\bf{P}}{\bf{P}}}(\cdot ,{G}_{j},{T}_{k})\,{[}^{{}^{\circ }}{C}^{2}]-\bar{{\bf{P}}{\bf{P}}}(\cdot ,{G}_{j},{T}_{RB})\,{[}^{{}^{\circ }}{C}^{2}].$$

**Fig. 5:**
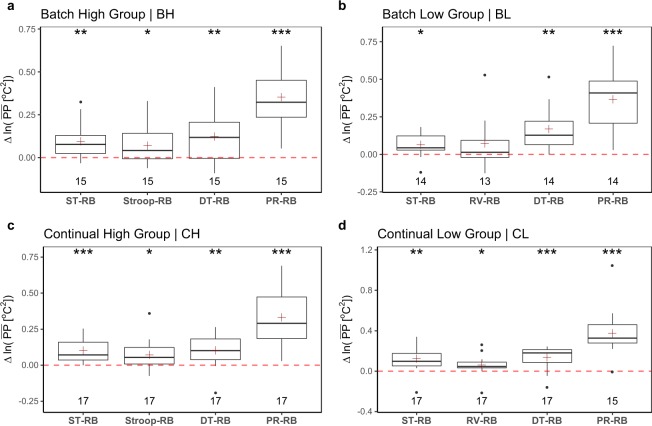
Experimental validity of the perinasal perspiration channel. It performs well across the board. We used the ln(⋅) transformation to comply with normality assumptions.

Equation (1) produces the boxplots in Fig. 5. The NULL hypothesis is that arousal within participants in treatments ST, Stroop, DT, and PR is no different than arousal in resting baseline RB. The results indicate that perinasal perspiration captures well the stressful effect of all these treatments across groups - a clear sign of a high quality data channel. Specifically, in ST (*p* < 0.05 or better, paired t-test in all groups); in Stroop (*p* < 0.05, paired t-test in all applicable groups); in DT (*p* < 0.01 or better, paired t-test in all groups); and in PR (*p* < 0.001, paired t-test in all groups).

#### Validity of EDA as a stress tracker

Let **EDA**(*S*_*i*_, *G*_*j*_, *T*_*k*_) represent the wrist EDA signal of participant *S*_*i*_, in group *G*_*j*_, for treatment *T*_*k*_, where *i* ∈ {1, …, 63}, *j* ∈ {BH, BL, CH, CL}, and *k* ∈ {ST, Stroop, RV, DT, PR}, respectively. In each group *G*_*j*_, we normalize within participants the expected EDA values by computing the distributions of paired differences between the participants’ mean **EDA** in *T*_*k*_ and *T*_RB_:

2 $$\Delta \bar{{\bf{E}}{\bf{D}}{\bf{A}}}(\cdot ,{G}_{j},{T}_{k})=\bar{{\bf{E}}{\bf{D}}{\bf{A}}}(\cdot ,{G}_{j},{T}_{k})\,[\mu S]-\bar{{\bf{E}}{\bf{D}}{\bf{A}}}(\cdot ,{G}_{j},{T}_{RB})\,[\mu S].$$

**Fig. 6:**
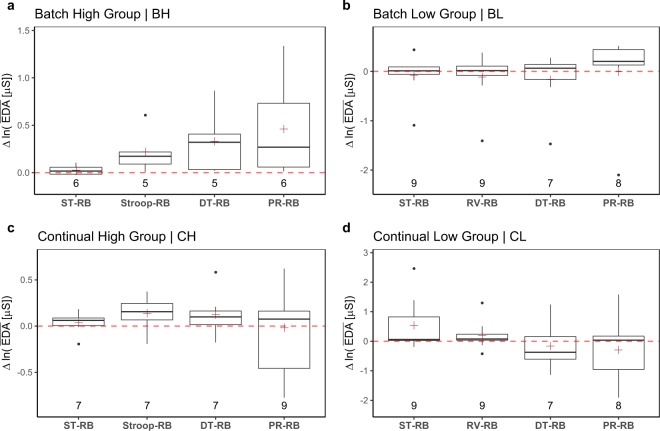
Experimental validity of the EDA channel. It performs poorly across the board. We used the ln(⋅) transformation to comply with normality assumptions.

Equation (2) produces the boxplots in Fig. 6. The NULL hypothesis is that arousal within participants in treatments ST, Stroop, DT, and PR is no different than arousal in resting baseline RB. The EDA results indicate that there was no significant elevation of arousal within participants for any of these treatments (*p* > 0.05, paired t-tests in all cases). This suggests that the EDA channel cannot track participants’ stress condition and its use in analytics with respect to this experiment should best be avoided.

One reason for EDA’s subpar performance is the small *n* numbers left in each group to partake in the validity tests. This is partly due to loss of data because of technical problems, and partly due to signal rejections in QC1. The latter took place because a significant number of signals exhibited values lower than the sensor’s specification. Taking also into account that these cases were characteristic of certain participants, suggests that a portion of the sample had very weak electrodermal responses in the wrist, where the E4 sensor was attached. This outcome is consistent with other reports in the literature^26^, leaving perinasal perspiration as the only viable cholinergic channel in the present experiment.

#### Validity of BR as a stress tracker

Let **BR**(*S*_*i*_, *C*_*j*_, *T*_*k*_) represent the breathing rate signal of participant *S*_*i*_, in group *G*_*j*_, for treatment *T*_*k*_, where *i* ∈ {1, …, 63}, *j* ∈ {BH, BL, CH, CL}, and *k* ∈ {RB, ST, Stroop, RV, DT, PR}, respectively. In each group *G*_*j*_, we normalize within participants the expected BR values by computing the distributions of paired differences between the participants’ mean **BR** in *T*_*k*_ and *T*_RB_:

3 $$\Delta \bar{{\bf{B}}{\bf{R}}}(\cdot ,{G}_{j},{T}_{k})=\bar{{\bf{B}}{\bf{R}}}(\cdot ,{G}_{j},{T}_{k})\,[\text{BPM}]-\bar{{\bf{B}}{\bf{R}}}(\cdot ,{G}_{j},{T}_{RB})\,[\text{BPM}].$$

**Fig. 7:**
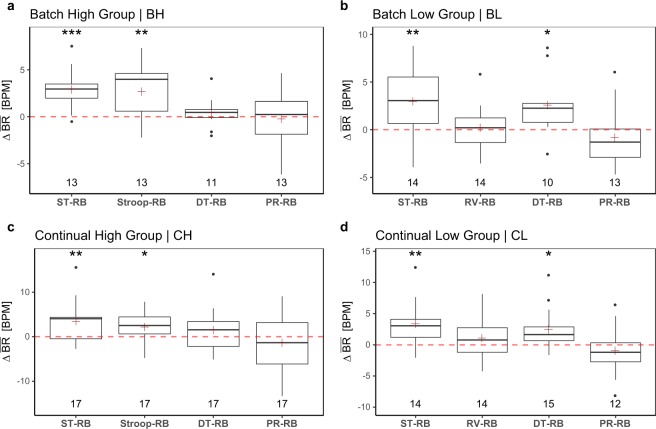
Experimental validity of the breathing rate channel. It largely captures the stressful effect of ST and DT, but not of PR.

Equation (3) produces the boxplots in Fig. 7. The NULL hypothesis is that arousal within participants in treatments ST, Stroop, DT, and PR is no different than arousal in resting baseline RB.

The results indicate that breathing rate captures well the stressful effect of ST across groups (*p* < 0.001, paired t-test in group BH and *p* < 0.01, paired t-tests in groups BL, CH, CL). Breathing rate also captures the stressful effect of Stroop (*p* < 0.01, paired t-test in group BH and *p* < 0.05, paired t-test in group CH). In DT, the performance of the breathing channel deteriorates, capturing stress effects only for groups BL and CL (*p* < 0.05, paired t-tests in groups), while missing them in groups BH and CH (*p* > 0.05, paired t-tests in groups). The breathing channel altogether misses stressful effects in PR (*p* > 0.05, paired t-tests for all cases). This disappointing performance in PR can be explained by breathing modulation incurred during speech. Hence, the BR channel should not be used in analytics with respect to the PR treatment. However, BR can be used in analytics with respect to all other treatments.

#### Validity of chest HR as a stress tracker

Let  Chest HR ≡ **HR**_**C**_(*S*_*i*_, *G*_*j*_, *T*_*k*_) represent the chest heart rate signal of participant *S*_*i*_, in group *G*_*j*_, for treatment *T*_*k*_, where *i* ∈ {1, …, 63}, *j* ∈ {BH, BL, CH, CL}, and *k* ∈ {ST, Stroop, RV, DT, PR}, respectively. In each group *G*_*j*_, we normalize within participants the expected HR_C_ values by computing the distributions of paired differences between the participants’ mean **HR**_**C**_ in *T*_*k*_ and *T*_RB_:

4 $$\Delta {\overline{{\bf{H}}{\bf{R}}}}_{{\bf{C}}}(\cdot ,{G}_{j},{T}_{k})={\overline{{\bf{H}}{\bf{R}}}}_{{\bf{C}}}(\cdot ,{G}_{j},{T}_{k})\,[\text{BPM}]-{\overline{{\bf{H}}{\bf{R}}}}_{{\bf{C}}}(\cdot ,{G}_{j},{T}_{RB})\,[\text{BPM}].$$

**Fig. 8:**
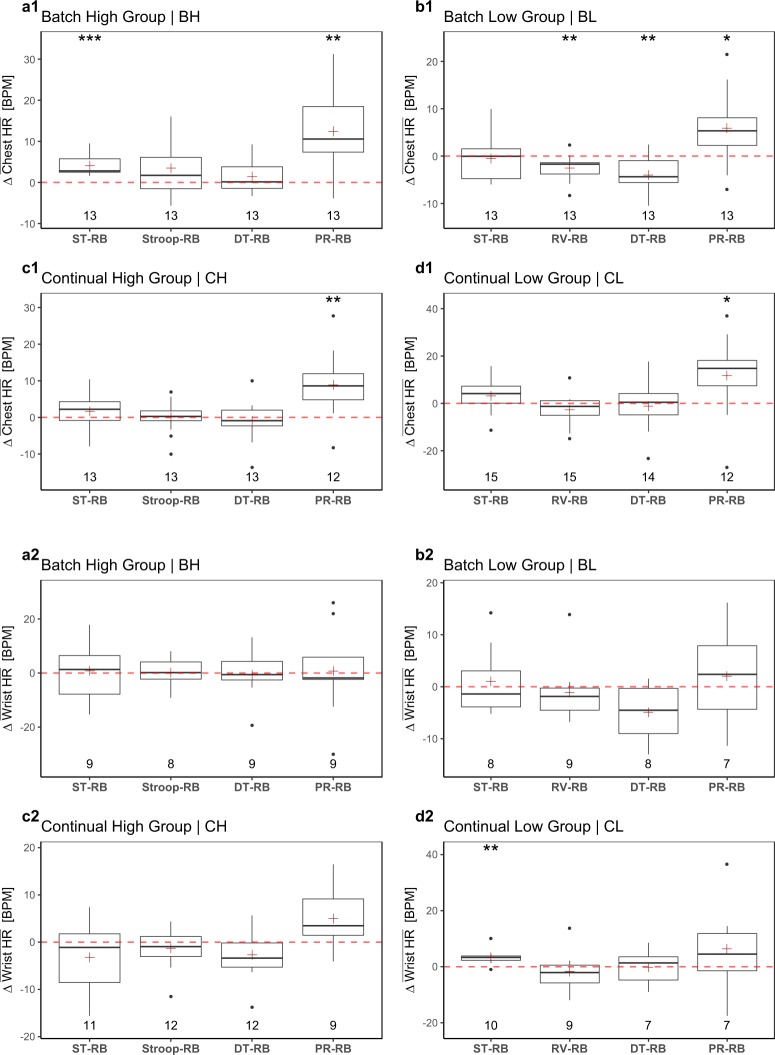
Experimental validity of the heart rate channels. (**a1–d1**) Experimental validity of the chest heart rate channel. It captures the stressful effect of PR, but largely misses it in ST and DT. (**a2–d2**) Experimental validity of the wrist heart rate channel. It performs poorly across the board.

Equation (4) produces the boxplots in Fig. 8a1–d1. The NULL hypothesis is that arousal within participants in treatments ST, Stroop, DT, and PR is no different than arousal in resting baseline RB.

The results indicate that heart rate when measured in the chest captures well the stress effect of PR (*p* < 0.01, paired t-test in BH, paired Wilcoxon signed-rank test in CH and *p* < 0.05, paired t-tests in BL, CL). However, the chest heart rate performance is disappointing in all the other treatments. Specifically, it captures the stressful effect of ST only in one group (*p* < 0.001, paired t-test in BH), while misses it in the other three groups (*p* > 0.05, paired t-tests in BL, CL, paired Wilcoxon signed-rank test in CH). Similarly, it captures the stressful effect of DT only in one group (*p* < 0.01, paired t-test in BL), while misses it in the other three groups (*p* > 0.05, paired t-tests in BH, CL, paired Wilcoxon signed-rank test in CH). It altogether misses the stressful effect of Stroop (*p* > 0.05, paired t-test in BH, paired Wilcoxon signed-rank test in CH). Based on these results, the chest heart rate channel could be considered as a complementary adrenergic channel to breathing rate. Indeed, while the chest heart rate channel appears to be a valid stress tracker in presentation tasks, the breathing rate channel appears to be a valid stress tracker for most other office tasks, except those involving speech delivery.

#### Validity of wrist HR as a stress tracker

Let Wrist HR  ≡ **HR**_**W**_(*S*_*i*_, *G*_*j*_, *T*_*k*_) represent the wrist heart rate signal of participant *S*_*i*_, in group *G*_*j*_, for treatment *T*_*k*_, where *i* ∈ {1, …, 63}, *j* ∈ {BH, BL, CH, CL}, and *k* ∈ {ST, Stroop, RV, DT, PR}, respectively. In each group *G*_*j*_, we normalize within participants the expected HR_W_ values by computing the distributions of paired differences between the participants’ mean **HR**_**W**_ in *T*_*k*_ and *T*_RB_:

5 $$\Delta {\overline{{\bf{H}}{\bf{R}}}}_{{\bf{W}}}(\cdot ,{G}_{j},{T}_{k})={\overline{{\bf{H}}{\bf{R}}}}_{{\bf{W}}}(\cdot ,{G}_{j},{T}_{k})\,[\text{BPM}]-{\overline{{\bf{H}}{\bf{R}}}}_{{\bf{W}}}(\cdot ,{G}_{j},{T}_{RB})\,[\text{BPM}].$$

Equation (5) produces the boxplots in Fig. 8a2–d2. The NULL hypothesis is that arousal within participants in treatments ST, Stroop, DT, and PR is no different than arousal in resting baseline RB.

The wrist heart rate results indicate that there was no significant elevation of arousal within participants for any of these treatments (*p* > 0.05, paired t-tests in BH, CH, CL cases, paired Wilcoxon signed-rank test in BL case). This suggests that the wrist heart rate channel cannot track participants’ stress condition and its use in analytics with respect to this experiment should be avoided.

A likely reason for wrist heart rate’s subpar performance is the small *n* numbers left in each group to partake in the validity tests. This is partly due to loss of data because of technical problems, and partly due to signal rejections in QC2. As analyzed in an earlier section, the latter take place because some Wrist HR signals found to overestimate with respect to the corresponding Chest HR signals.

## Usage Notes

### Usage of S-Interface

The dataset includes both the thermal sequences (primary data), and the perinasal perspiration signals that were extracted from these sequences. If one is interested to extract the perinasal signals anew, s/he has to use the S-Interface software^24^, selecting the (outer) tracking and (inner) measurement region of interest (MROI) in the first frame of each .dat file. The perinasal MROI is bound at the top by the subject’s nostrils, at the bottom by the subject’s lips, and on the left and right sides by the ends of the subject’s mouth. Based on this initial selection, the tracker is capable of following up this tissue area for the duration of the session, giving the chance to the physiological signal extractor to operate on a valid data set. In rare instances, the tracker momentarily fails. This happens when, for example, the participant performs a very abrupt head turn. The end result of such momentary failures are spikes in the perinasal signal. These spikes are removed by applying a noise-reduction algorithm reported in^32^. This algorithm is included in the S-Interface thermal imaging configuration. In even rarer instances, the tracker drifts away from the perinasal area. This typically happens when the subject has turned her/his head at an extreme angle and stayed there for some time. The user can reposition the tracker by simply clicking the mouse in the perinasal area. The tracker is restored and the signal extraction process resumes from that point onward on the right footing.

### Usage of R scripts

In the paper’s GitHub repository (Zaman, S. & Pavlidis, I. Office-Tasks-2019-Methods. *GitHub* https://github.com/UH-CPL/Office-Tasks-2019-Methods), there is detailed description of the R scripts that generate out of the raw data the curated data and validate them. Analysts who are content with our quality control and validation processes can use the curated dataset in^18^. However, in case analysts are interested to extract more information form the raw data, they can resort to the GitHub repository (Zaman, S. & Pavlidis, I. Office-Tasks-2019-Methods. *GitHub* https://github.com/UH-CPL/Office-Tasks-2019-Methods) and intervene in the scripts. For example, some analysts may decide to not eliminate from consideration Chest HR signals with zero drops, but keep them after reducing their noise levels.

## Supplementary information

Supplementary Information.

## Data Availability

**Data acquisition interface:** We use S-Interface, a modular software system available on figshare^24^ that supports real-time data acquisition from sensors and computer peripherals. S-Interface’s imaging subsystem reads radiometric data from the thermal facial camera and applies certain operations on them to extract perinasal perspiration (PP) signals; it also acquires the facial and operational theater recordings from the visual cameras. S-Interface’s participant input subsystem captures the participants’ computer interactions. Specifically: S-Interface’s imaging configuration runs on the system computer and includes: 1. The tracker plug-in (an implementation in C# of ref.^17^), which follows the participants’ perinasal area in the thermal imagery, nullifying the effect of head motion. 2. The perspiratory morphological signal extractor (an implementation in C# of ref.^15^), which operates upon the orthorectified perinasal MROI, yielding a signal commensurate to the extent of activated perspiration pores. This perspiration signal serves as a proxy of arousal. 3. The visual facial and operational theater plug-ins that capture the corresponding video streams. S-Interface’s participant input configuration runs on the participant computer and includes: 1. The screen capture plug-in, which calls upon the VisioForge SDK to record the participants’ computer display. 2. The keyboard plug-in, which captures the participants’ interactions with the computer’s input device. **Protocol interface**: To enforce consistency in the execution of the experiment and the delivery of the stressors, we developed a custom interface (p-Interface). The p-Interface implements the experimental protocol, guiding participants step by step through the designed treatments. Specifically, in the ST and DT treatments, the p-Interface presents to the participants a basic editor to write their reports. It also features a basic email client to deliver the email interruptions and allow participants to send back their responses. The p-Interface is written in Javascript and is available under the Tools folder in^18^. **Survey interface**: We developed an interface to deliver questionnaires to participants and collect their responses. This survey interface is implemented via surveygizmo and is available under the Tools folder in^18^. **Stroop application**: We wrote an application to deliver the Stroop color word test for the BH and CH groups. This application is presented to the participants from within the p-Interface. The Stroop application is written in Javascript and is available under the Tools folder in^18^. **Data curation, quality control, and validation scripts**: We developed four sets of R scripts to curate, ensure quality control, and validate the raw data collected via the S-Interface and other tools in this project. Both the scripts and the raw data reside in GitHub (Zaman, S. & Pavlidis, I. Office-Tasks-2019-Methods. *GitHub* https://github.com/UH-CPL/Office-Tasks-2019-Methods). The first set of scripts curates the raw data. The second set of scripts operates upon the curated data, performing the first level of quality control (QC1). The third set of scripts operates upon the QC1 data, performing a second level of quality control (QC2). The fourth set of scripts carries out validity tests on the QC2 data. The final outcomes of these script sets are the quality controlled data residing in^18^, as well as the statistical plots featured in the present manuscript and its Supplementary Information file. Some additional information about relevant practical issues can be found in^33^.
