# Supplementary material for: Stress and productivity patterns of interrupted, synergistic, and antagonistic office activities
Source: Sci Data. 2019 Nov 8;6:264. doi: 10.1038/s41597-019-0249-5 (PMC6841929; doi:10.1038/s41597-019-0249-5)
Supplement: Supplementary file 1 — Supplementary Information. [file 41597_2019_249_MOESM1_ESM.pdf]

Supplementary Information

Stress and productivity patterns of interrupted, synergistic, and antagonistic office activities

Shaila Zaman[1], Amanveer Wesley[1],
Dennis Rodrigo Da Cunha Silva[2], Pradeep Buddharaju[1],
Fatema Akbar[3], Ge Gao[4], Gloria Mark[3],
Ricardo Gutierrez-Osuna[2], Ioannis Pavlidis[1*]

October 24, 2019

1. Computational Physiology Laboratory, University of Houston 2. Perception, Sensing, and Instrumentation Laboratory, Texas A & M University 3. Department of Informatics, University of California, Irvine 4. College of Information Studies, University of Maryland

*corresponding author: Ioannis Pavlidis (ipavlidis@uh.edu)

Contents

1. Imaging Methods **3**

2. Summary Biographic and Psychometric Plots **4**

3. Summary Quality Control Plots **7**

4. Report Prompts **12**

5. Email Prompts **13**

6. Heart Rate Variability (HRV) **15**

7. Quality Controlled 1 Chest HR, Wrist HR, and EDA Signals **18**

1. Imaging Methods

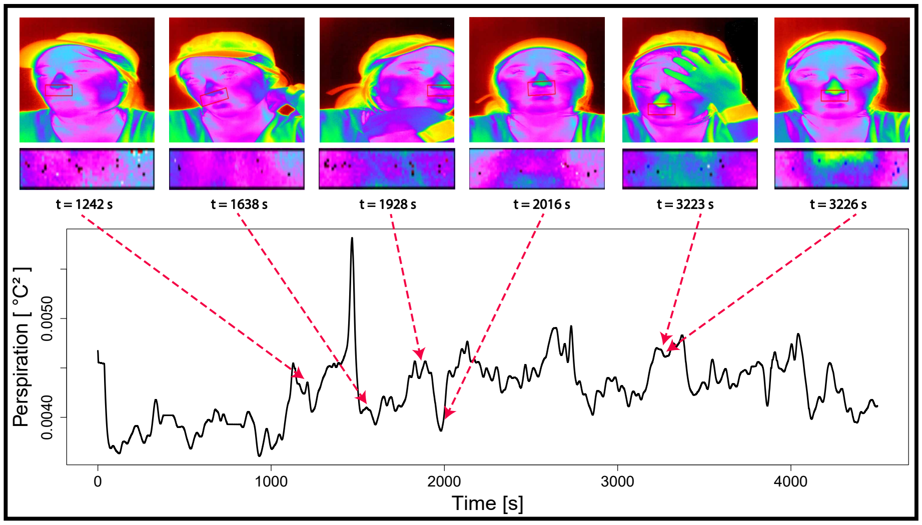

Figure S1: **Extraction of perinasal perspiration for participant T005. TOP:** Motion tracking (Ref. [17]) of the perinasal area (red rectangle). **MIDDLE:** The perinasal signal is extracted from the upper orbicularis oris region - a subset of the perinasal area. Black dots within the measurement region manifest active perspiration pores detected algorithmically (Ref. [15]). **BOTTOM:** The algorithm turns this spatial perspiration pattern into a signal by applying a morphological filter. Elevations in the signal correspond to densification of active perspiration pores, characterizing overarousal bouts.

2. Summary Biographic and Psychometric Plots

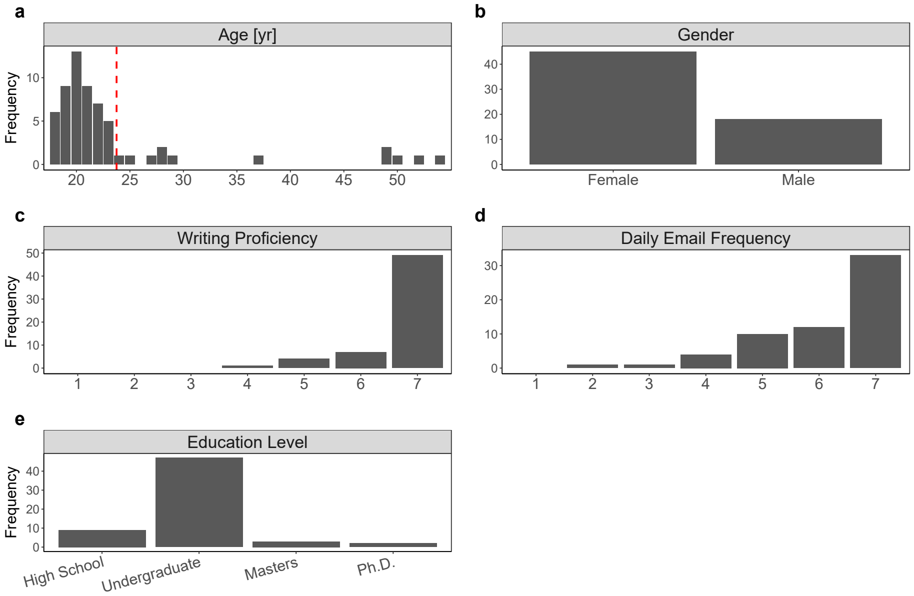

Figure S2: **Biographic variables.** **a.** Age distribution of participants. **b.** Gender distribution of participants. **c.** Participants' self-assessment of writing proficiency in a seven-point Likert scale, where $1 \equiv$ Not Fluent At All and $7 \equiv$ Very Fluent. All participants were native English speakers or bilingual. **d.** Participants' self-reported daily use of email in a seven-point Likert scale, where $1 \equiv$ Never and $7 \equiv$ Very Often. **e.** Participants' reported education levels.

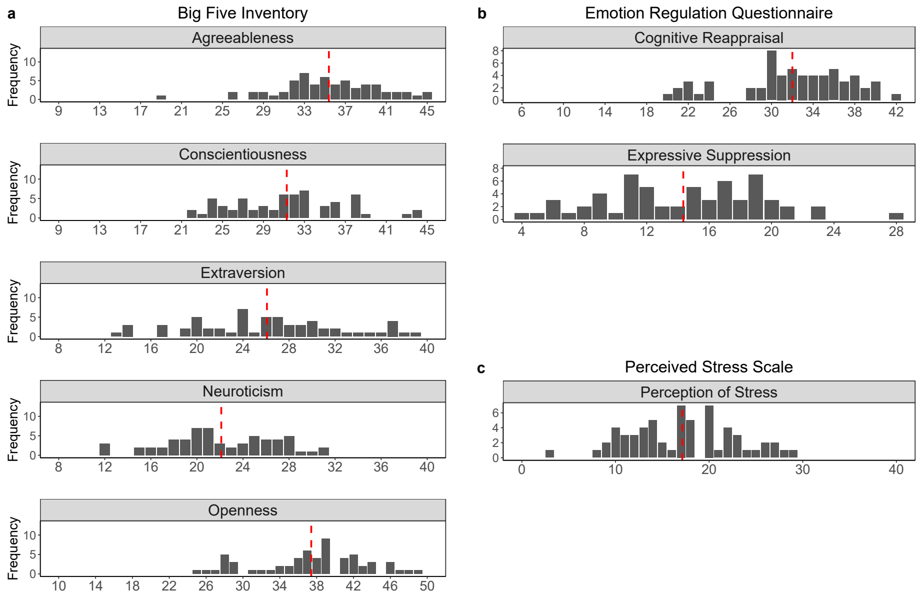

Figure S3: **Trait psychometric variables; red dashed lines indicate means. a.** Big Five Inventory distributions. **b.** Emotion Regulation Questionnaire distributions. **c.** Perceived Stress Scale distribution.

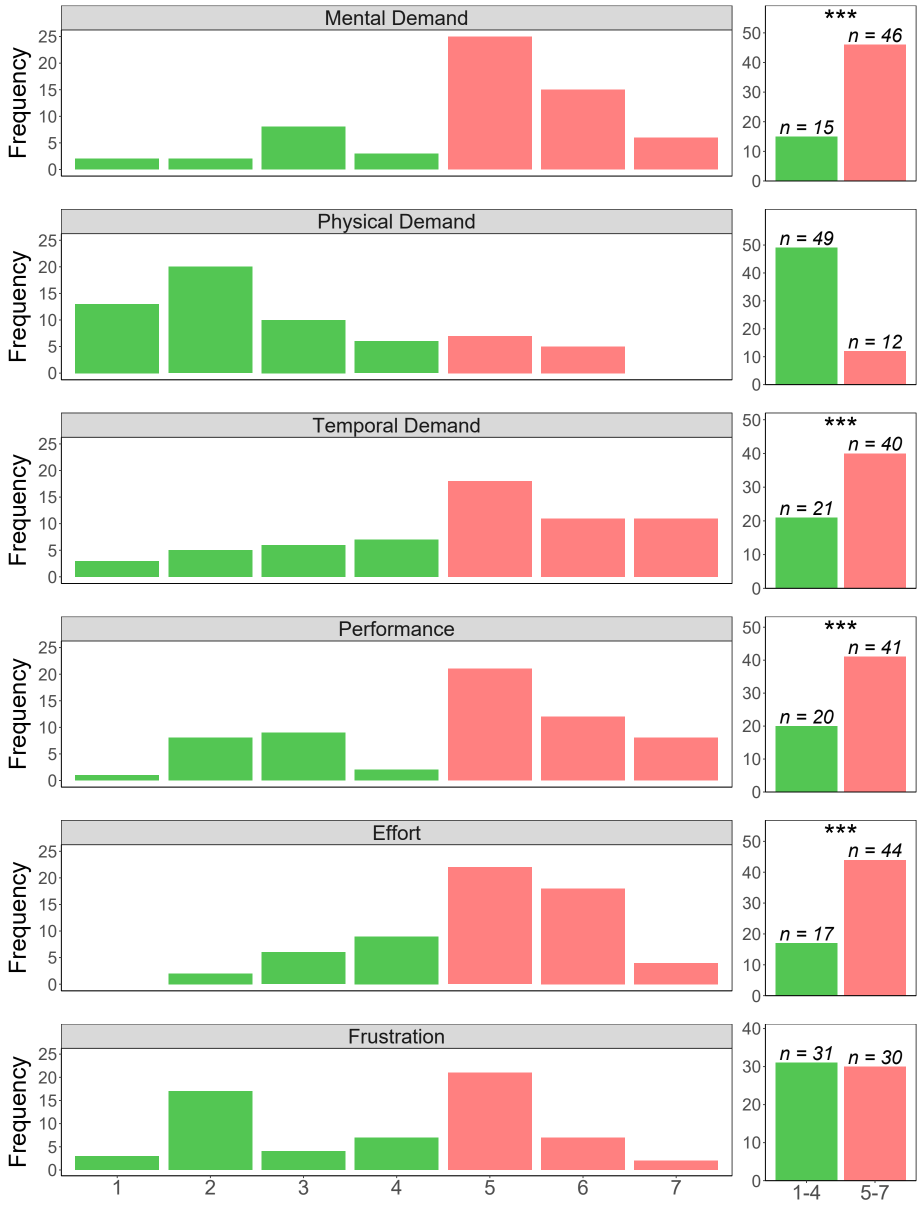

Figure S4: **NASA TLX responses following DT. LEFT:** Distributions of seven-point Likert sub-scales. **RIGHT:** Binarization of sub-scales to trivial loading (1-4) vs. substantial loading (5-7) clusters.

3. Summary Quality Control Plots

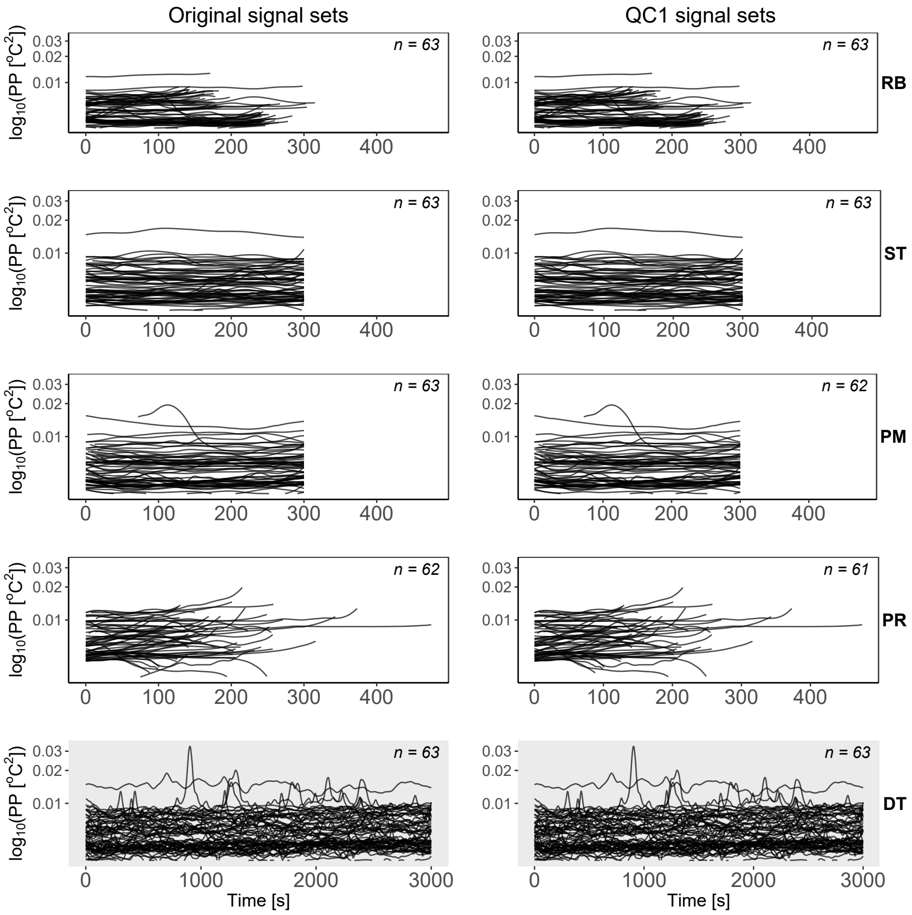

Figure S5: **Sets of perinasal perspiration (PP) signals before and after quality control (QC1).** PP signals, much like all cholinergic signals, are of exponential nature [33]. Hence, a $\log_{10}$ transformation was applied to facilitate visualization.

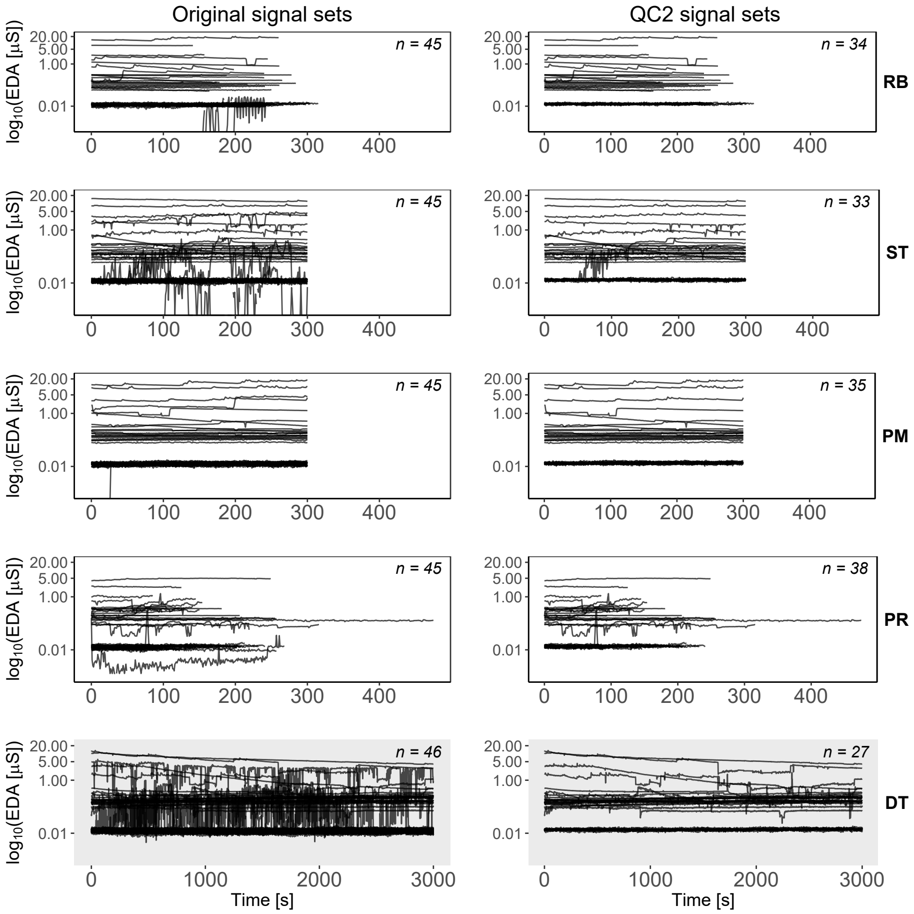

Figure S6: **Sets of EDA signals before and after two levels of quality control.** EDA signals, much like all cholinergic signals, are of exponential nature [33]. Hence, a $\log_{10}$ transformation was applied to facilitate visualization.

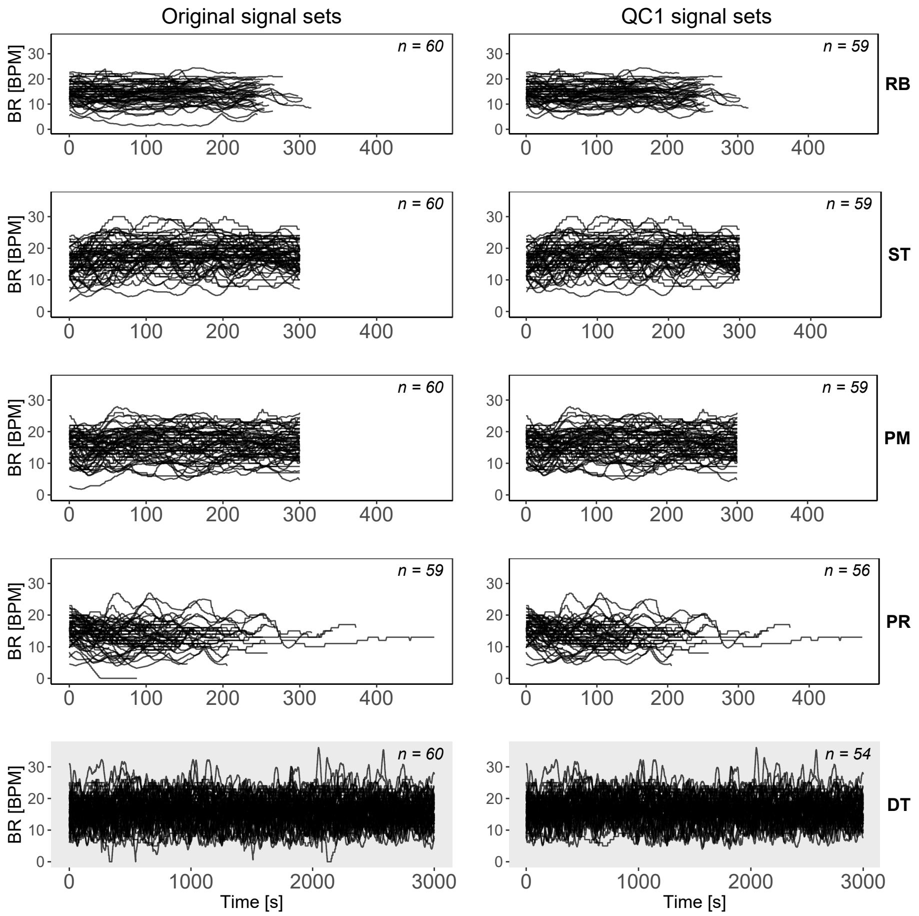

Figure S7: **Sets of breathing rate signals before and after quality control (QC1).** There is limited evidence of measurement disruptions in the original signal sets.

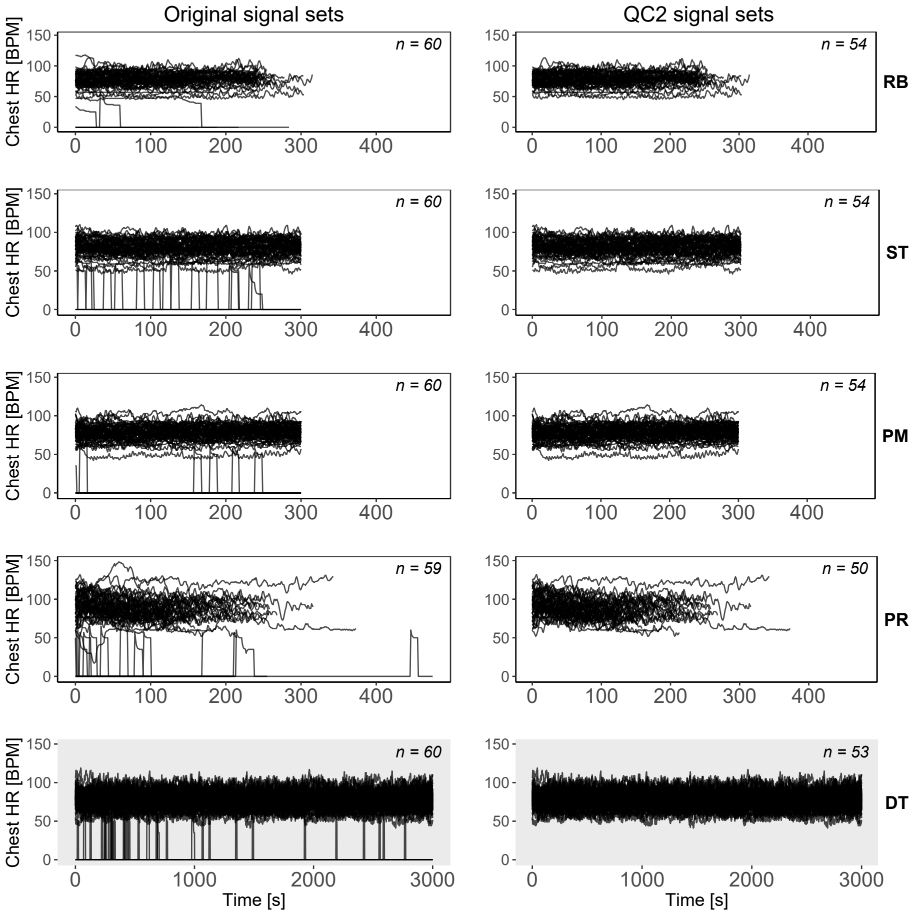

Figure S8: **Sets of heart rate signals measured on the chest before and after two levels of quality control.** There is evidence of measurement disruptions (i.e., zero drops) in certain cases of the original signal sets.

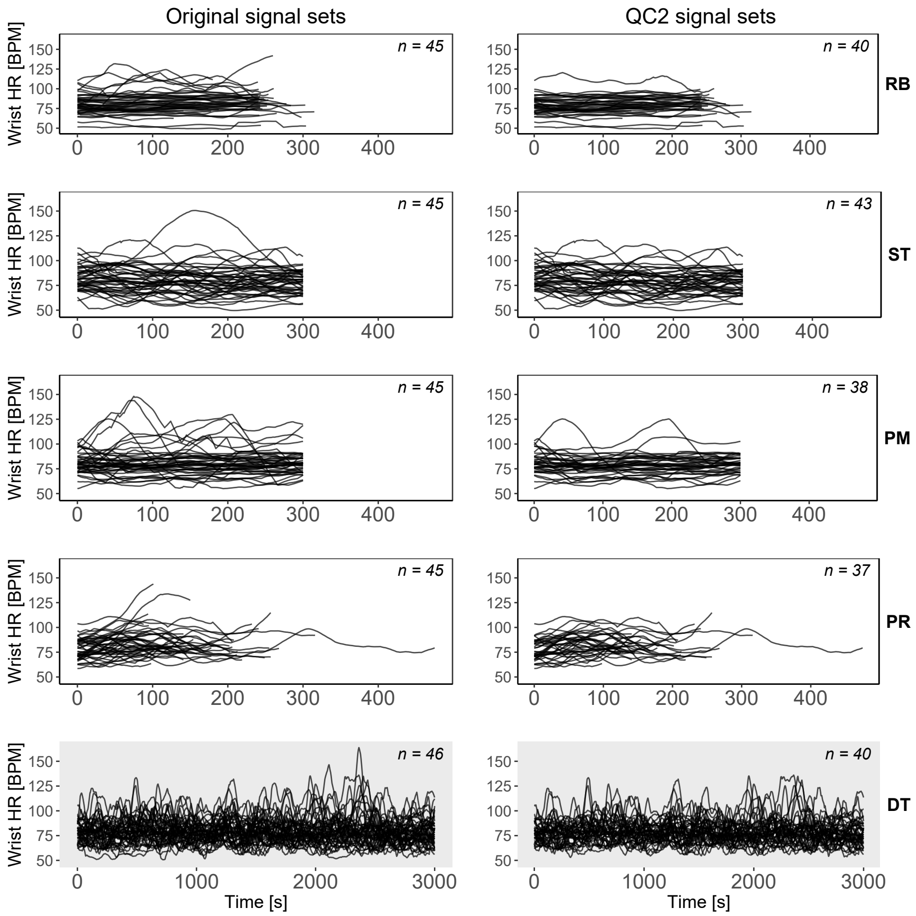

Figure S9: **Sets of heart rate signals measured on the wrist before and after two levels of quality control.** There is limited evidence of measurement disruptions in the original signal sets.

4. Report Prompts

Single Task (ST) Report Prompt
You have 5 minutes to write about the topic below:
The best way for a society to prepare its young people for leadership in government, industry, or other fields is by instilling in them a sense of cooperation, not competition. Do you agree or disagree? Explain your reasoning in detail.

Dual Task (DT) Report Prompt
You have 50 minutes to write. You will be given a topic. You can research the topic on the web. Please do not watch videos on this topic, but rather use written documents on the subject.

version for **H**igh Stress Groups

Your performance will be based on the quality of your report, the quality of your responses to email, and the quality of a 5-minute presentation you will give to a group of evaluators at the end.

version for **L**ow Stress Groups

Your performance will be based on both the content of your report and the relevance and sufficiency of your responses to emails.

Paragraph 1: Explain what the Technological Singularity is in your own words.

Paragraph 2: Explain & discuss the view of your first Technological Singularity theorist.

Paragraph 3: Explain & discuss the view of your second Technological Singularity theorist.

Paragraph 4: Explain and discuss your own view of Technological Singularity.

Paragraph 5: Conclude your essay summarizing the views of the theorists and your own.

NOTE: Copying and pasting in the essay window are disabled.

5. Email Prompts

NOTE: The numbering of emails does not imply order. Emails were delivered to participants in a random order to have a counterbalanced design. The numbering here merely indexes the participant responses contained in 'Reports and Emails.xlsx' of the Open Science Framework (OSF) repository (Ref. [18]).

Email 1

Title: Traveling tips

From: Jacob Fennelbroth

I need to plan a domestic trip soon, and it will be my first domestic flight in the US, as I am a new international student. What advice would you give me regarding this trip? and do you have personal experience with traveling domestically that you can share?

Email 2

Title: College, career, and finances

From: Kount Kristo

Several years ago, I had to delay my college education for financial reasons and get a job to support myself. Now, I am considering reapplying to school to complete my education and progress in my career. I am skeptical about going to a 4 year undergrad program as a 40 year old, but I have to do it. I am undecided between the state university and a smaller community college. Can you share any advice or tell me what it's like for others who have decided to continue their education? Any advice/information to help me make a decision would be great.

Email 3

Title: How well can we all be?

From: Mustafa Akulker

To help us collect some information to design an emotional well-being program, could you please answer the following questions: If someone you cared about lied to you, would you be angry? Why or why not? Have you ever lied to someone you cared about? Is it reasonable to expect other people to be honest with you, in all circumstances? What are the exceptions?

Email 4

Title: Career options and internships

From: Marcus Lance

I am planning on applying for a summer internship at a tech company. I am a sophomore and wondering when it is good time to start applying for summer internships. What qualifications do you think a tech company would usually look for an intern? Would you mind sharing any experiences or advice?

Email 5

Title: Finding a balance

From: Jamall Johnson

My friend always says I never take time to have fun because I'am working all the time. Is that bad? What do you think? I am looking for advice regarding work/study balance, so I thought I would reach out to you.

Email 6
Title: I need help scheduling a meeting
From: Petre Hinkledge
Please find a time slot for a student, administrator, and professor to have an hour long meeting, in a conference room that can seat 3 people. People's schedules are presented on the first tab of the linked spreadsheet, and room availability is in the second.

People have two types of availability: completely free (shown in white) and possible commitments (shown in yellow), which can be moved if necessary. You should not schedule a meeting in unavailable time slots (shown in red). Scheduling decisions should minimize the number of possible commitments rescheduled, and prioritize the schedule of the professor, then the administrator, then, lastly, the student. Reply to this email with the meeting start time and room you select.

Email 7
Title: Planing a meeting
From: Svetlana Romanoff
Please find a time slot for a student, administrator, and professor to have an hour long meeting in a conference room that can seat 3 people. People's schedules are presented on the first tab of the linked spreadsheet, and room availability is in the second.

People have two types of availability: completely free (shown in white) and possible commitments (shown in yellow), which can be moved if necessary. You should not schedule a meeting in unavailable time slots (shown in red). Scheduling decisions should minimize the number of possible commitments rescheduled, and prioritize the schedule of the professor, then the administrator, then, lastly, the student. Reply to this email with the meeting start time and room you select.

Email 8
Title: What time will work?
From: Jarla Knovak
Please find a time slot for a student, administrator, and professor to have an hour long meeting, in a conference room that can seat 3 people. People's schedules are presented on the first tab of the linked spreadsheet, and room availability is in the second.

People have two types of availability: completely free (shown in white) and possible commitments (shown in yellow), which can be moved if necessary. You should not schedule a meeting in unavailable time slots (shown in red). Scheduling decisions should minimize the number of possible commitments rescheduled, and prioritize the schedule of the professor, then the administrator, then, lastly, the student. Reply to this email with the meeting start time and room you select.

6. Heart Rate Variability (HRV)

Data Records of HRV - Supplementary Data Folder

Under the Supplementary Data folder on the OSF repository (Ref. [18]), there is a comma separated value (csv) file that holds the HRV data (15.9 MB). In this file, in addition to the columns holding the participant ID (***Column* A**) and group information (***Column* B**), there are columns holding treatment | task information (***Column* C** | ***Column* D**), absolute | relative timing (***Column* E** | ***Column* F**), and the RR values (***Column* G**). As it is the case with all other variables in the present paper, the OSF repository holds the quality controlled RR values. The raw variable values and the R code that operates upon them, to implement the quality control and validation processes described herein, reside on GitHub (Zaman, S. & Pavlidis, I. Office-Tasks-2019-Methods. *GitHub* `https://github.com/UH-CPL/Office-Tasks-2019-Methods`).

Quality Control of HRV

In addition to heart rate signals, BioHarness (Zephyr Technology, Annapolis, MD) reports beat to beat time intervals in ms, that is, RR values. These values are indicators of heart rate variability (HRV), a measure that can track sympathetic arousal, much like heart rate. RR values have their own set of noise problems, including ectopic beats, and thus a quality control check is in order.

The left column in Supplemental Fig. S10 shows the superimposed time series of RR values for all participants per treatment. Each RR value is depicted as a vertical line. From this visualization it is clear that there is noise in the form of very large RR values, particularly in the Presentation (PR) treatment, but also in Priming (PM) and in Dual Task (DT). The standard way to reduce such noise in RR data is to remove values that are on the tail end of the distribution (Ref. [30]). Specifically, we filter out RR values that are beyond two standard deviations from the mean, thus retaining $\sim 95\%$ of the values, while eliminating $\sim 5\%$ of extreme values fraught with noise. The cleansing effect of this action is shown in the right column of Supplemental Fig. S10. Please note that filtering is based on means and standard deviations computed per subject per treatment, to account for inter-individual variability.

We re-compute the means per subject per session for the RR data that passed quality control. These updated means, called NN (Ref. [30]), are considered reliable HRV indicators that can be used in analytics. Hence, the next step is to subject NN to experimental validity analysis, much like we did with the other five physiological variables.

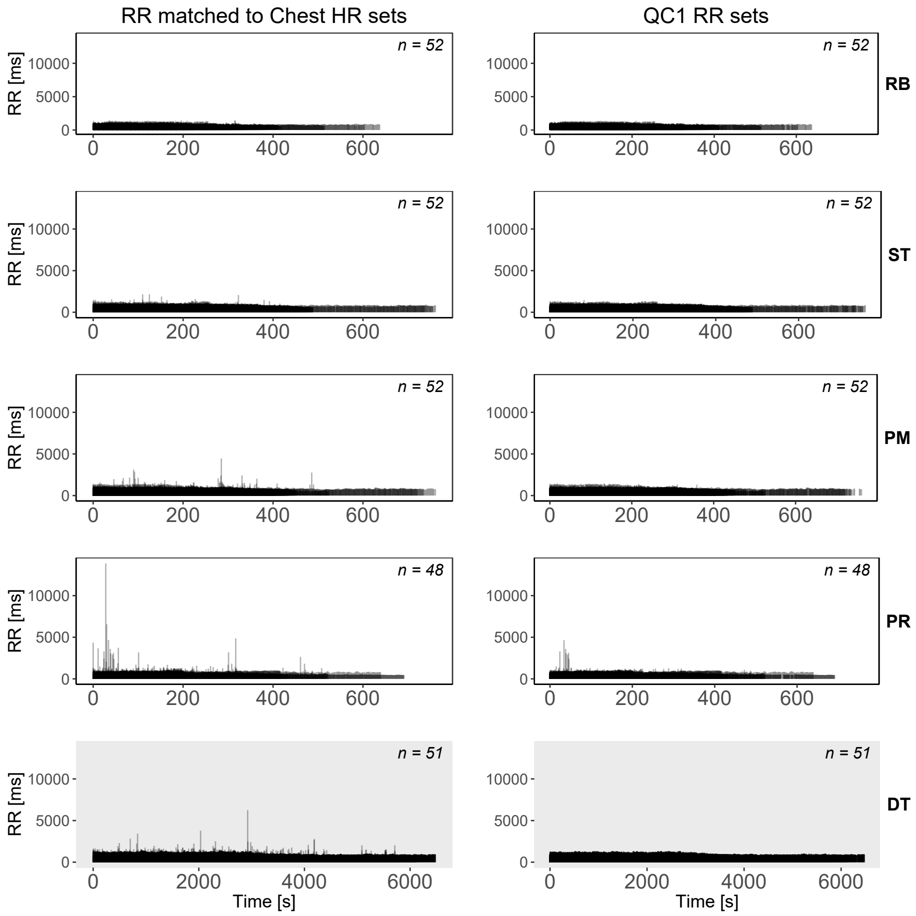

Figure S10: **Sets of RR data measured on the chest before and after quality control (QC1).** To facilitate meaningful cardiac channel comparisons, the original RR sets (left column) match the Chest HR sets that survived quality control (Supplemental Fig. S8). The outliers in the original RR data suggest ectopic beats, which took place mostly during PR and DT.

Experimental Validation of HRV

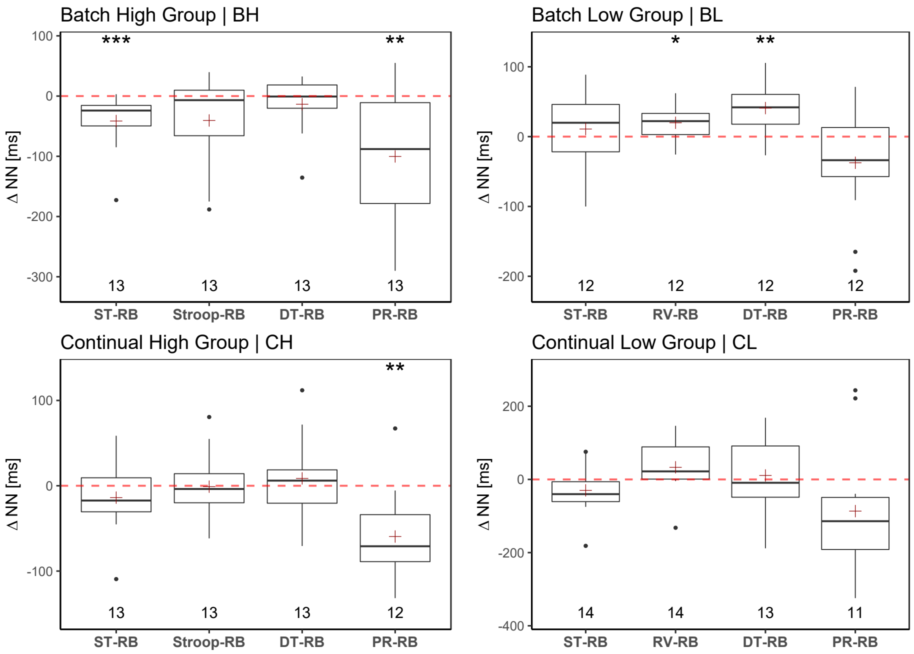

Figure S11: **Experimental validity of the chest NN variable.** Antithetical to heart rate boxplots in the main text, NN boxplots that are below the zero line manifest stressful treatments. Hence, NN largely captures the stressful effect of PR, but mostly misses it in ST and DT, a performance nearly on par with the Chest HR channel.

In contradistinction to heart rate, which increases with stress, HRV indicators, such as NN, tend to decrease with stress. Hence, validity for NN manifests when the normalized distribution means of stressful treatments are significantly lower than the zero line (Supplemental Fig. S11). Let $\mathrm{NN_C}(S_i, G_j, T_k)$ represent the chest NN value of participant S_i, in group G_j, for treatment T_k, where $i \in \{1, \cdots, 63\}$, $j \in \{\mathrm{BH}, \mathrm{BL}, \mathrm{CH}, \mathrm{CL}\}$, and $k \in \{\mathrm{ST}, \mathrm{Stroop}, \mathrm{RV}, \mathrm{DT}, \mathrm{PR}\}$, respectively. In each group G_j, we normalize within-participant the expected $\mathrm{NN_C}$ values by computing the distributions of paired differences between his/her $\mathrm{NN_C}$ in T_k and T_{RB}:

$$\Delta\mathrm{NN_C}(\cdot, G_j, T_k) = \mathrm{NN_C}(\cdot, G_j, T_k)\ [\mathrm{ms}] - \mathrm{NN_C}(\cdot, G_j, T_{\mathrm{RB}})\ [\mathrm{ms}]. \qquad \text{(S1)}$$

Equation (S1) produces the boxplots in Supplemental Fig. S11. The NULL hypothesis is that arousal within participants in treatments ST, Stroop, DT, and PR is no different than arousal in resting baseline RB.

The results indicate that $\mathrm{NN_C}$ mirrors to a large degree the performance of chest heart rate (Chest HR) presented in the main text. Indeed, $\mathrm{NN_C}$ cap-

tures well the stress effect of PR for the High stressor groups ($p < 0.01$, paired t-tests in BH, CH). For the Low stressor groups BL and CL, NN_C exhibits the correct trend but, unlike Chest HR (Fig. 8a1-d1), fails to reach significance. For all other treatments NN_C mirrors the disappointing performance of Chest HR. Specifically, NN_C captures the stressful effect of ST only in one group ($p < 0.001$, paired Wilcoxon signed-rank test in BH), while misses it in the other three groups ($p > 0.05$, paired t-tests in BL, CH, CL). It altogether misses the stressful effect of Stroop and DT. Based on these results, Chest HR is the preferred cardiac variable for this experiment, as NN slightly trails its performance. Overall, the NN_C results reaffirm the conclusion that cardiac variables tend to capture moderate office stressors, bearing an emotional component, such as presentations to an audience. At the same time, cardiac variables tend to perform poorly in mild office stressors of cognitive nature, such as standard report writing.

7. Quality Controlled 1 Chest HR, Wrist HR, and EDA Signals

This is an exhaustive list of the Chest HR, Wrist HR, and EDA signals that survived quality control 1 (QC 1) checks. Panels where the signals got eliminated in QC 1 feature a cross mark. Chest HR and Wrist HR signals are superimposed on the same graph to facilitate comparison. Display of signals progresses per participant per treatment, with heart rate signal panels appearing on the left and the corresponding EDA signal panels on the right. In heart rate signal panels, dashed lines indicate the respective means. Signals that are found by quality control 2 (QC 2) processes to be invalid, lend a lightened shade of their color to the panel background.

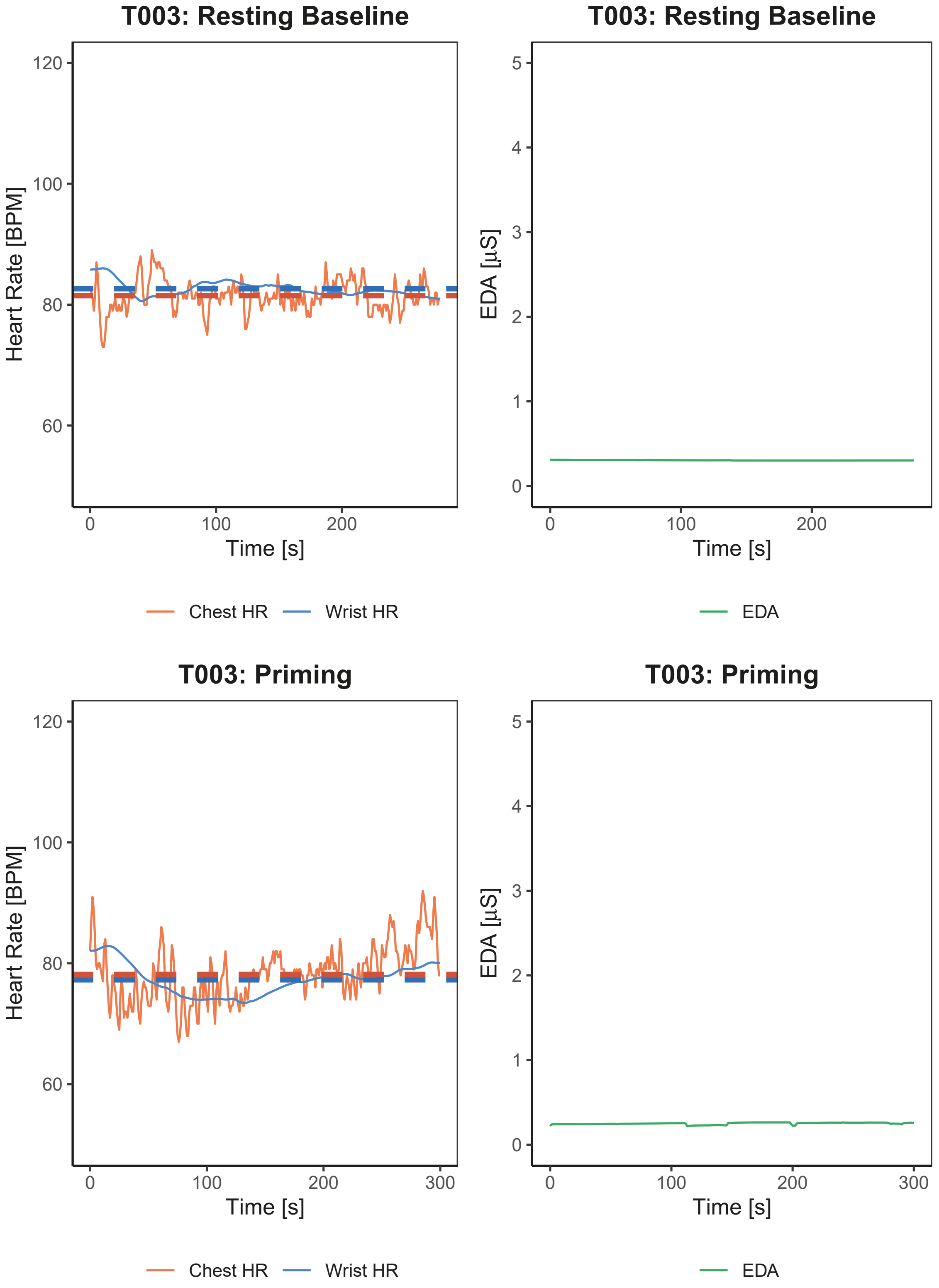
T003: Resting Baseline
Heart Rate [BPM]
Time [s]
Chest HR
Wrist HR
T003: Resting Baseline
EDA [µS]
Time [s]
EDA
T003: Priming
Heart Rate [BPM]
Time [s]
Chest HR
Wrist HR
T003: Priming
EDA [µS]
Time [s]
EDA

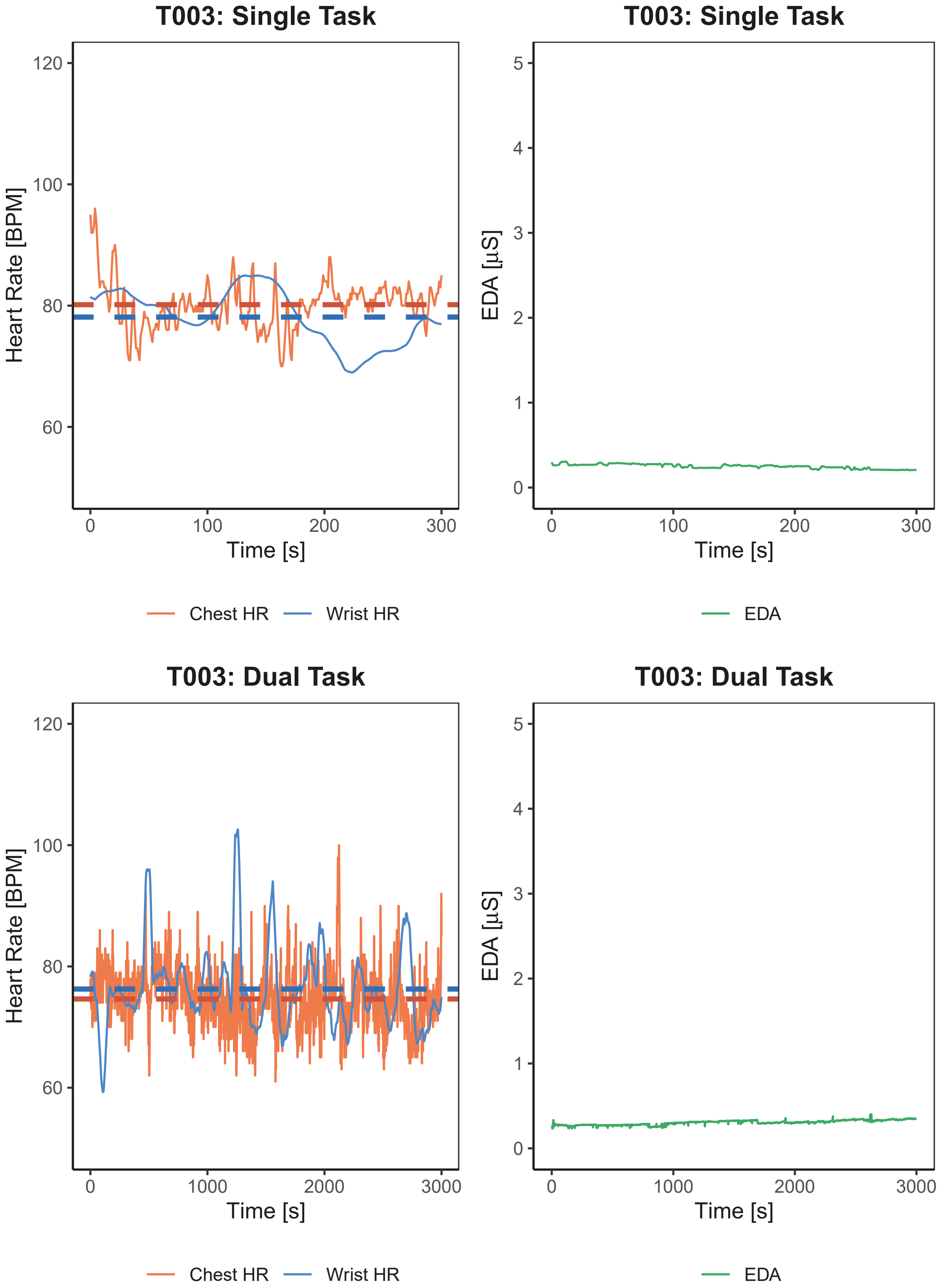
T003: Single Task
Heart Rate [BPM]
120
100
80
60
0
100
200
300
Time [s]
Chest HR
Wrist HR
T003: Single Task
EDA [µS]
5
4
3
2
1
0
0
100
200
300
Time [s]
EDA
T003: Dual Task
Heart Rate [BPM]
120
100
80
60
0
1000
2000
3000
Time [s]
Chest HR
Wrist HR
T003: Dual Task
EDA [µS]
5
4
3
2
1
0
0
1000
2000
3000
Time [s]
EDA

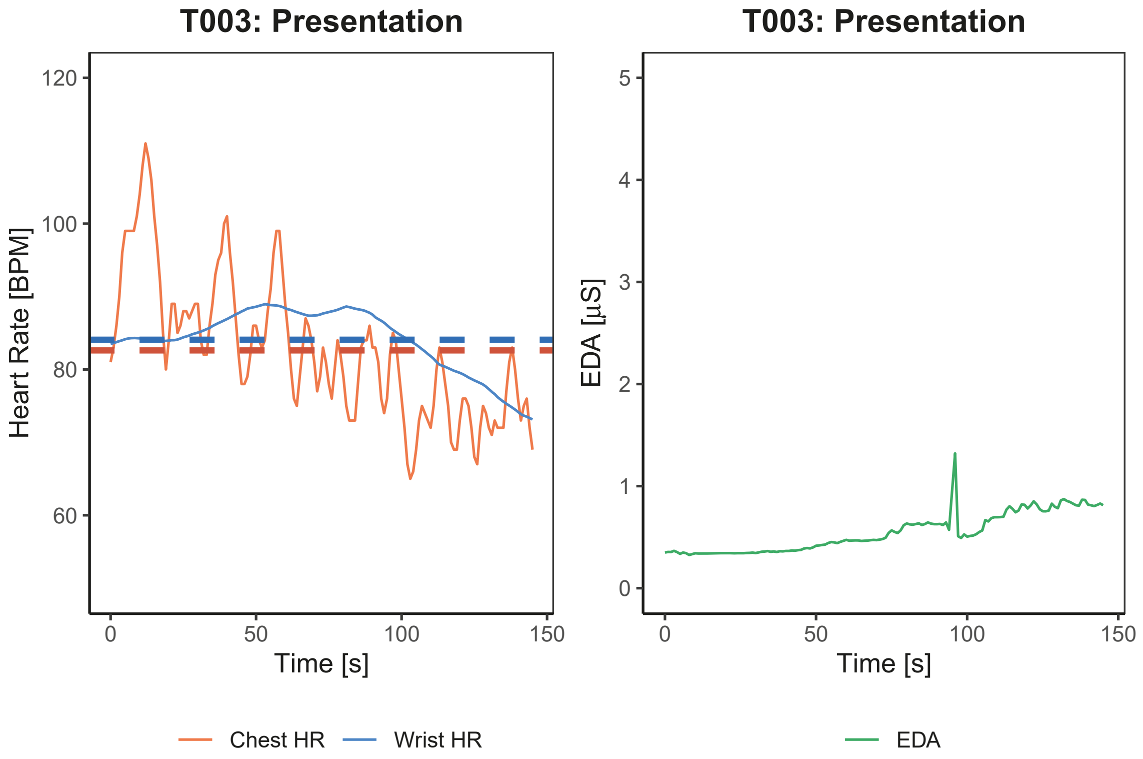

T003: Presentation
Heart Rate [BPM]
Time [s]
Chest HR
Wrist HR
T003: Presentation
EDA [µS]
Time [s]
EDA

--

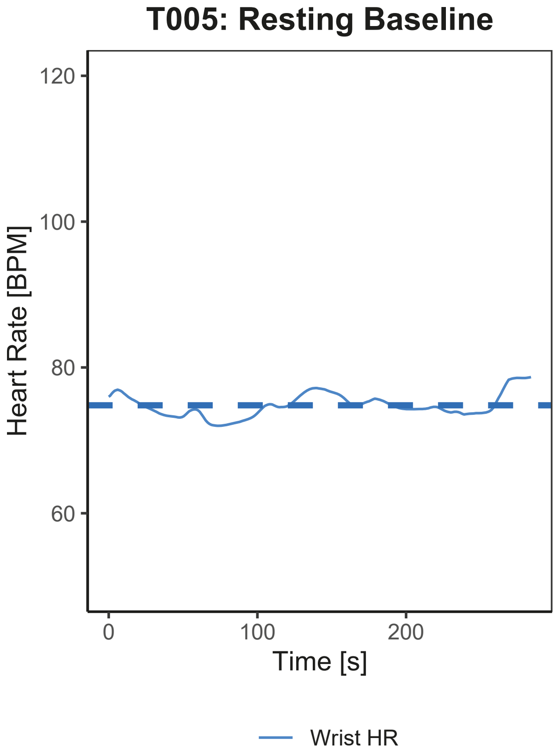
T005: Resting Baseline
Heart Rate [BPM]
120
100
80
60
0
100
200
Time [s]
Wrist HR

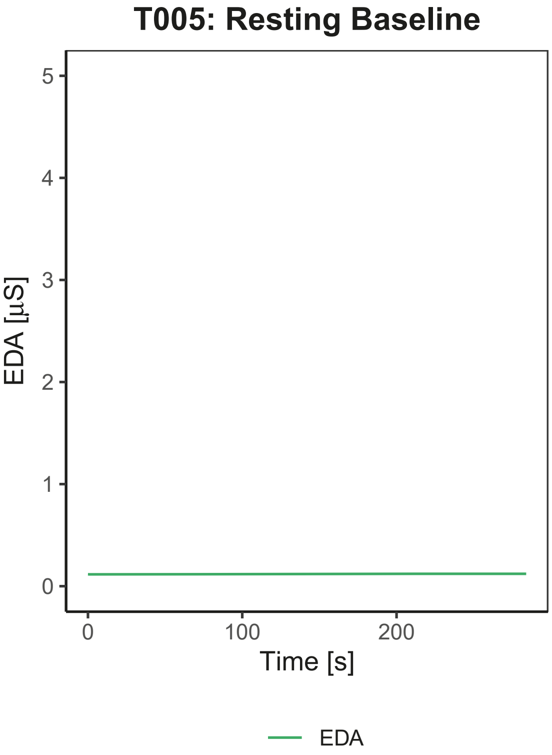
T005: Resting Baseline
EDA [µS]
5
4
3
2
1
0
0
100
200
Time [s]
EDA

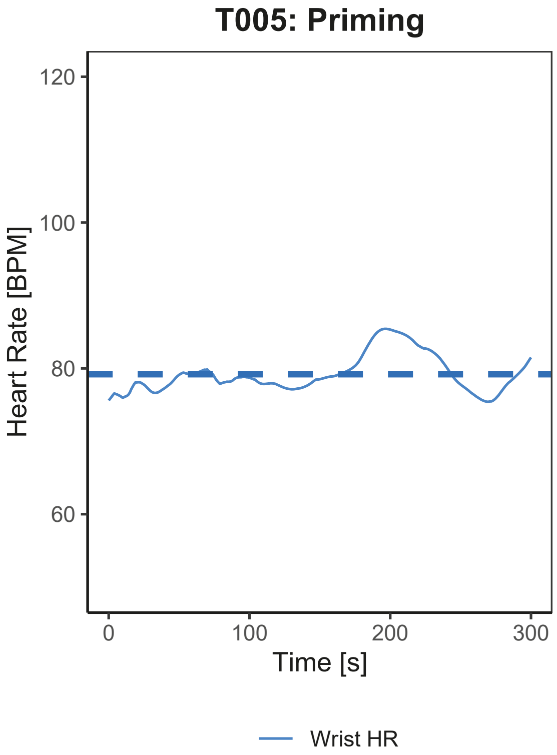
T005: Priming
Heart Rate [BPM]
120
100
80
60
0
100
200
300
Time [s]
Wrist HR

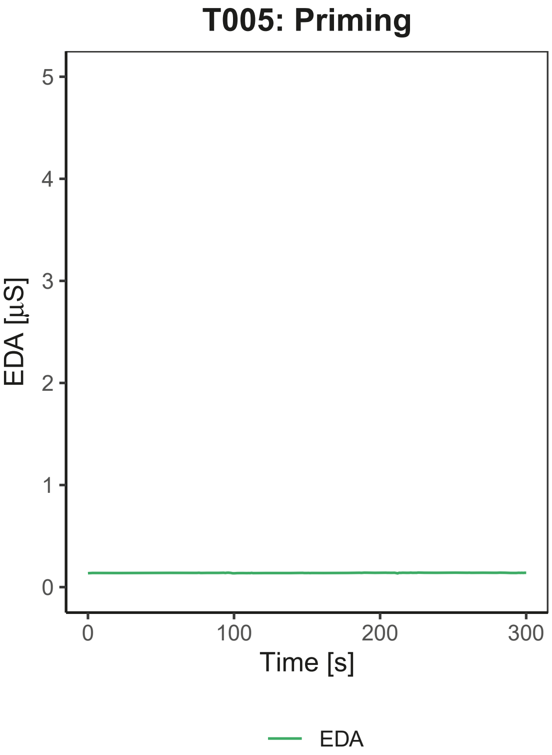
T005: Priming
EDA [µS]
5
4
3
2
1
0
0
100
200
300
Time [s]
EDA

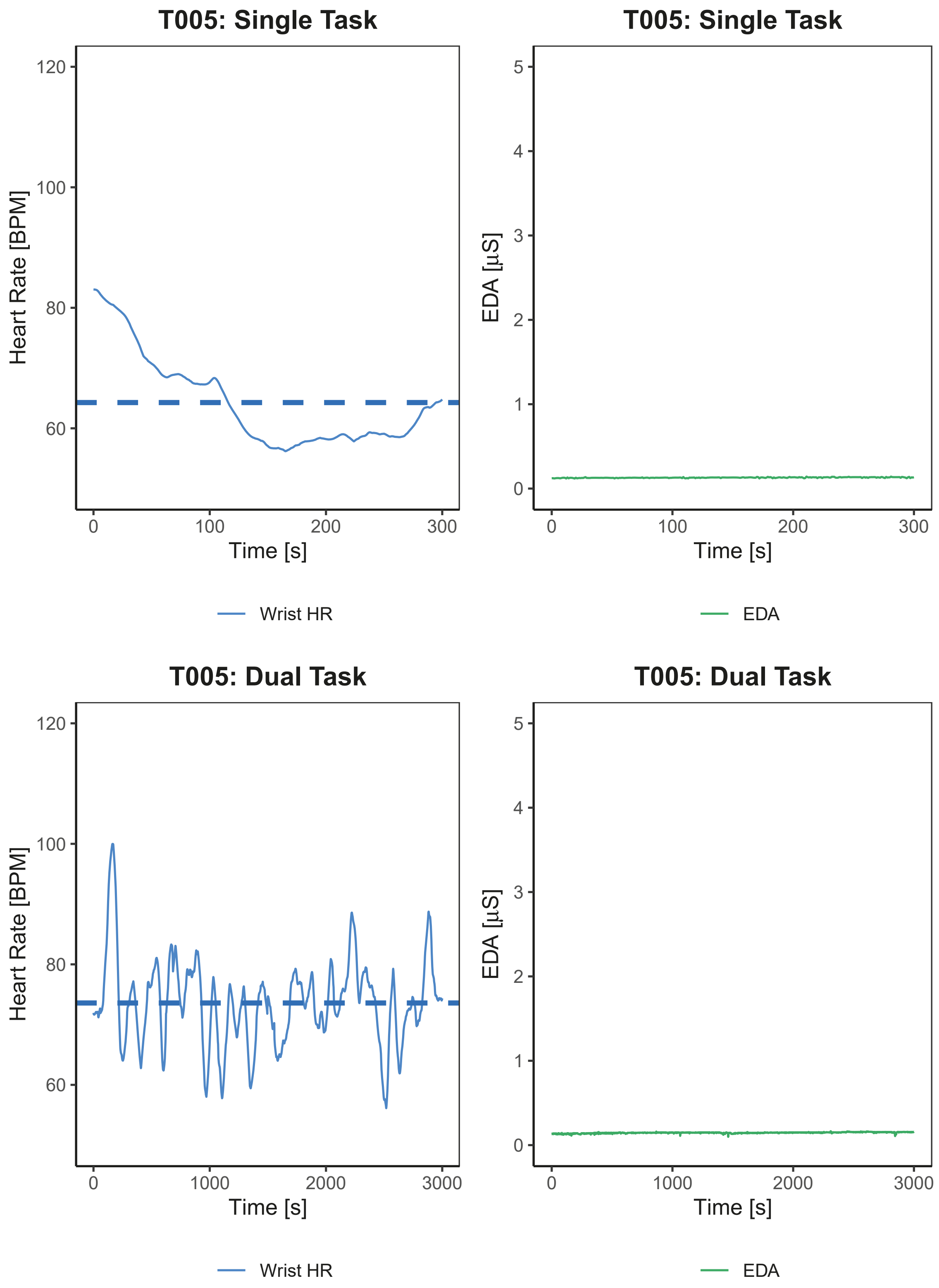

T005: Single Task
Heart Rate [BPM]
Time [s]
Wrist HR
T005: Single Task
EDA [µS]
Time [s]
EDA
T005: Dual Task
Heart Rate [BPM]
Time [s]
Wrist HR
T005: Dual Task
EDA [µS]
Time [s]
EDA

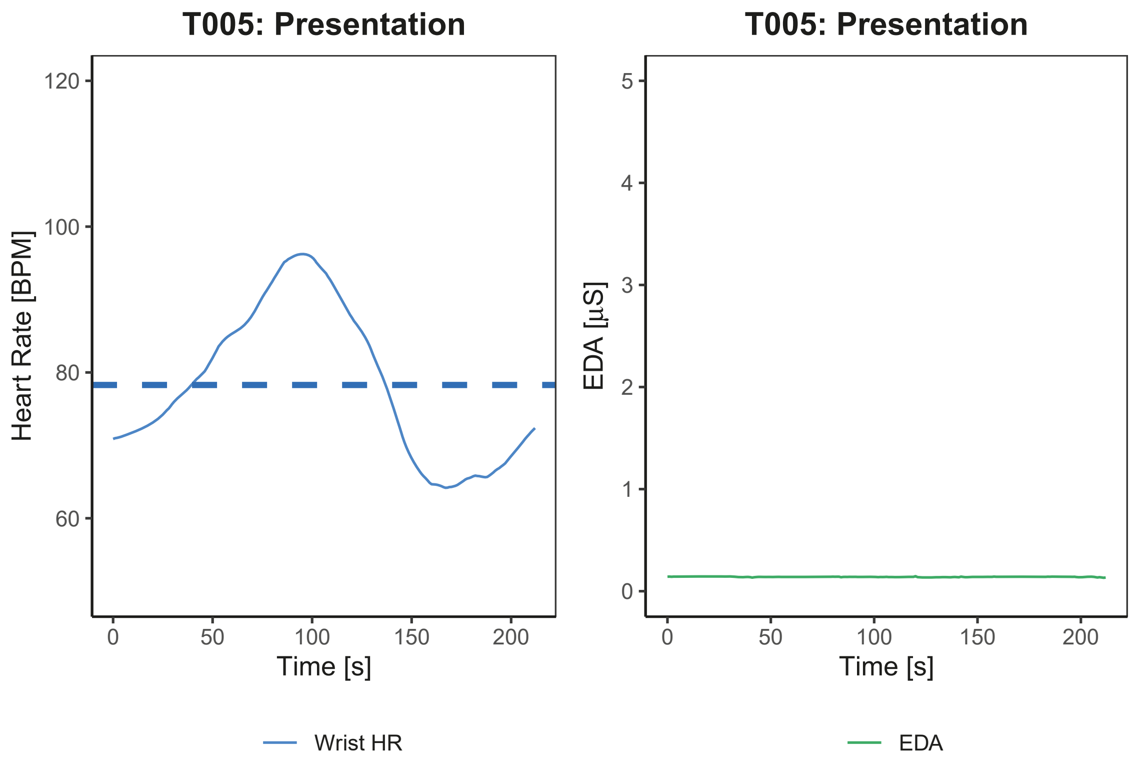
T005: Presentation
Heart Rate [BPM]
120
100
80
60
0
50
100
150
200
Time [s]
Wrist HR
T005: Presentation
EDA [µS]
5
4
3
2
1
0
0
50
100
150
200
Time [s]
EDA

```
## ------------------------------------------------------------------------------------- ##
```

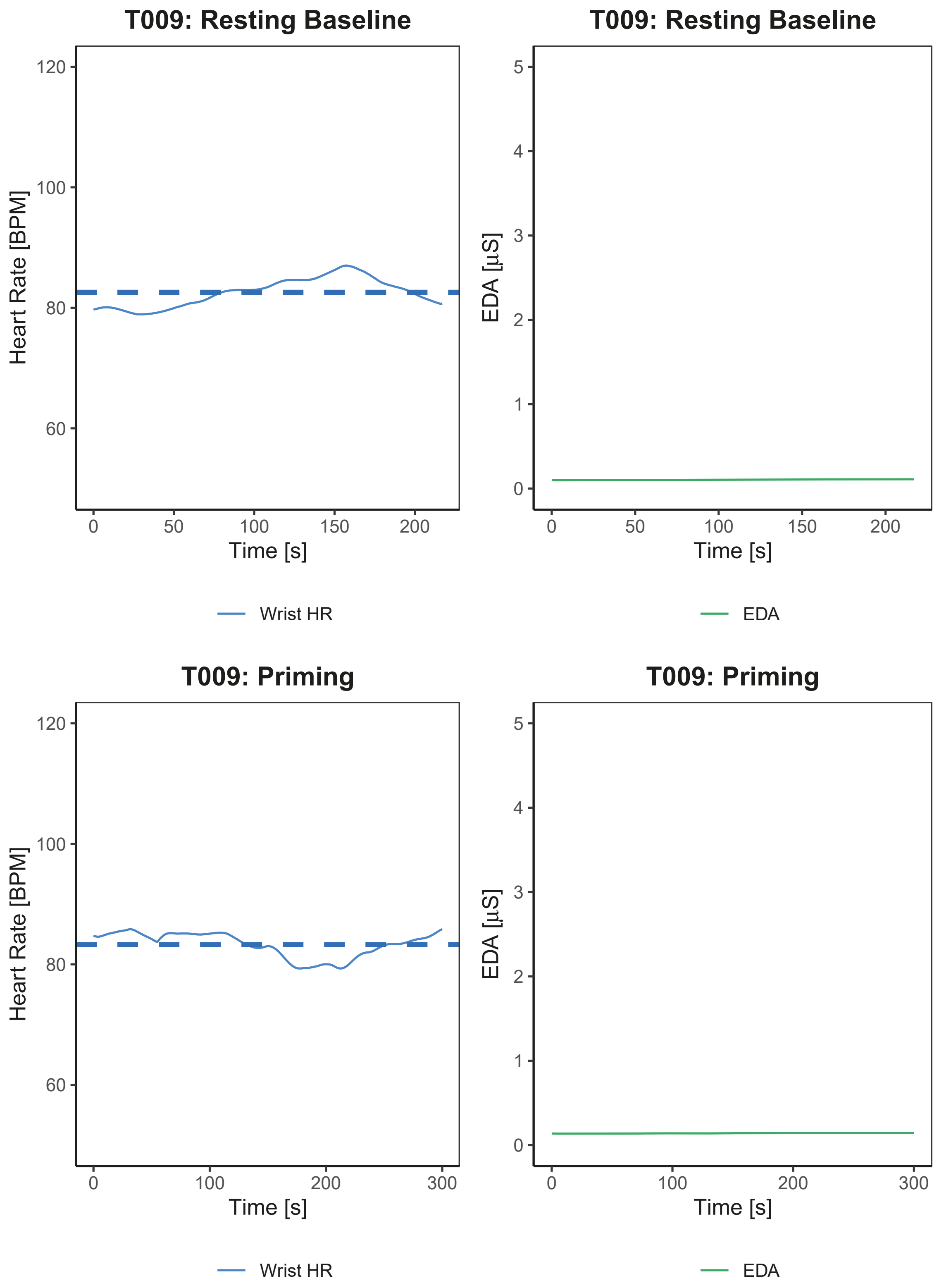
T009: Resting Baseline
Heart Rate [BPM]
120
100
80
60
0
50
100
150
200
Time [s]
Wrist HR
T009: Resting Baseline
EDA [µS]
5
4
3
2
1
0
0
50
100
150
200
Time [s]
EDA
T009: Priming
Heart Rate [BPM]
120
100
80
60
0
100
200
300
Time [s]
Wrist HR
T009: Priming
EDA [µS]
5
4
3
2
1
0
0
100
200
300
Time [s]
EDA

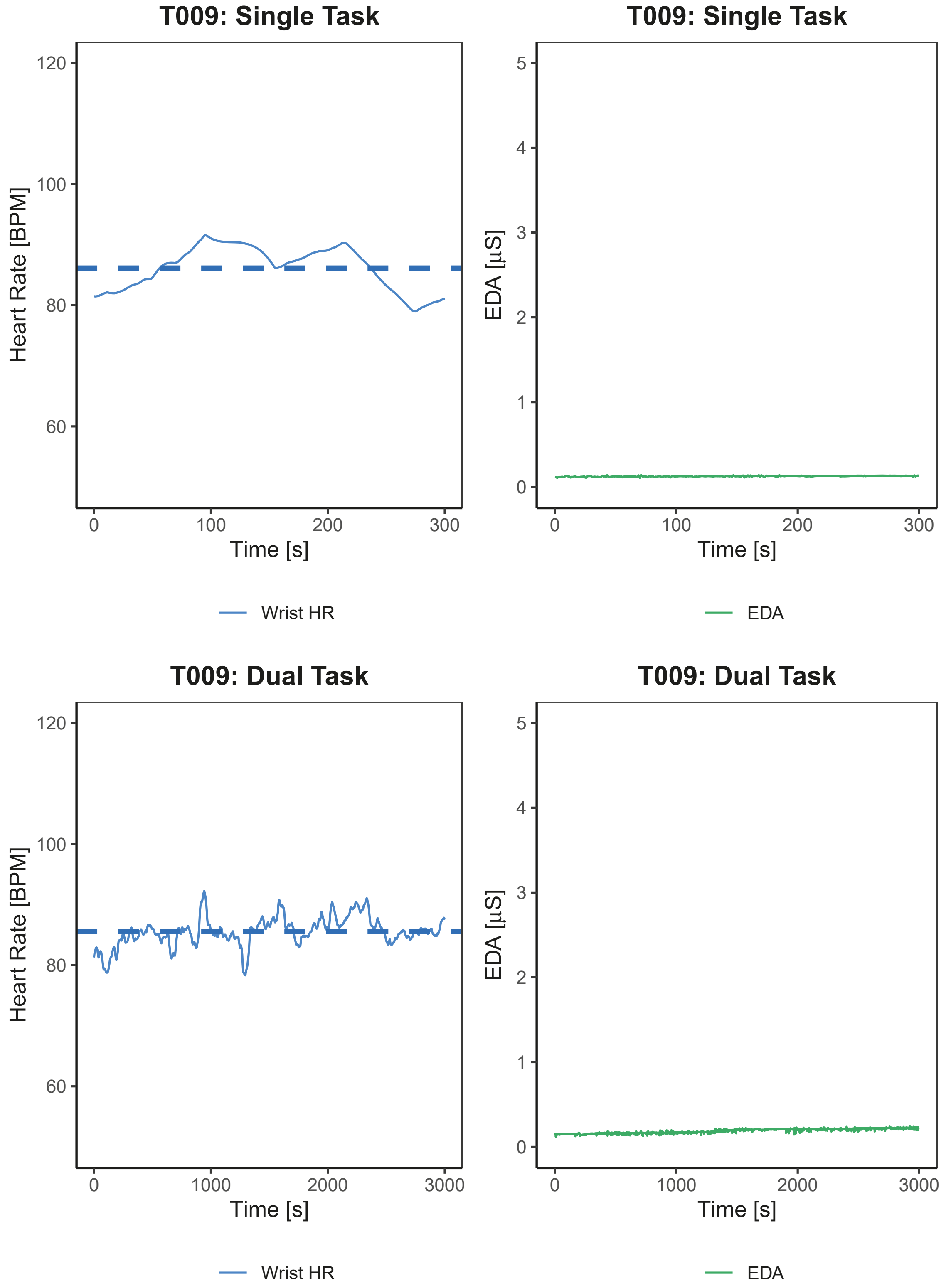
T009: Single Task
Heart Rate [BPM]
Time [s]
Wrist HR
T009: Single Task
EDA [µS]
Time [s]
EDA
T009: Dual Task
Heart Rate [BPM]
Time [s]
Wrist HR
T009: Dual Task
EDA [µS]
Time [s]
EDA

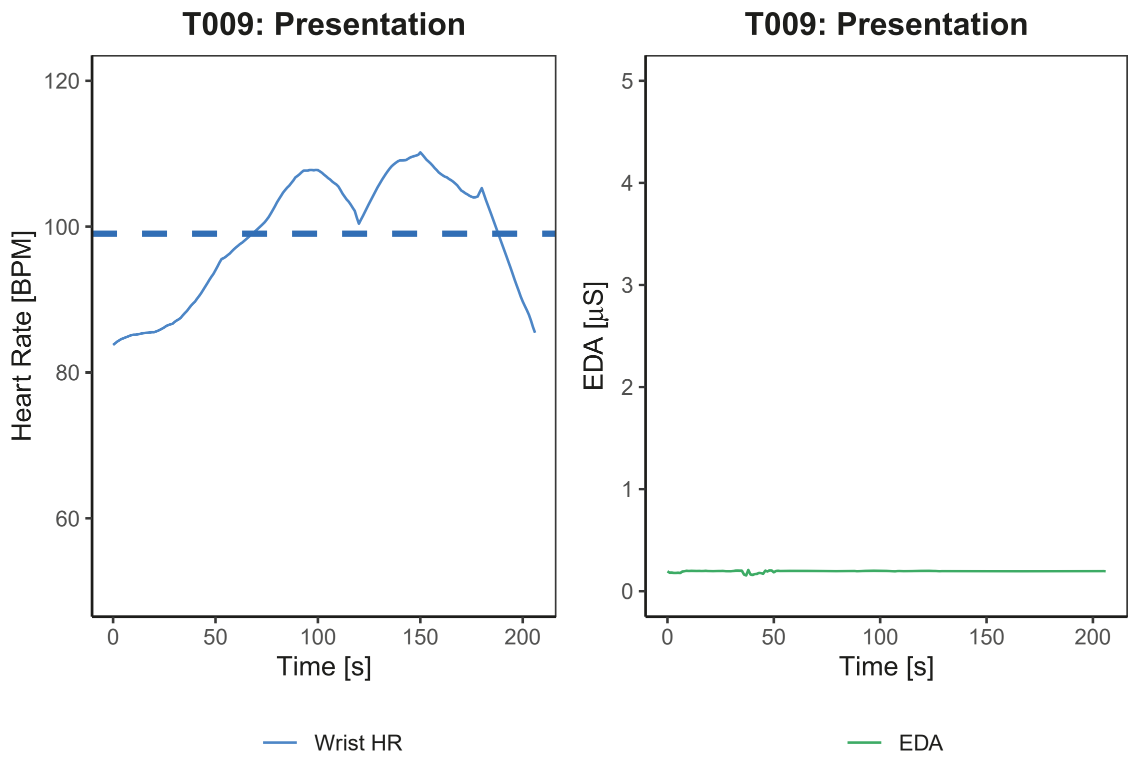
T009: Presentation
Heart Rate [BPM]
120
100
80
60
0
50
100
150
200
Time [s]
Wrist HR
T009: Presentation
EDA [µS]
5
4
3
2
1
0
0
50
100
150
200
Time [s]
EDA

--

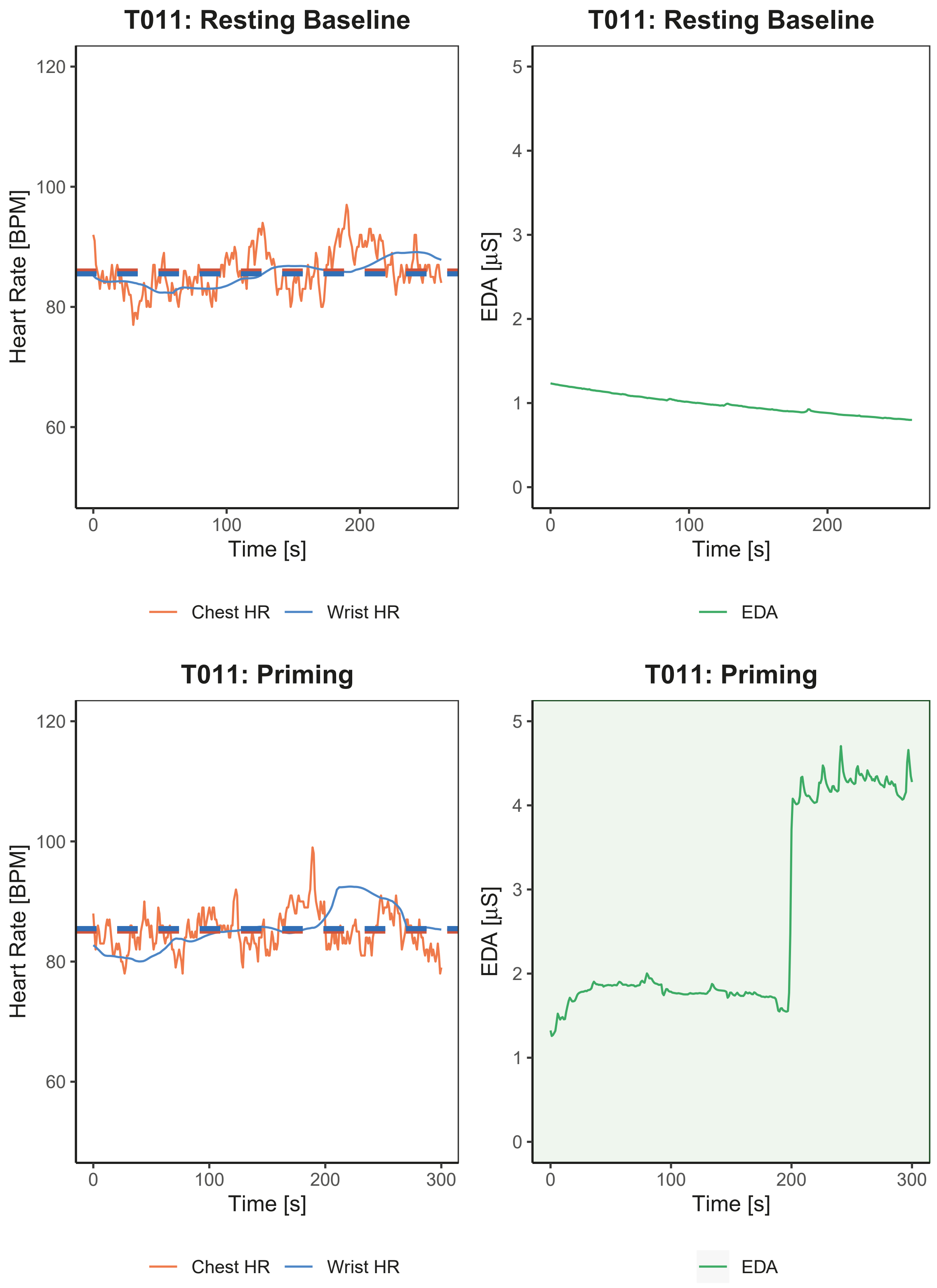
T011: Resting Baseline
Heart Rate [BPM]
120
100
80
60
0
100
200
Time [s]
Chest HR
Wrist HR
T011: Resting Baseline
EDA [µS]
5
4
3
2
1
0
0
100
200
Time [s]
EDA
T011: Priming
Heart Rate [BPM]
120
100
80
60
0
100
200
300
Time [s]
Chest HR
Wrist HR
T011: Priming
EDA [µS]
5
4
3
2
1
0
0
100
200
300
Time [s]
EDA

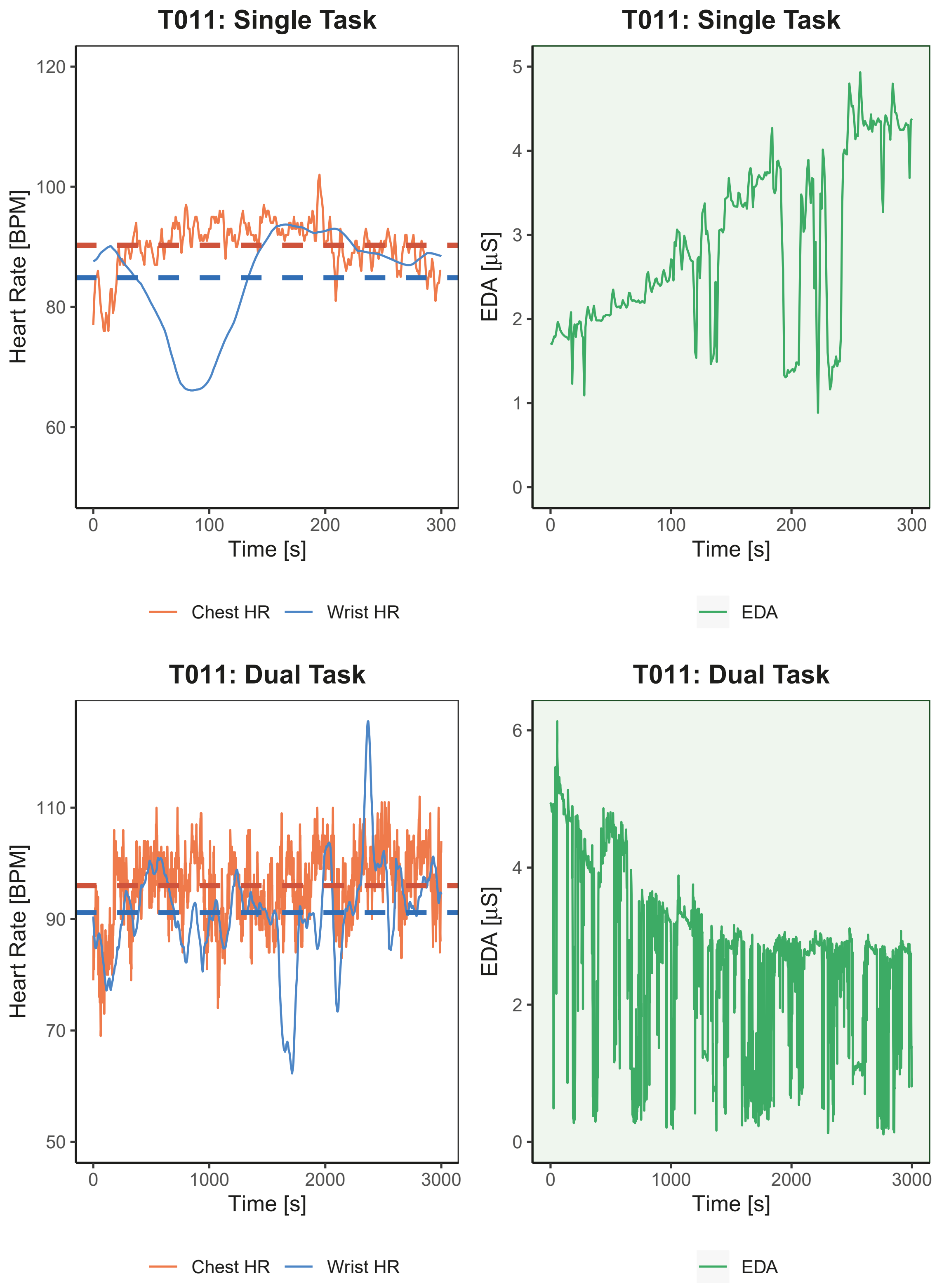
T011: Single Task
Heart Rate [BPM]
120
100
80
60
0
100
200
300
Time [s]
Chest HR
Wrist HR
T011: Single Task
EDA [µS]
5
4
3
2
1
0
0
100
200
300
Time [s]
EDA
T011: Dual Task
Heart Rate [BPM]
110
90
70
50
0
1000
2000
3000
Time [s]
Chest HR
Wrist HR
T011: Dual Task
EDA [µS]
6
4
2
0
0
1000
2000
3000
Time [s]
EDA

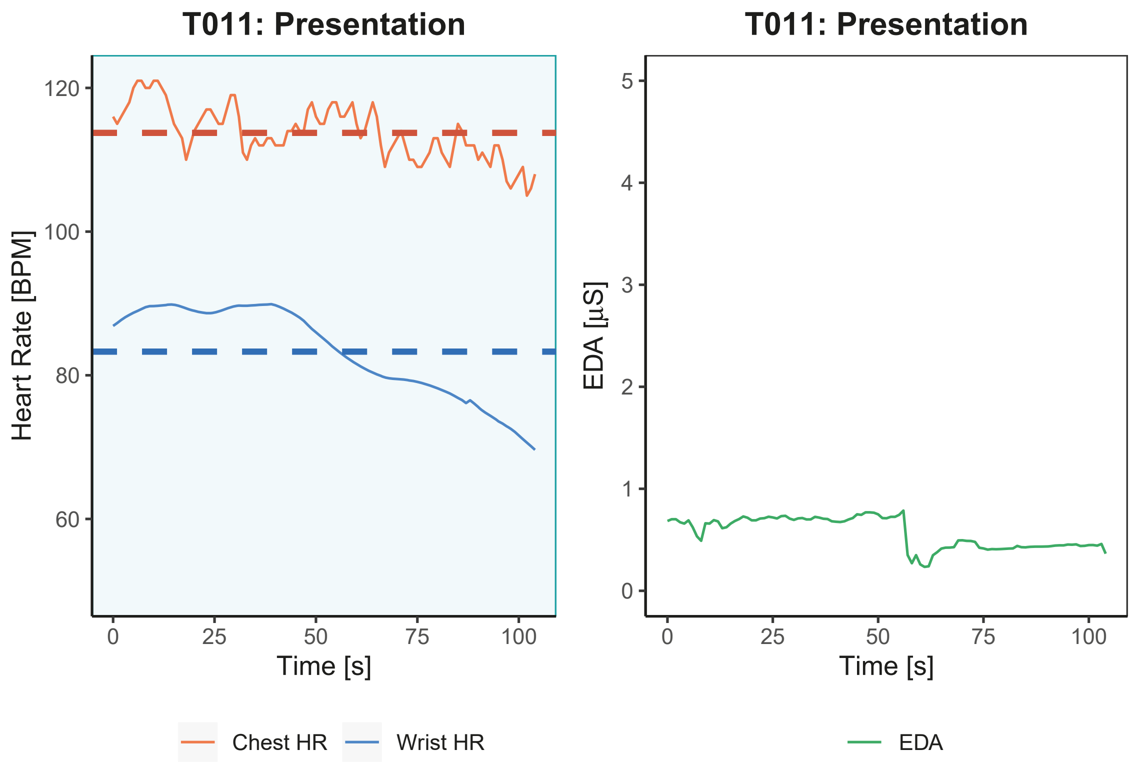

T011: Presentation
Heart Rate [BPM]
120
100
80
60
0
25
50
75
100
Time [s]
Chest HR
Wrist HR
T011: Presentation
EDA [µS]
5
4
3
2
1
0
0
25
50
75
100
Time [s]
EDA

--

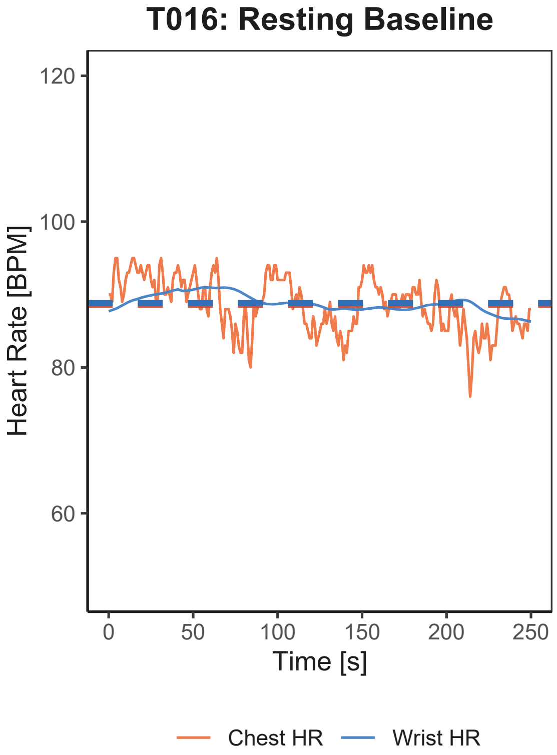

T016: Resting Baseline
Heart Rate [BPM]
120
100
80
60
0
50
100
150
200
250
Time [s]
Chest HR
Wrist HR

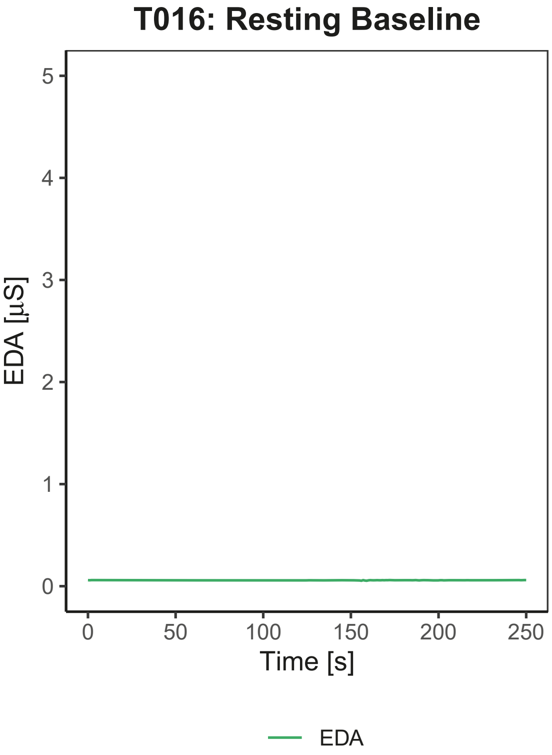

T016: Resting Baseline
EDA [µS]
5
4
3
2
1
0
0
50
100
150
200
250
Time [s]
EDA

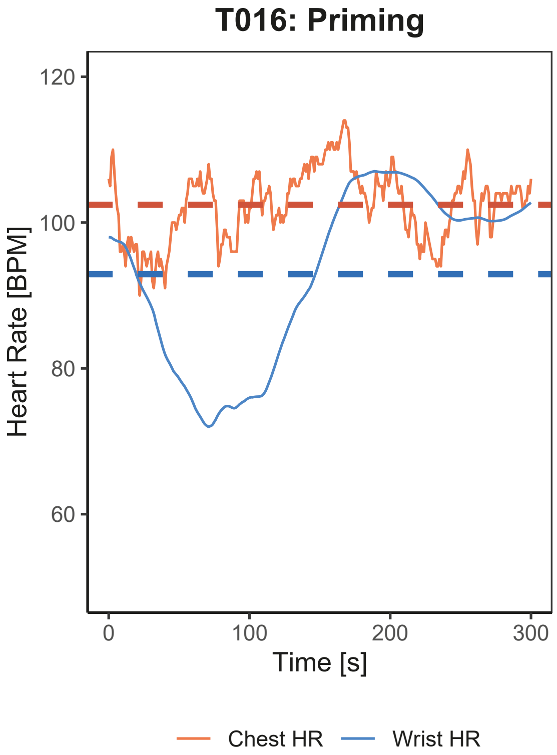

T016: Priming
Heart Rate [BPM]
120
100
80
60
0
100
200
300
Time [s]
Chest HR
Wrist HR

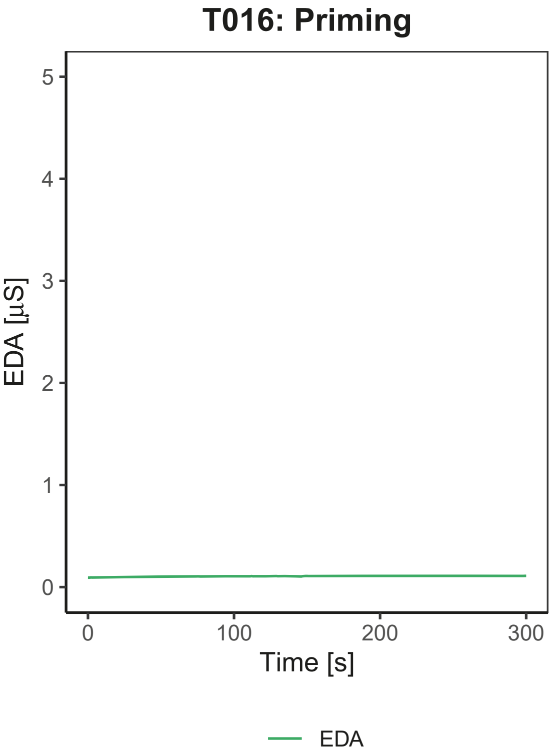

T016: Priming
EDA [µS]
5
4
3
2
1
0
0
100
200
300
Time [s]
EDA

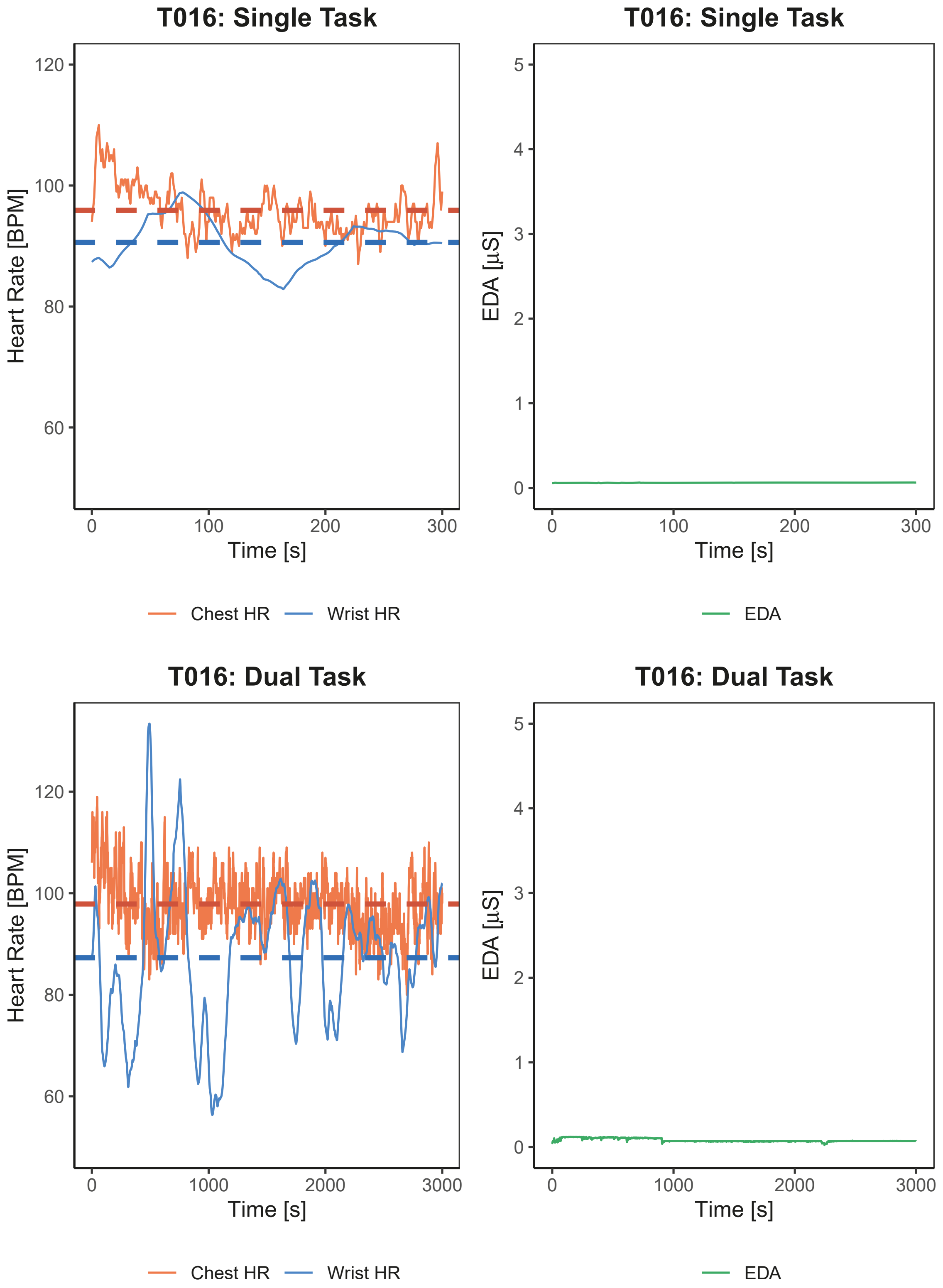
T016: Single Task
Heart Rate [BPM]
Time [s]
Chest HR
Wrist HR
T016: Single Task
EDA [µS]
Time [s]
EDA
T016: Dual Task
Heart Rate [BPM]
Time [s]
Chest HR
Wrist HR
T016: Dual Task
EDA [µS]
Time [s]
EDA

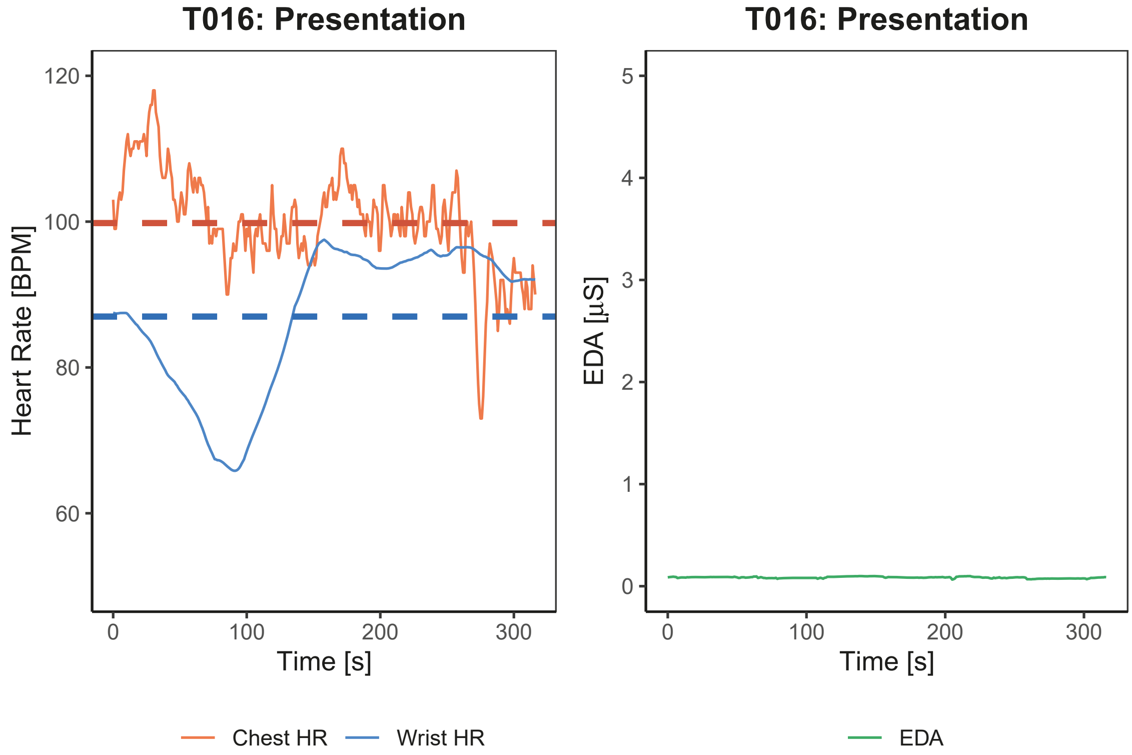
T016: Presentation
Heart Rate [BPM]
120
100
80
60
0
100
200
300
Time [s]
Chest HR
Wrist HR
T016: Presentation
EDA [µS]
5
4
3
2
1
0
0
100
200
300
Time [s]
EDA

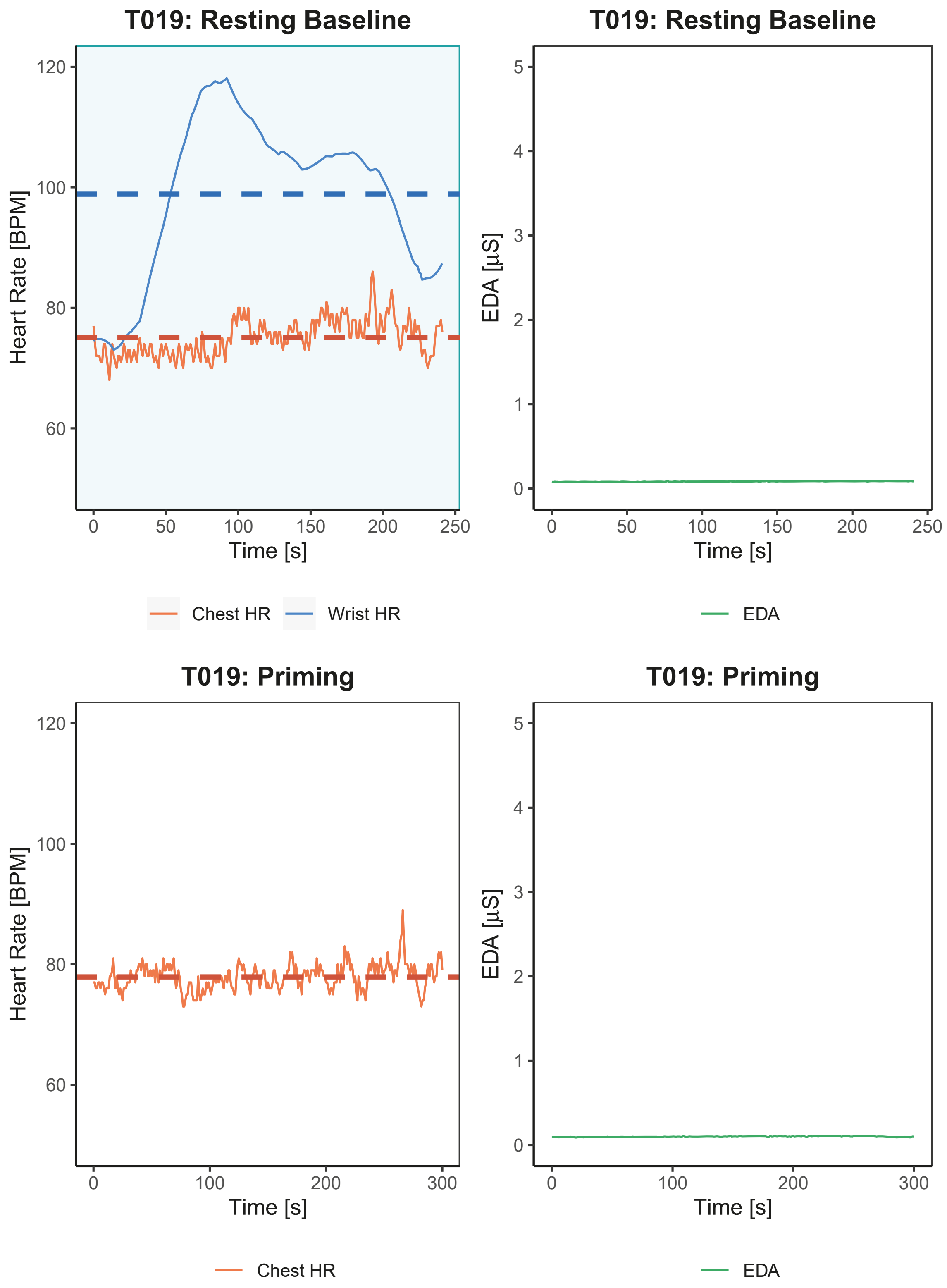
T019: Resting Baseline
Heart Rate [BPM]
120
100
80
60
0
50
100
150
200
250
Time [s]
Chest HR
Wrist HR
T019: Resting Baseline
EDA [µS]
5
4
3
2
1
0
0
50
100
150
200
250
Time [s]
EDA
T019: Priming
Heart Rate [BPM]
120
100
80
60
0
100
200
300
Time [s]
Chest HR
T019: Priming
EDA [µS]
5
4
3
2
1
0
0
100
200
300
Time [s]
EDA

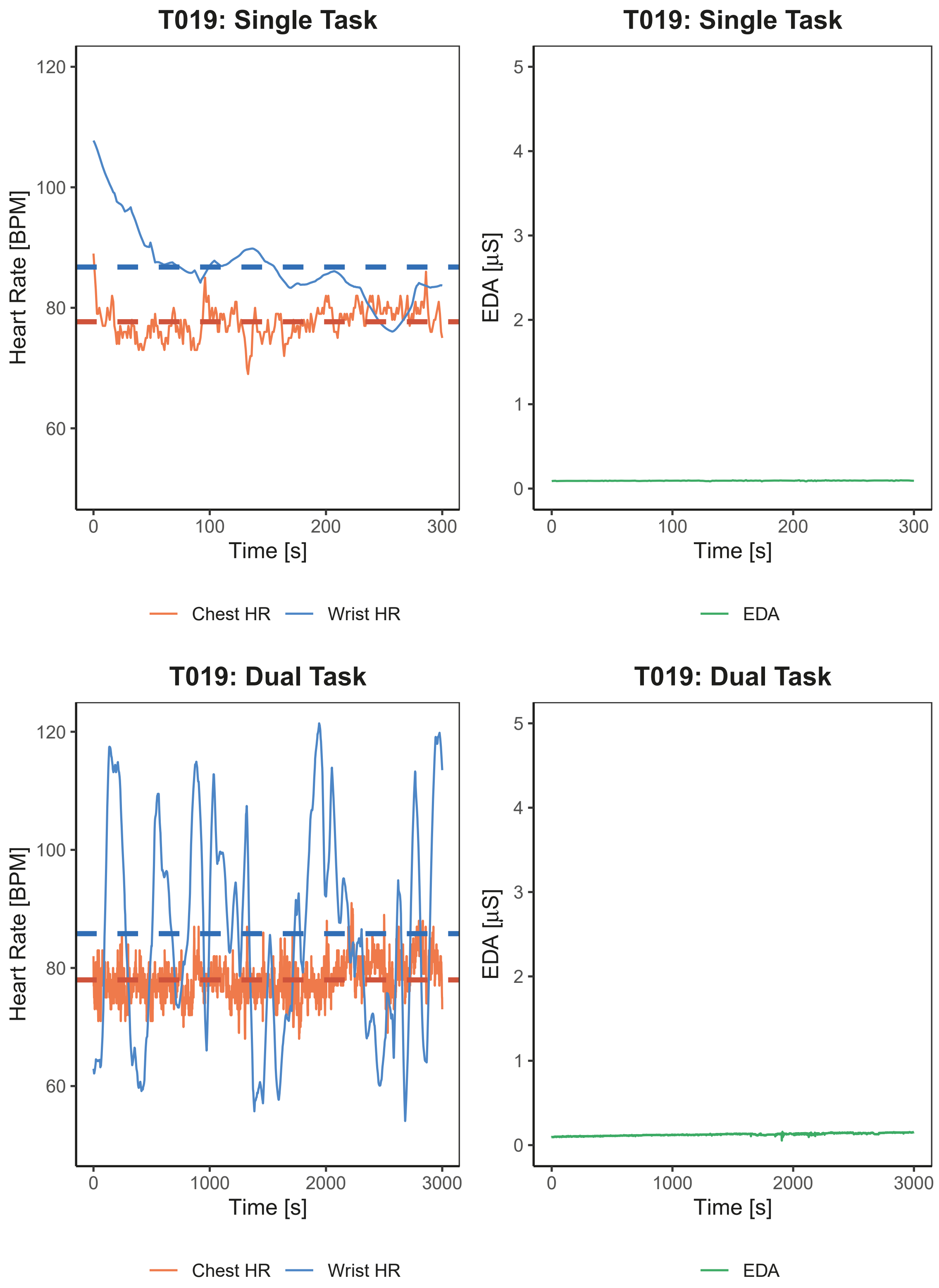
T019: Single Task
Heart Rate [BPM]
Time [s]
Chest HR
Wrist HR
T019: Single Task
EDA [µS]
Time [s]
EDA
T019: Dual Task
Heart Rate [BPM]
Time [s]
Chest HR
Wrist HR
T019: Dual Task
EDA [µS]
Time [s]
EDA

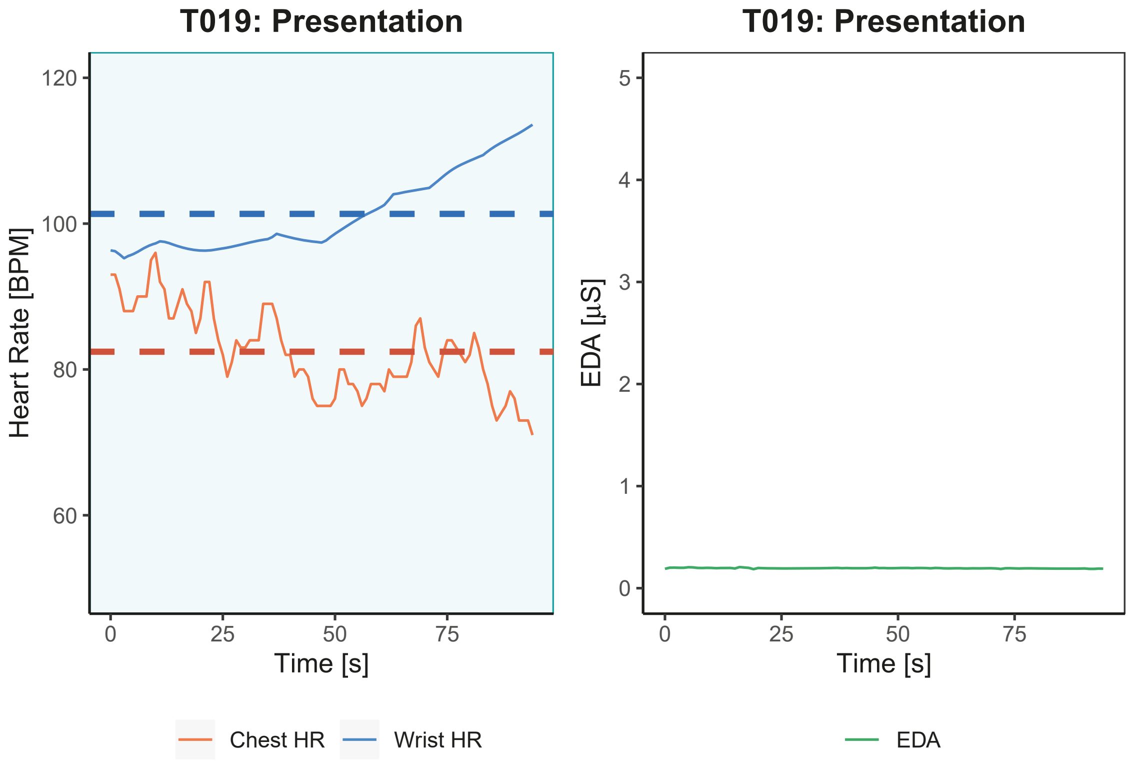

T019: Presentation
Heart Rate [BPM]
120
100
80
60
0
25
50
75
Time [s]
Chest HR
Wrist HR
T019: Presentation
EDA [µS]
5
4
3
2
1
0
0
25
50
75
Time [s]
EDA

--

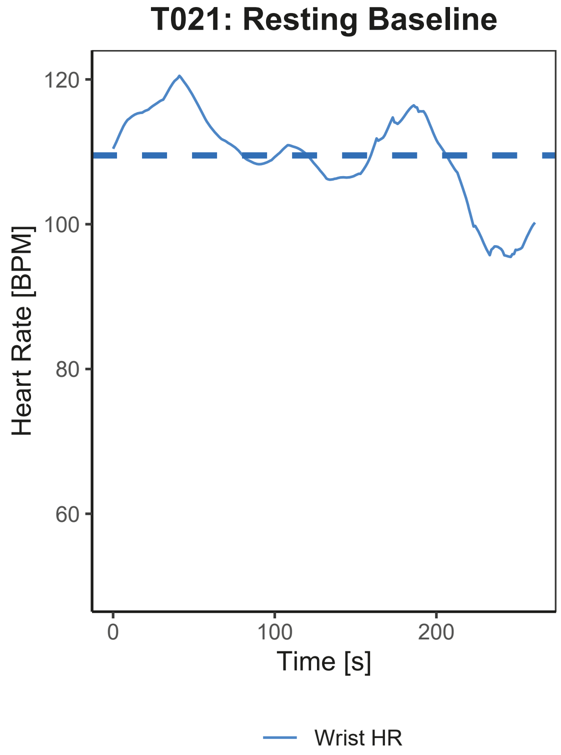
T021: Resting Baseline
Heart Rate [BPM]
120
100
80
60
0
100
200
Time [s]
Wrist HR

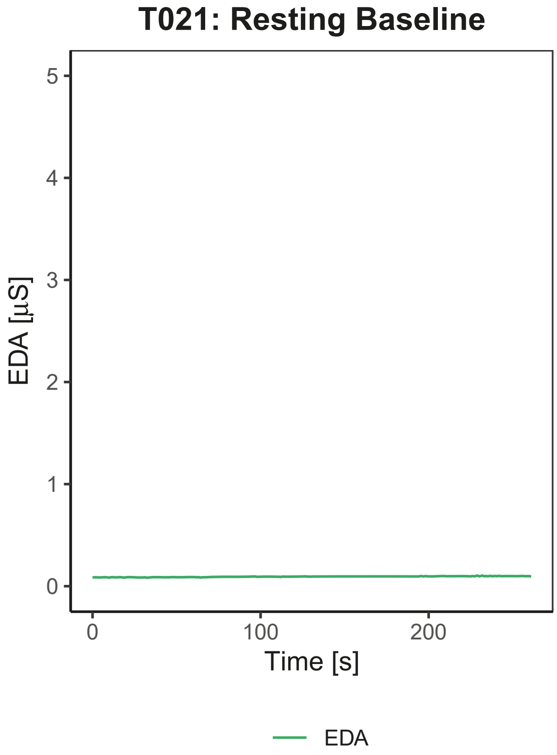
T021: Resting Baseline
EDA [µS]
5
4
3
2
1
0
0
100
200
Time [s]
EDA

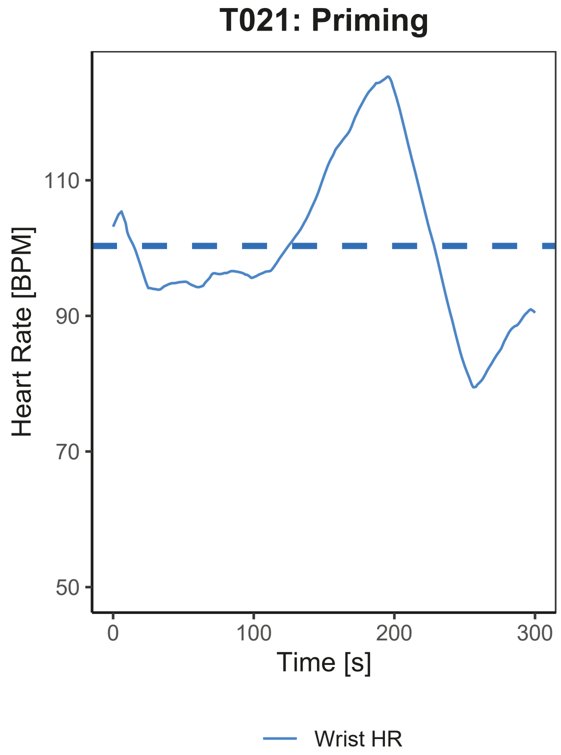
T021: Priming
Heart Rate [BPM]
110
90
70
50
0
100
200
300
Time [s]
Wrist HR

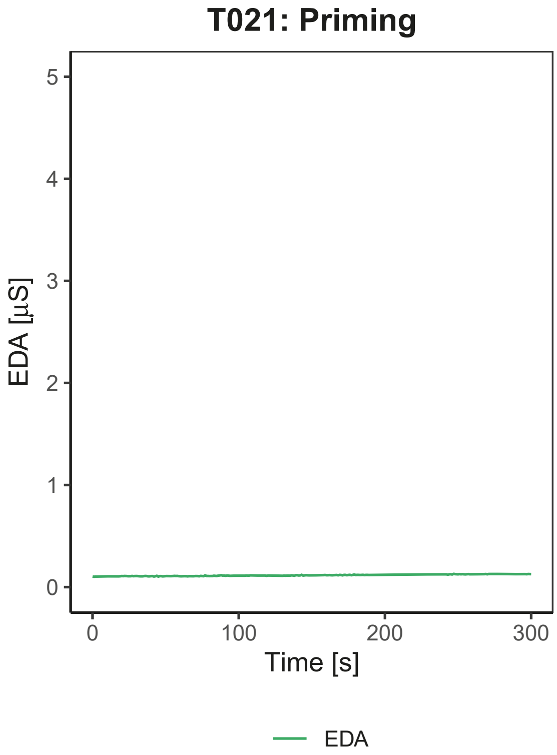
T021: Priming
EDA [µS]
5
4
3
2
1
0
0
100
200
300
Time [s]
EDA

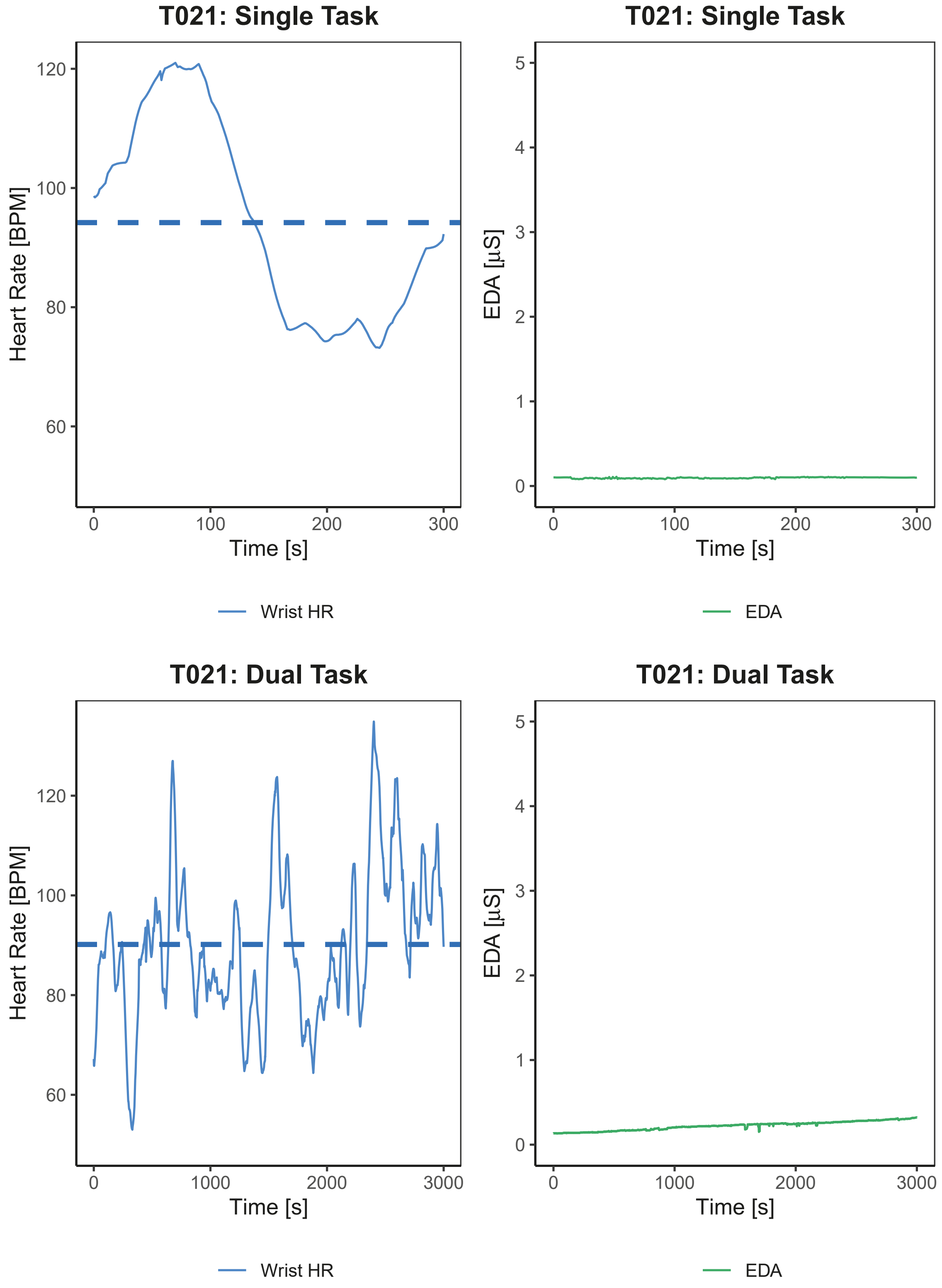
T021: Single Task
Heart Rate [BPM]
Time [s]
Wrist HR
T021: Single Task
EDA [µS]
Time [s]
EDA
T021: Dual Task
Heart Rate [BPM]
Time [s]
Wrist HR
T021: Dual Task
EDA [µS]
Time [s]
EDA

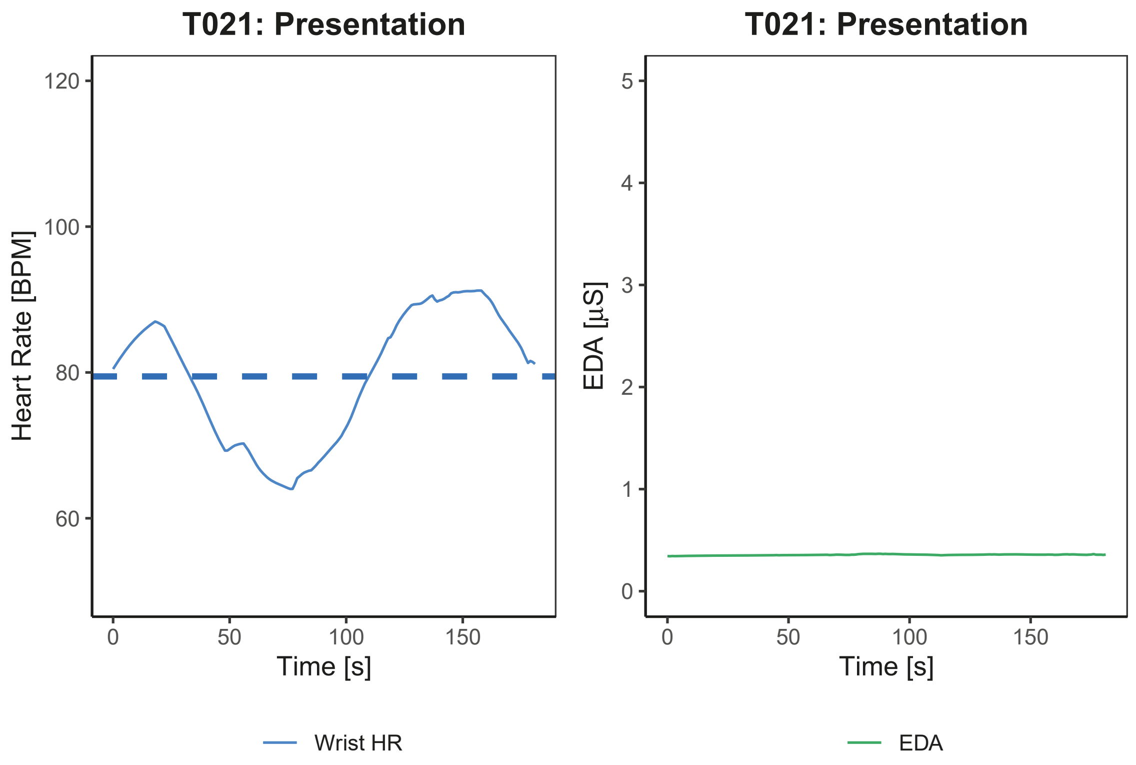
T021: Presentation
Heart Rate [BPM]
120
100
80
60
0
50
100
150
Time [s]
Wrist HR
T021: Presentation
EDA [µS]
5
4
3
2
1
0
0
50
100
150
Time [s]
EDA

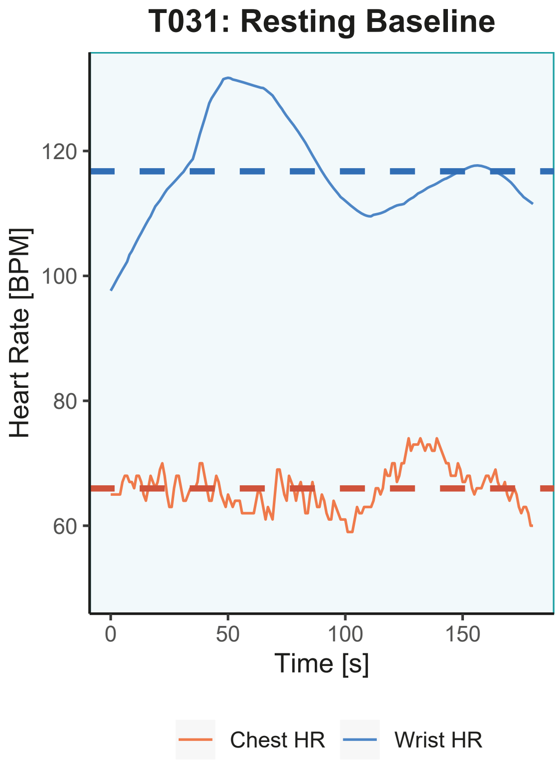
T031: Resting Baseline
Heart Rate [BPM]
Time [s]
Chest HR
Wrist HR

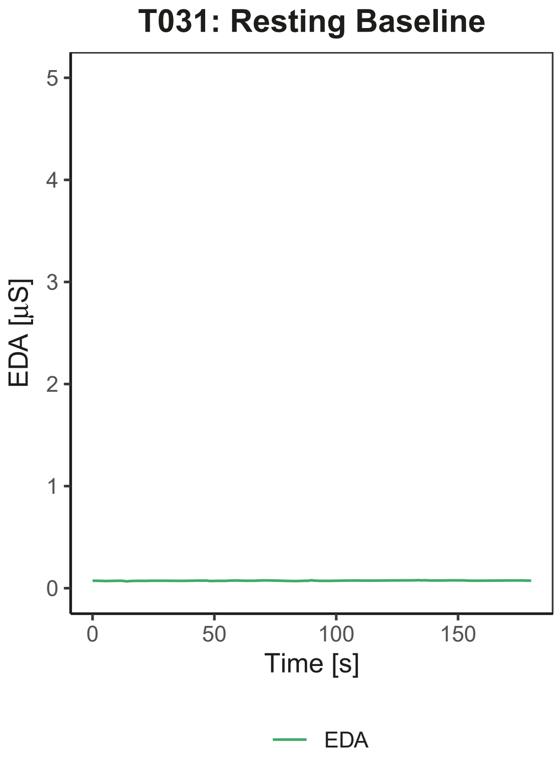
T031: Resting Baseline
EDA [µS]
Time [s]
EDA

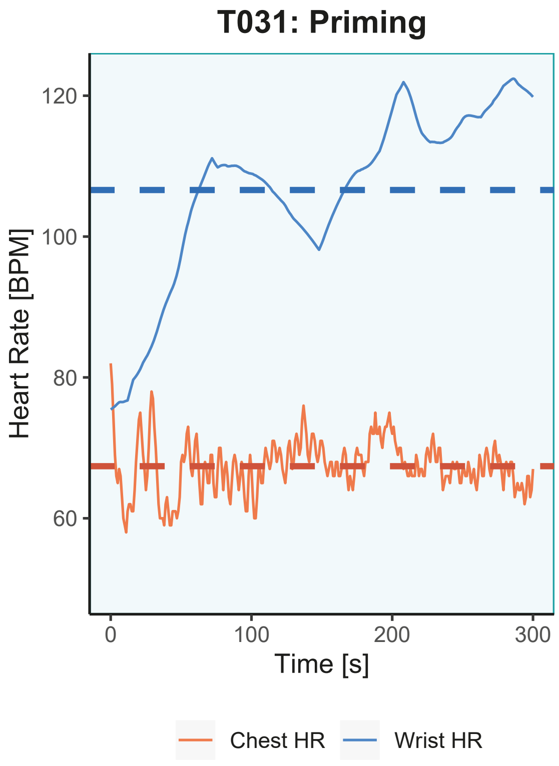
T031: Priming
Heart Rate [BPM]
Time [s]
Chest HR
Wrist HR

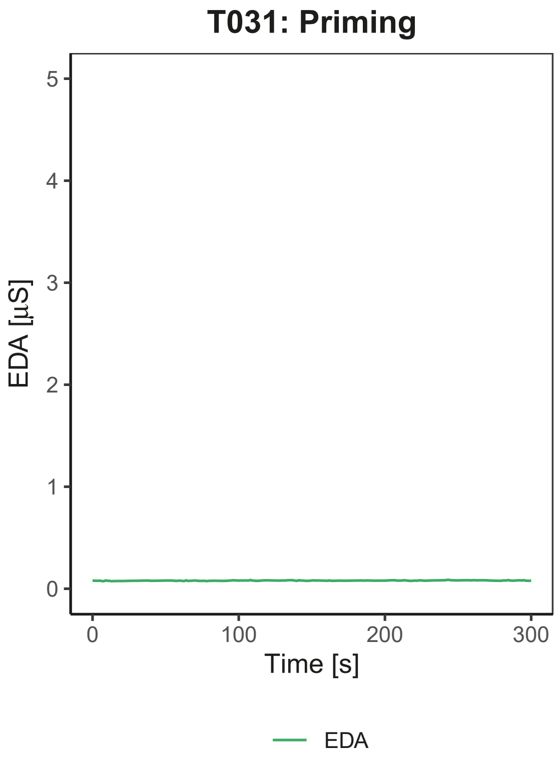
T031: Priming
EDA [µS]
Time [s]
EDA

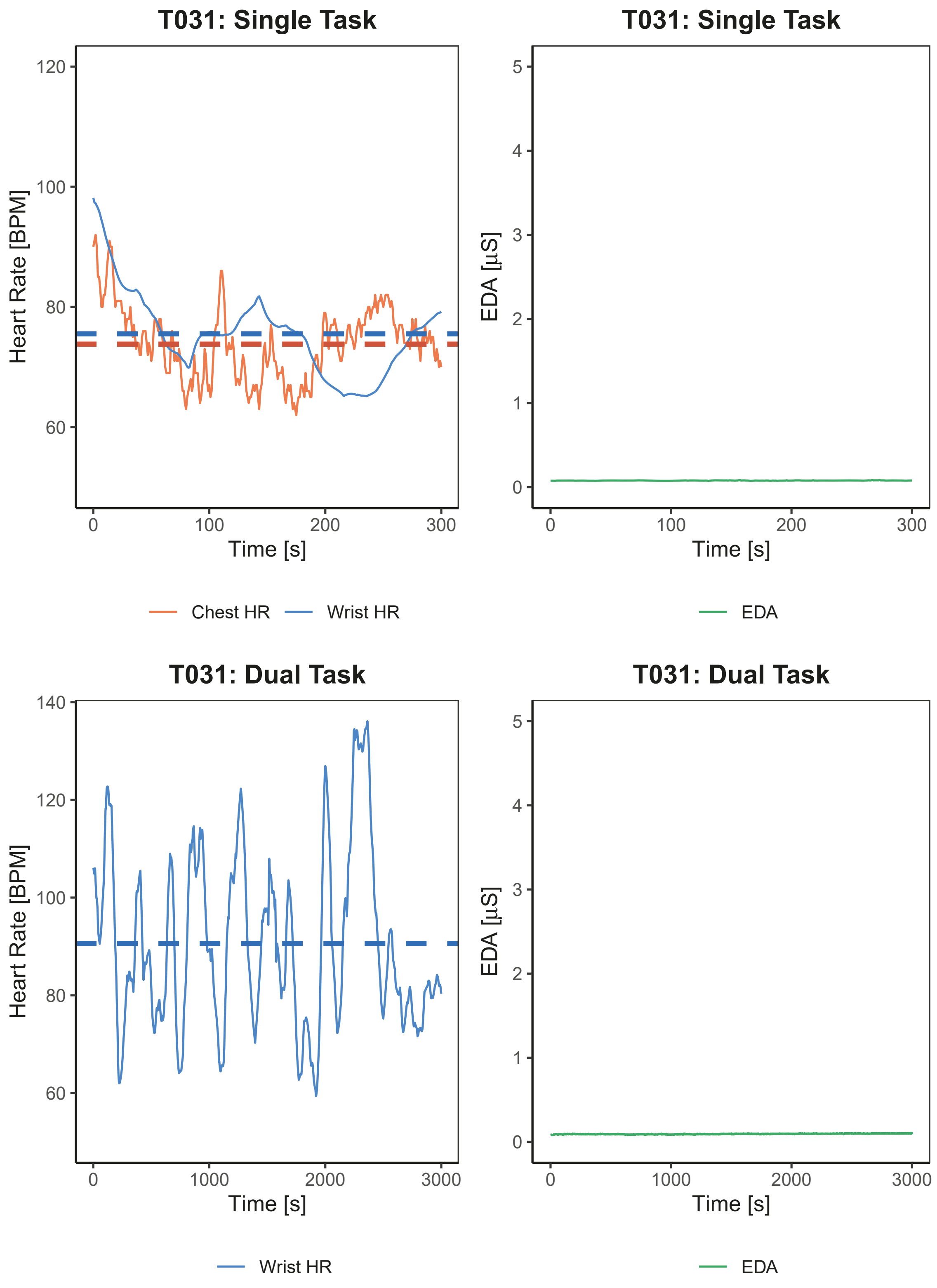

T031: Single Task
Heart Rate [BPM]
Time [s]
Chest HR
Wrist HR
T031: Single Task
EDA [µS]
Time [s]
EDA
T031: Dual Task
Heart Rate [BPM]
Time [s]
Wrist HR
T031: Dual Task
EDA [µS]
Time [s]
EDA

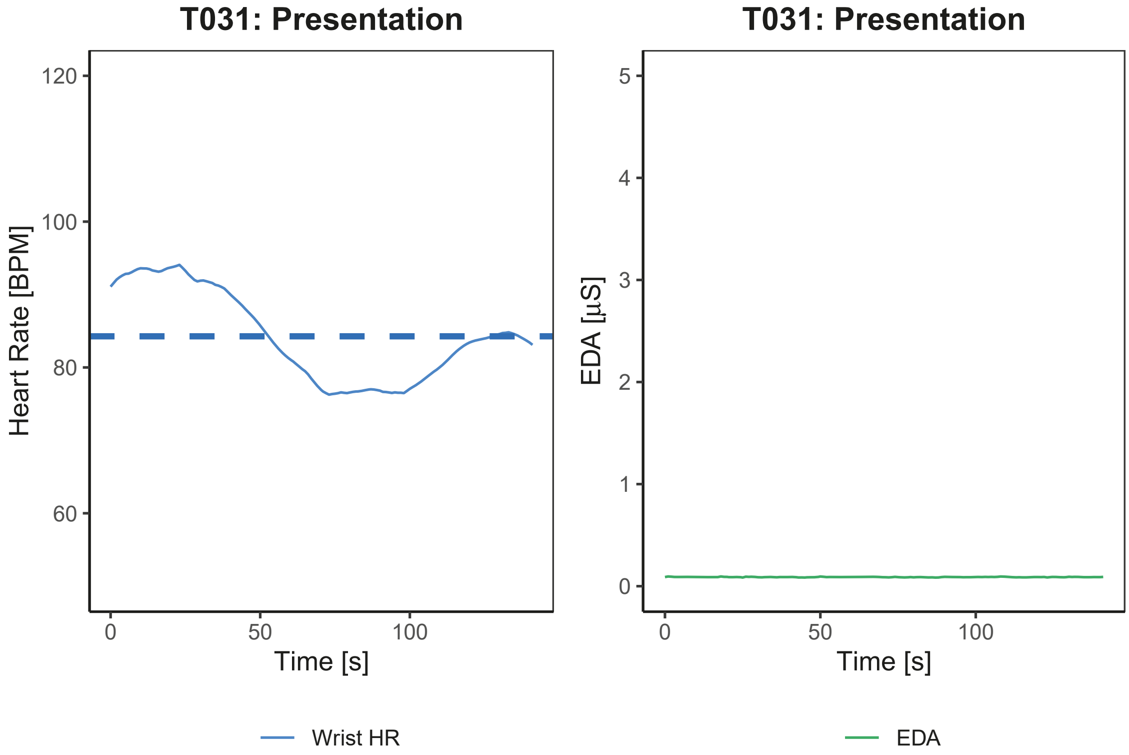
T031: Presentation
Heart Rate [BPM]
120
100
80
60
0
50
100
Time [s]
Wrist HR
T031: Presentation
EDA [µS]
5
4
3
2
1
0
0
50
100
Time [s]
EDA

--

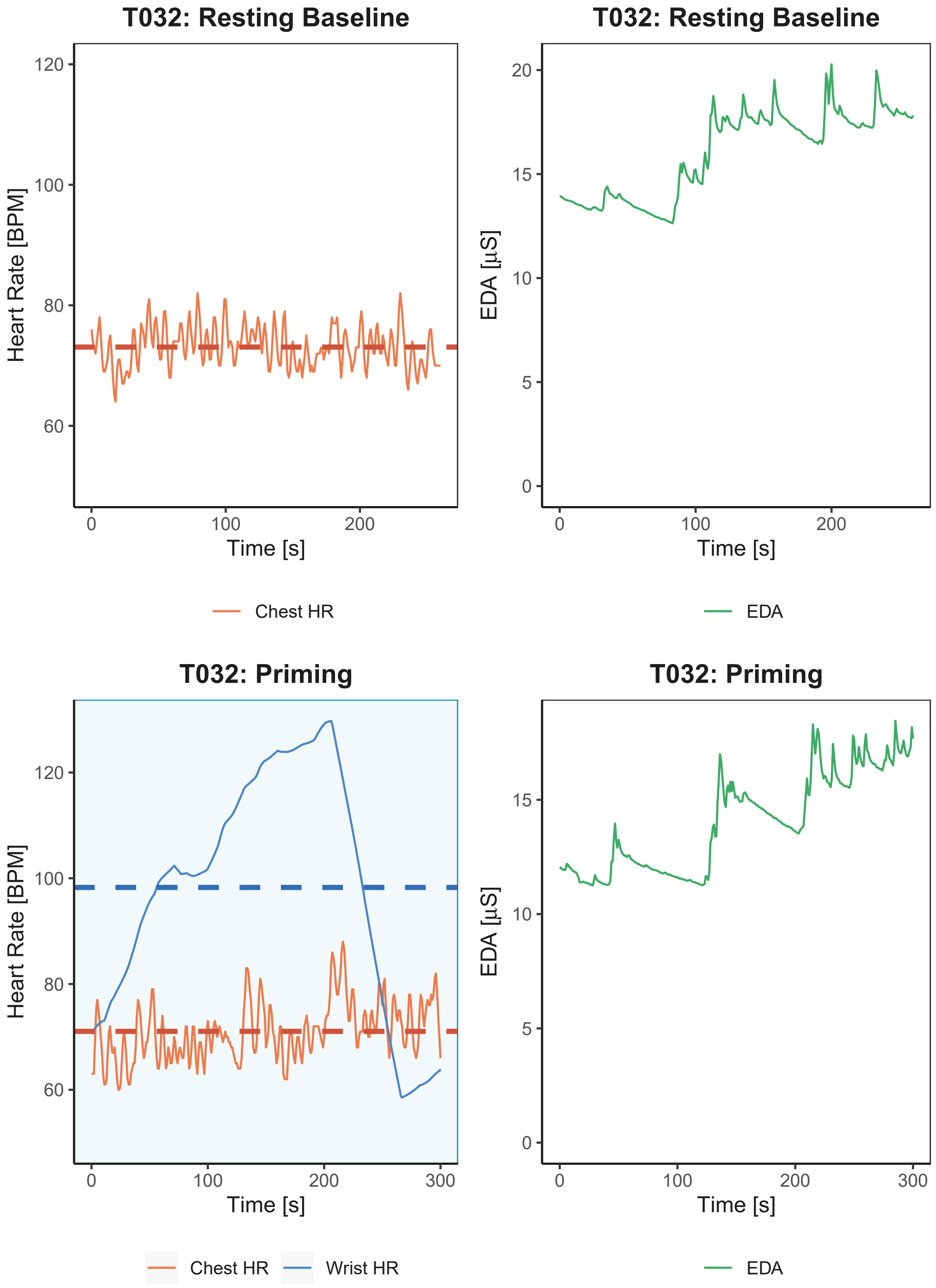
T032: Resting Baseline
Heart Rate [BPM]
Time [s]
Chest HR
T032: Resting Baseline
EDA [µS]
Time [s]
EDA
T032: Priming
Heart Rate [BPM]
Time [s]
Chest HR
Wrist HR
T032: Priming
EDA [µS]
Time [s]
EDA

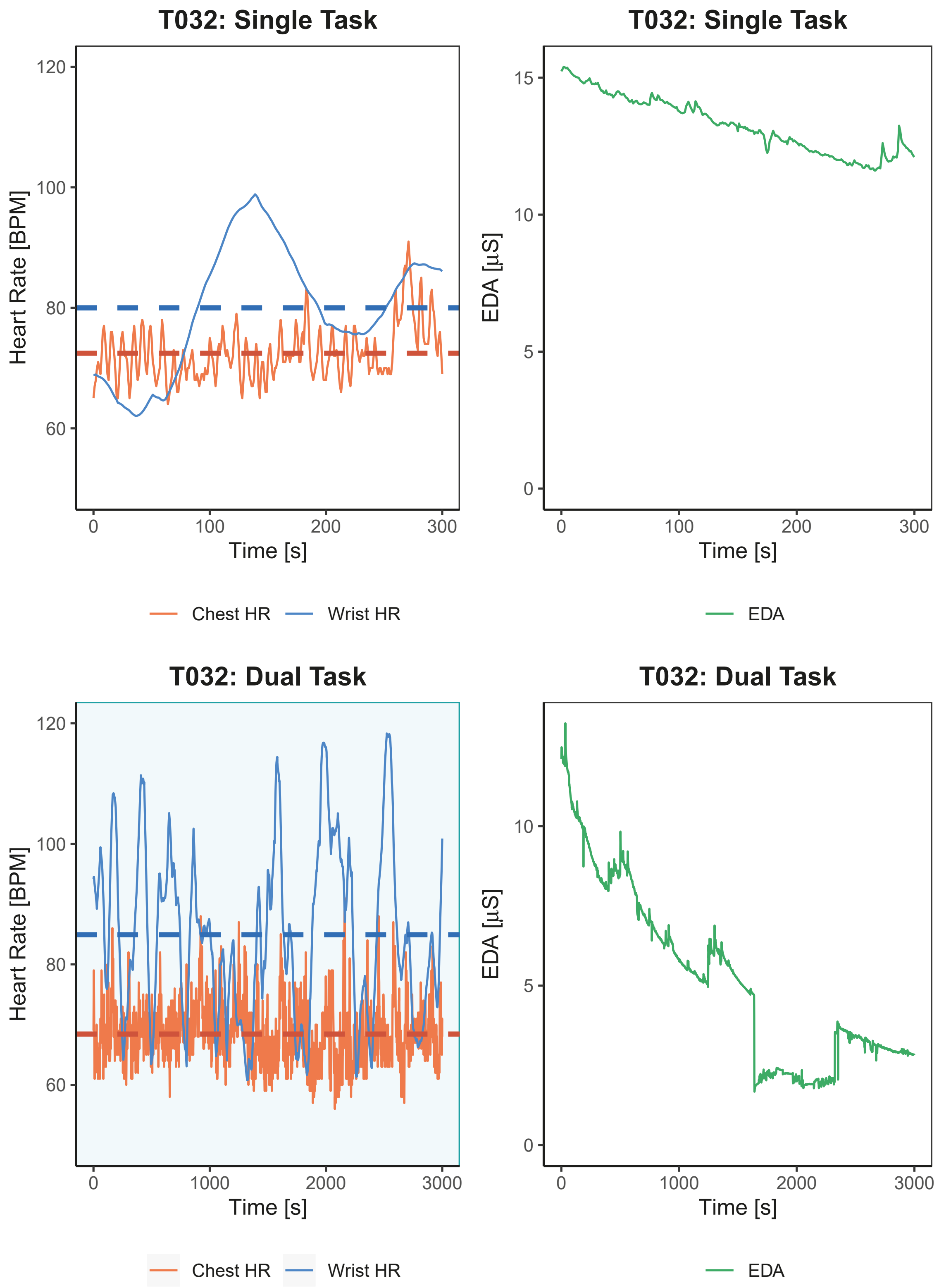
T032: Single Task
Heart Rate [BPM]
120
100
80
60
0
100
200
300
Time [s]
Chest HR
Wrist HR
T032: Single Task
EDA [µS]
15
10
5
0
0
100
200
300
Time [s]
EDA
T032: Dual Task
Heart Rate [BPM]
120
100
80
60
0
1000
2000
3000
Time [s]
Chest HR
Wrist HR
T032: Dual Task
EDA [µS]
10
5
0
0
1000
2000
3000
Time [s]
EDA

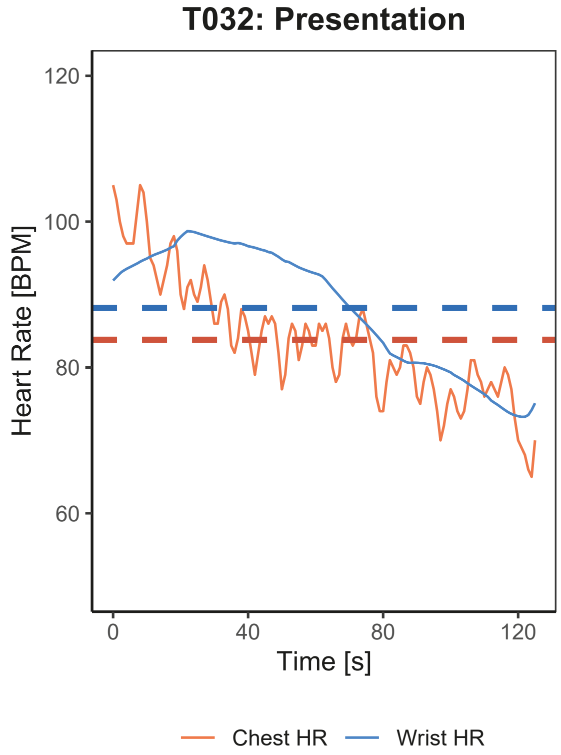

T032: Presentation
Heart Rate [BPM]
120
100
80
60
0
40
80
120
Time [s]
Chest HR
Wrist HR

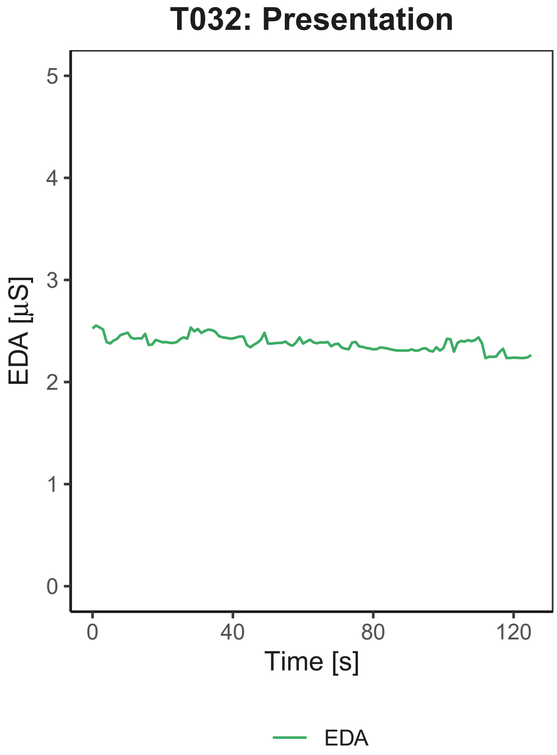

T032: Presentation
EDA [µS]
5
4
3
2
1
0
0
40
80
120
Time [s]
EDA


```
## ----------------------------------------------------------------------------- ##
```

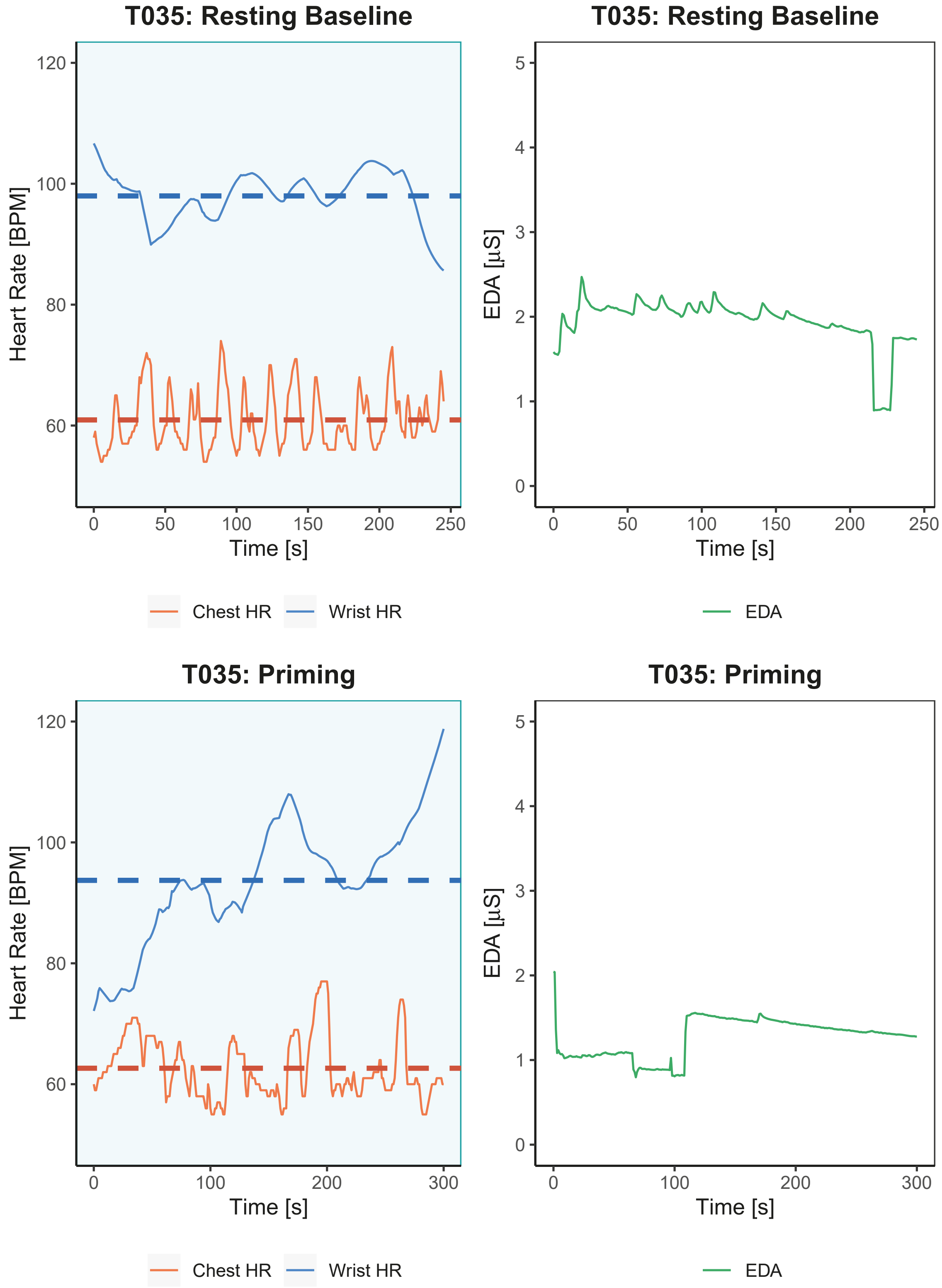
T035: Resting Baseline
Heart Rate [BPM]
Time [s]
Chest HR
Wrist HR
T035: Resting Baseline
EDA [µS]
Time [s]
EDA
T035: Priming
Heart Rate [BPM]
Time [s]
Chest HR
Wrist HR
T035: Priming
EDA [µS]
Time [s]
EDA

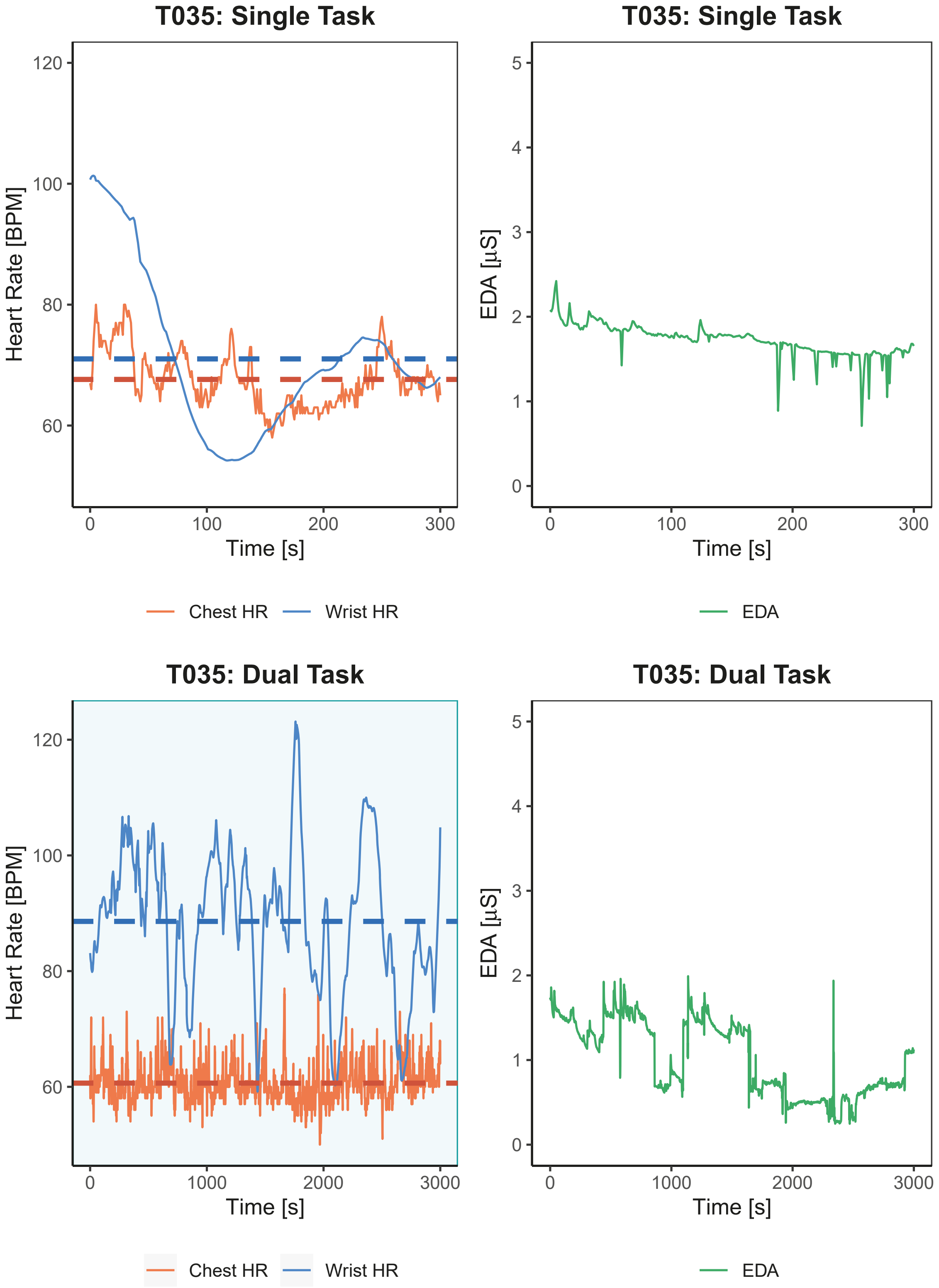
T035: Single Task
Heart Rate [BPM]
Time [s]
Chest HR
Wrist HR
T035: Single Task
EDA [µS]
Time [s]
EDA
T035: Dual Task
Heart Rate [BPM]
Time [s]
Chest HR
Wrist HR
T035: Dual Task
EDA [µS]
Time [s]
EDA

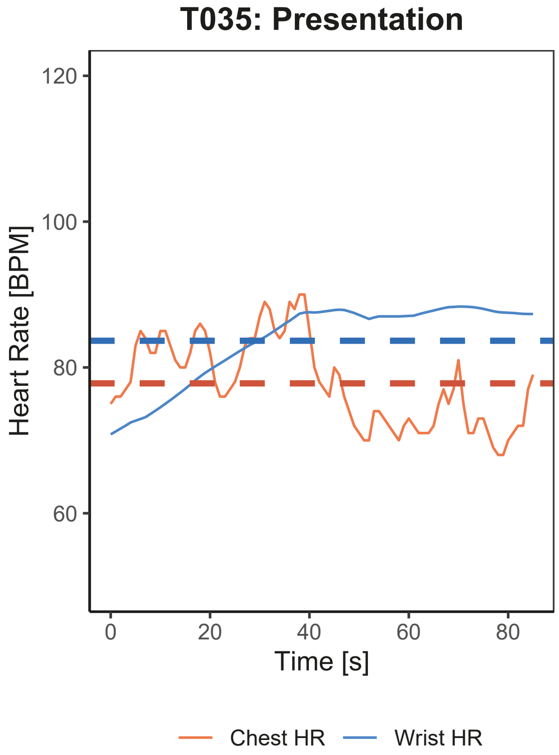
T035: Presentation
Heart Rate [BPM]
120
100
80
60
0
20
40
60
80
Time [s]
Chest HR
Wrist HR

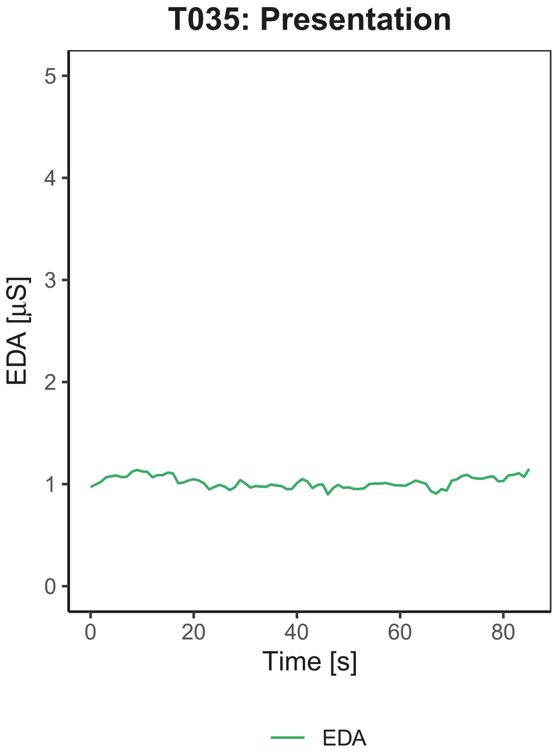
T035: Presentation
EDA [µS]
5
4
3
2
1
0
0
20
40
60
80
Time [s]
EDA

--

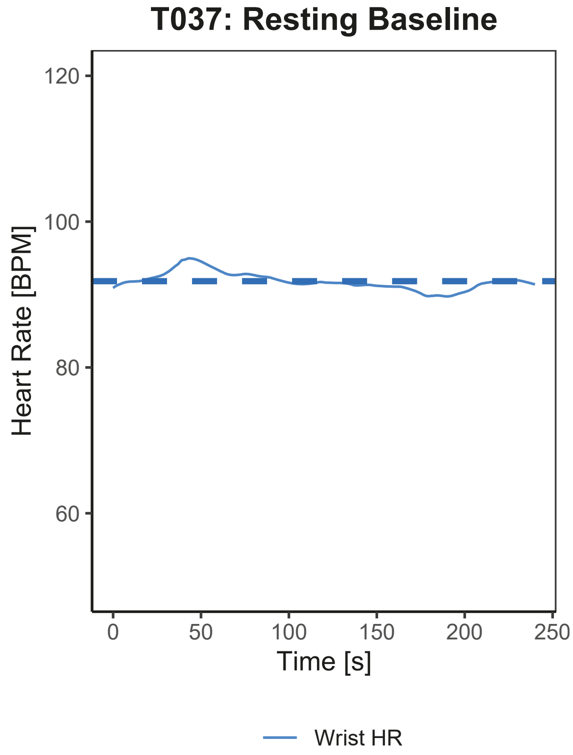
T037: Resting Baseline
Heart Rate [BPM]
Time [s]
Wrist HR

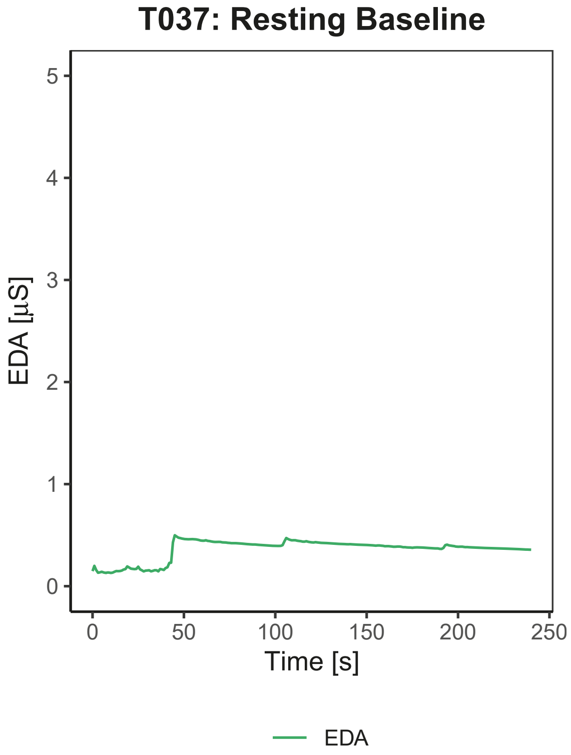
T037: Resting Baseline
EDA [µS]
Time [s]
EDA

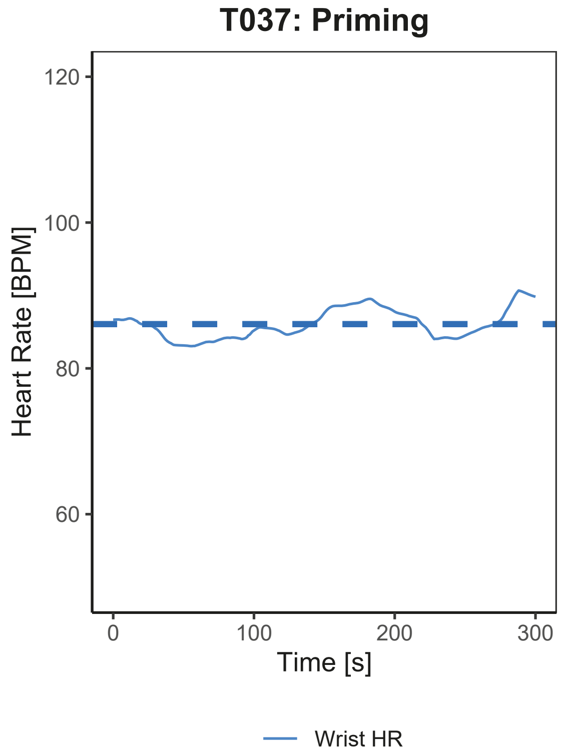
T037: Priming
Heart Rate [BPM]
Time [s]
Wrist HR

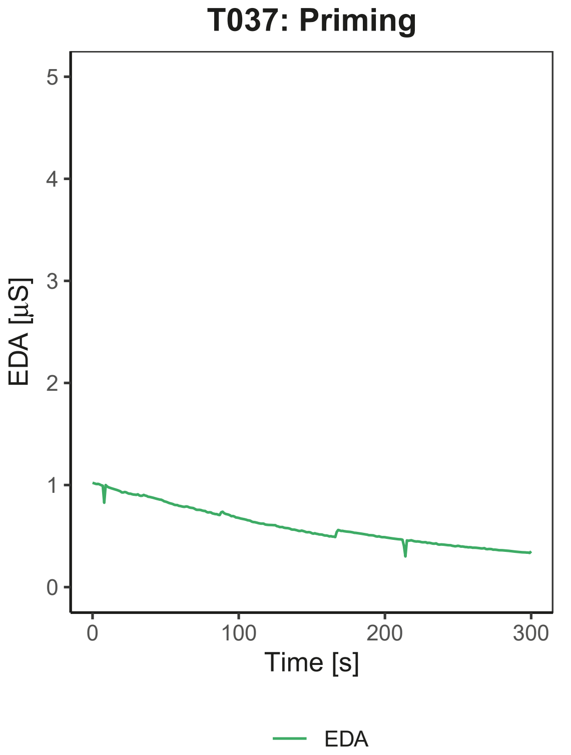
T037: Priming
EDA [µS]
Time [s]
EDA

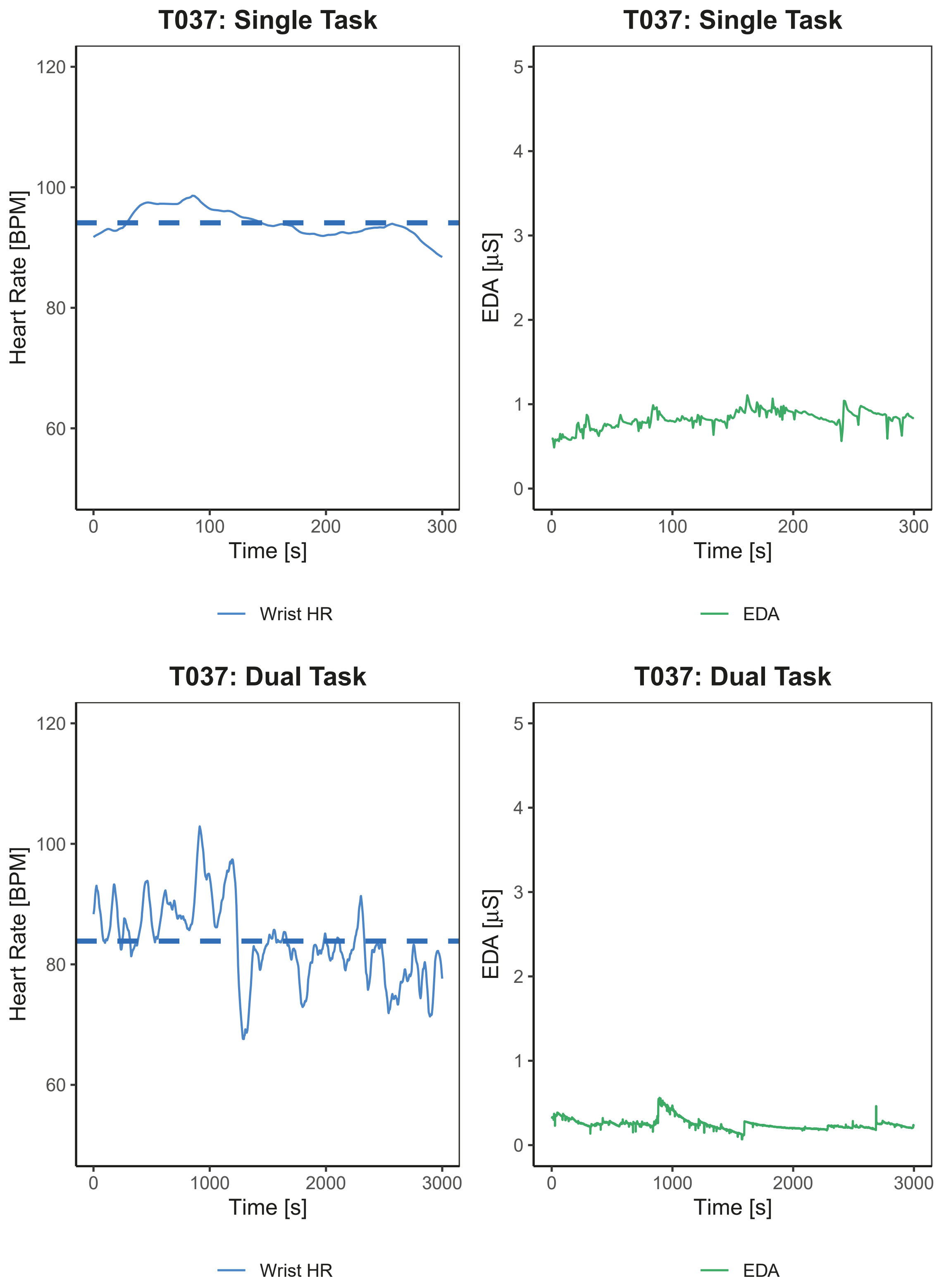
T037: Single Task
Heart Rate [BPM]
Time [s]
Wrist HR
T037: Single Task
EDA [µS]
Time [s]
EDA
T037: Dual Task
Heart Rate [BPM]
Time [s]
Wrist HR
T037: Dual Task
EDA [µS]
Time [s]
EDA

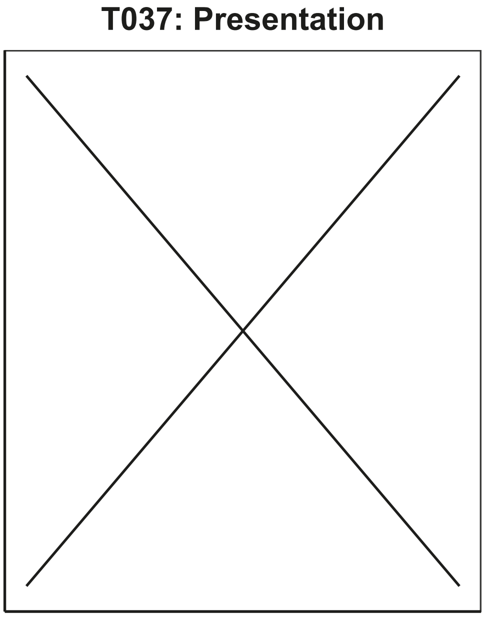
T037: Presentation

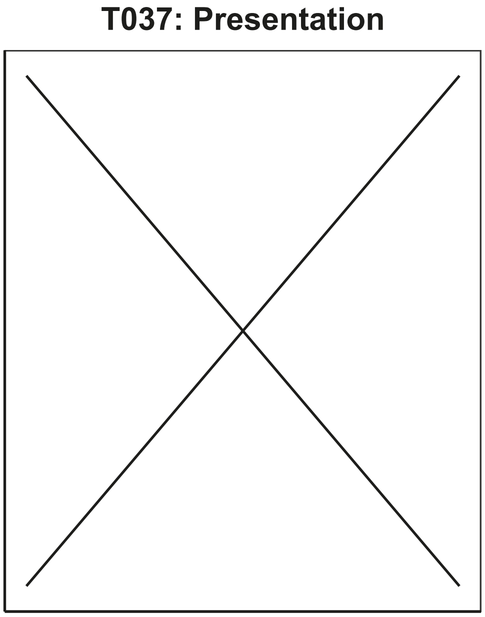
T037: Presentation

```
## ---------------------------------------------------------------------------------- ##
```

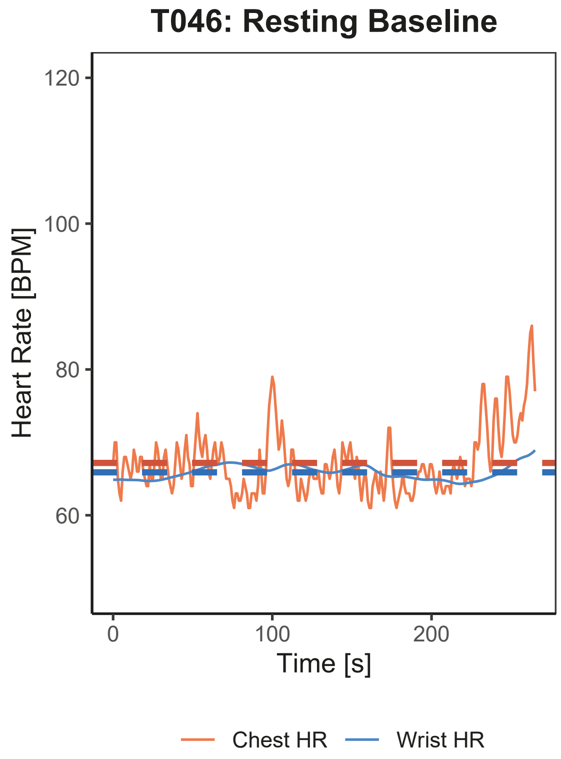
T046: Resting Baseline
Heart Rate [BPM]
120
100
80
60
0
100
200
Time [s]
Chest HR
Wrist HR

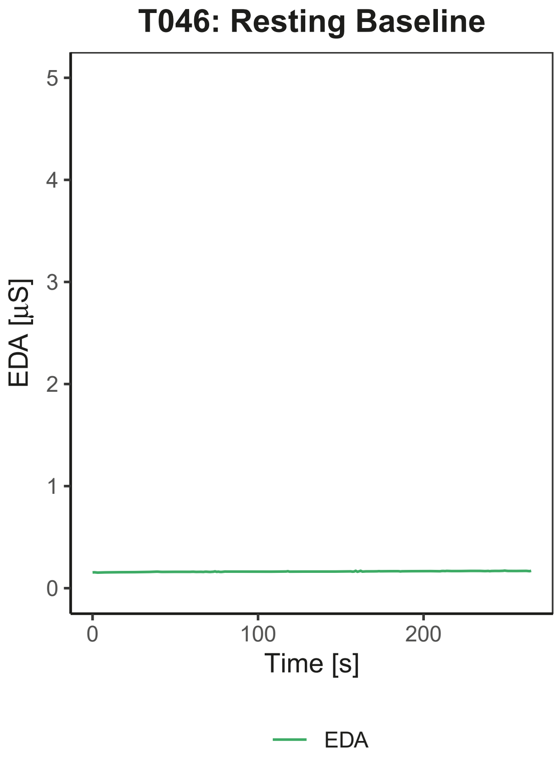
T046: Resting Baseline
EDA [µS]
5
4
3
2
1
0
0
100
200
Time [s]
EDA

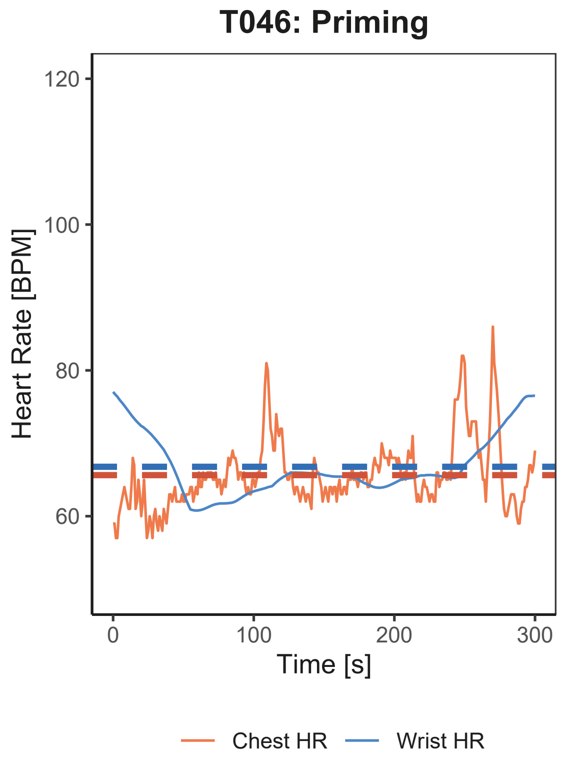
T046: Priming
Heart Rate [BPM]
120
100
80
60
0
100
200
300
Time [s]
Chest HR
Wrist HR

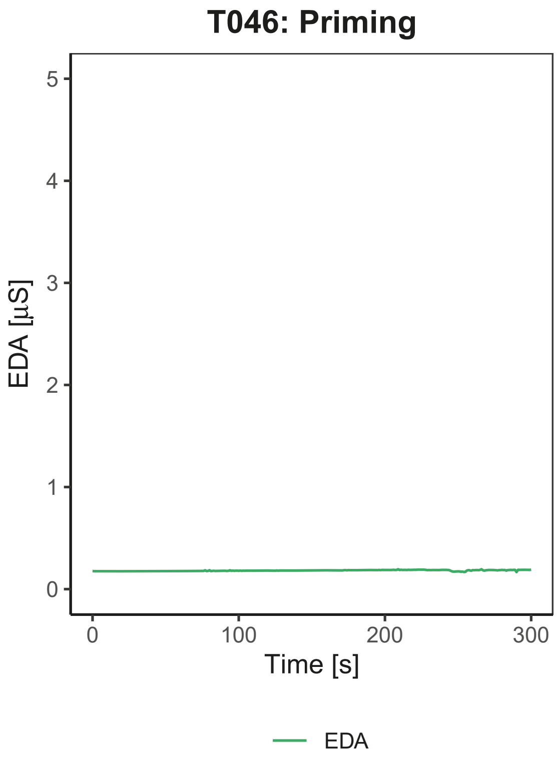
T046: Priming
EDA [µS]
5
4
3
2
1
0
0
100
200
300
Time [s]
EDA

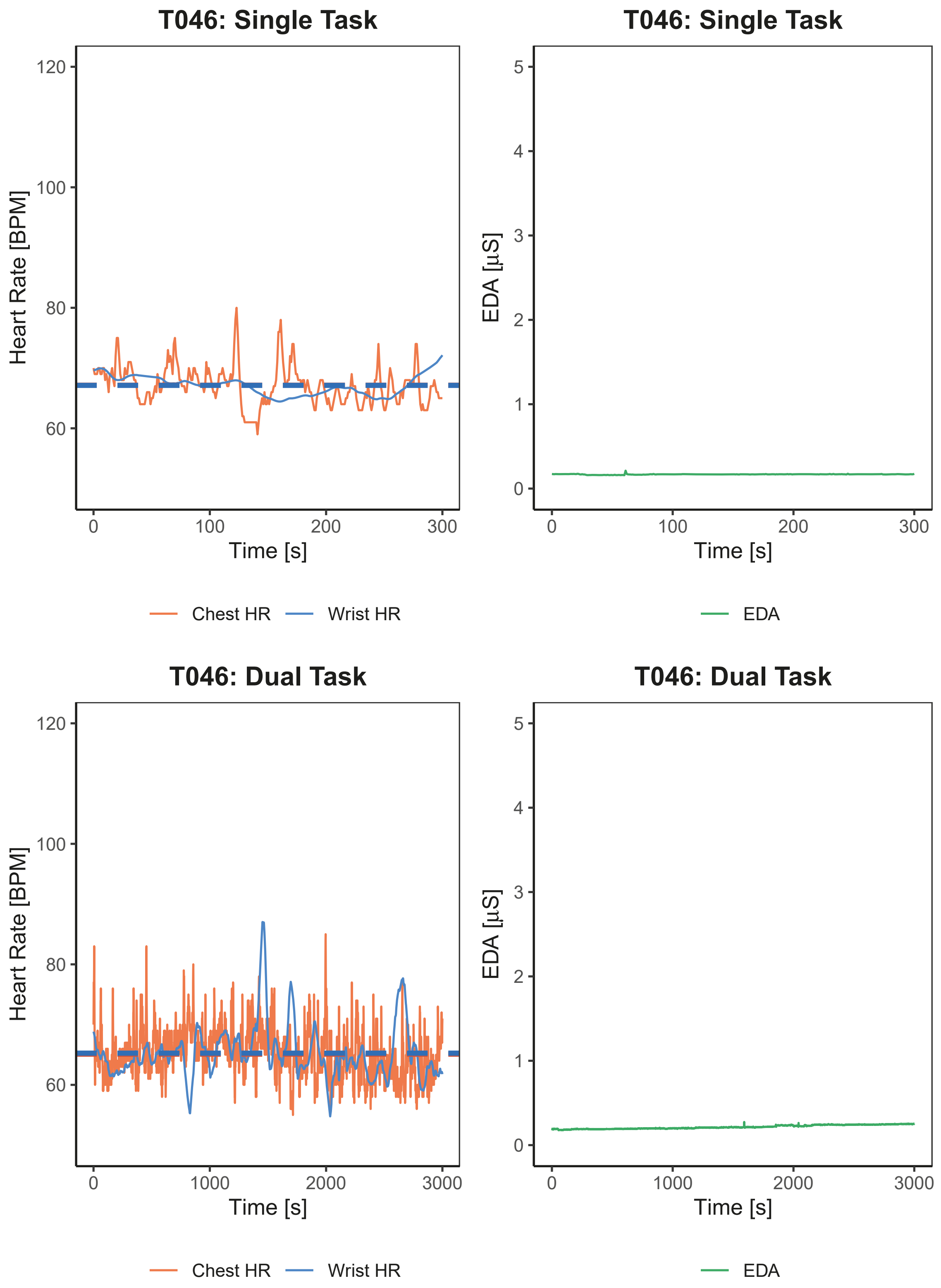
T046: Single Task
Heart Rate [BPM]
Time [s]
Chest HR
Wrist HR
T046: Single Task
EDA [µS]
Time [s]
EDA
T046: Dual Task
Heart Rate [BPM]
Time [s]
Chest HR
Wrist HR
T046: Dual Task
EDA [µS]
Time [s]
EDA

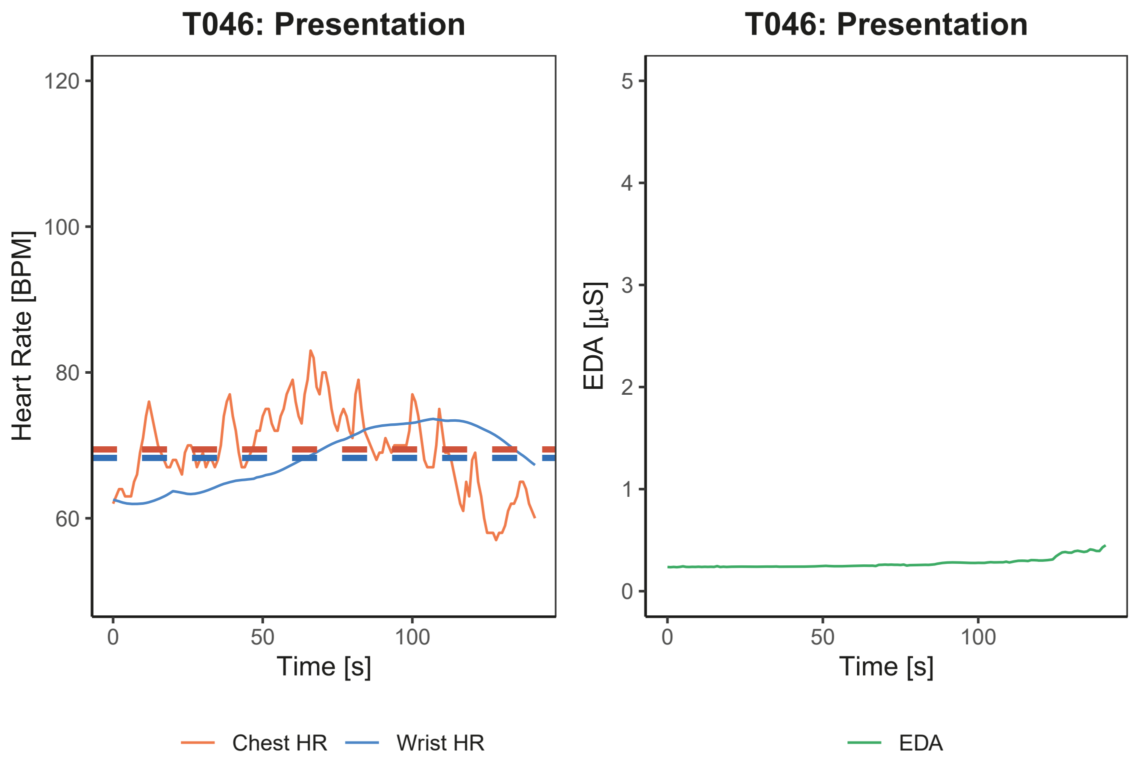
T046: Presentation
Heart Rate [BPM]
120
100
80
60
0
50
100
Time [s]
Chest HR
Wrist HR
T046: Presentation
EDA [µS]
5
4
3
2
1
0
0
50
100
Time [s]
EDA

--

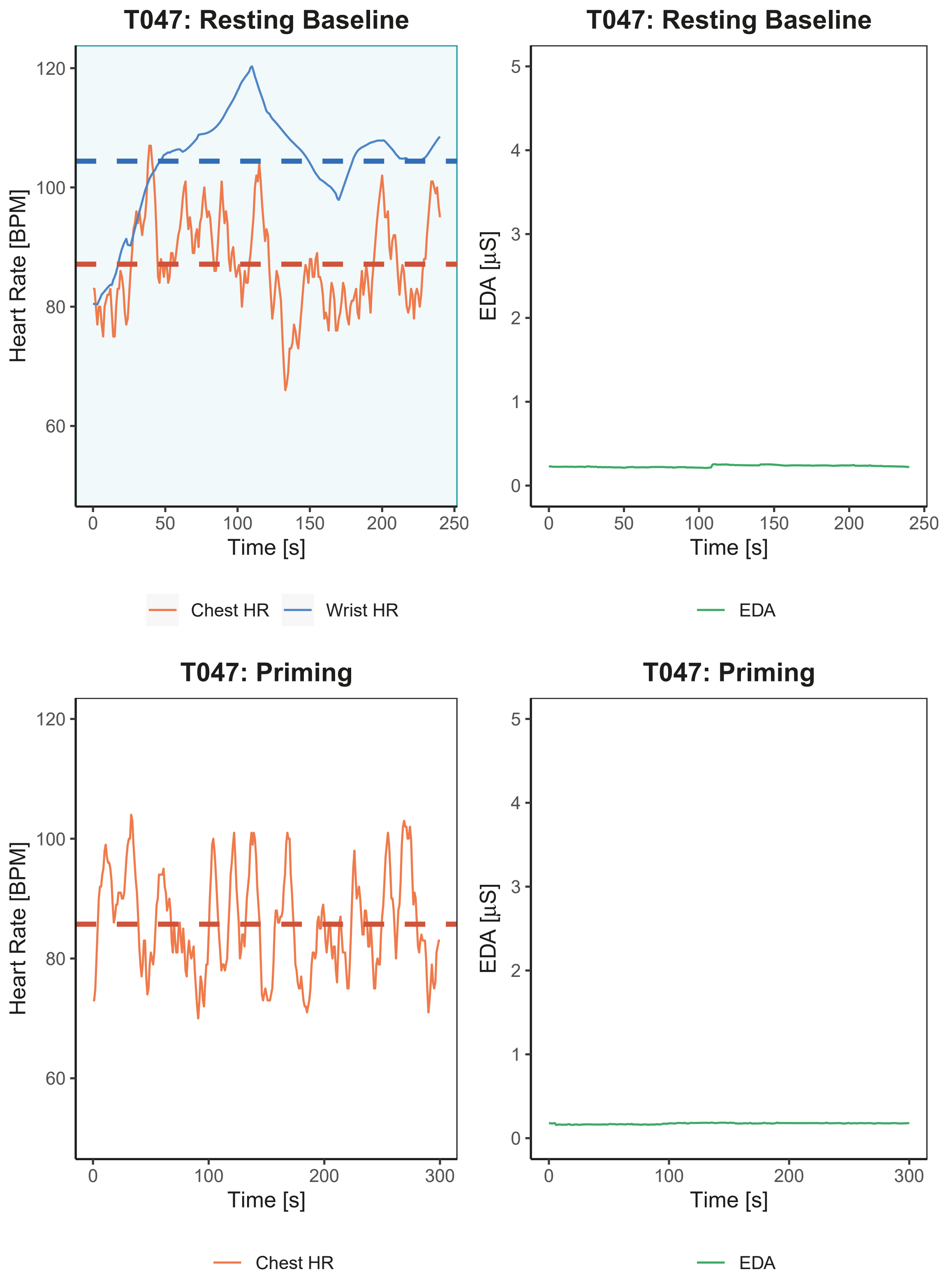
T047: Resting Baseline
Heart Rate [BPM]
Time [s]
EDA [µS]
Chest HR
Wrist HR
EDA
T047: Priming
Heart Rate [BPM]
Time [s]
EDA [µS]
Chest HR
EDA

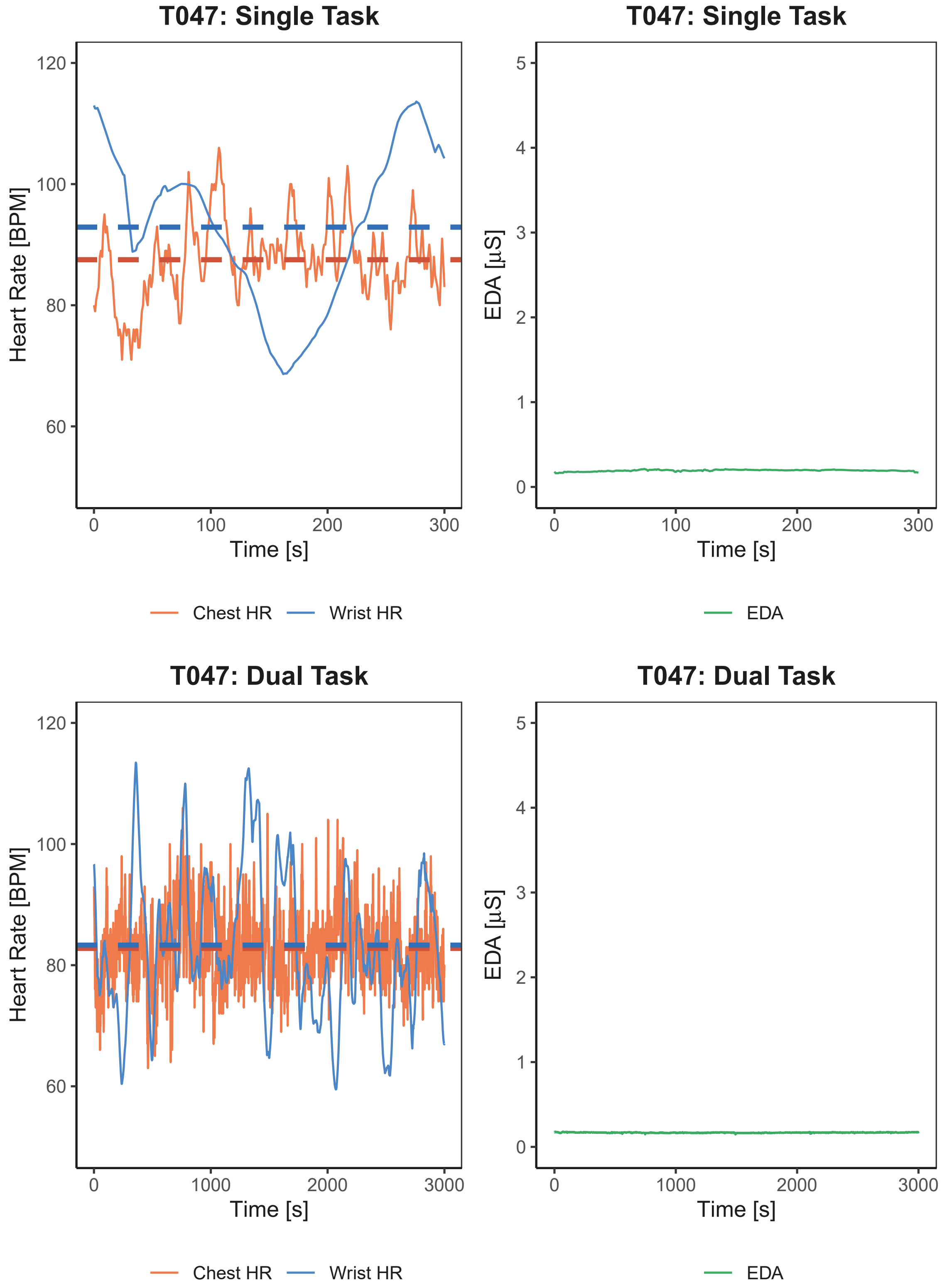
T047: Single Task
Heart Rate [BPM]
120
100
80
60
0
100
200
300
Time [s]
Chest HR
Wrist HR
T047: Single Task
EDA [µS]
5
4
3
2
1
0
0
100
200
300
Time [s]
EDA
T047: Dual Task
Heart Rate [BPM]
120
100
80
60
0
1000
2000
3000
Time [s]
Chest HR
Wrist HR
T047: Dual Task
EDA [µS]
5
4
3
2
1
0
0
1000
2000
3000
Time [s]
EDA

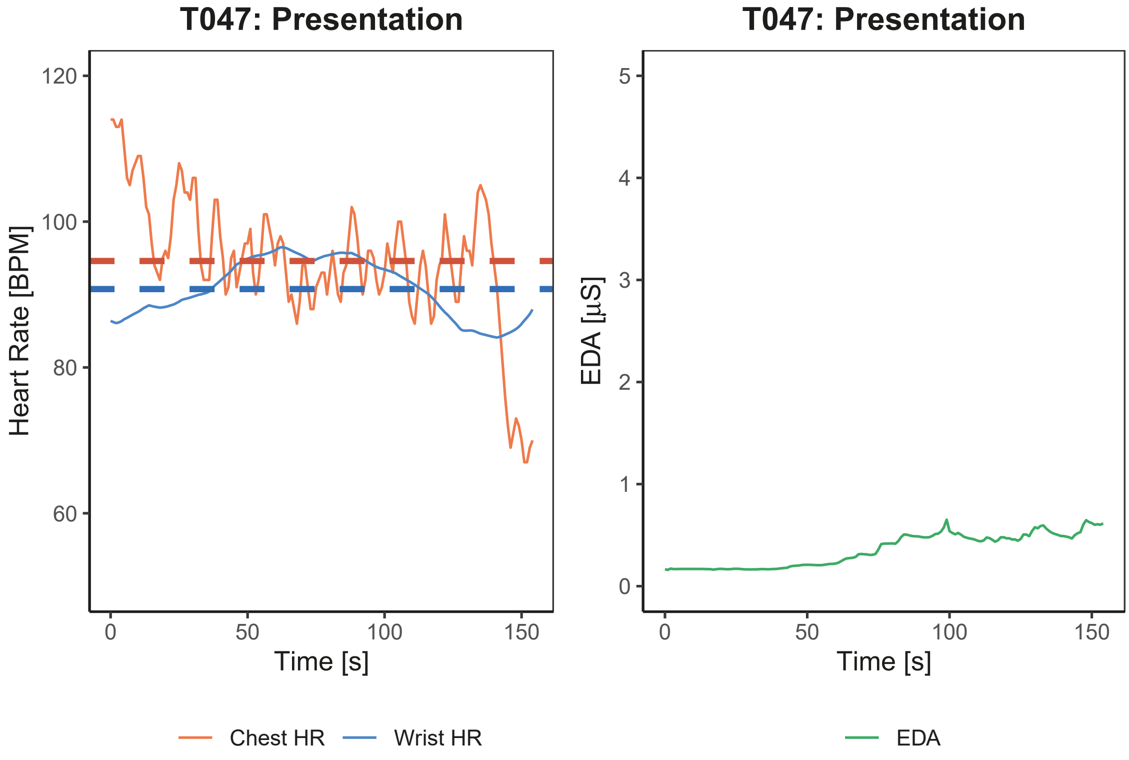

T047: Presentation
Heart Rate [BPM]
120
100
80
60
0
50
100
150
Time [s]
T047: Presentation
EDA [µS]
5
4
3
2
1
0
0
50
100
150
Time [s]
Chest HR
Wrist HR
EDA

--

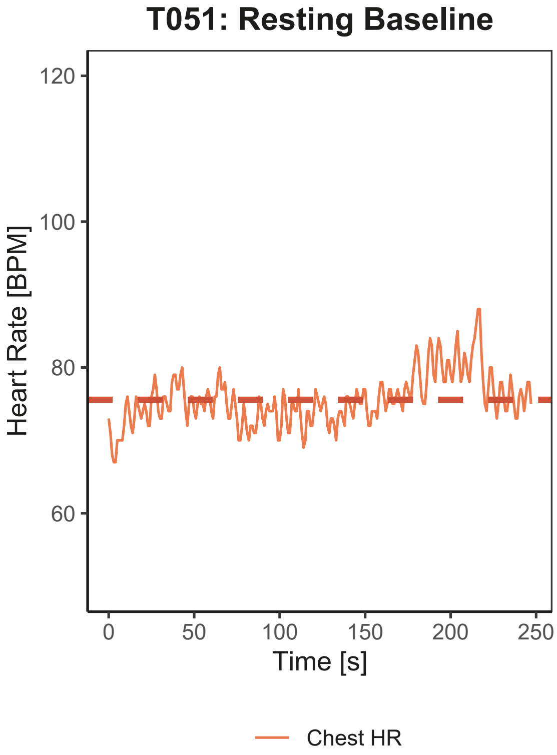
T051: Resting Baseline
Heart Rate [BPM]
120
100
80
60
0
50
100
150
200
250
Time [s]
Chest HR

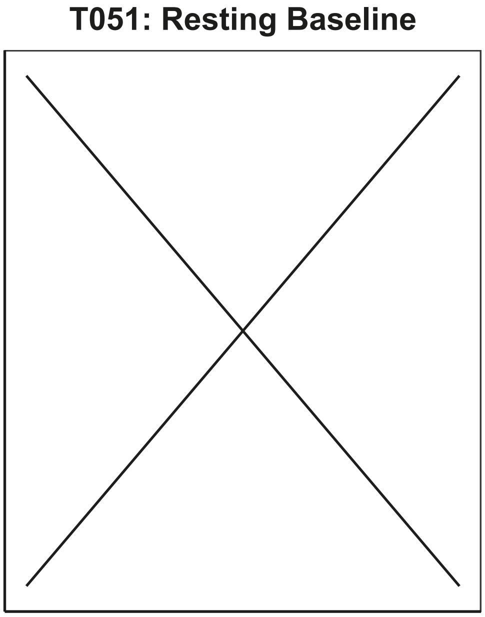
T051: Resting Baseline

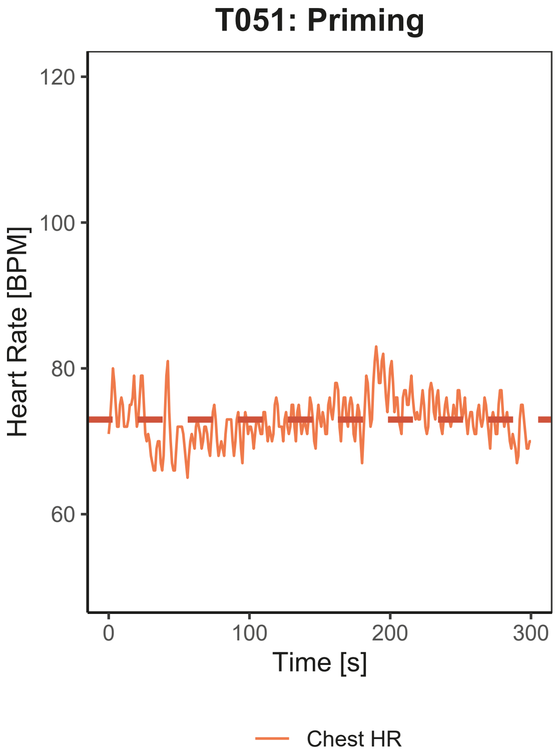
T051: Priming
Heart Rate [BPM]
120
100
80
60
0
100
200
300
Time [s]
Chest HR

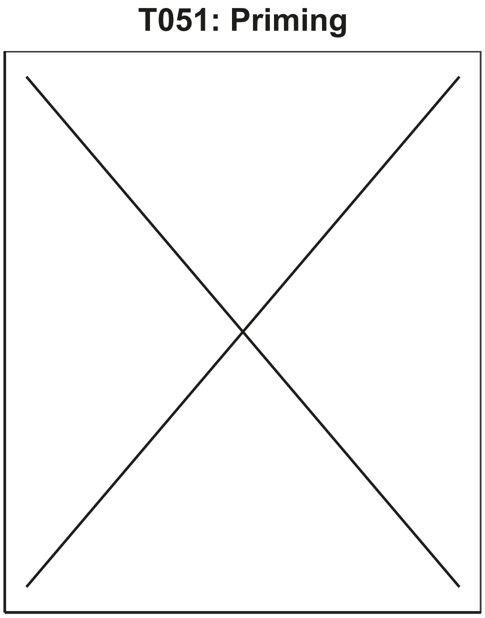
T051: Priming

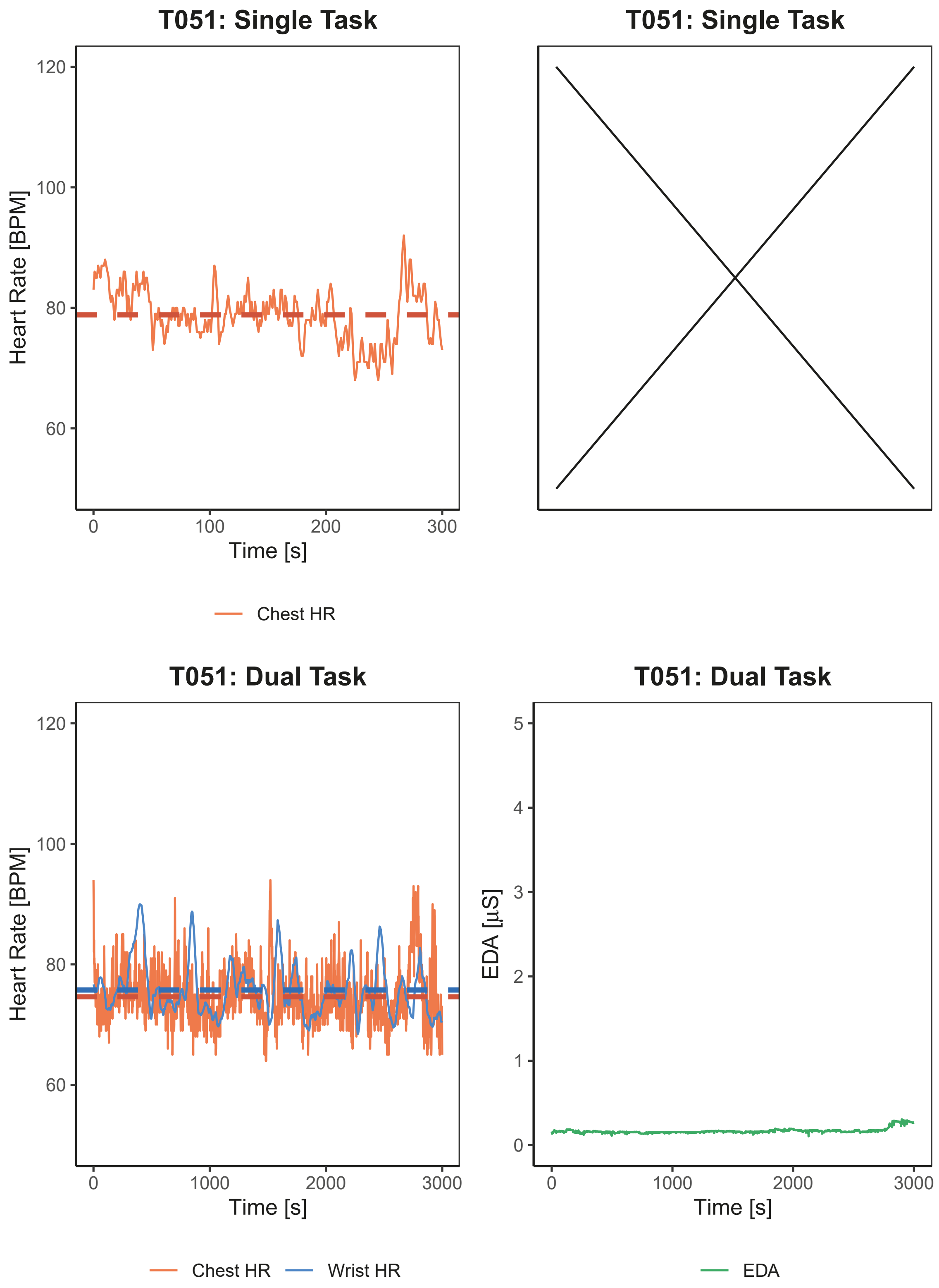
T051: Single Task
Heart Rate [BPM]
Time [s]
Chest HR
T051: Single Task
T051: Dual Task
Heart Rate [BPM]
Time [s]
Chest HR
Wrist HR
T051: Dual Task
EDA [µS]
Time [s]
EDA

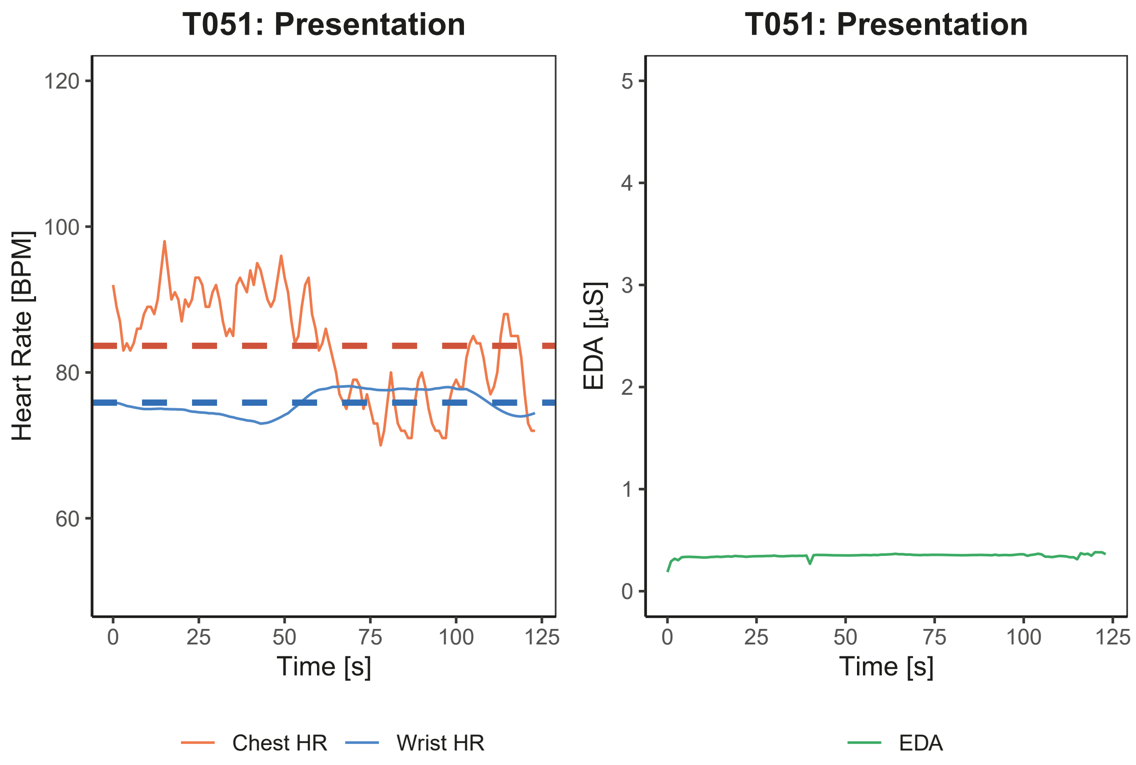
T051: Presentation
Heart Rate [BPM]
120
100
80
60
0
25
50
75
100
125
Time [s]
Chest HR
Wrist HR
T051: Presentation
EDA [µS]
5
4
3
2
1
0
0
25
50
75
100
125
Time [s]
EDA

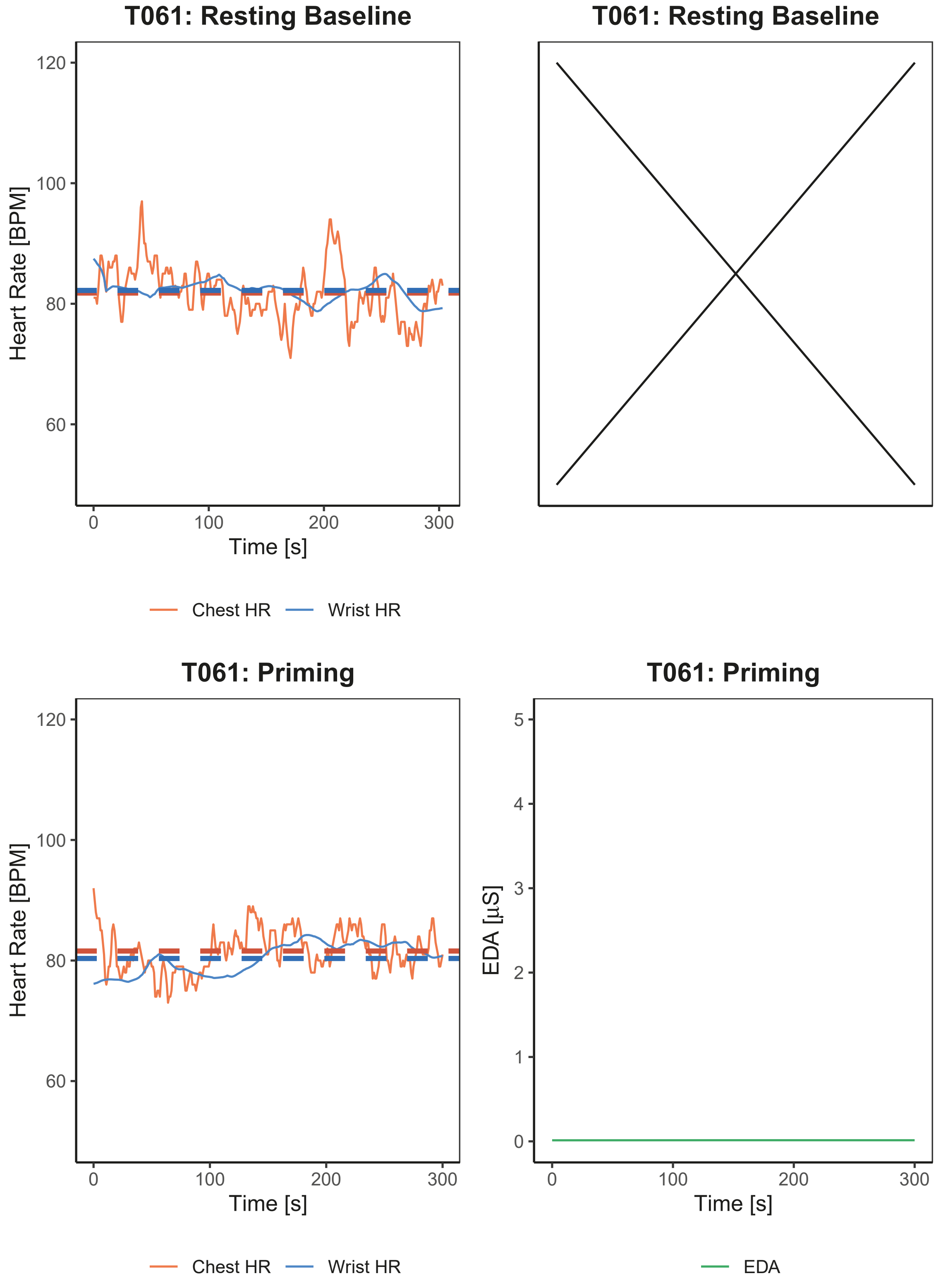
T061: Resting Baseline
Heart Rate [BPM]
120
100
80
60
0
100
200
300
Time [s]
T061: Resting Baseline
Chest HR
Wrist HR
T061: Priming
Heart Rate [BPM]
120
100
80
60
0
100
200
300
Time [s]
Chest HR
Wrist HR
T061: Priming
EDA [µS]
5
4
3
2
1
0
0
100
200
300
Time [s]
EDA

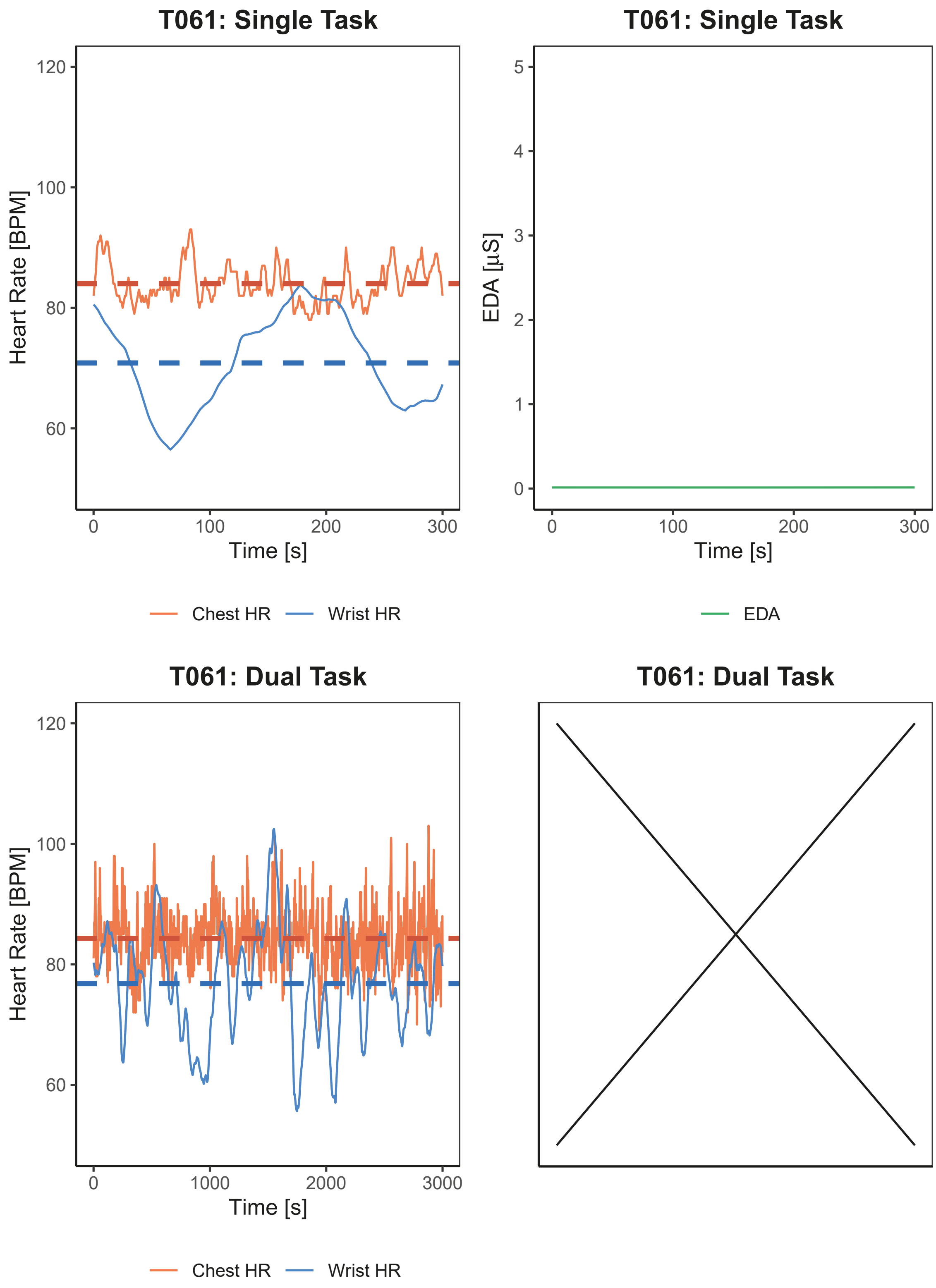
T061: Single Task
Heart Rate [BPM]
Time [s]
Chest HR
Wrist HR
T061: Single Task
EDA [µS]
Time [s]
EDA
T061: Dual Task
Heart Rate [BPM]
Time [s]
Chest HR
Wrist HR
T061: Dual Task

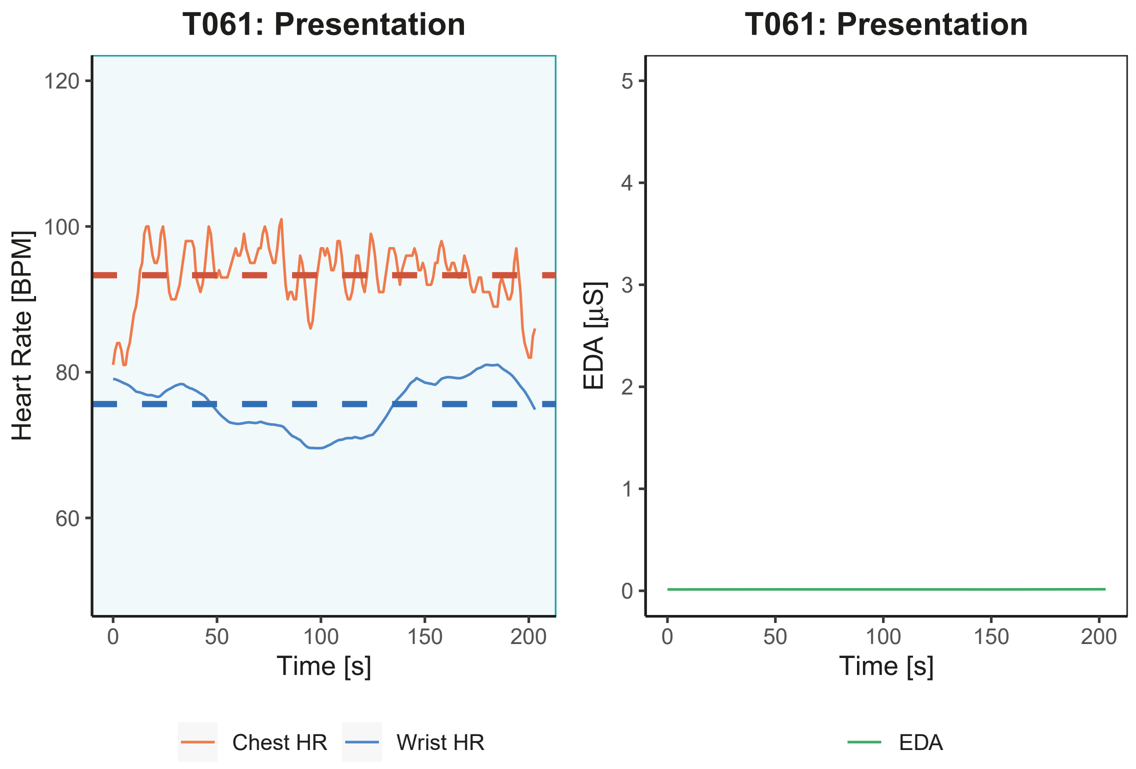
T061: Presentation
Heart Rate [BPM]
120
100
80
60
0
50
100
150
200
Time [s]
T061: Presentation
EDA [µS]
5
4
3
2
1
0
0
50
100
150
200
Time [s]
Chest HR
Wrist HR
EDA

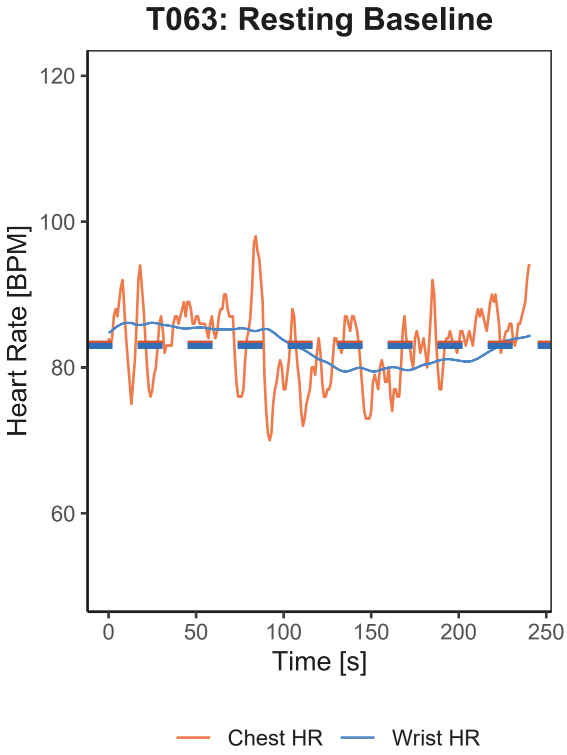
T063: Resting Baseline
Heart Rate [BPM]
120
100
80
60
0
50
100
150
200
250
Time [s]
Chest HR
Wrist HR

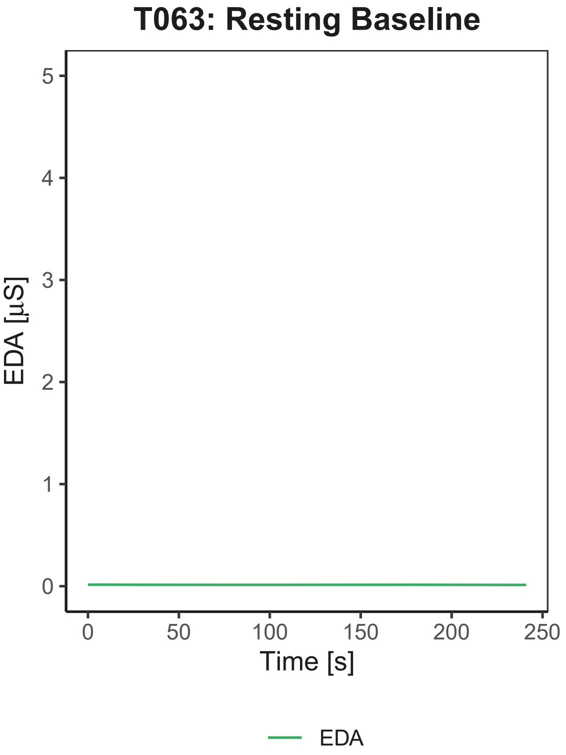
T063: Resting Baseline
EDA [µS]
5
4
3
2
1
0
0
50
100
150
200
250
Time [s]
EDA

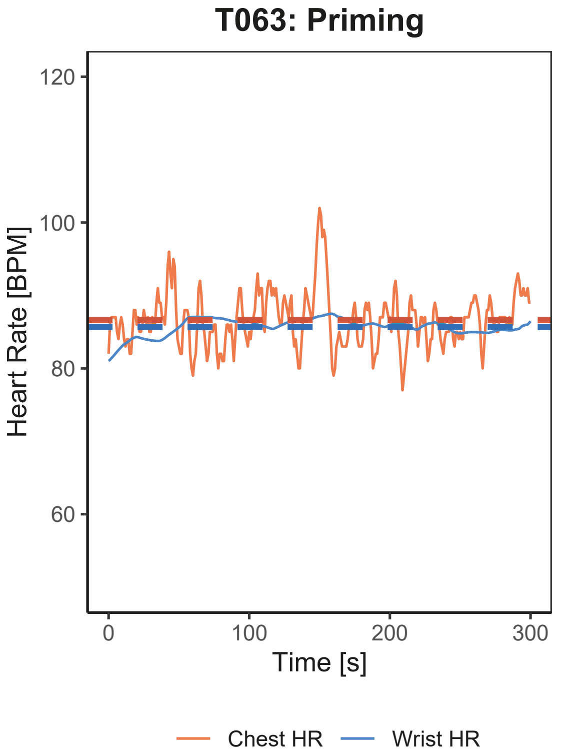
T063: Priming
Heart Rate [BPM]
120
100
80
60
0
100
200
300
Time [s]
Chest HR
Wrist HR

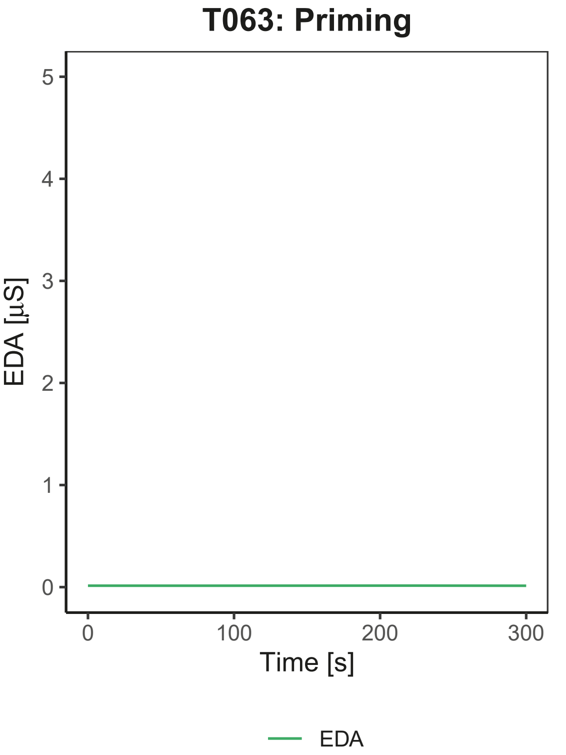
T063: Priming
EDA [µS]
5
4
3
2
1
0
0
100
200
300
Time [s]
EDA

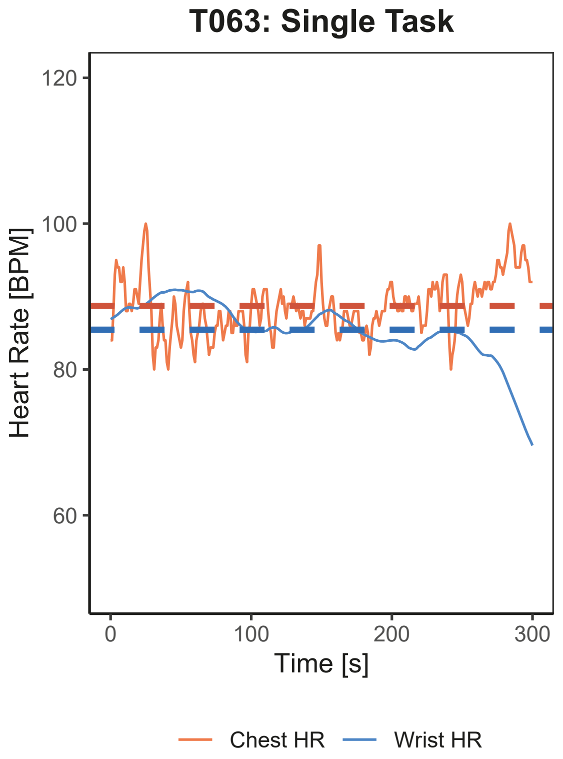
T063: Single Task
Heart Rate [BPM]
120
100
80
60
0
100
200
300
Time [s]
Chest HR
Wrist HR

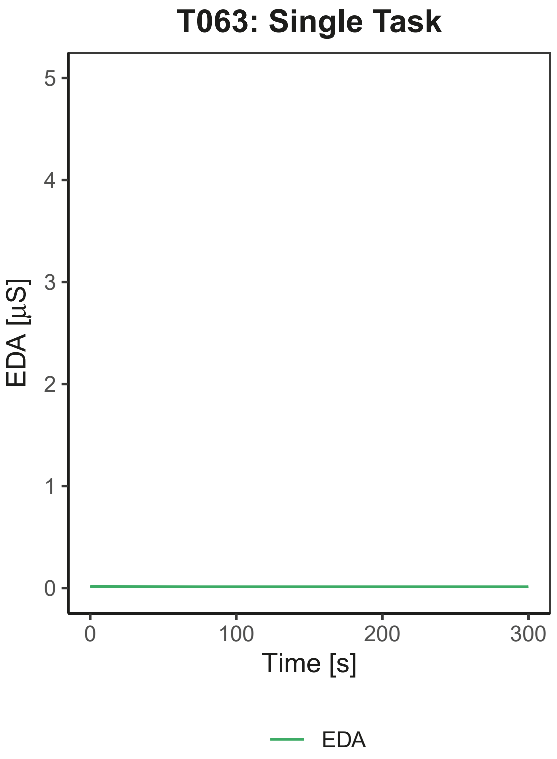
T063: Single Task
EDA [µS]
5
4
3
2
1
0
0
100
200
300
Time [s]
EDA

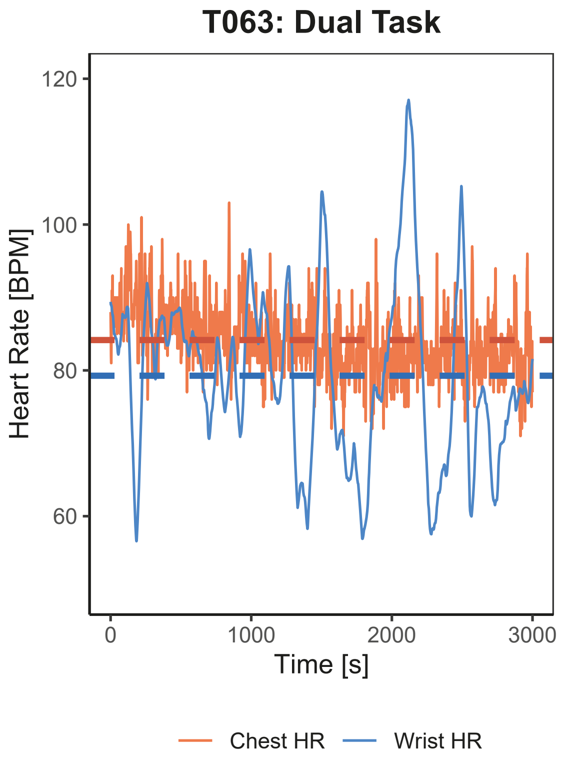
T063: Dual Task
Heart Rate [BPM]
120
100
80
60
0
1000
2000
3000
Time [s]
Chest HR
Wrist HR

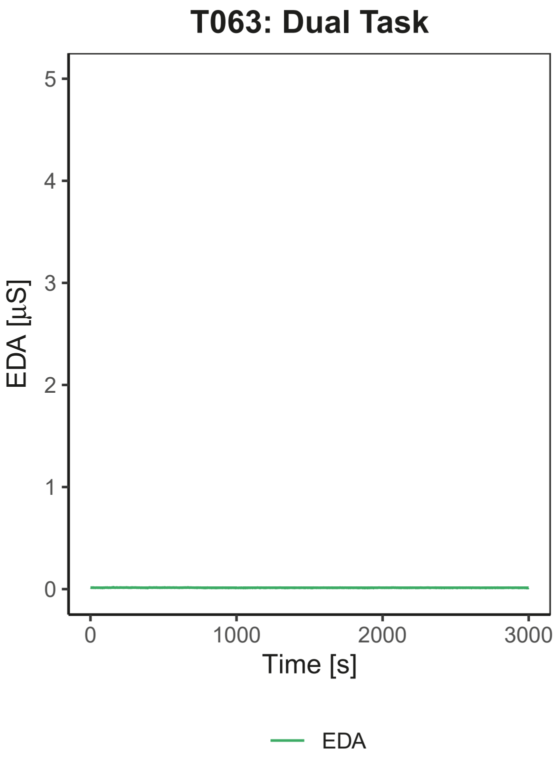
T063: Dual Task
EDA [µS]
5
4
3
2
1
0
0
1000
2000
3000
Time [s]
EDA

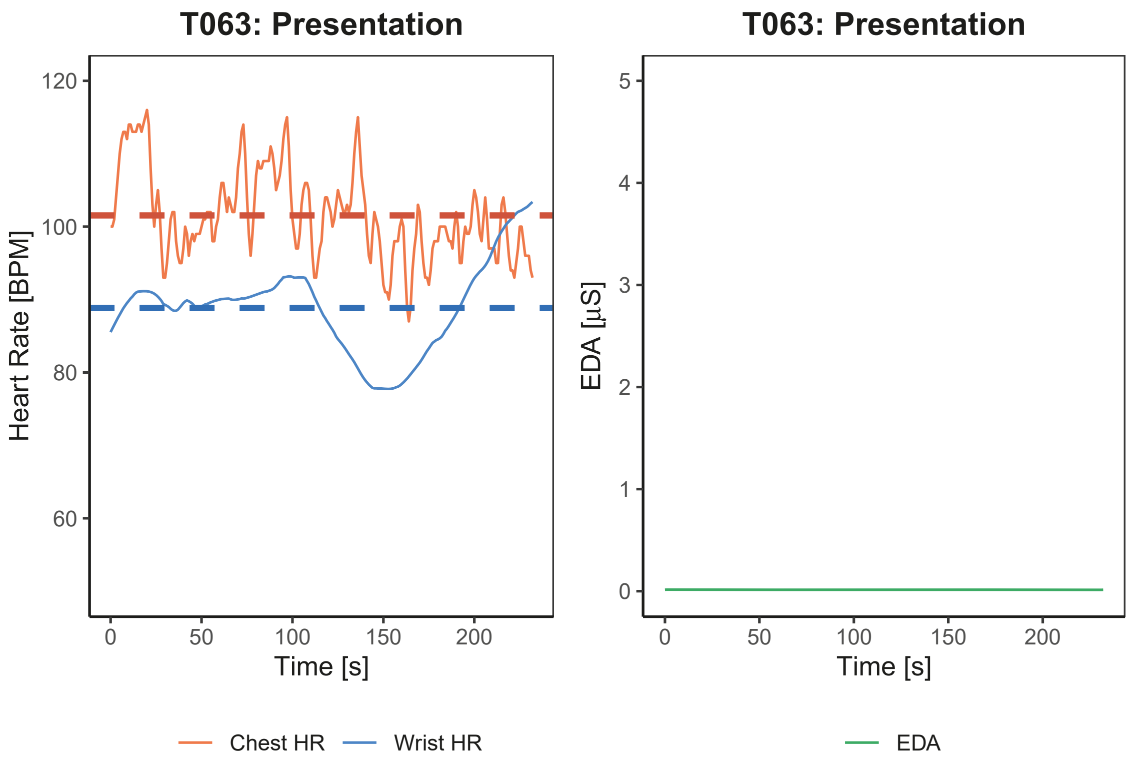

T063: Presentation
Heart Rate [BPM]
120
100
80
60
0
50
100
150
200
Time [s]
Chest HR
Wrist HR
T063: Presentation
EDA [µS]
5
4
3
2
1
0
0
50
100
150
200
Time [s]
EDA

--

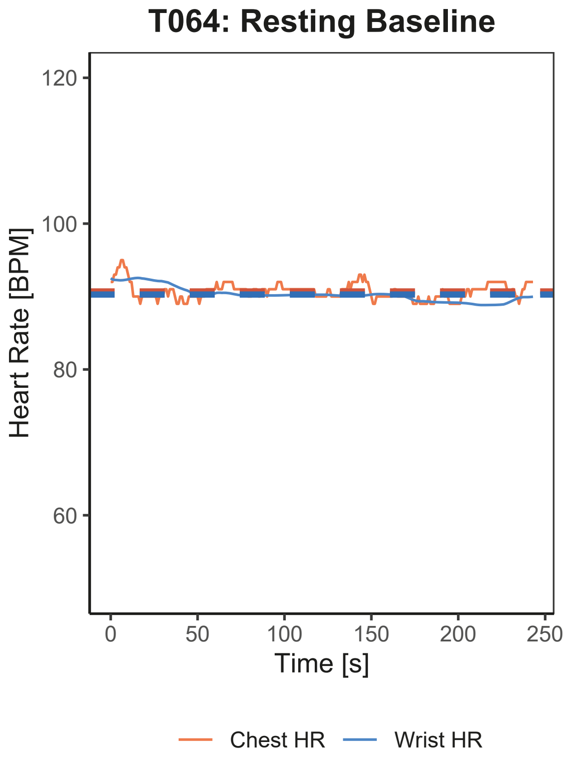
T064: Resting Baseline
Heart Rate [BPM]
120
100
80
60
0
50
100
150
200
250
Time [s]
Chest HR
Wrist HR

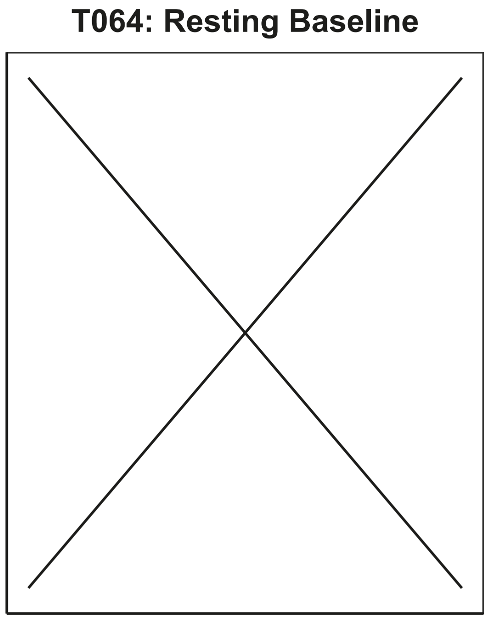
T064: Resting Baseline

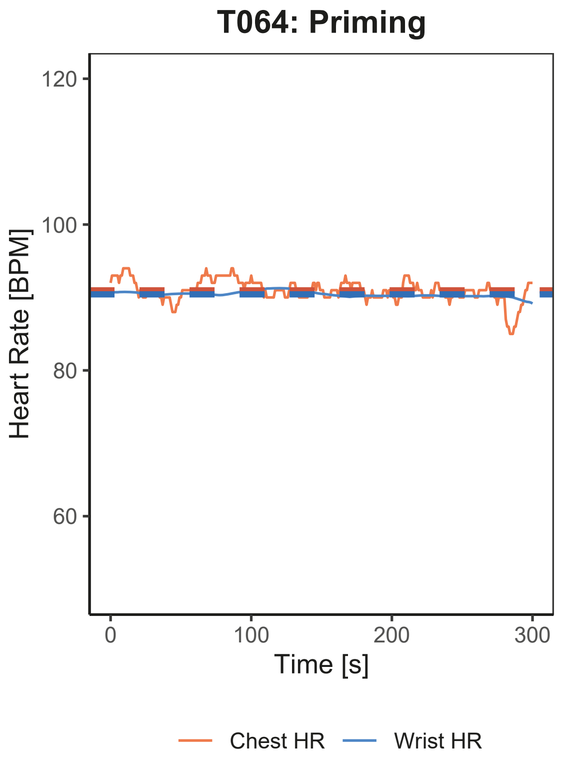
T064: Priming
Heart Rate [BPM]
120
100
80
60
0
100
200
300
Time [s]
Chest HR
Wrist HR

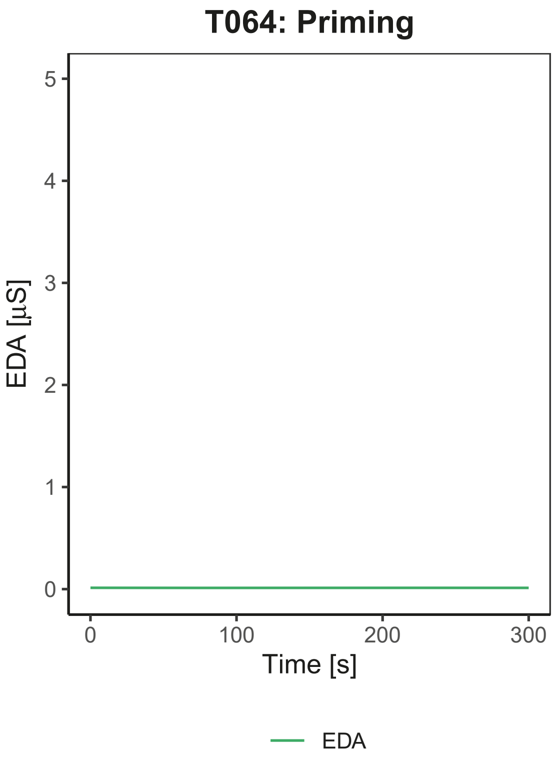
T064: Priming
EDA [µS]
5
4
3
2
1
0
0
100
200
300
Time [s]
EDA

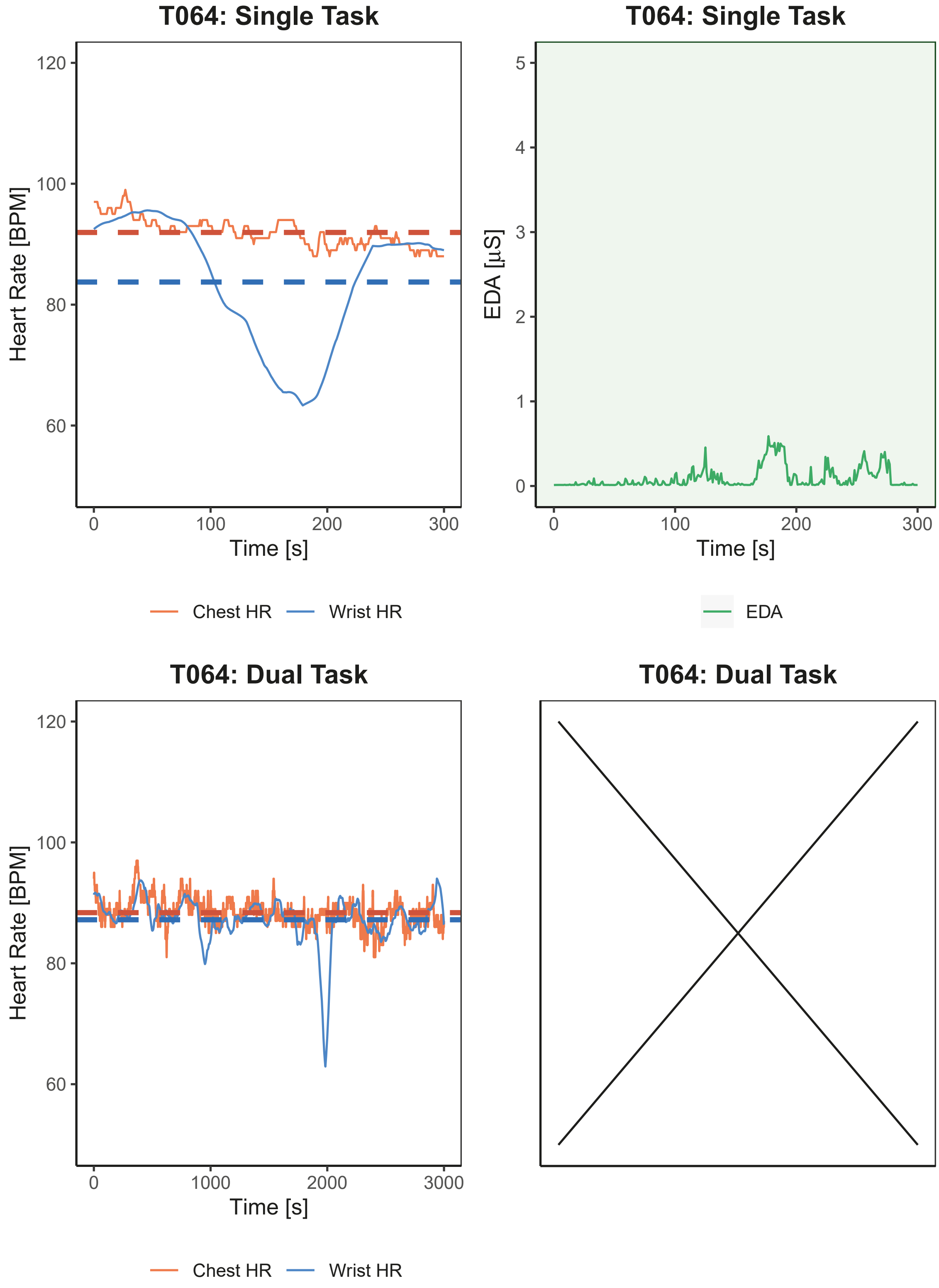
T064: Single Task
Heart Rate [BPM]
Time [s]
Chest HR
Wrist HR
T064: Single Task
EDA [µS]
Time [s]
EDA
T064: Dual Task
Heart Rate [BPM]
Time [s]
Chest HR
Wrist HR
T064: Dual Task

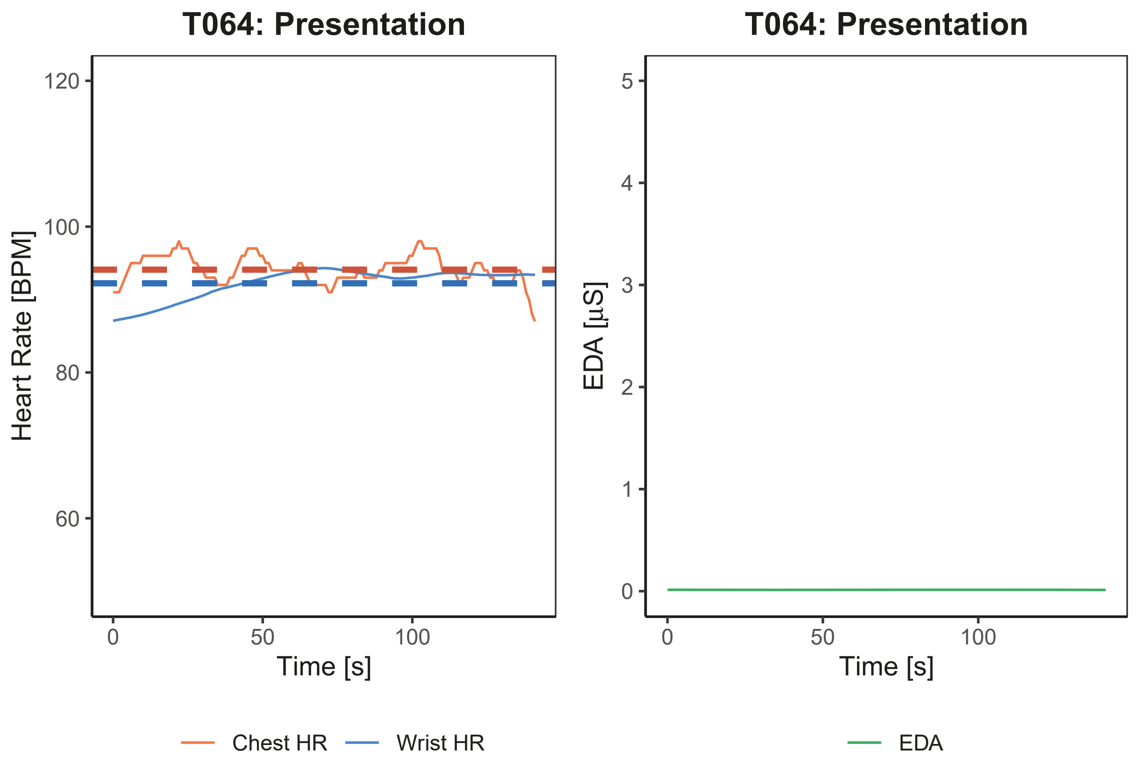
T064: Presentation
Heart Rate [BPM]
120
100
80
60
0
50
100
Time [s]
Chest HR
Wrist HR
T064: Presentation
EDA [µS]
5
4
3
2
1
0
0
50
100
Time [s]
EDA

--

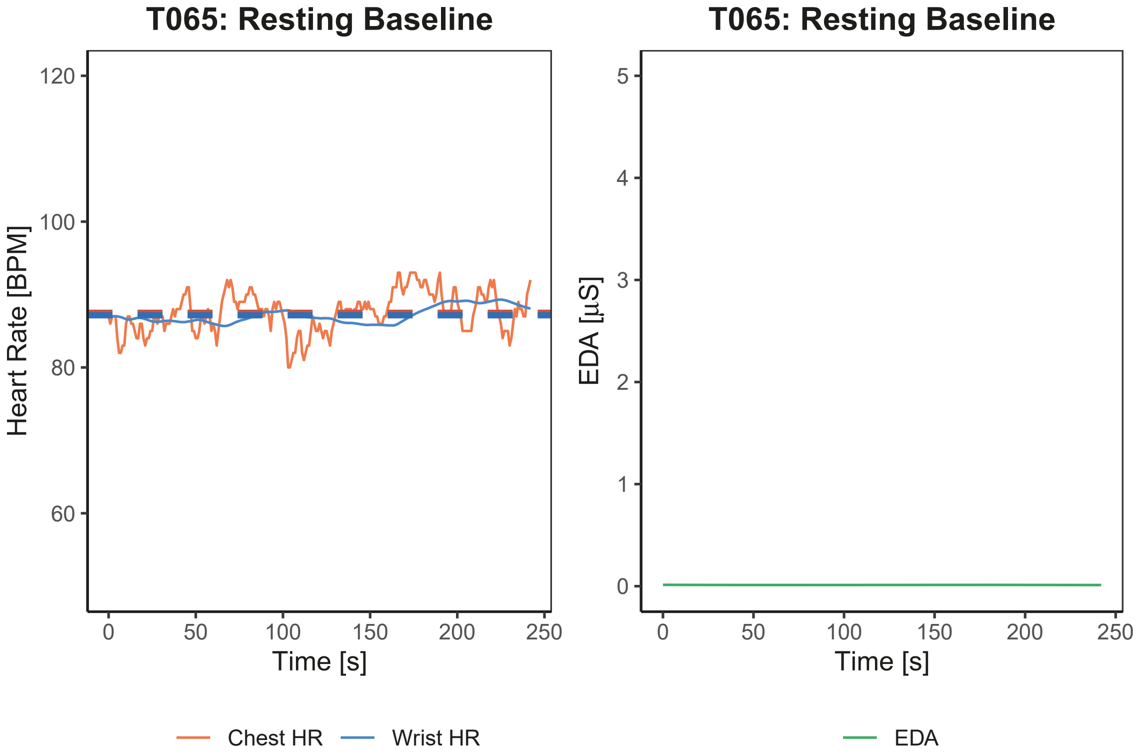
T065: Resting Baseline
Heart Rate [BPM]
Time [s]
Chest HR
Wrist HR
T065: Resting Baseline
EDA [µS]
Time [s]
EDA

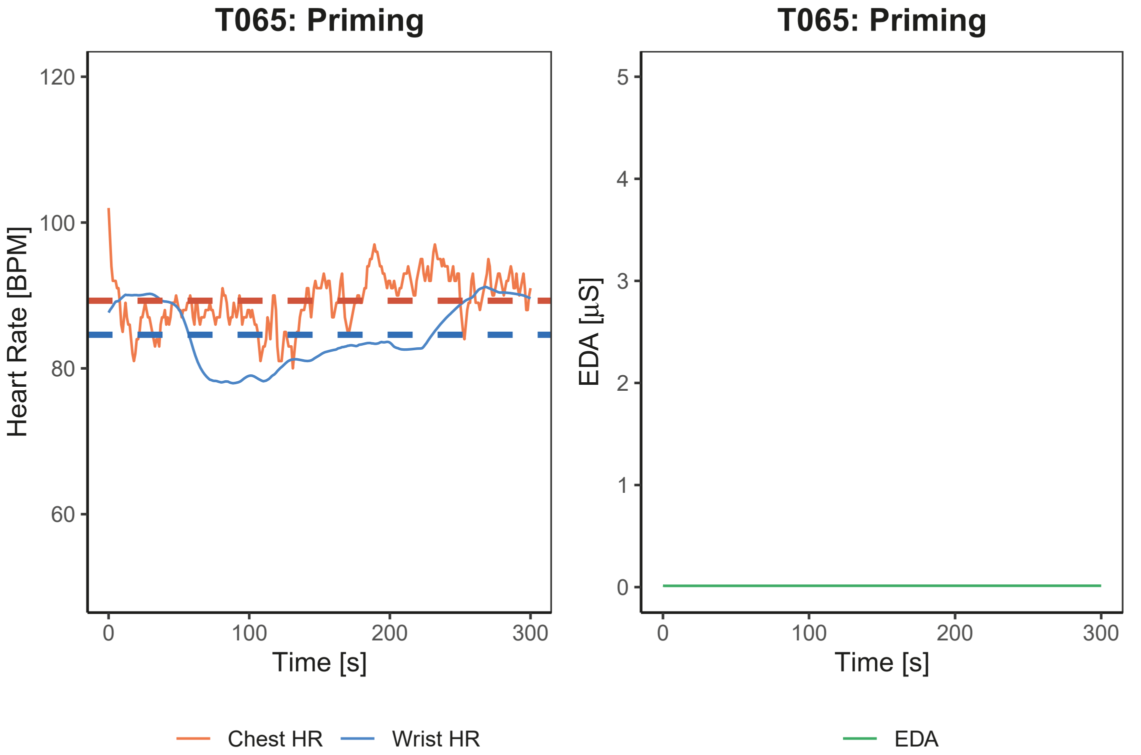
T065: Priming
Heart Rate [BPM]
Time [s]
Chest HR
Wrist HR
T065: Priming
EDA [µS]
Time [s]
EDA

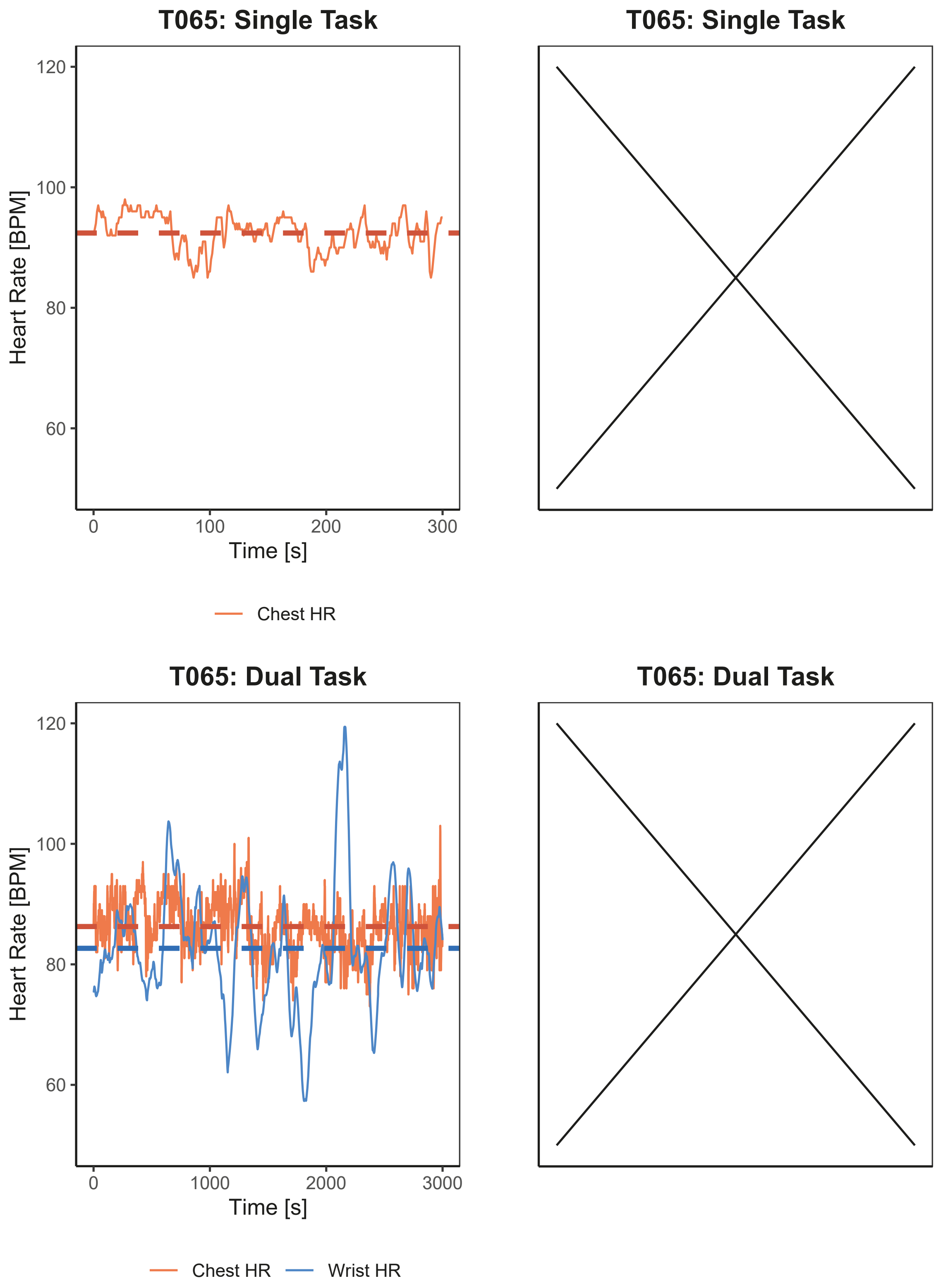
T065: Single Task
Heart Rate [BPM]
120
100
80
60
0
100
200
300
Time [s]
Chest HR
T065: Single Task
T065: Dual Task
Heart Rate [BPM]
120
100
80
60
0
1000
2000
3000
Time [s]
Chest HR
Wrist HR
T065: Dual Task

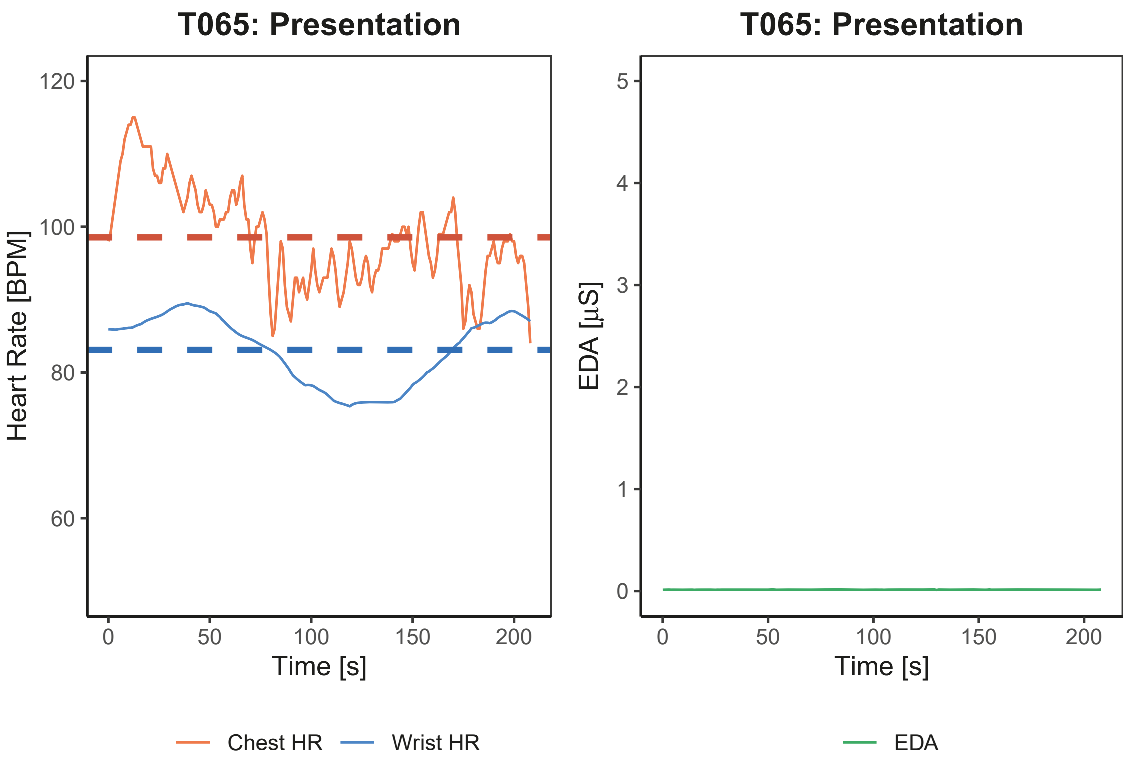

T065: Presentation
Heart Rate [BPM]
Time [s]
T065: Presentation
EDA [µS]
Time [s]
Chest HR
Wrist HR
EDA

--

T066: Resting Baseline

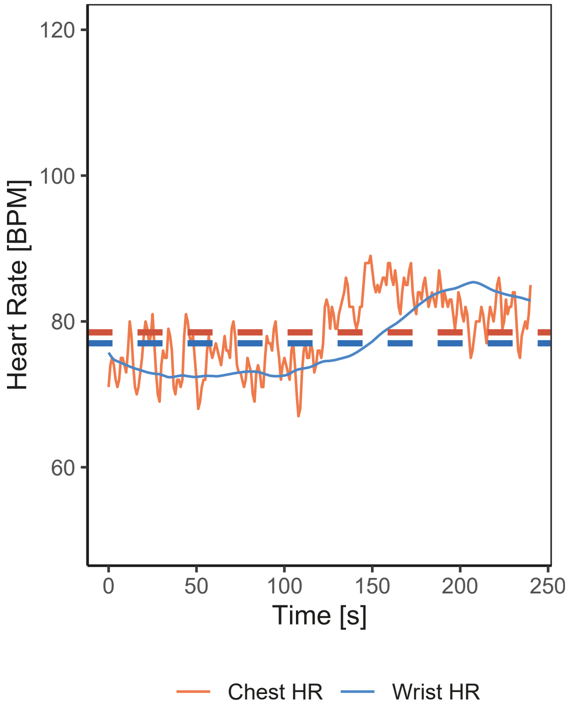

T066: Resting Baseline

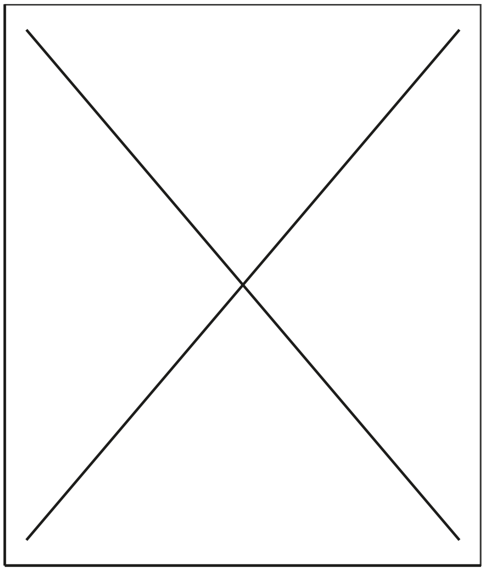

T066: Priming

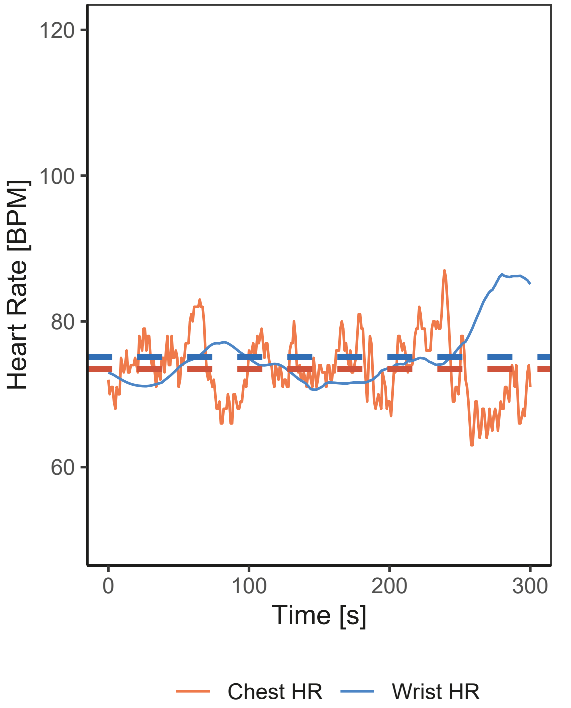

T066: Priming

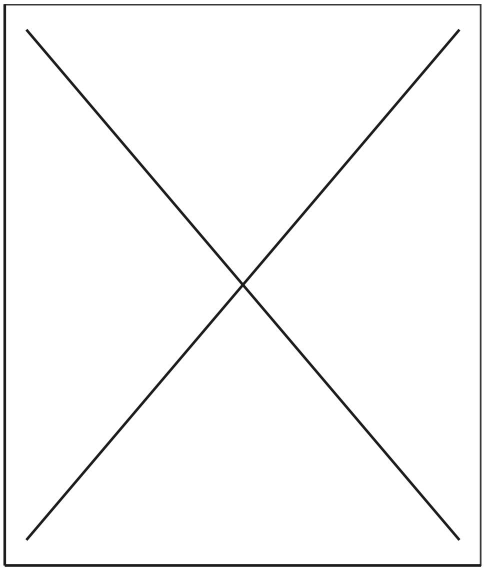

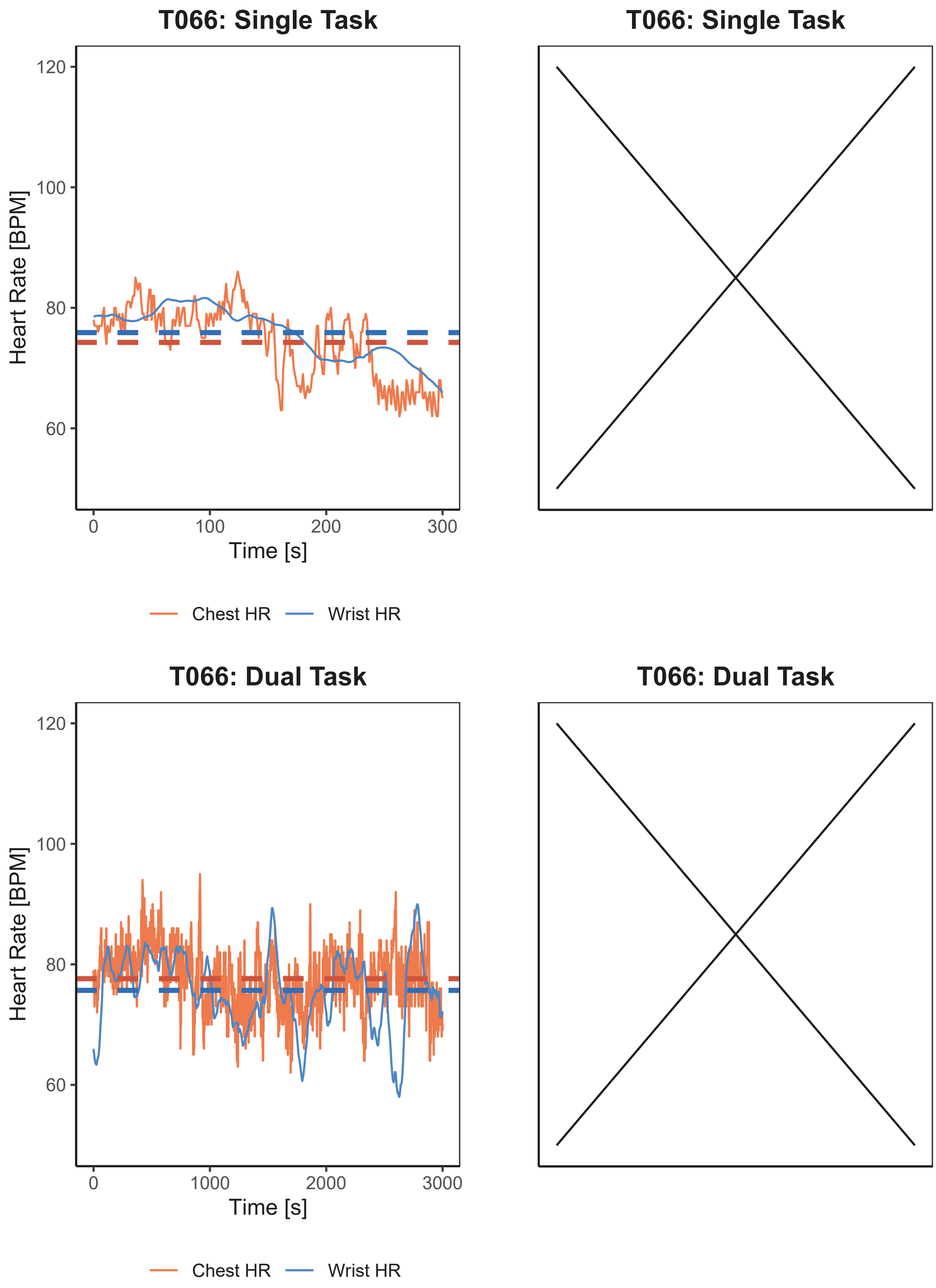
T066: Single Task
Heart Rate [BPM]
120
100
80
60
0
100
200
300
Time [s]
Chest HR
Wrist HR
T066: Single Task
T066: Dual Task
Heart Rate [BPM]
120
100
80
60
0
1000
2000
3000
Time [s]
Chest HR
Wrist HR
T066: Dual Task

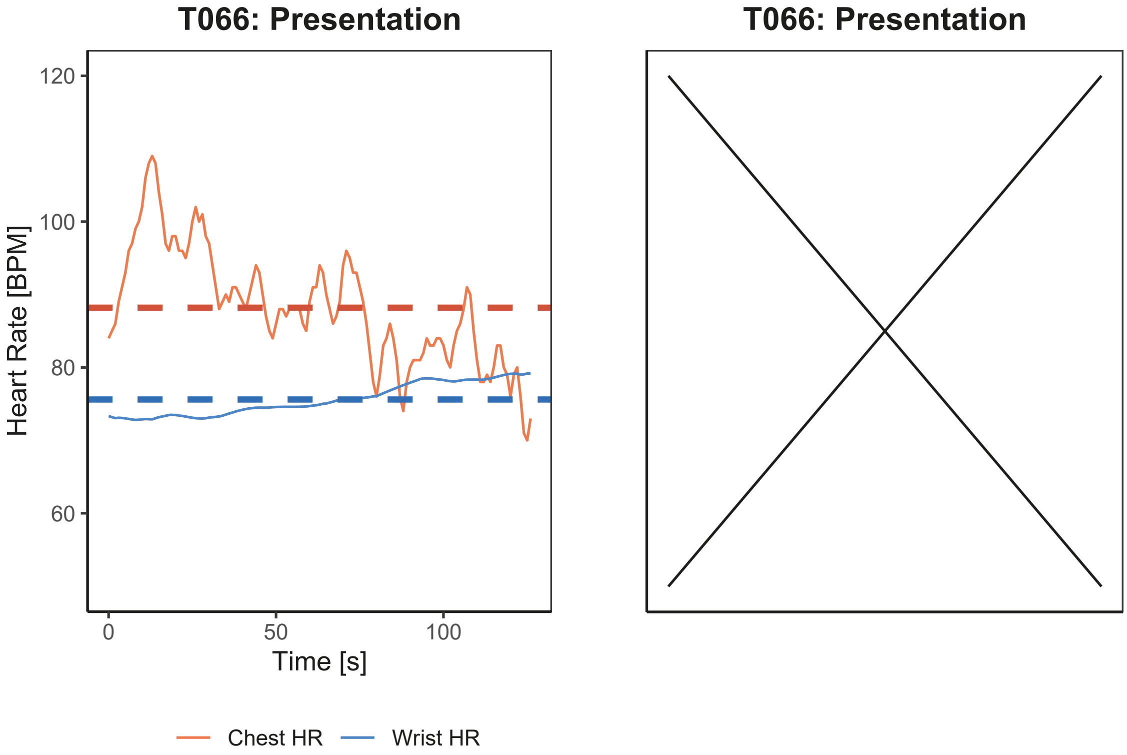

T066: Presentation
120
100
80
60
Heart Rate [BPM]
0
50
100
Time [s]
T066: Presentation
Chest HR
Wrist HR

--

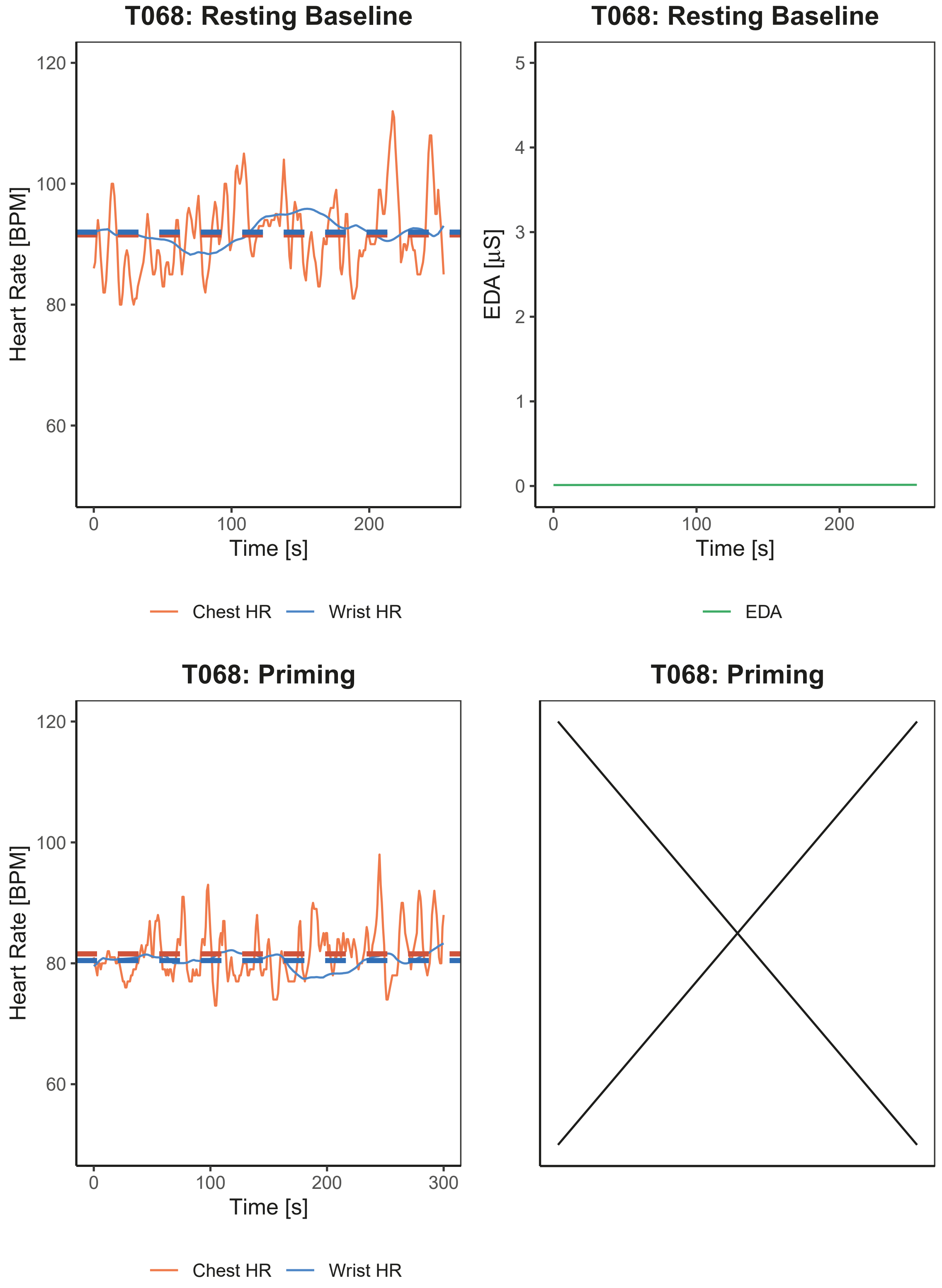
T068: Resting Baseline
Heart Rate [BPM]
120
100
80
60
0
100
200
Time [s]
Chest HR
Wrist HR
T068: Resting Baseline
EDA [µS]
5
4
3
2
1
0
0
100
200
Time [s]
EDA
T068: Priming
Heart Rate [BPM]
120
100
80
60
0
100
200
300
Time [s]
Chest HR
Wrist HR
T068: Priming

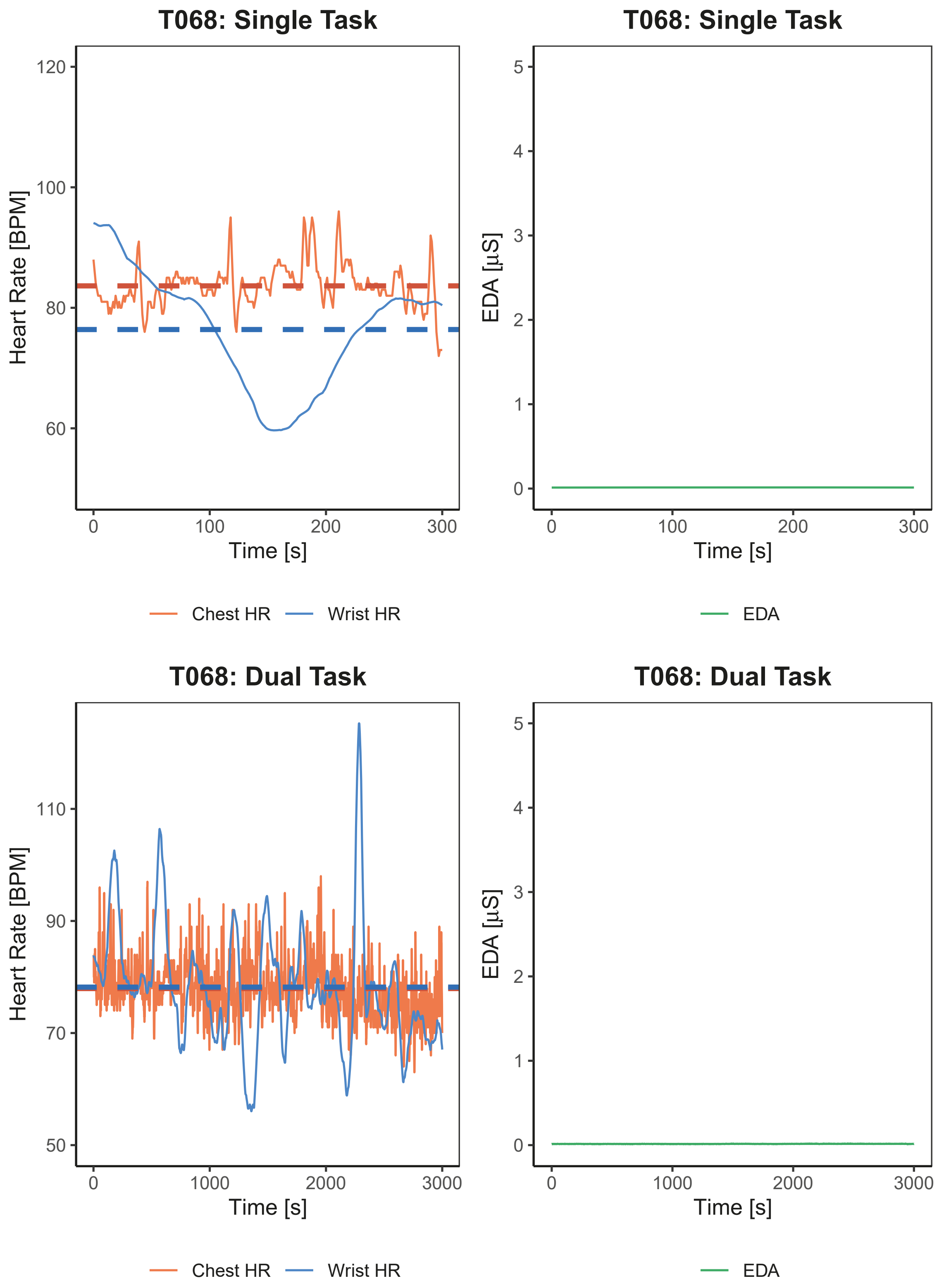
T068: Single Task
Heart Rate [BPM]
Time [s]
Chest HR
Wrist HR
T068: Single Task
EDA [µS]
Time [s]
EDA
T068: Dual Task
Heart Rate [BPM]
Time [s]
Chest HR
Wrist HR
T068: Dual Task
EDA [µS]
Time [s]
EDA

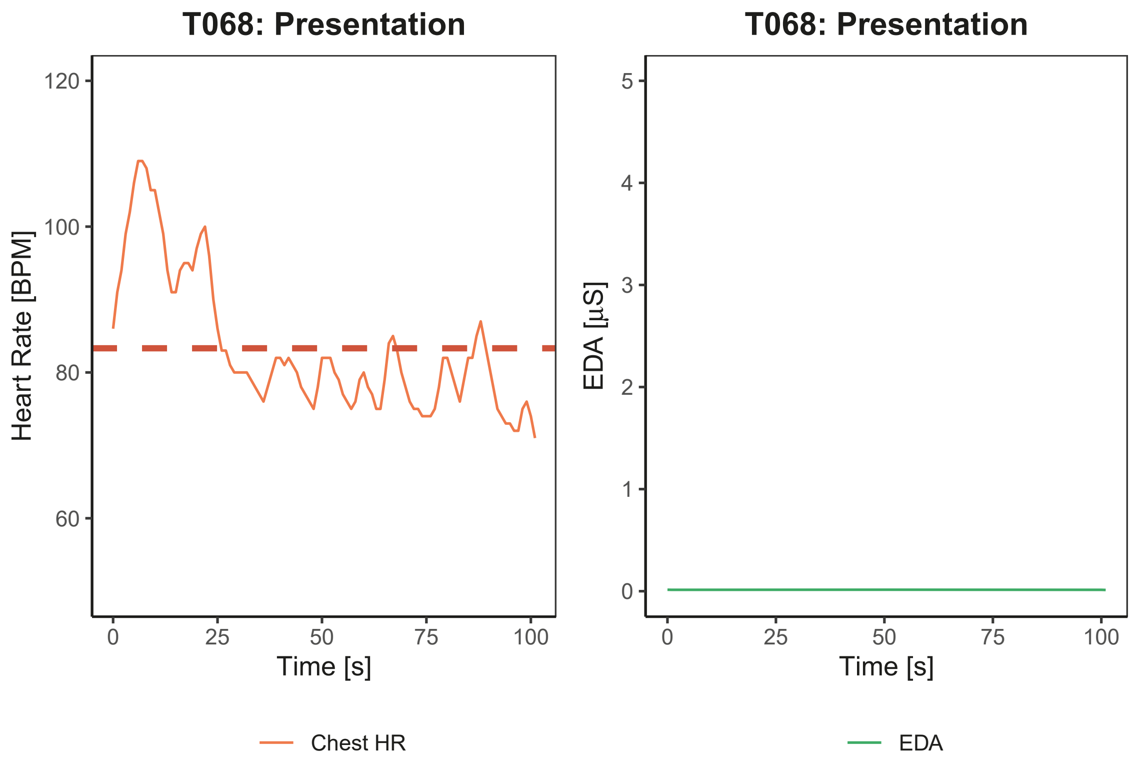
T068: Presentation
Heart Rate [BPM]
120
100
80
60
0
25
50
75
100
Time [s]
Chest HR
T068: Presentation
EDA [µS]
5
4
3
2
1
0
0
25
50
75
100
Time [s]
EDA

--

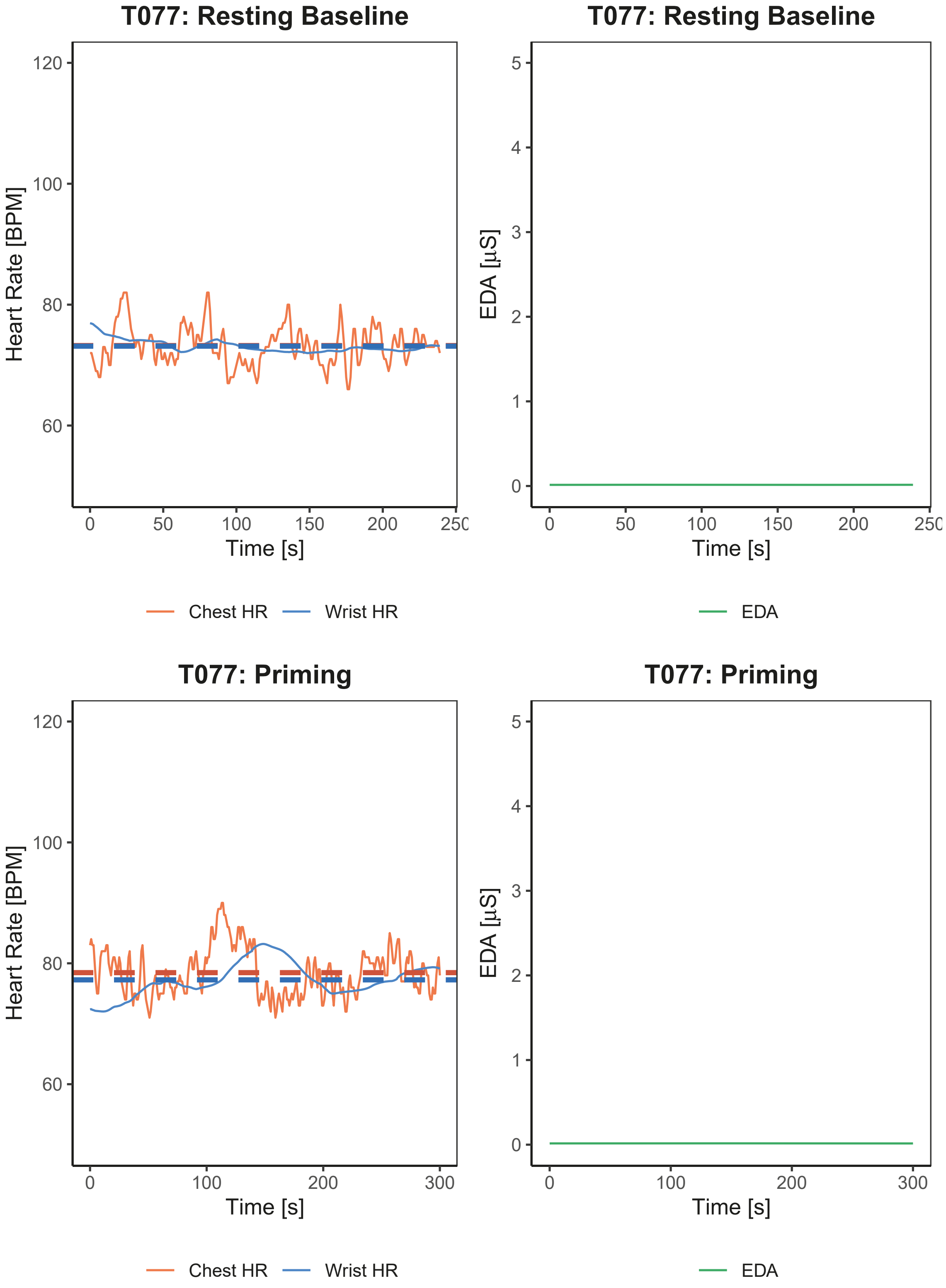
T077: Resting Baseline
Heart Rate [BPM]
120
100
80
60
0
50
100
150
200
250
Time [s]
Chest HR
Wrist HR
T077: Resting Baseline
EDA [µS]
5
4
3
2
1
0
0
50
100
150
200
250
Time [s]
EDA
T077: Priming
Heart Rate [BPM]
120
100
80
60
0
100
200
300
Time [s]
Chest HR
Wrist HR
T077: Priming
EDA [µS]
5
4
3
2
1
0
0
100
200
300
Time [s]
EDA

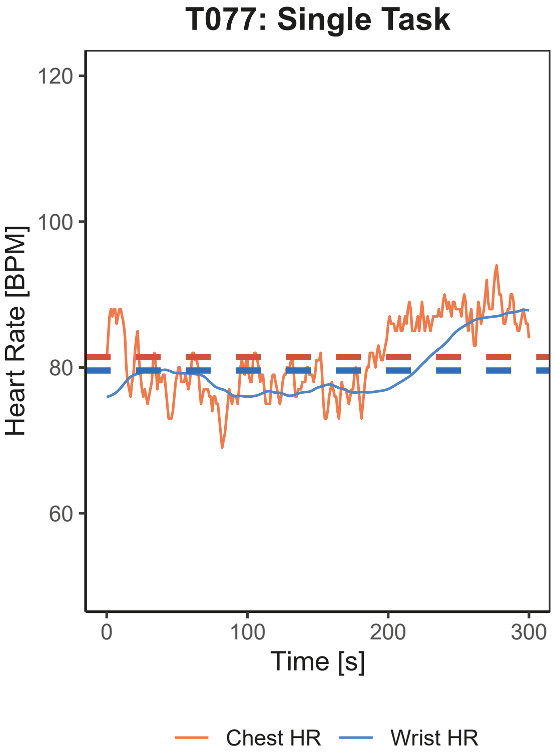
T077: Single Task
Heart Rate [BPM]
120
100
80
60
0
100
200
300
Time [s]
Chest HR
Wrist HR

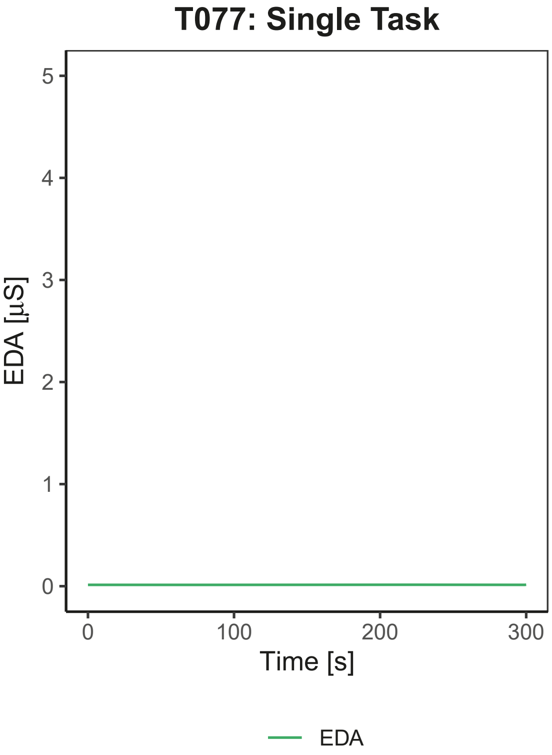
T077: Single Task
EDA [µS]
5
4
3
2
1
0
0
100
200
300
Time [s]
EDA

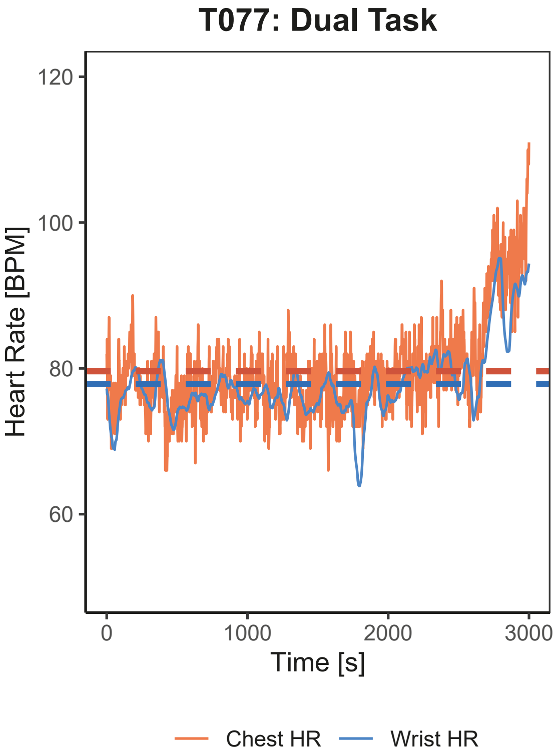
T077: Dual Task
Heart Rate [BPM]
120
100
80
60
0
1000
2000
3000
Time [s]
Chest HR
Wrist HR

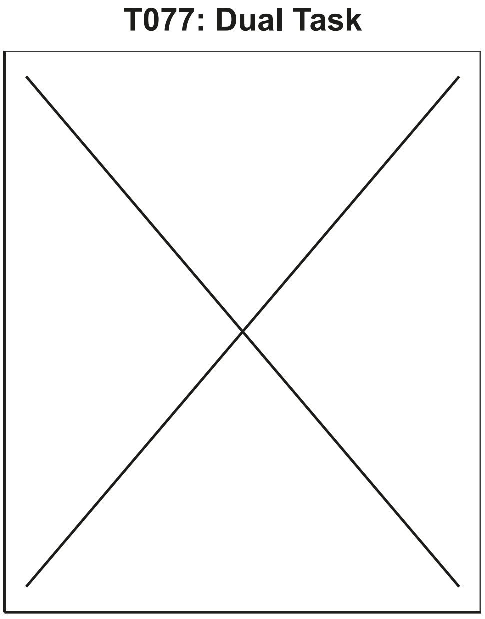
T077: Dual Task

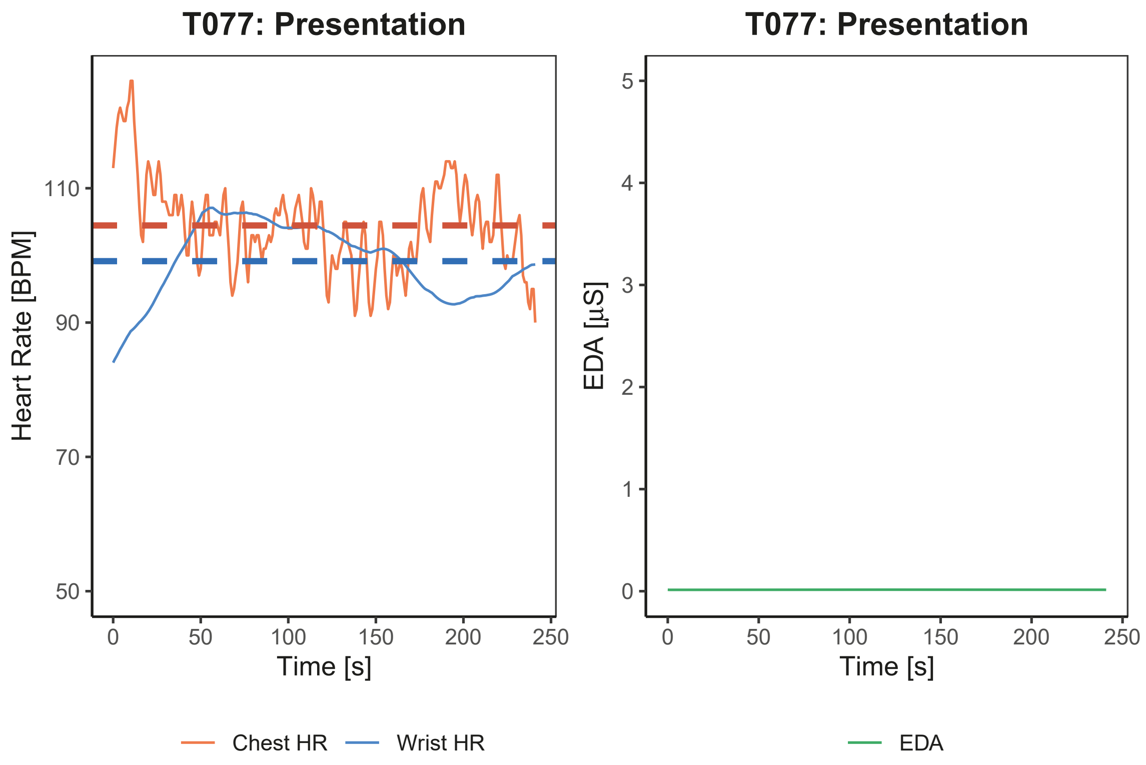
T077: Presentation
Heart Rate [BPM]
110
90
70
50
0
50
100
150
200
250
Time [s]
T077: Presentation
EDA [µS]
5
4
3
2
1
0
0
50
100
150
200
250
Time [s]
Chest HR
Wrist HR
EDA

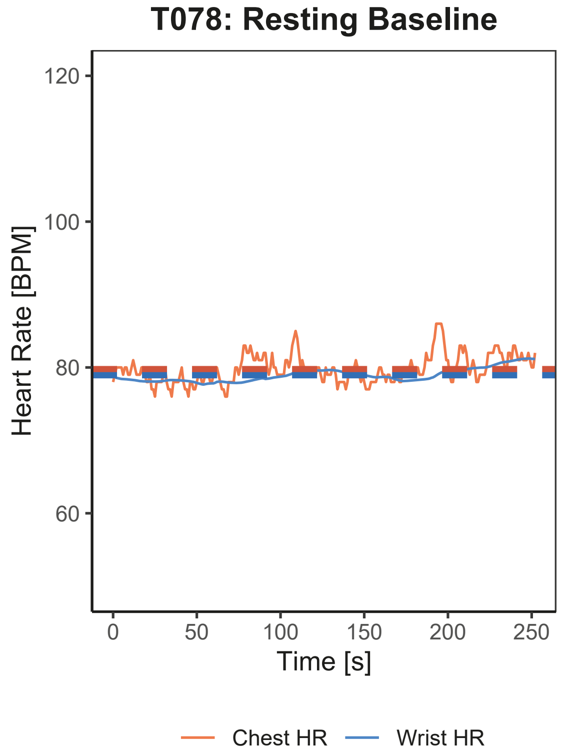
T078: Resting Baseline
Heart Rate [BPM]
120
100
80
60
0
50
100
150
200
250
Time [s]
Chest HR
Wrist HR

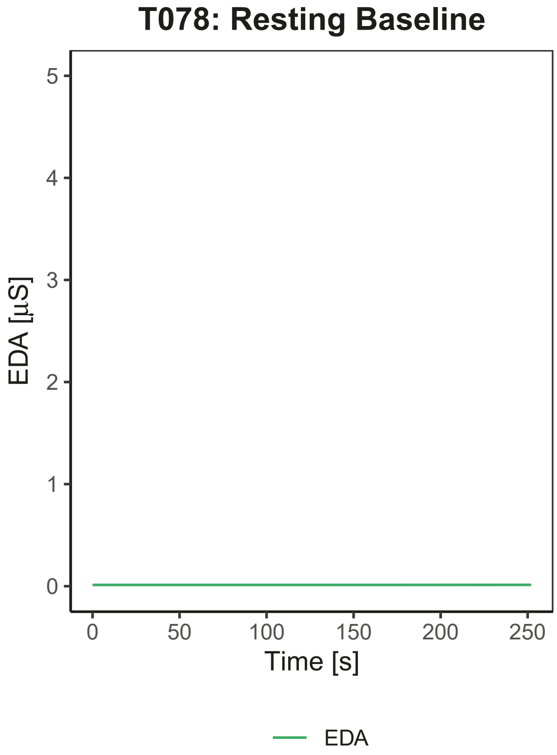
T078: Resting Baseline
EDA [μS]
5
4
3
2
1
0
0
50
100
150
200
250
Time [s]
EDA

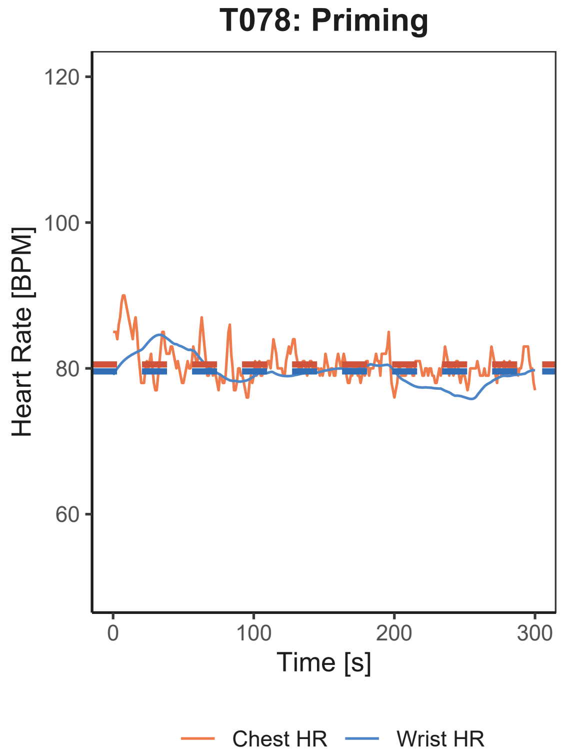
T078: Priming
Heart Rate [BPM]
120
100
80
60
0
100
200
300
Time [s]
Chest HR
Wrist HR

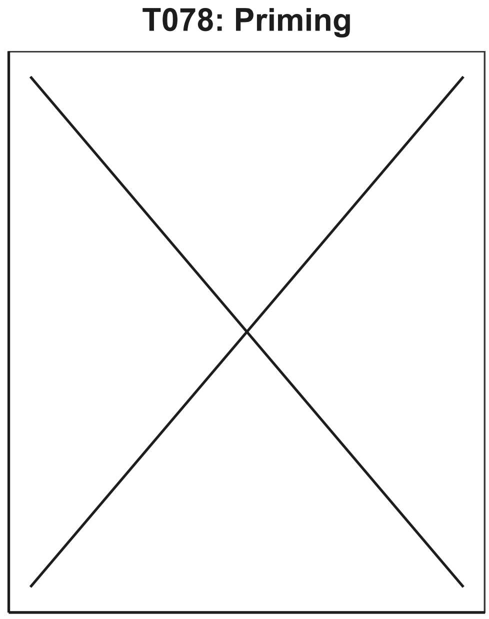
T078: Priming

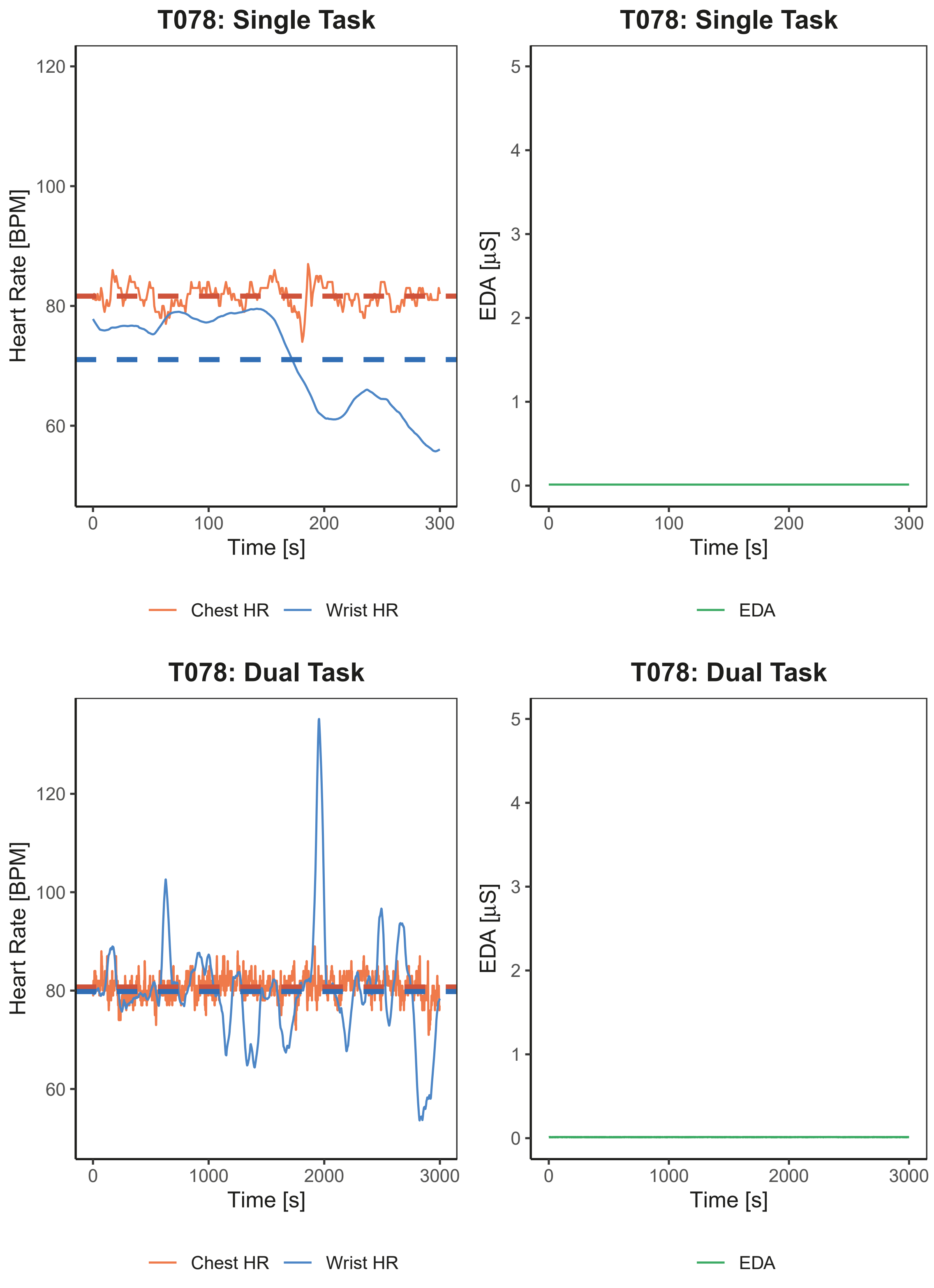
T078: Single Task
Heart Rate [BPM]
Time [s]
Chest HR
Wrist HR
T078: Single Task
EDA [µS]
Time [s]
EDA
T078: Dual Task
Heart Rate [BPM]
Time [s]
Chest HR
Wrist HR
T078: Dual Task
EDA [µS]
Time [s]
EDA

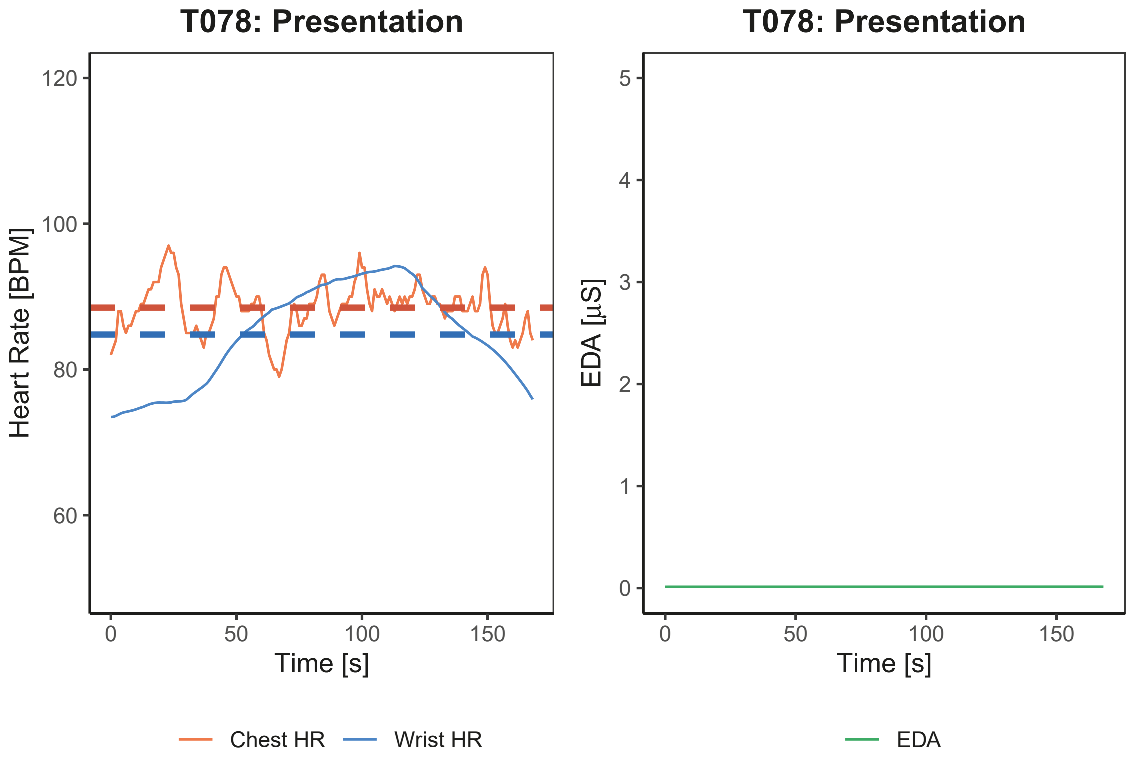
T078: Presentation
Heart Rate [BPM]
120
100
80
60
0
50
100
150
Time [s]
Chest HR
Wrist HR
T078: Presentation
EDA [µS]
5
4
3
2
1
0
0
50
100
150
Time [s]
EDA

--

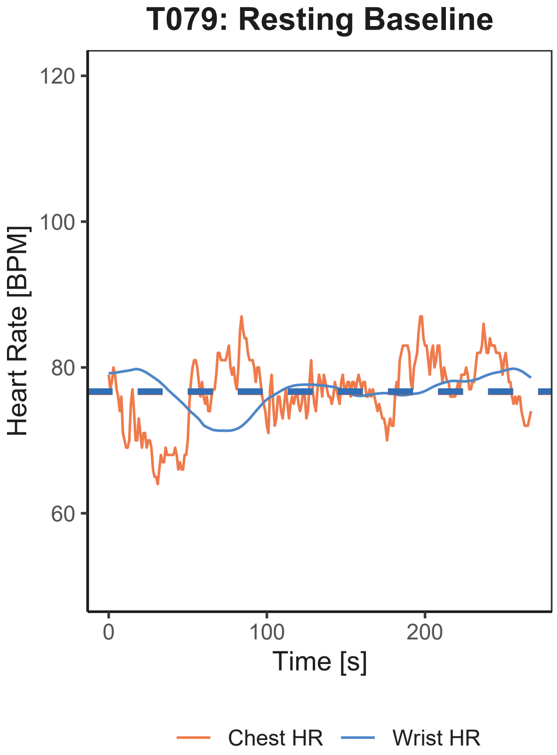
T079: Resting Baseline
Heart Rate [BPM]
120
100
80
60
0
100
200
Time [s]
Chest HR
Wrist HR

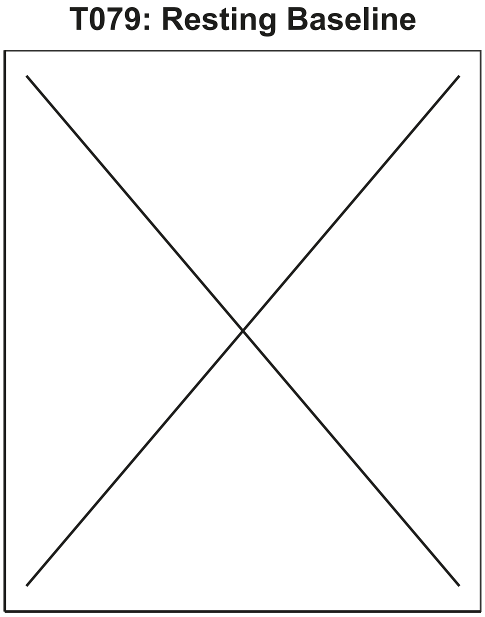
T079: Resting Baseline

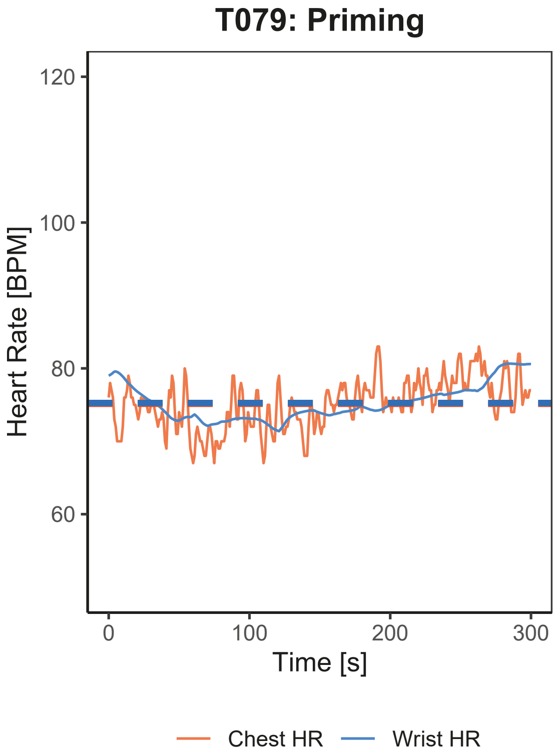
T079: Priming
Heart Rate [BPM]
120
100
80
60
0
100
200
300
Time [s]
Chest HR
Wrist HR

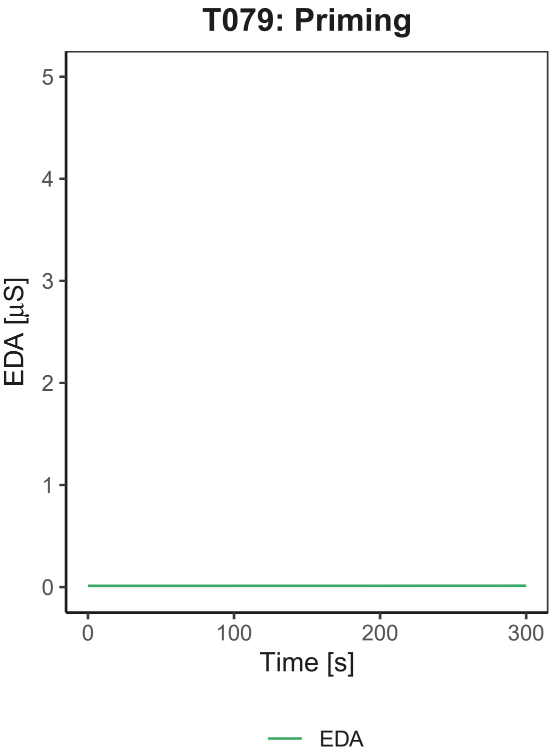
T079: Priming
EDA [µS]
5
4
3
2
1
0
0
100
200
300
Time [s]
EDA

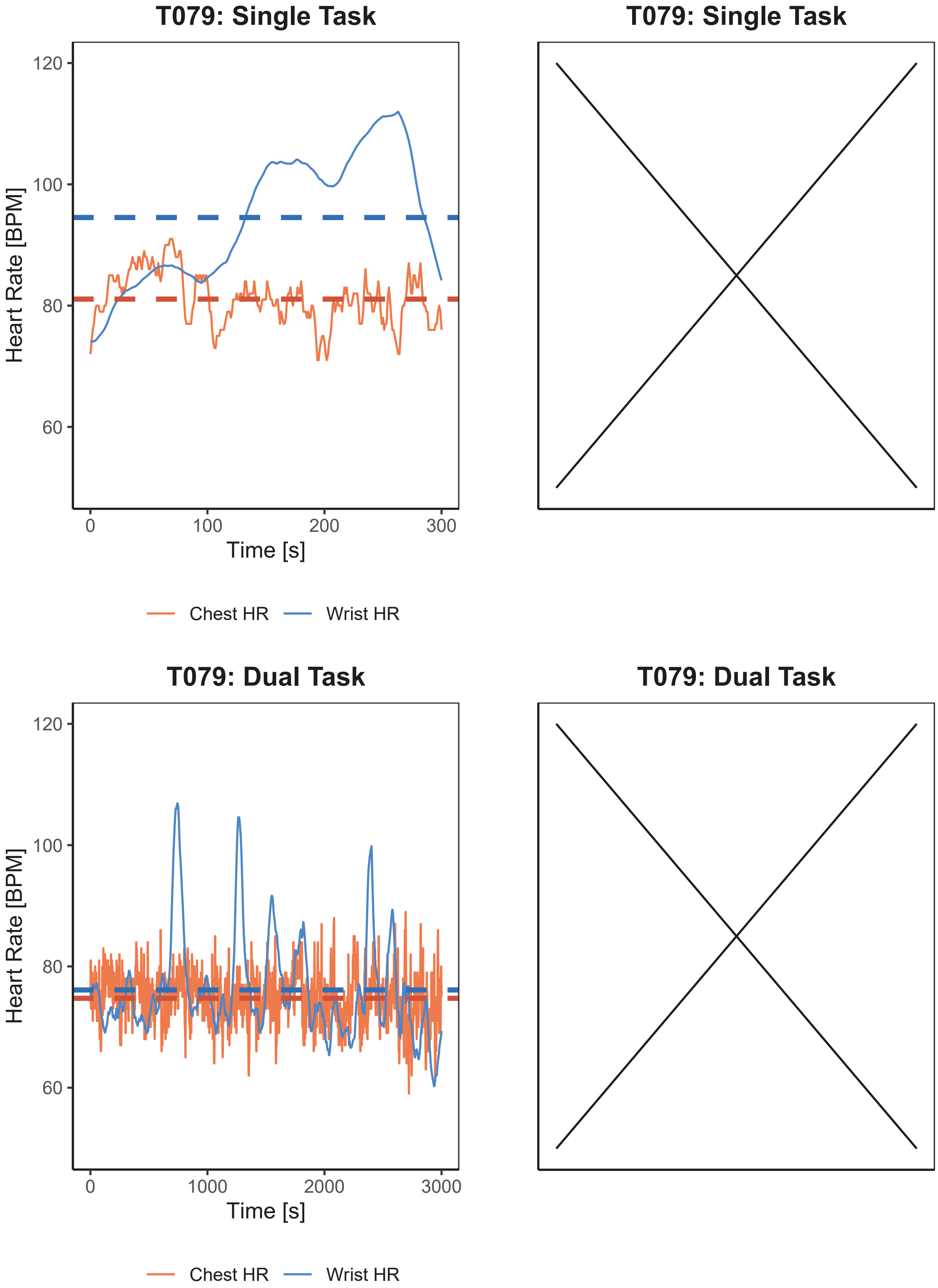
T079: Single Task
T079: Single Task
Heart Rate [BPM]
120
100
80
60
0
100
200
300
Time [s]
Chest HR
Wrist HR
T079: Dual Task
T079: Dual Task
Heart Rate [BPM]
120
100
80
60
0
1000
2000
3000
Time [s]
Chest HR
Wrist HR

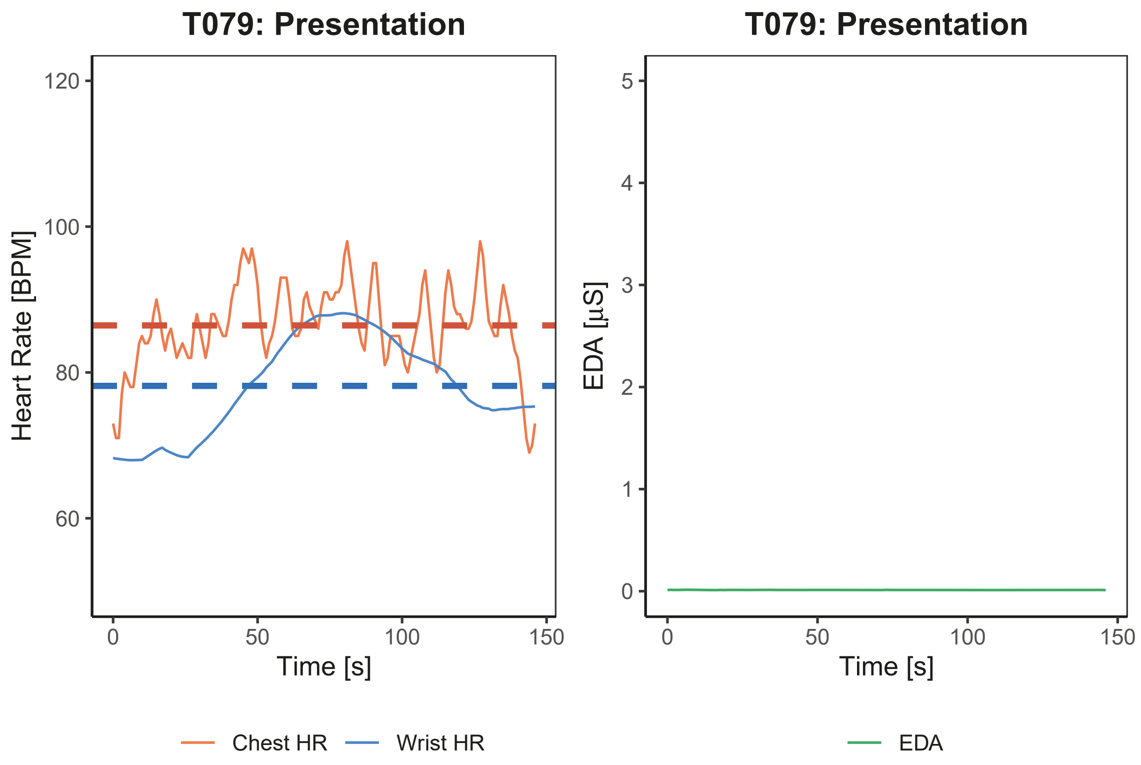
T079: Presentation
Heart Rate [BPM]
120
100
80
60
0
50
100
150
Time [s]
Chest HR
Wrist HR
T079: Presentation
EDA [µS]
5
4
3
2
1
0
0
50
100
150
Time [s]
EDA

--

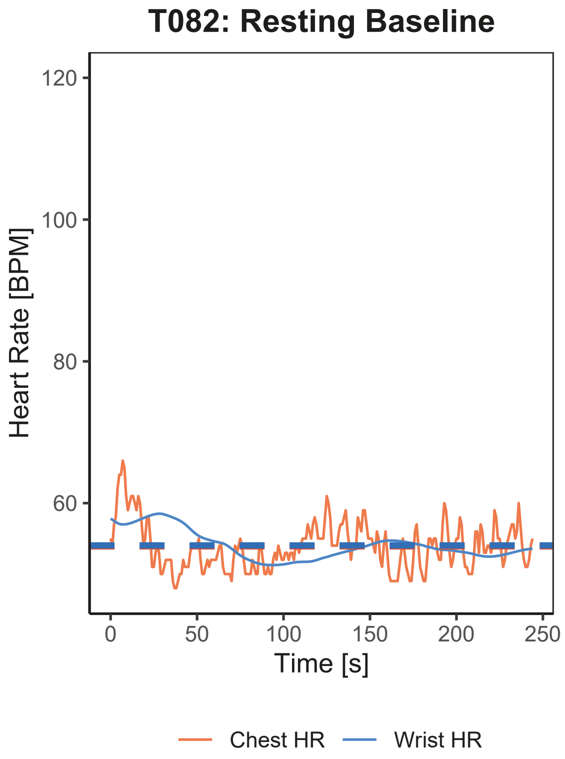
T082: Resting Baseline
Heart Rate [BPM]
120
100
80
60
0
50
100
150
200
250
Time [s]
Chest HR
Wrist HR

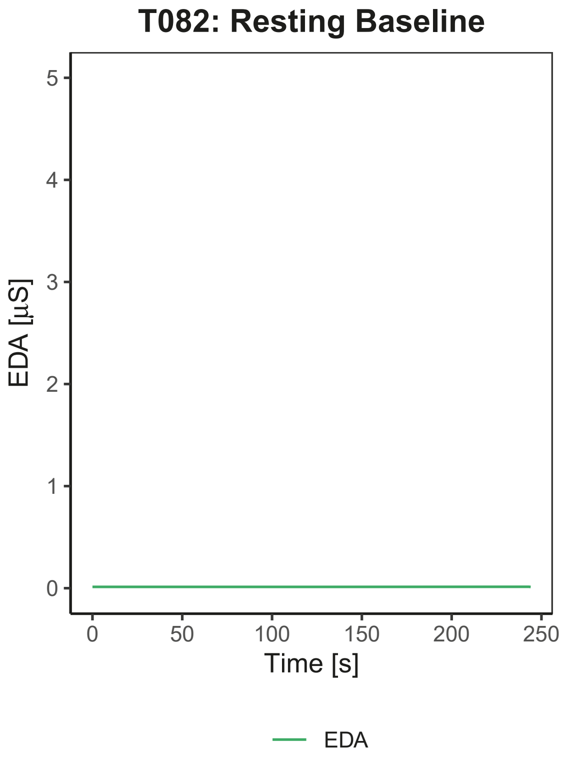
T082: Resting Baseline
EDA [µS]
5
4
3
2
1
0
0
50
100
150
200
250
Time [s]
EDA

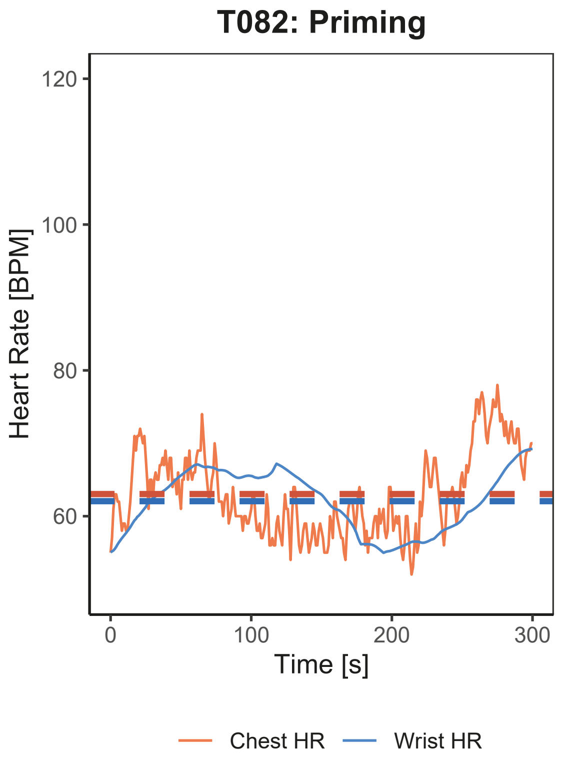
T082: Priming
Heart Rate [BPM]
120
100
80
60
0
100
200
300
Time [s]
Chest HR
Wrist HR

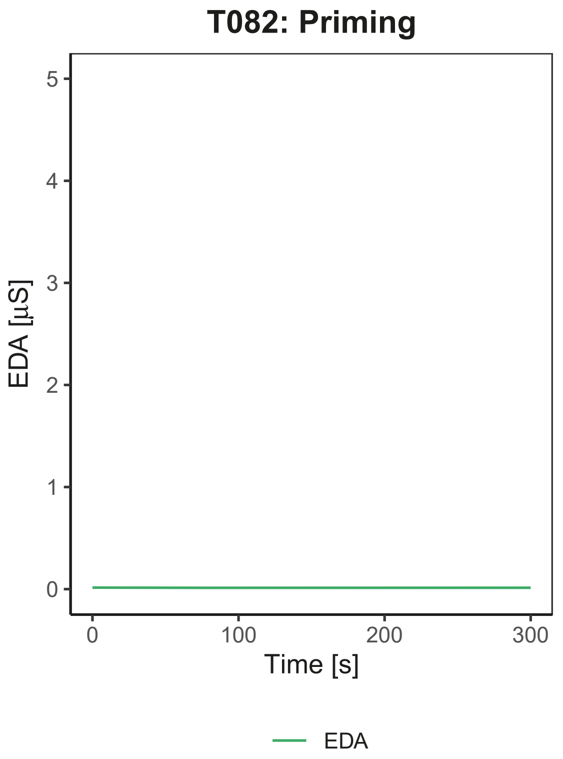
T082: Priming
EDA [µS]
5
4
3
2
1
0
0
100
200
300
Time [s]
EDA

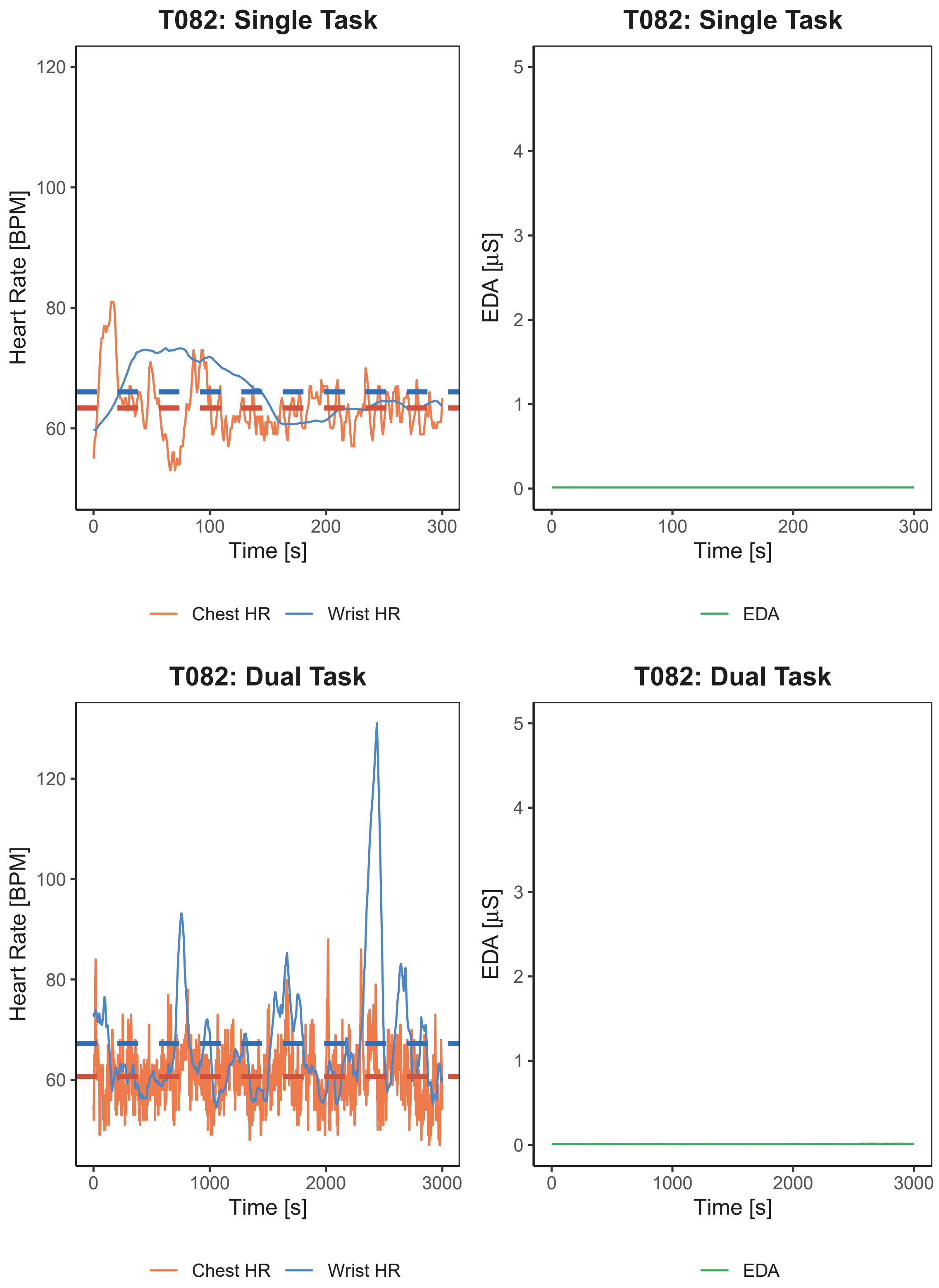
T082: Single Task
Heart Rate [BPM]
120
100
80
60
0
100
200
300
Time [s]
Chest HR
Wrist HR
T082: Single Task
EDA [µS]
5
4
3
2
1
0
0
100
200
300
Time [s]
EDA
T082: Dual Task
Heart Rate [BPM]
120
100
80
60
0
1000
2000
3000
Time [s]
Chest HR
Wrist HR
T082: Dual Task
EDA [µS]
5
4
3
2
1
0
0
1000
2000
3000
Time [s]
EDA

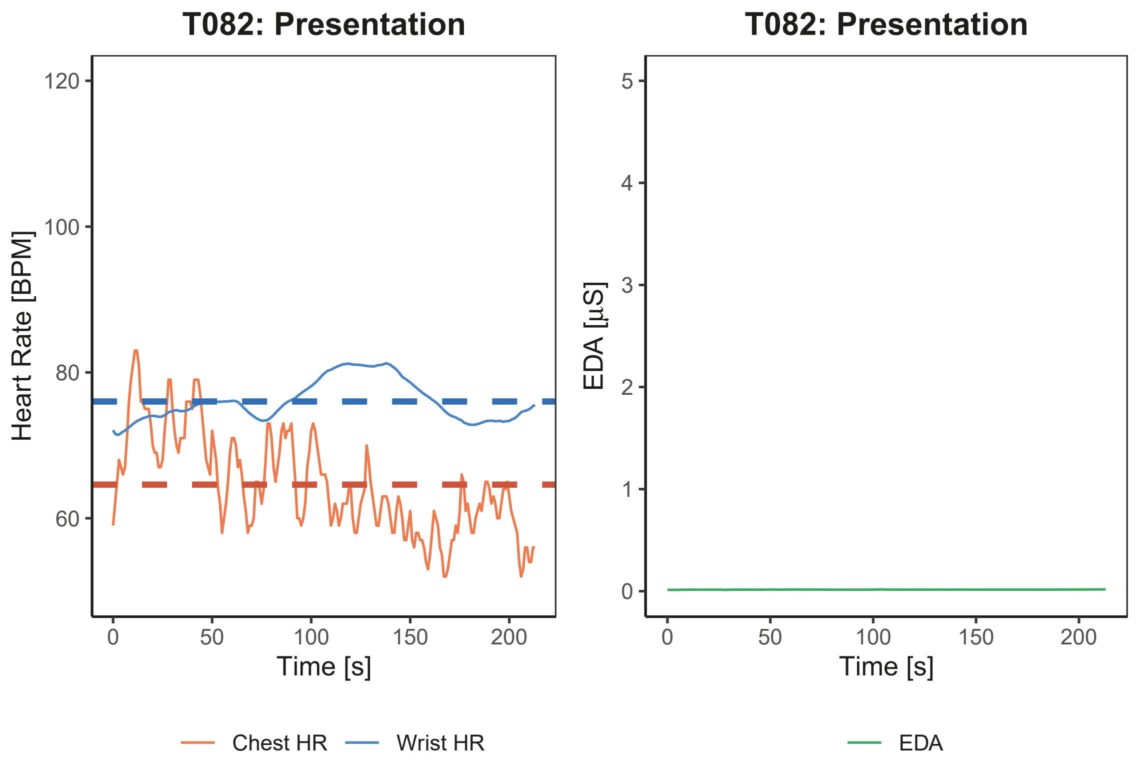
T082: Presentation
Heart Rate [BPM]
120
100
80
60
0
50
100
150
200
Time [s]
T082: Presentation
EDA [µS]
5
4
3
2
1
0
0
50
100
150
200
Time [s]
Chest HR
Wrist HR
EDA

--

T083: Resting Baseline

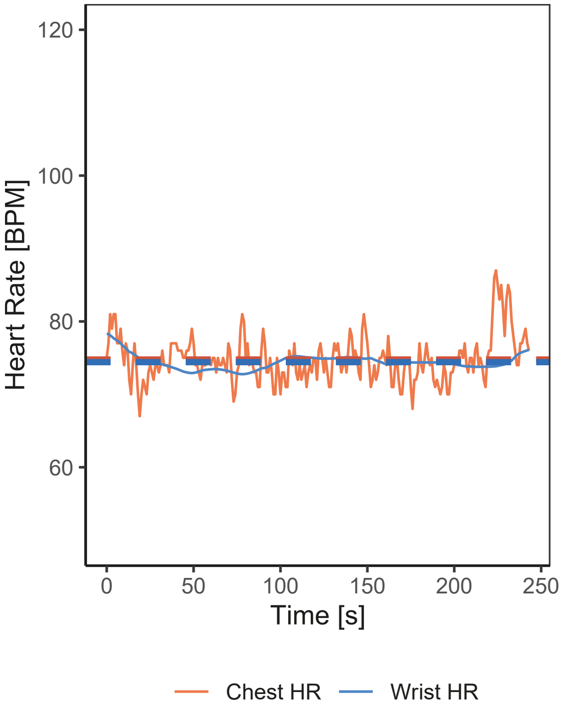

T083: Resting Baseline

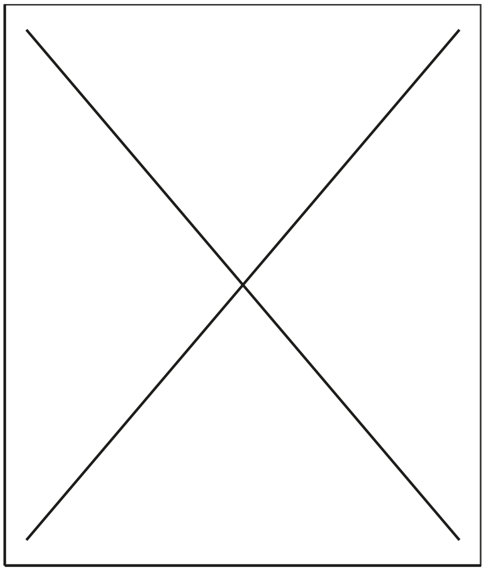

T083: Priming

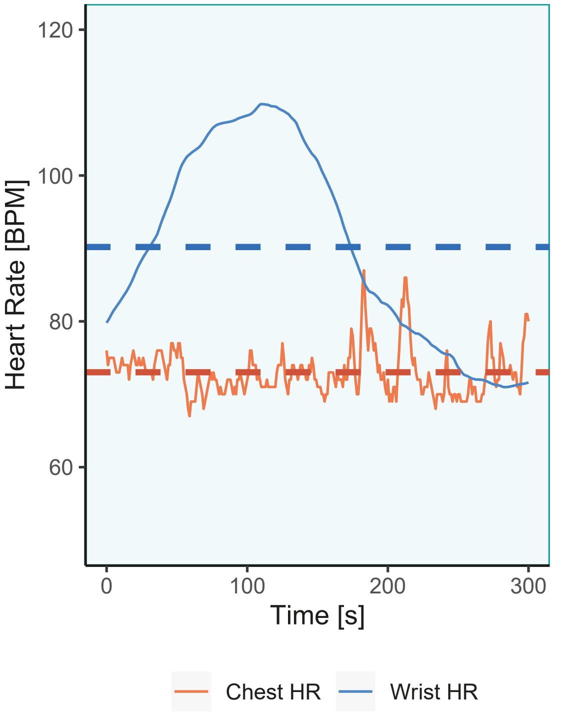

T083: Priming

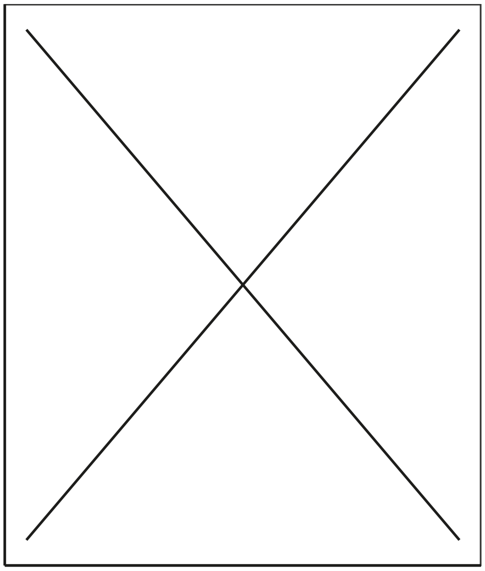

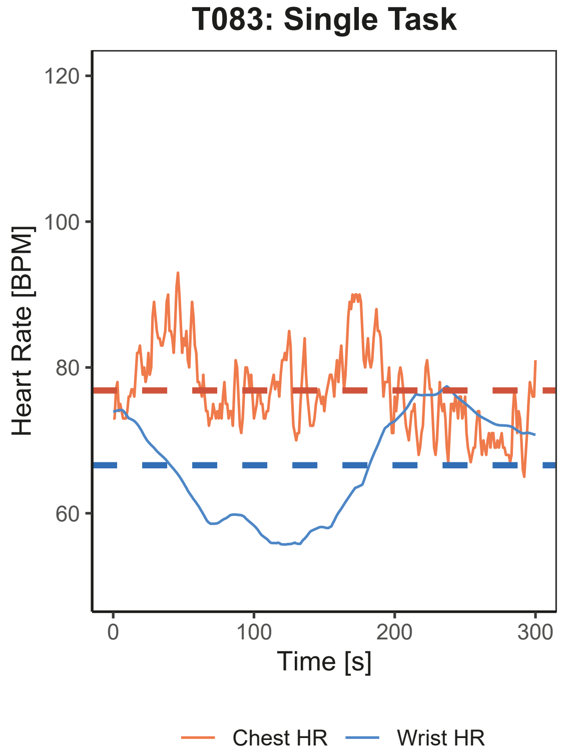
T083: Single Task
Heart Rate [BPM]
120
100
80
60
0
100
200
300
Time [s]
Chest HR
Wrist HR

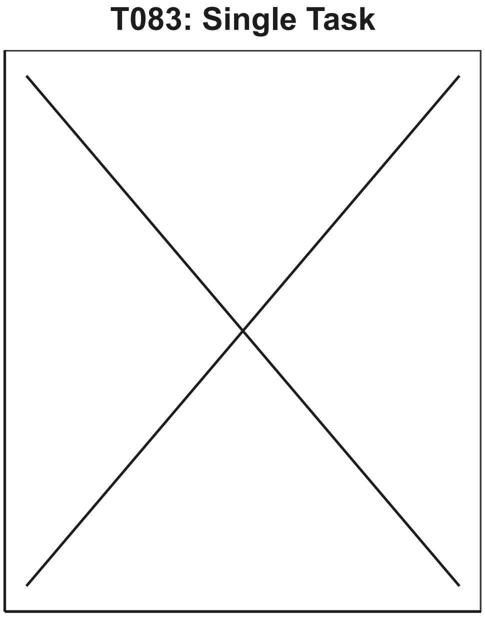
T083: Single Task

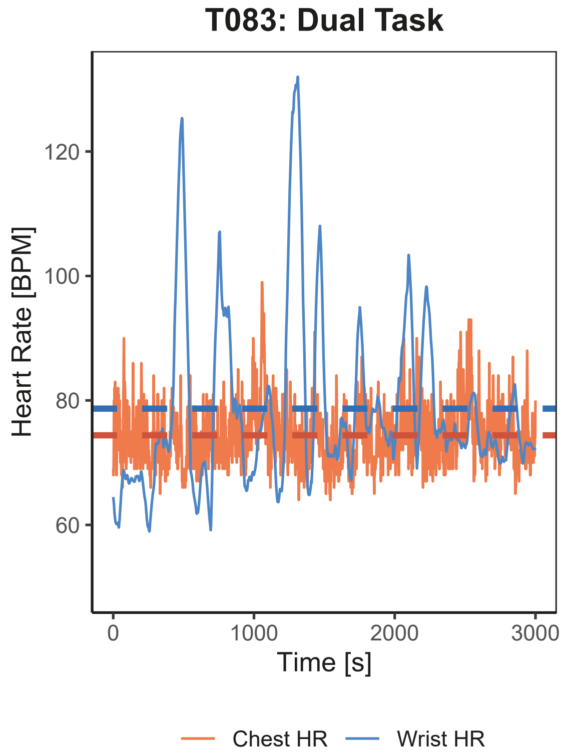
T083: Dual Task
Heart Rate [BPM]
120
100
80
60
0
1000
2000
3000
Time [s]
Chest HR
Wrist HR

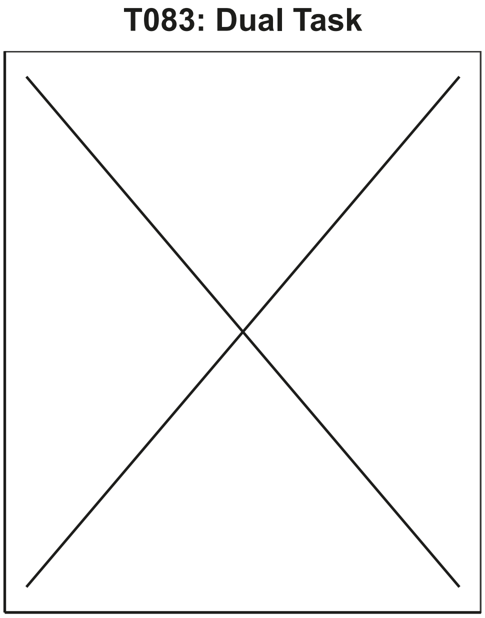
T083: Dual Task

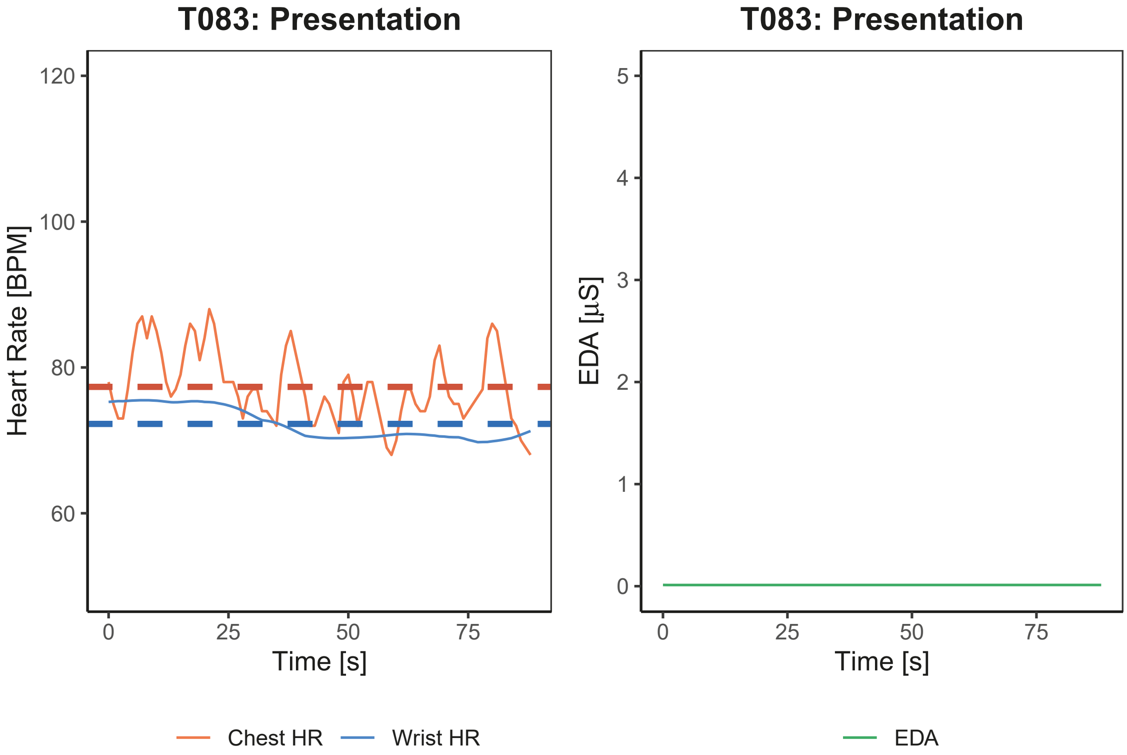
T083: Presentation
Heart Rate [BPM]
Time [s]
T083: Presentation
EDA [µS]
Time [s]
Chest HR
Wrist HR
EDA

--

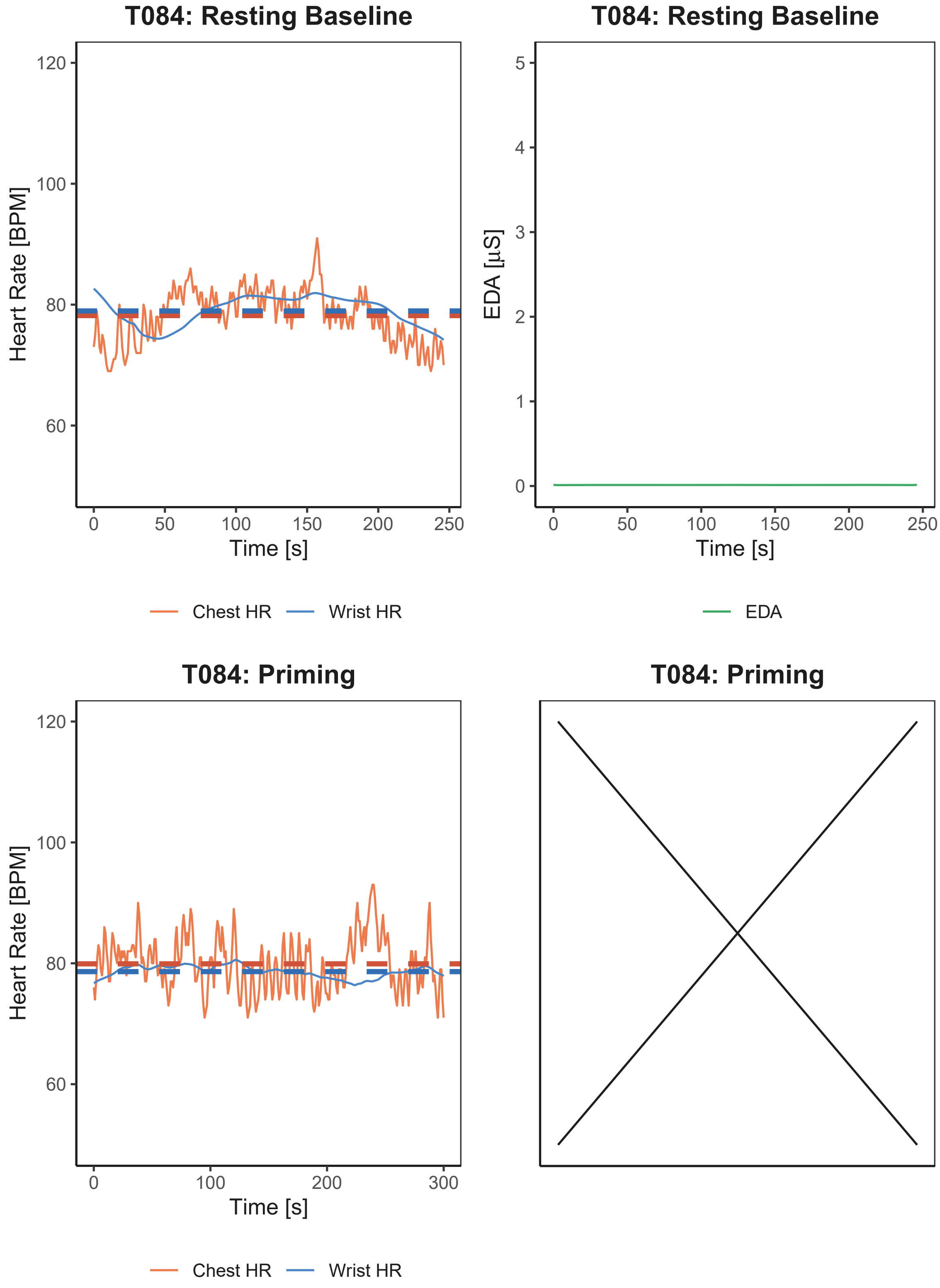
T084: Resting Baseline
Heart Rate [BPM]
120
100
80
60
0
50
100
150
200
250
Time [s]
Chest HR
Wrist HR
T084: Resting Baseline
EDA [µS]
5
4
3
2
1
0
0
50
100
150
200
250
Time [s]
EDA
T084: Priming
Heart Rate [BPM]
120
100
80
60
0
100
200
300
Time [s]
Chest HR
Wrist HR
T084: Priming

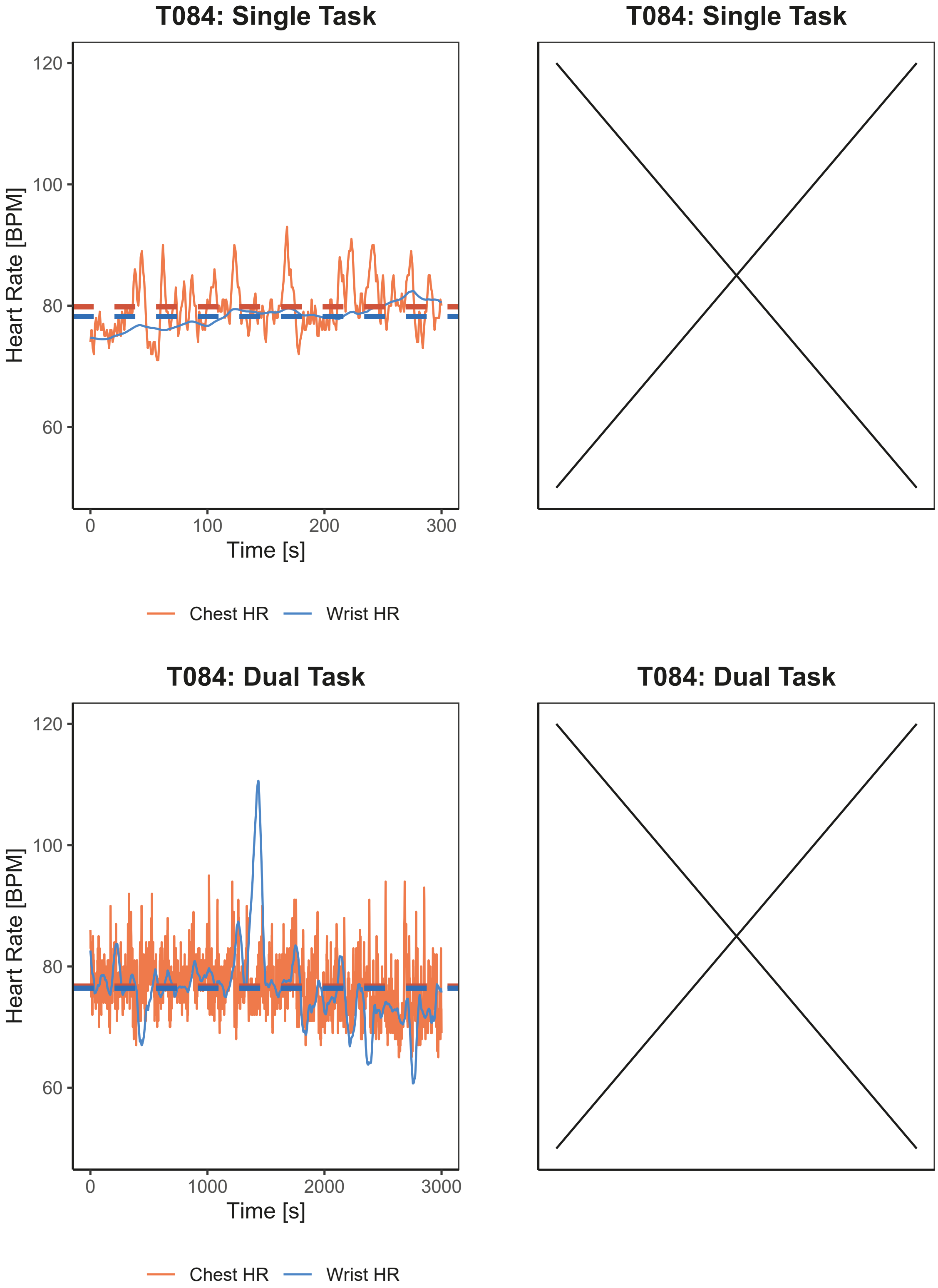
T084: Single Task
T084: Single Task
Heart Rate [BPM]
120
100
80
60
0
100
200
300
Time [s]
Chest HR
Wrist HR
T084: Dual Task
T084: Dual Task
Heart Rate [BPM]
120
100
80
60
0
1000
2000
3000
Time [s]
Chest HR
Wrist HR

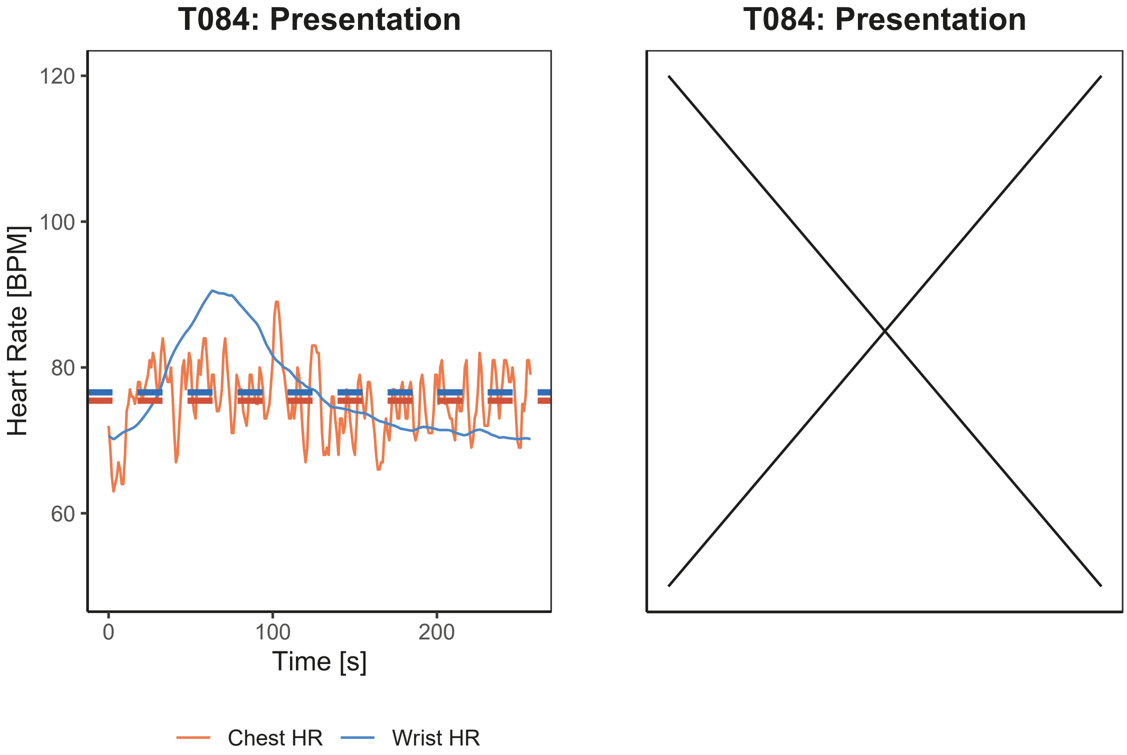
T084: Presentation
T084: Presentation
Heart Rate [BPM]
120
100
80
60
0
100
200
Time [s]
Chest HR
Wrist HR

```
## ------------------------------------------------------------------------------- ##
```

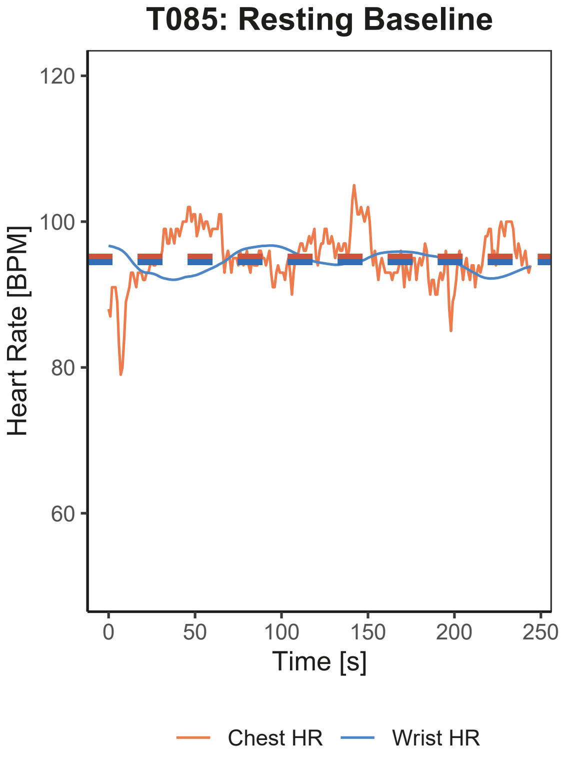
T085: Resting Baseline
Heart Rate [BPM]
120
100
80
60
0
50
100
150
200
250
Time [s]
Chest HR
Wrist HR

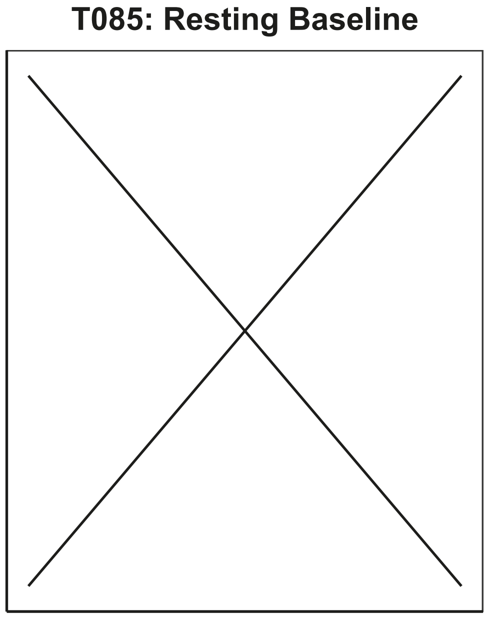
T085: Resting Baseline

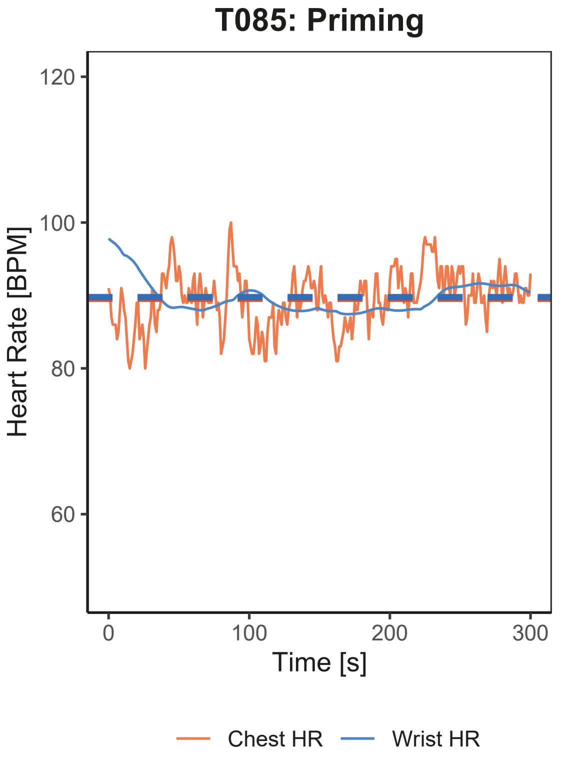
T085: Priming
Heart Rate [BPM]
120
100
80
60
0
100
200
300
Time [s]
Chest HR
Wrist HR

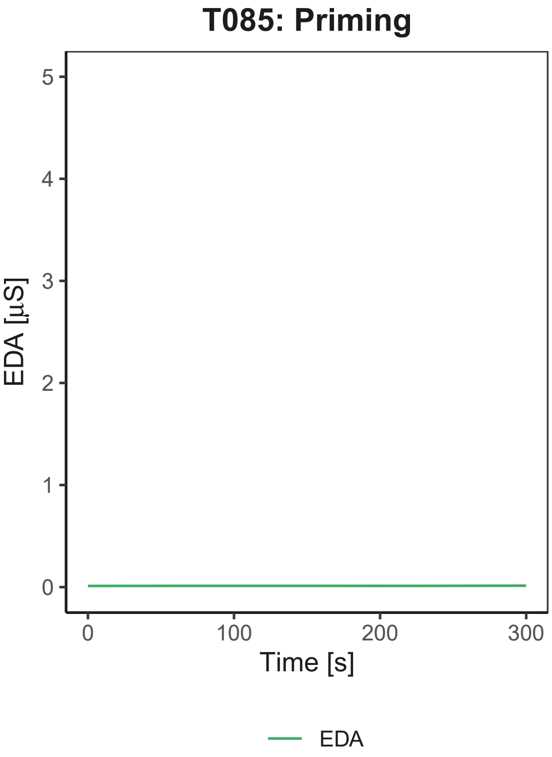
T085: Priming
EDA [µS]
5
4
3
2
1
0
0
100
200
300
Time [s]
EDA

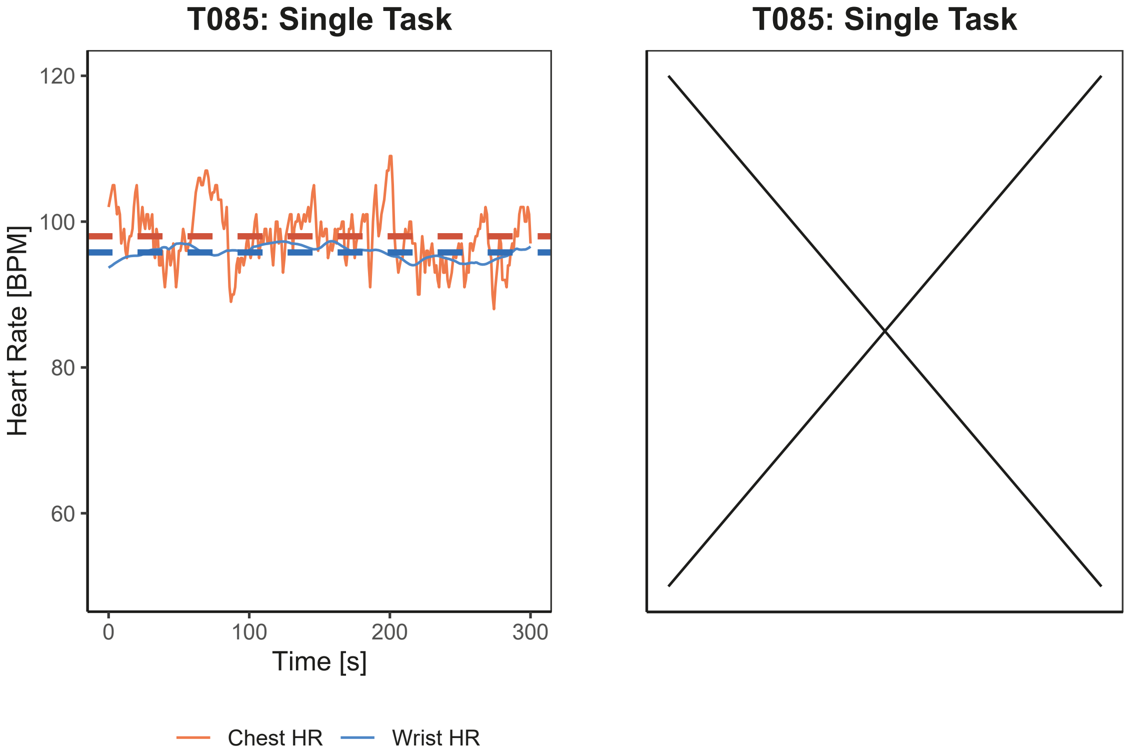
T085: Single Task
T085: Single Task
Heart Rate [BPM]
120
100
80
60
0
100
200
300
Time [s]
Chest HR
Wrist HR

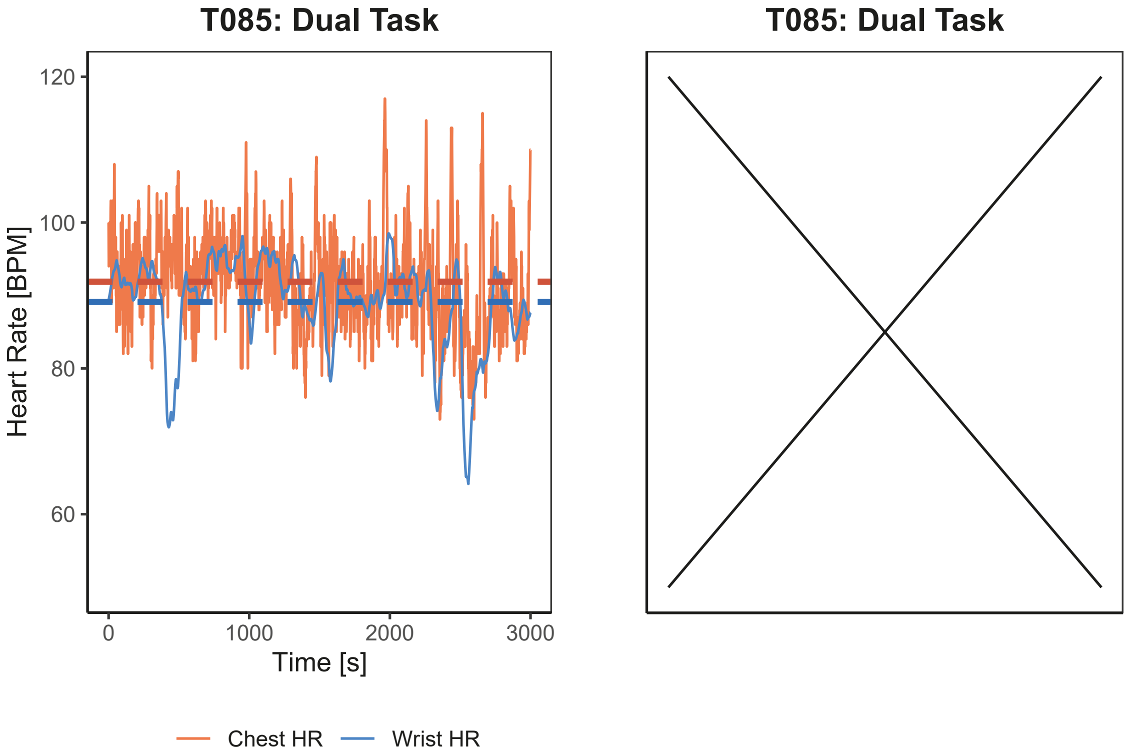
T085: Dual Task
T085: Dual Task
Heart Rate [BPM]
120
100
80
60
0
1000
2000
3000
Time [s]
Chest HR
Wrist HR

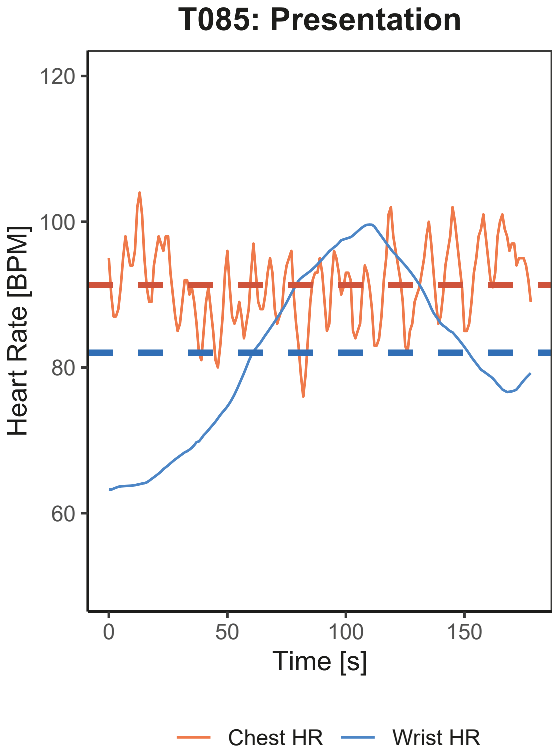
T085: Presentation
Heart Rate [BPM]
120
100
80
60
0
50
100
150
Time [s]
Chest HR
Wrist HR

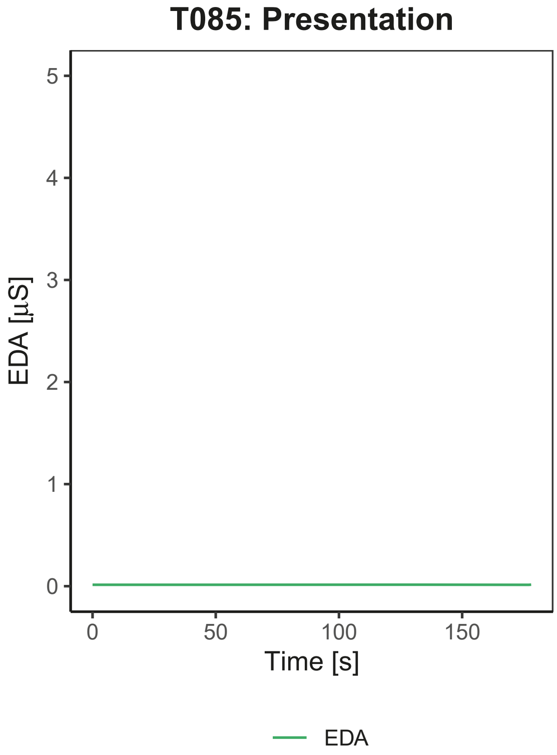
T085: Presentation
EDA [µS]
5
4
3
2
1
0
0
50
100
150
Time [s]
EDA

--

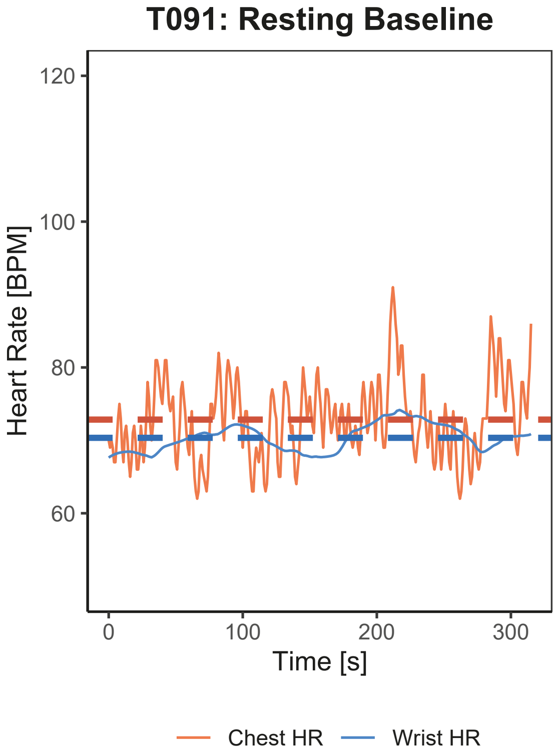
T091: Resting Baseline
Heart Rate [BPM]
120
100
80
60
0
100
200
300
Time [s]
Chest HR
Wrist HR

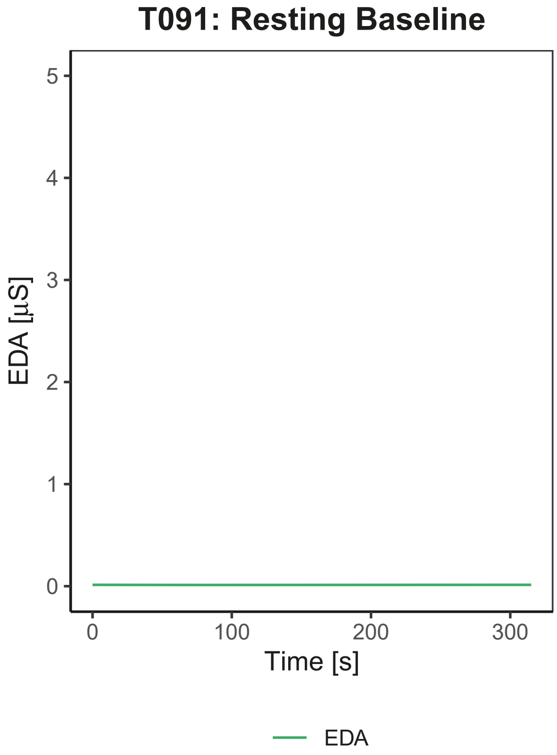
T091: Resting Baseline
EDA [µS]
5
4
3
2
1
0
0
100
200
300
Time [s]
EDA

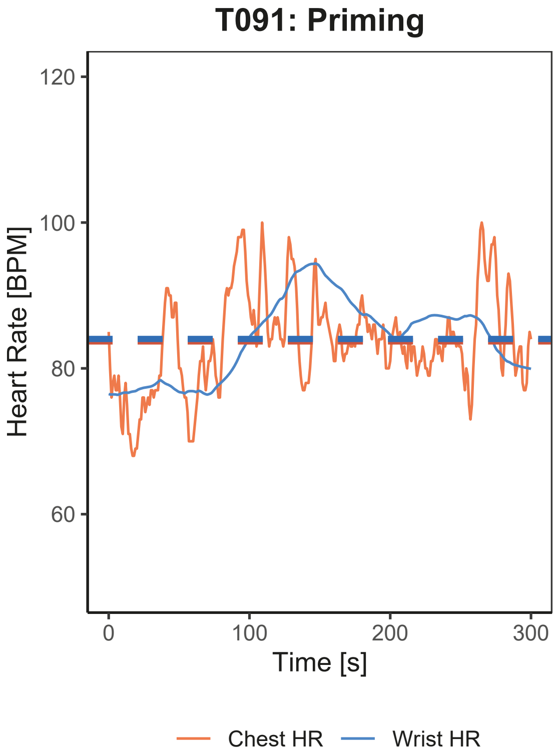
T091: Priming
Heart Rate [BPM]
120
100
80
60
0
100
200
300
Time [s]
Chest HR
Wrist HR

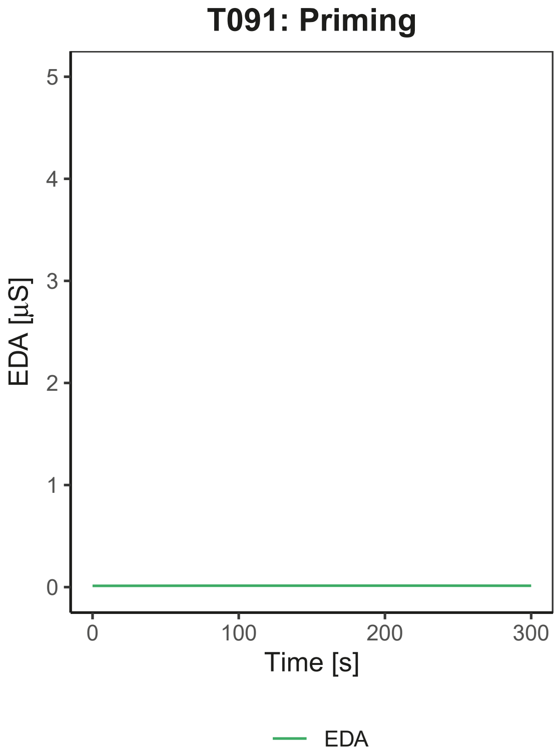
T091: Priming
EDA [µS]
5
4
3
2
1
0
0
100
200
300
Time [s]
EDA

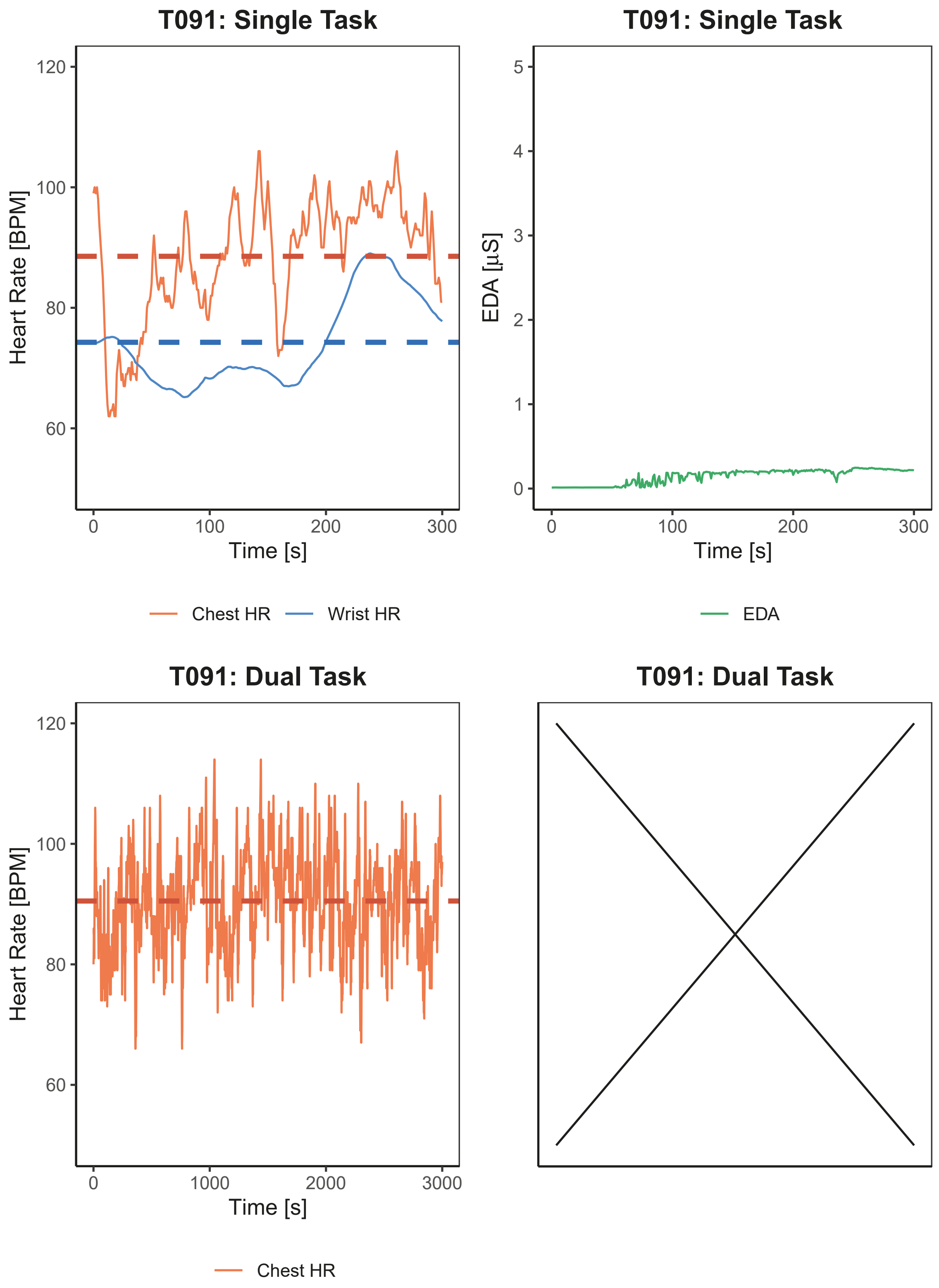
T091: Single Task
Heart Rate [BPM]
Time [s]
Chest HR
Wrist HR
T091: Single Task
EDA [µS]
Time [s]
EDA
T091: Dual Task
Heart Rate [BPM]
Time [s]
Chest HR
T091: Dual Task

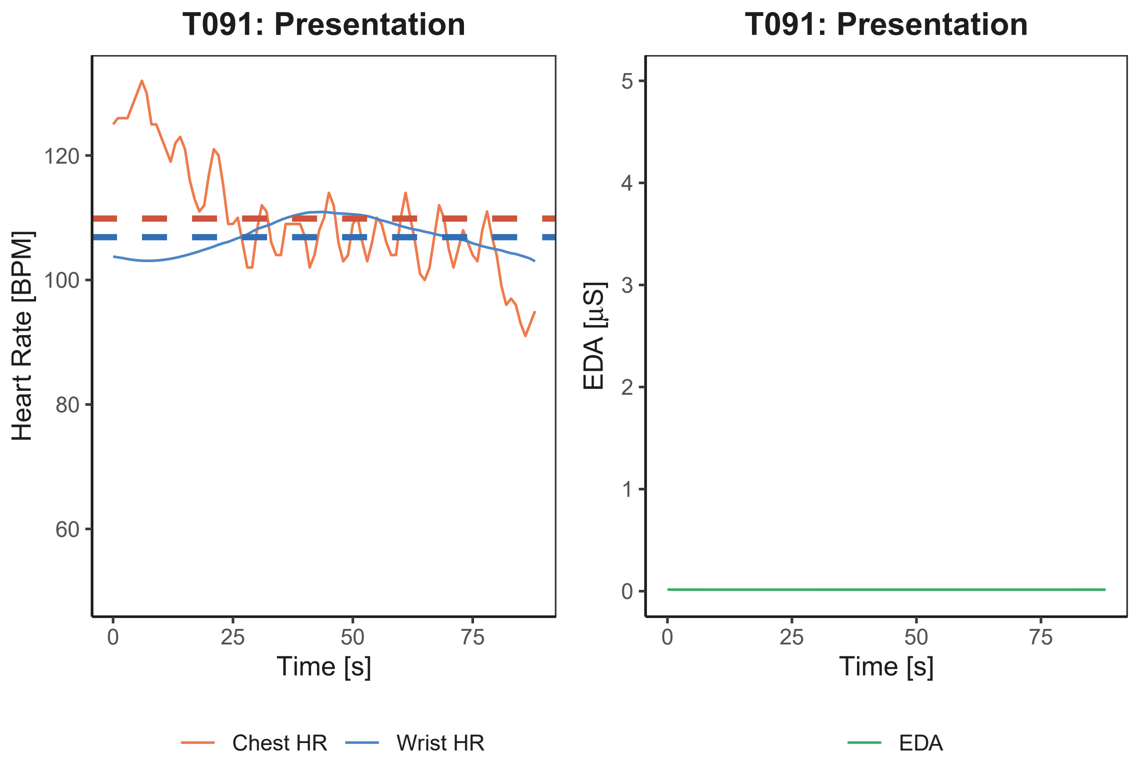
T091: Presentation
Heart Rate [BPM]
120
100
80
60
0
25
50
75
Time [s]
Chest HR
Wrist HR
T091: Presentation
EDA [µS]
5
4
3
2
1
0
0
25
50
75
Time [s]
EDA

--

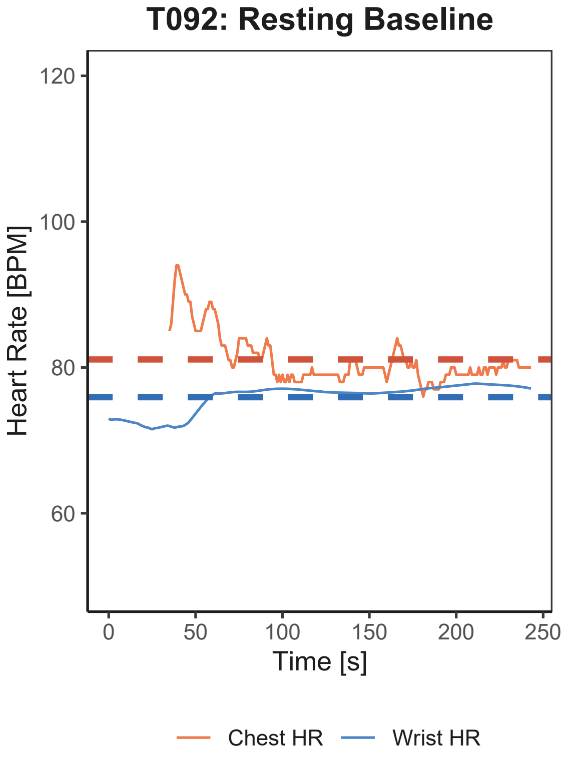
T092: Resting Baseline
Heart Rate [BPM]
120
100
80
60
0
50
100
150
200
250
Time [s]
Chest HR
Wrist HR

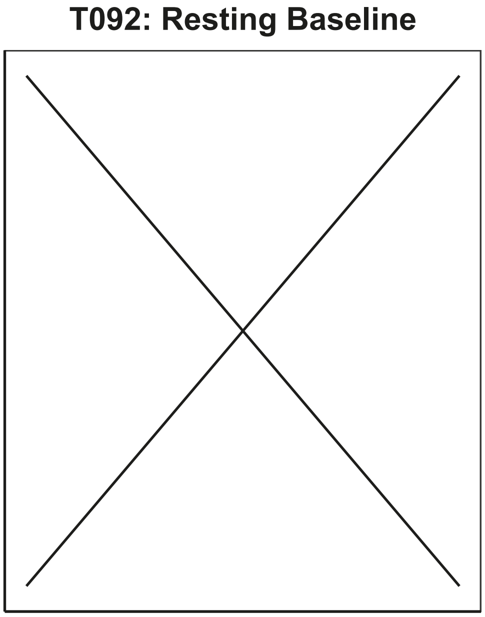
T092: Resting Baseline

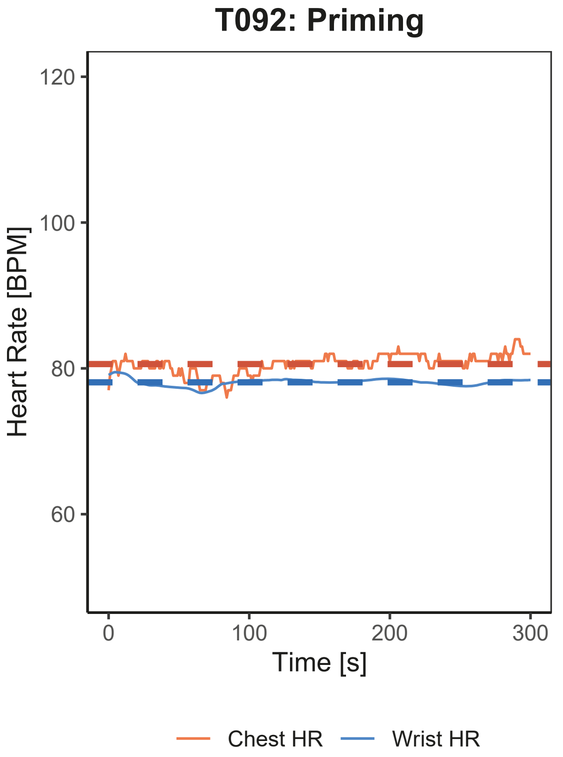
T092: Priming
Heart Rate [BPM]
120
100
80
60
0
100
200
300
Time [s]
Chest HR
Wrist HR

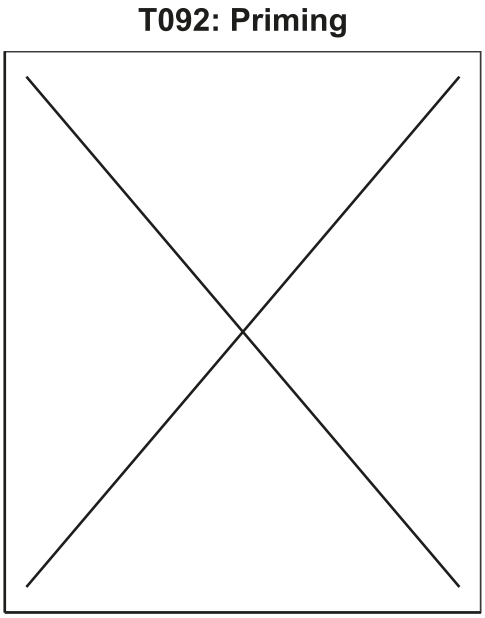
T092: Priming

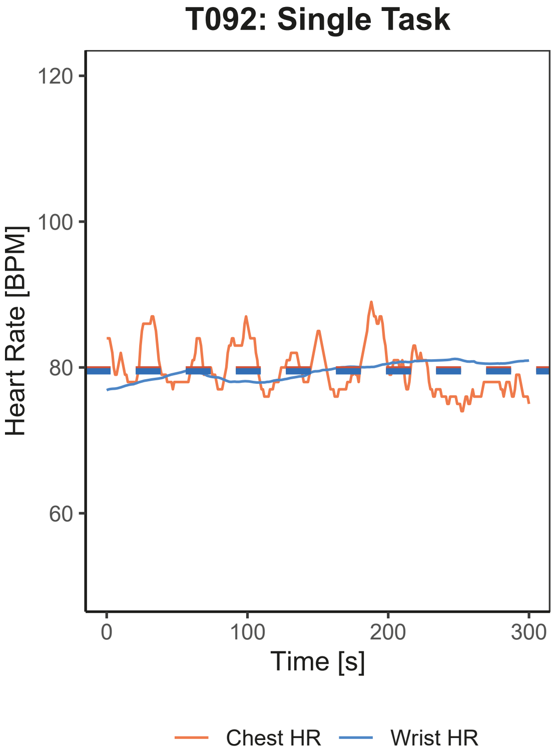
T092: Single Task
Heart Rate [BPM]
120
100
80
60
0
100
200
300
Time [s]
Chest HR
Wrist HR

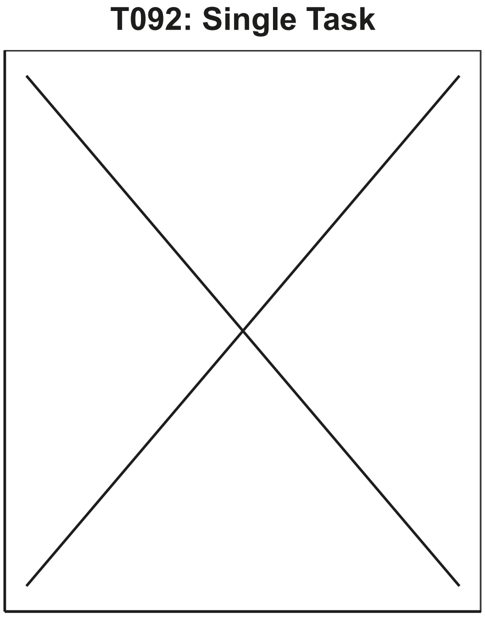
T092: Single Task

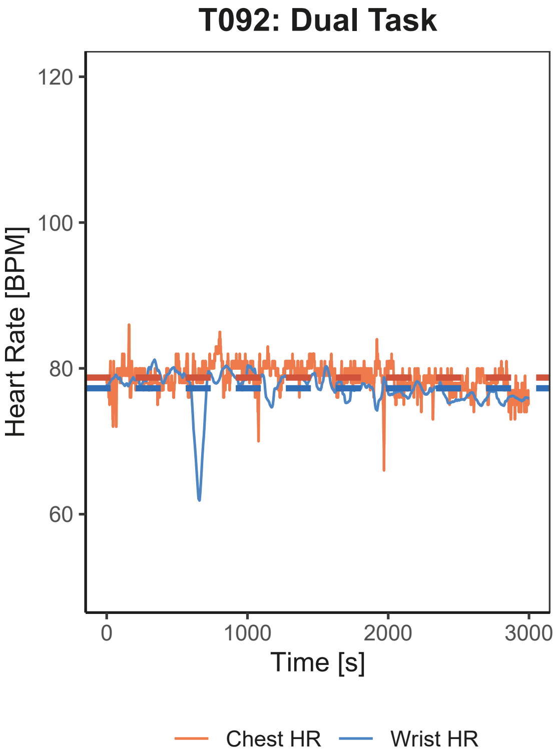
T092: Dual Task
Heart Rate [BPM]
120
100
80
60
0
1000
2000
3000
Time [s]
Chest HR
Wrist HR

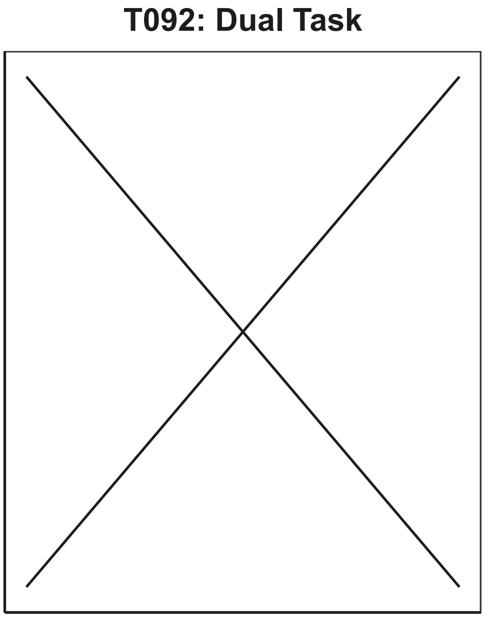
T092: Dual Task

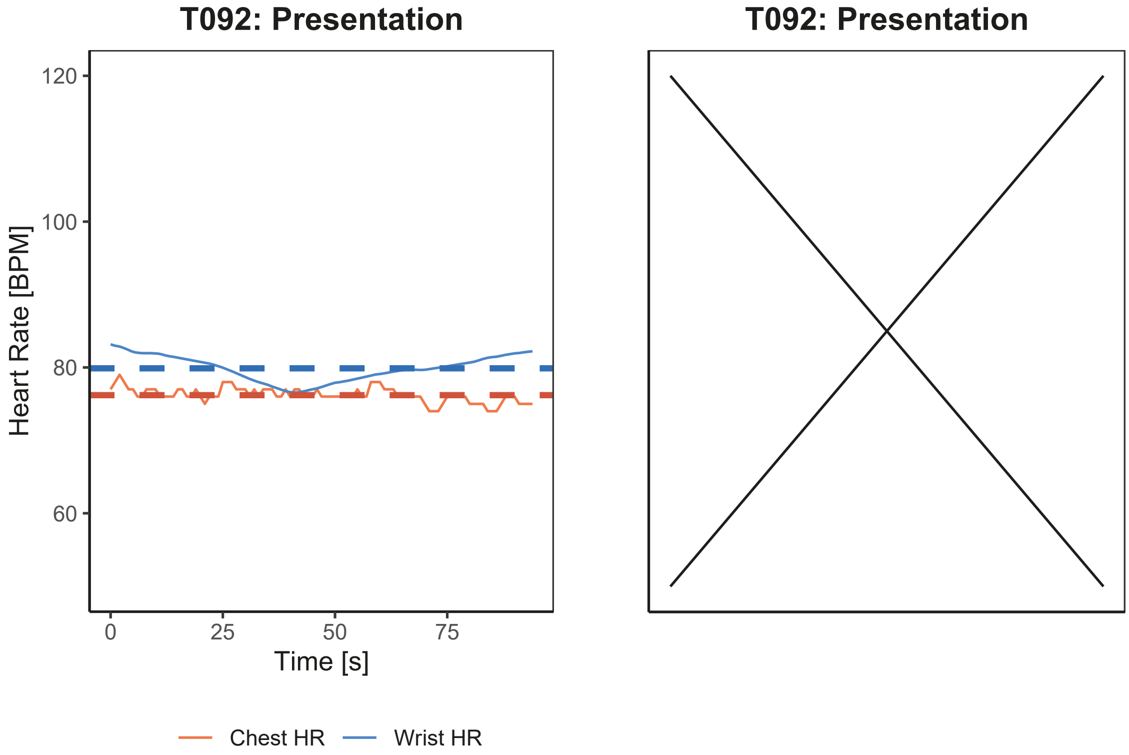
T092: Presentation
T092: Presentation
Heart Rate [BPM]
120
100
80
60
0
25
50
75
Time [s]
Chest HR
Wrist HR

--

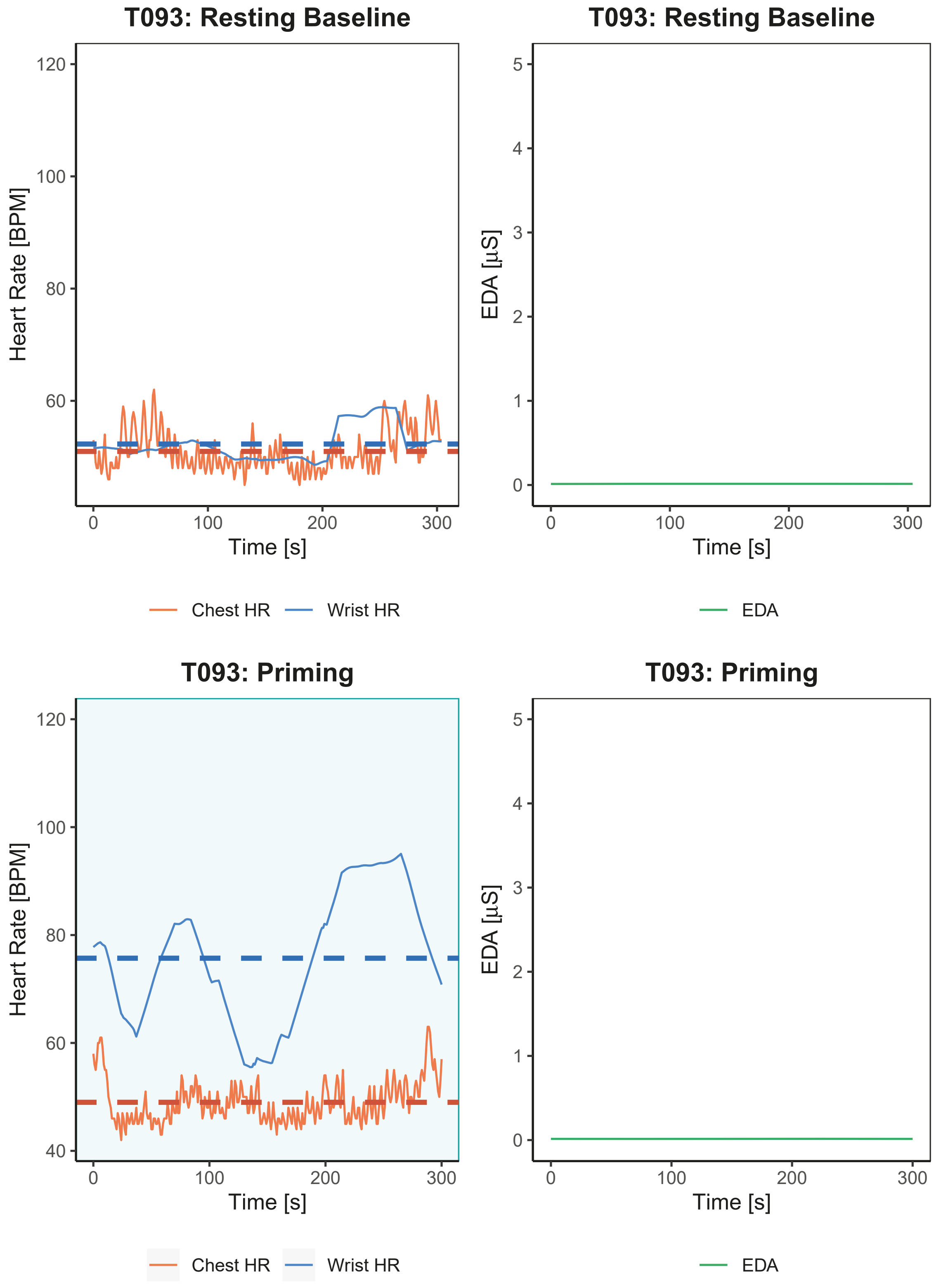
T093: Resting Baseline
Heart Rate [BPM]
120
100
80
60
0
100
200
300
Time [s]
Chest HR
Wrist HR
T093: Resting Baseline
EDA [µS]
5
4
3
2
1
0
0
100
200
300
Time [s]
EDA
T093: Priming
Heart Rate [BPM]
120
100
80
60
40
0
100
200
300
Time [s]
Chest HR
Wrist HR
T093: Priming
EDA [µS]
5
4
3
2
1
0
0
100
200
300
Time [s]
EDA

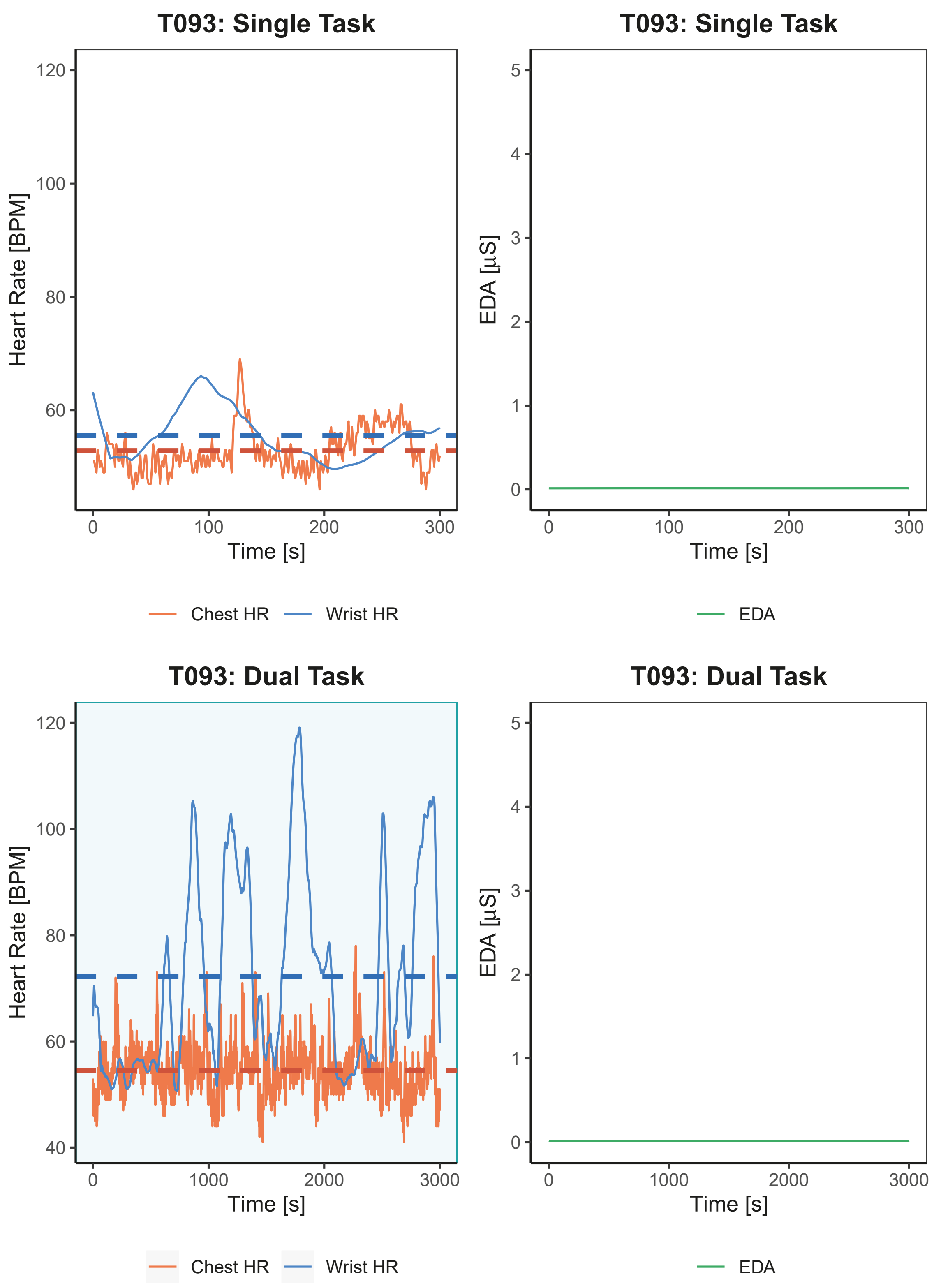
T093: Single Task
Heart Rate [BPM]
120
100
80
60
0
100
200
300
Time [s]
Chest HR
Wrist HR
T093: Single Task
EDA [µS]
5
4
3
2
1
0
0
100
200
300
Time [s]
EDA
T093: Dual Task
Heart Rate [BPM]
120
100
80
60
40
0
1000
2000
3000
Time [s]
Chest HR
Wrist HR
T093: Dual Task
EDA [µS]
5
4
3
2
1
0
0
1000
2000
3000
Time [s]
EDA

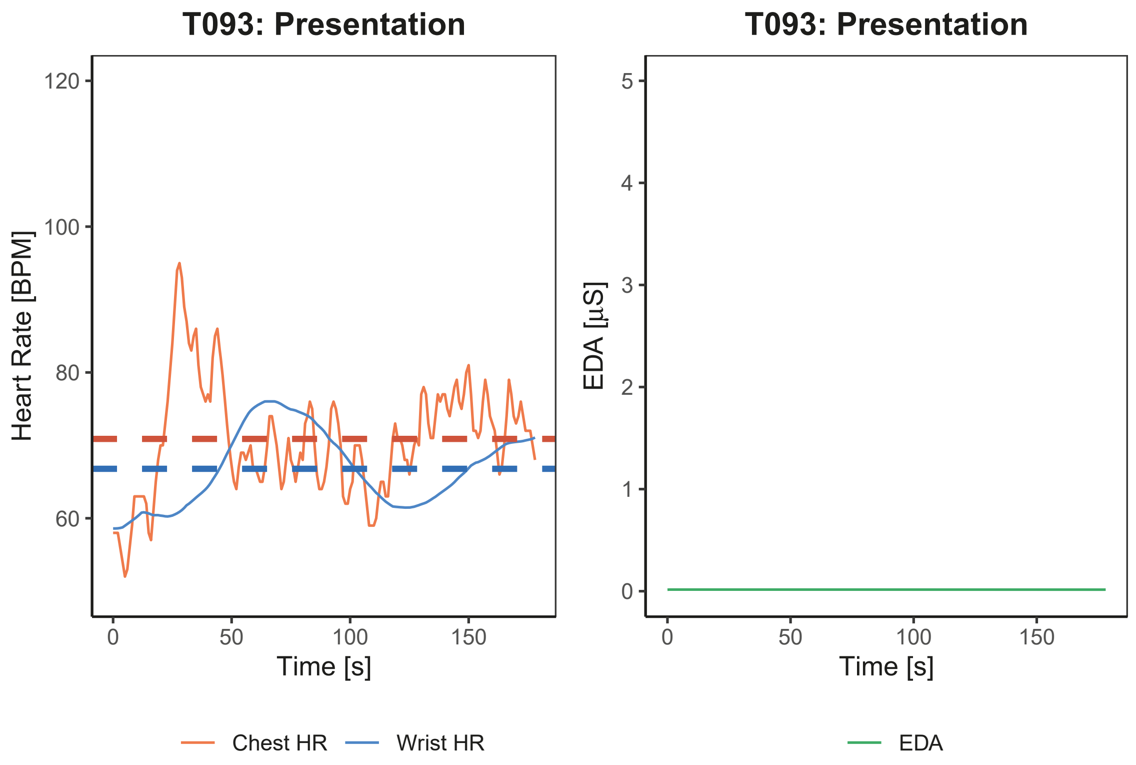
T093: Presentation
Heart Rate [BPM]
120
100
80
60
0
50
100
150
Time [s]
T093: Presentation
EDA [µS]
5
4
3
2
1
0
0
50
100
150
Time [s]
Chest HR
Wrist HR
EDA

--

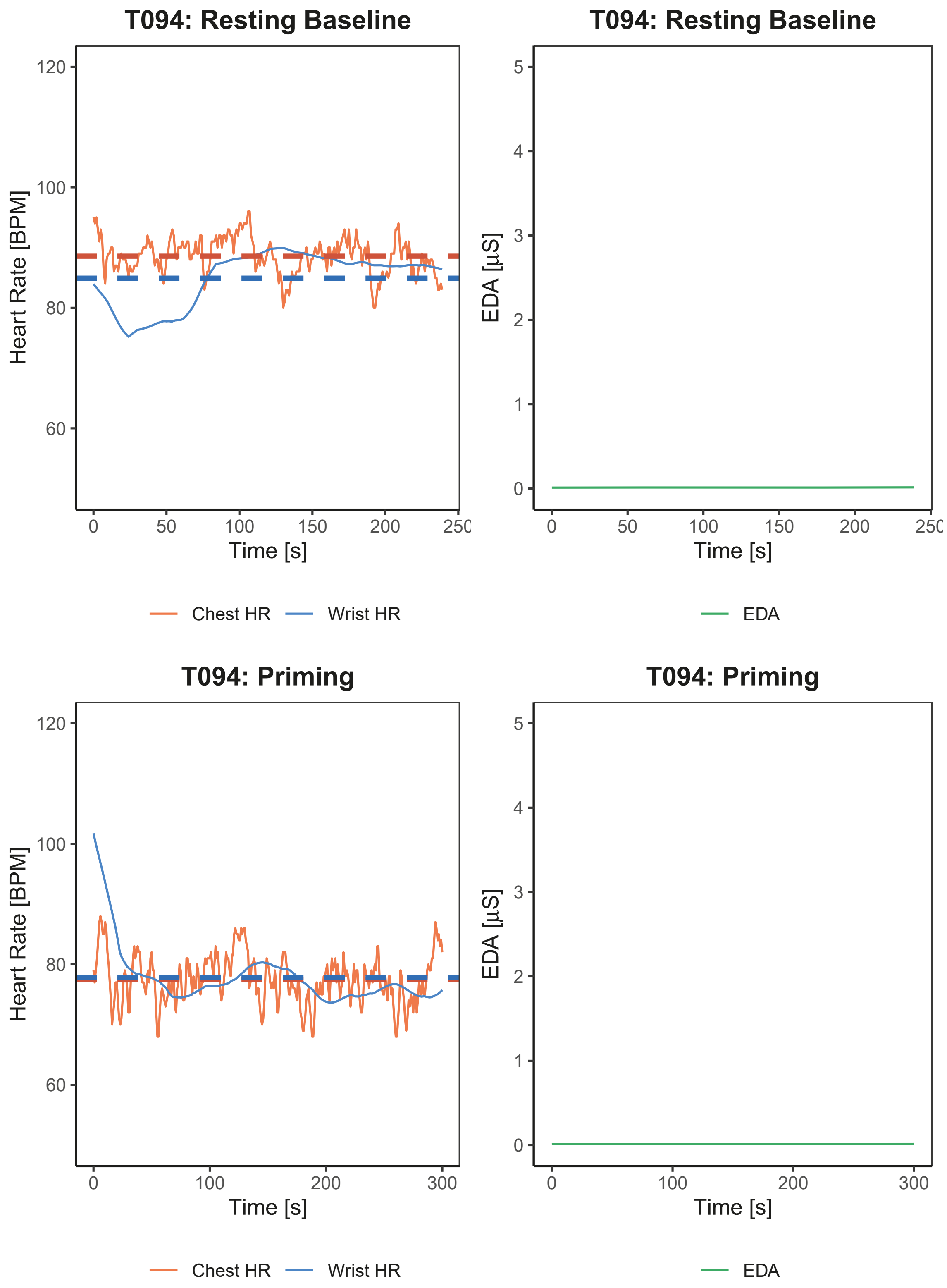
T094: Resting Baseline
Heart Rate [BPM]
120
100
80
60
0
50
100
150
200
250
Time [s]
Chest HR
Wrist HR
T094: Resting Baseline
EDA [µS]
5
4
3
2
1
0
0
50
100
150
200
250
Time [s]
EDA
T094: Priming
Heart Rate [BPM]
120
100
80
60
0
100
200
300
Time [s]
Chest HR
Wrist HR
T094: Priming
EDA [µS]
5
4
3
2
1
0
0
100
200
300
Time [s]
EDA

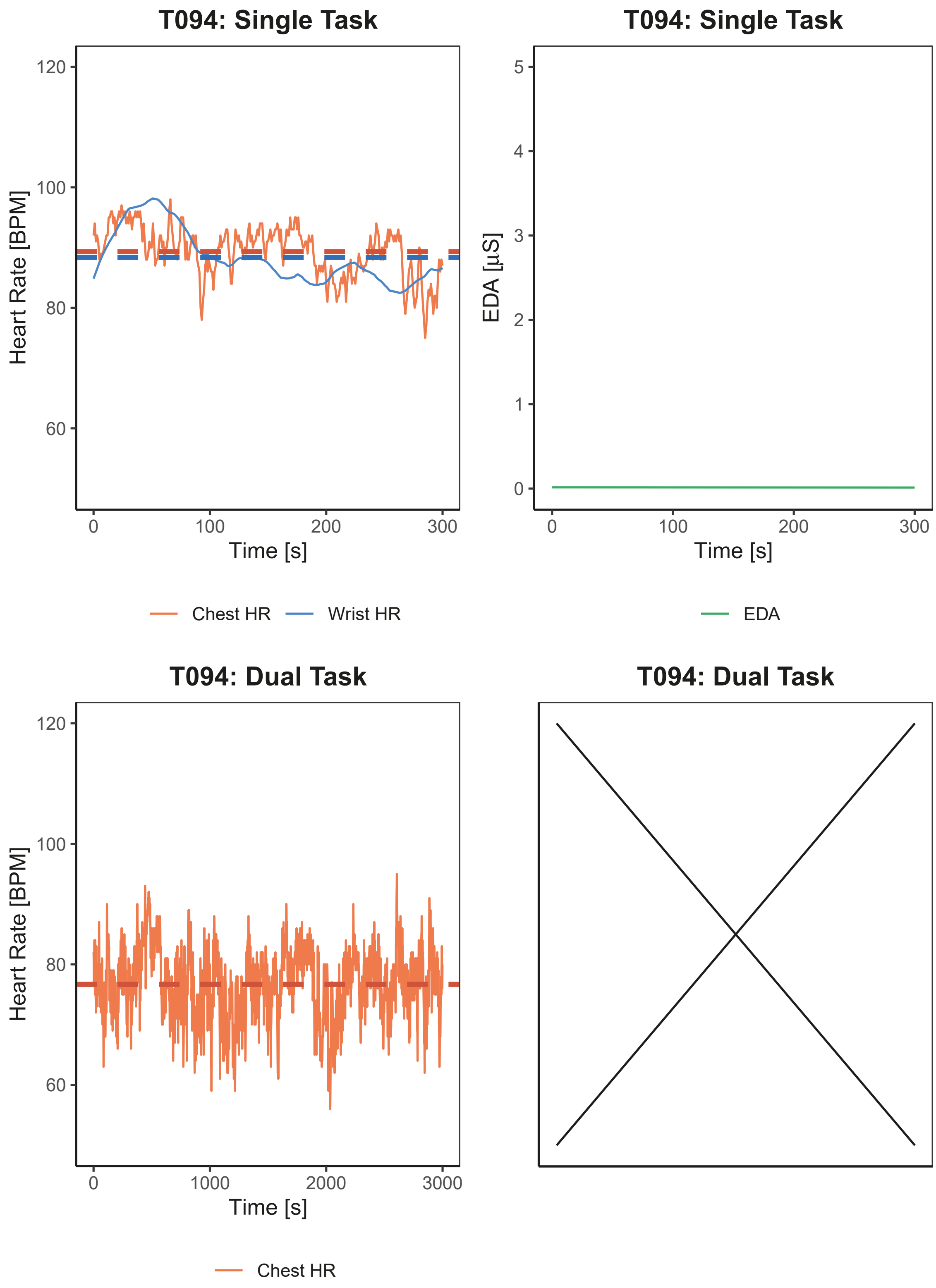
T094: Single Task
Heart Rate [BPM]
120
100
80
60
0
100
200
300
Time [s]
Chest HR
Wrist HR
T094: Single Task
EDA [µS]
5
4
3
2
1
0
0
100
200
300
Time [s]
EDA
T094: Dual Task
Heart Rate [BPM]
120
100
80
60
0
1000
2000
3000
Time [s]
Chest HR
T094: Dual Task

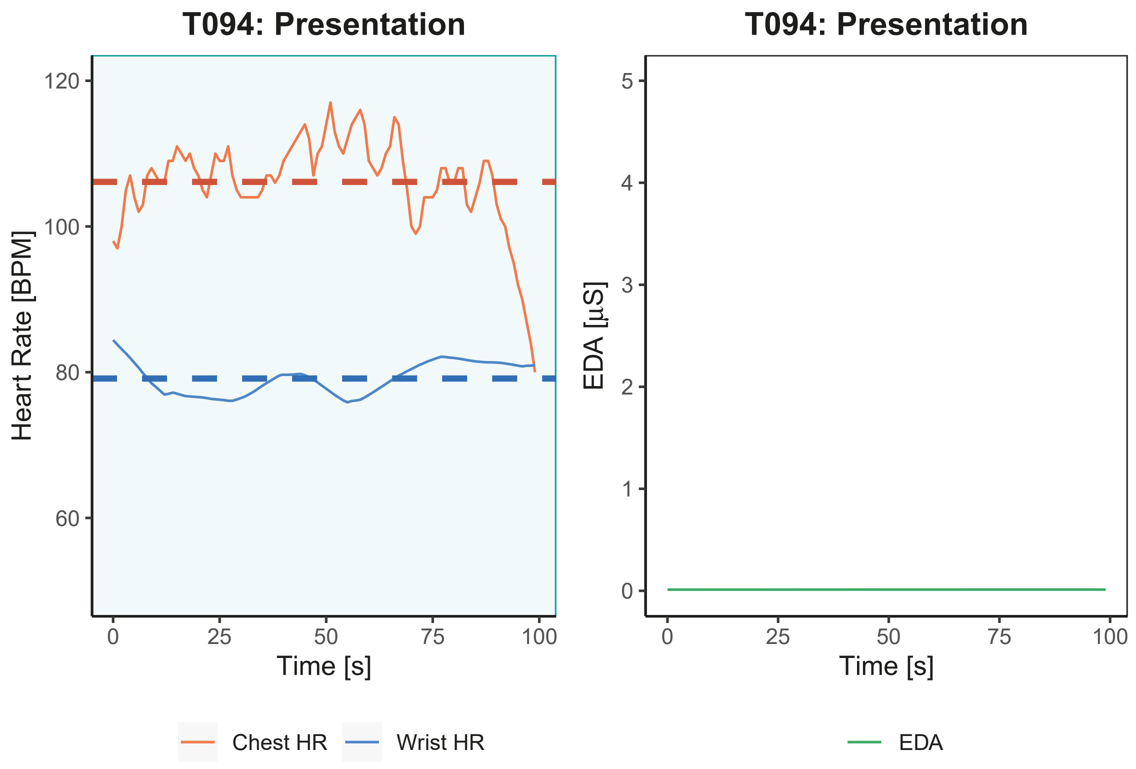

T094: Presentation
Heart Rate [BPM]
120
100
80
60
0
25
50
75
100
Time [s]
Chest HR
Wrist HR
T094: Presentation
EDA [µS]
5
4
3
2
1
0
0
25
50
75
100
Time [s]
EDA

--

T096: Resting Baseline

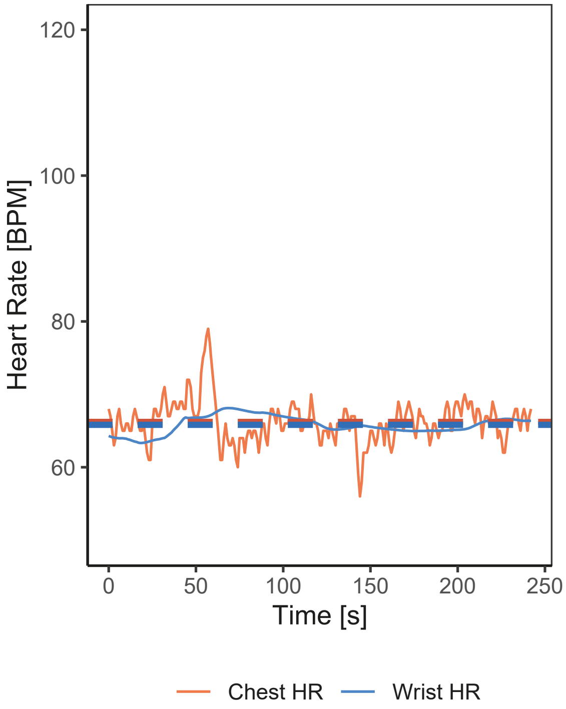

T096: Resting Baseline

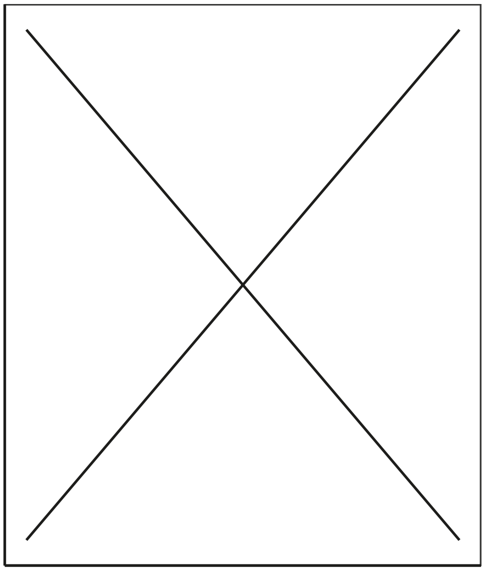

T096: Priming

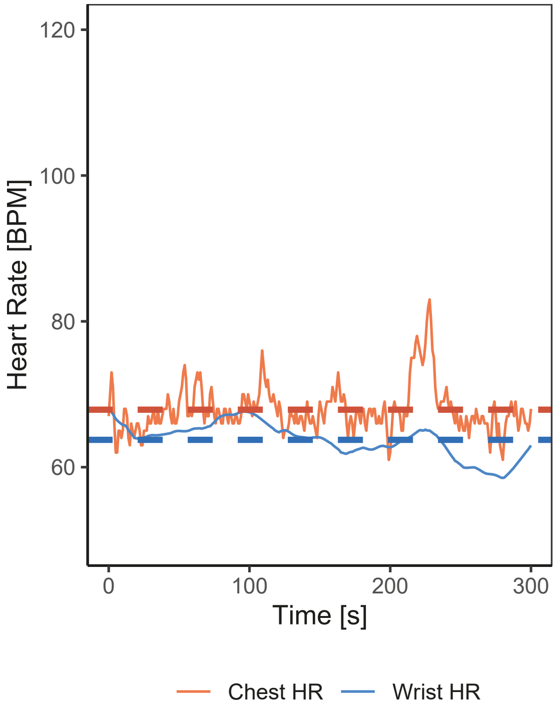

T096: Priming

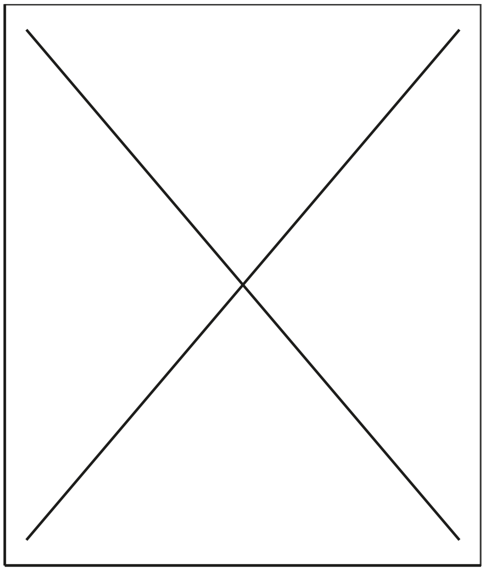

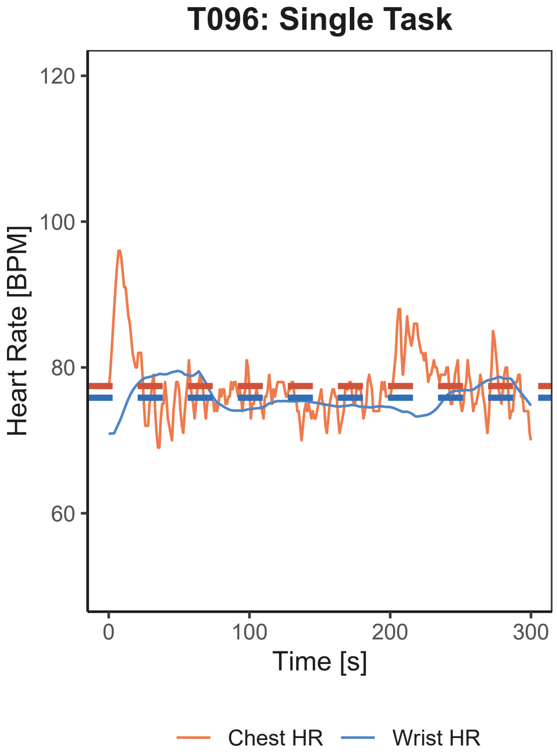
T096: Single Task
Heart Rate [BPM]
120
100
80
60
0
100
200
300
Time [s]
Chest HR
Wrist HR

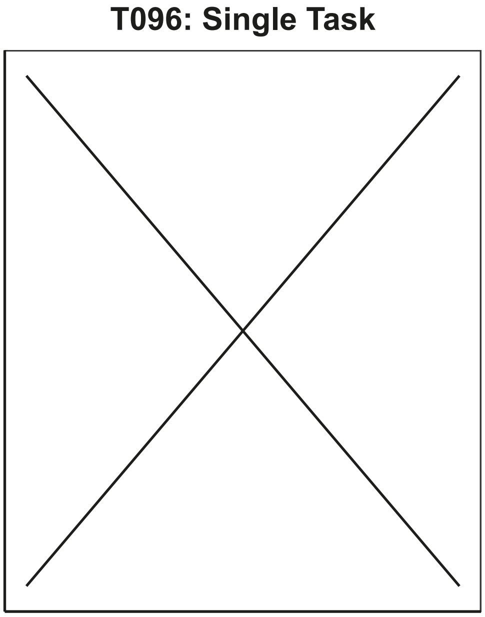
T096: Single Task

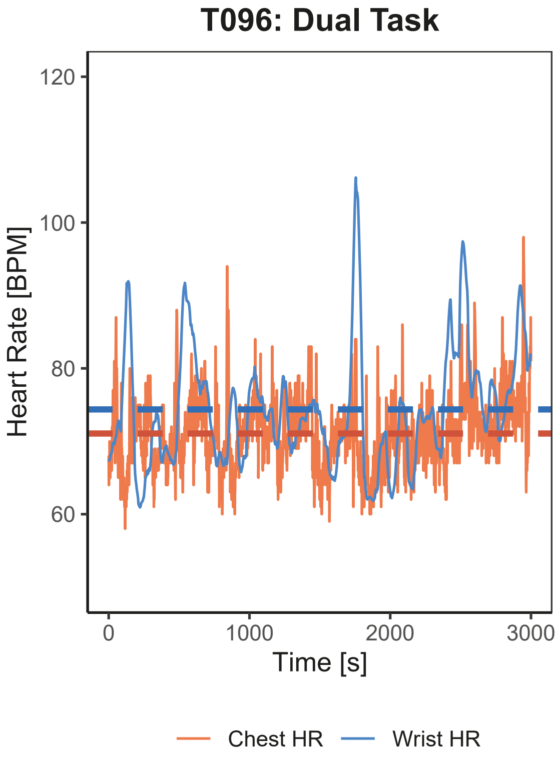
T096: Dual Task
Heart Rate [BPM]
120
100
80
60
0
1000
2000
3000
Time [s]
Chest HR
Wrist HR

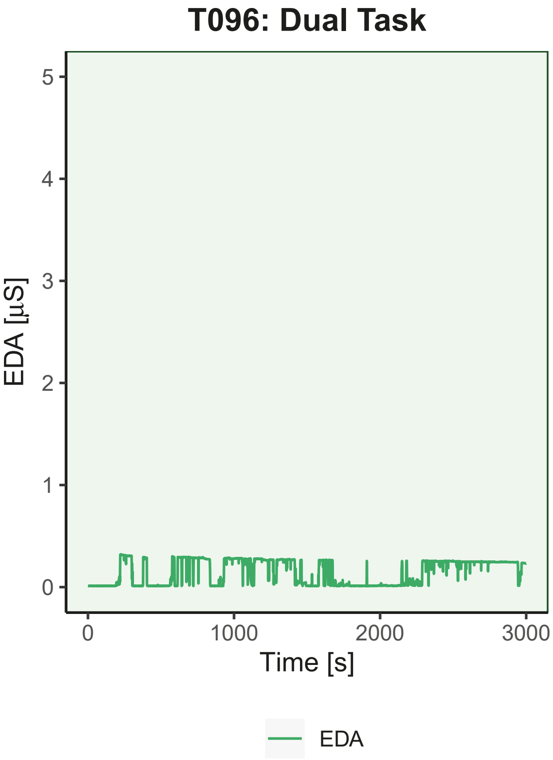
T096: Dual Task
EDA [µS]
5
4
3
2
1
0
0
1000
2000
3000
Time [s]
EDA

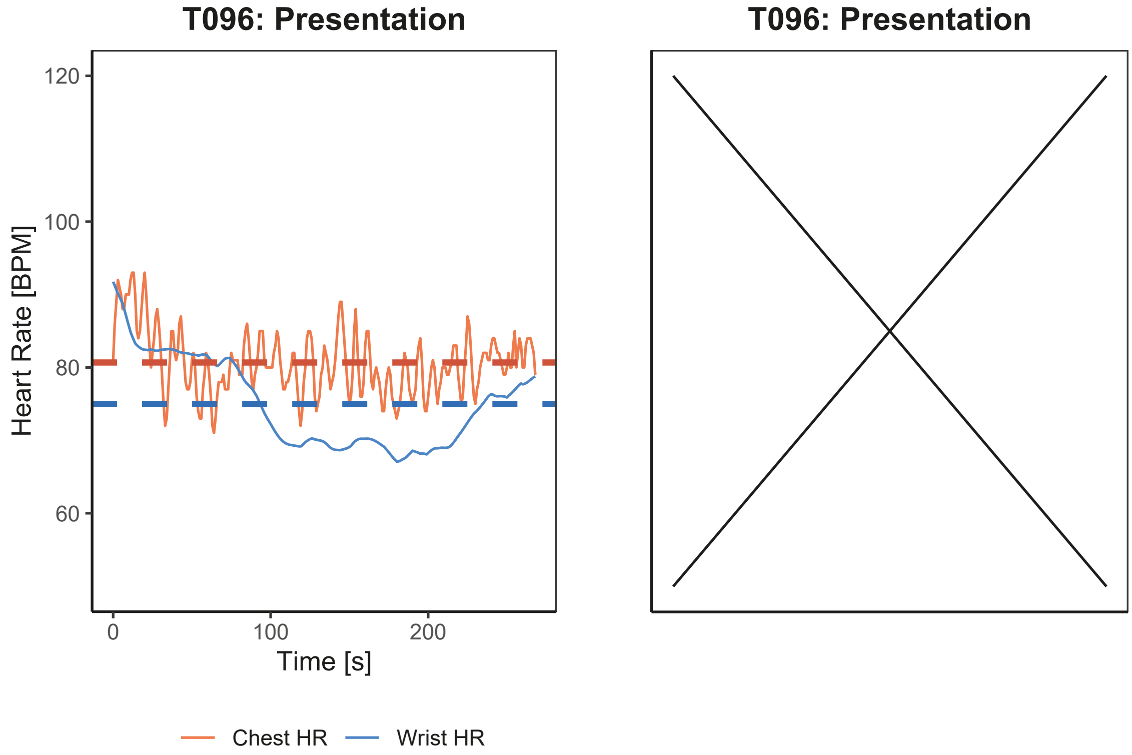
T096: Presentation
T096: Presentation
120
100
80
60
Heart Rate [BPM]
0
100
200
Time [s]
Chest HR
Wrist HR

```
## ------------------------------------------------------------------------------- ##
```

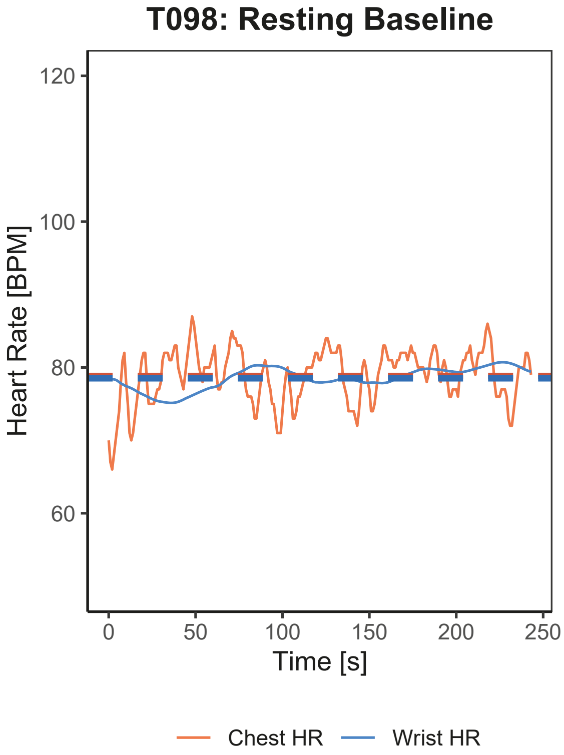
T098: Resting Baseline
Heart Rate [BPM]
120
100
80
60
0
50
100
150
200
250
Time [s]
Chest HR
Wrist HR

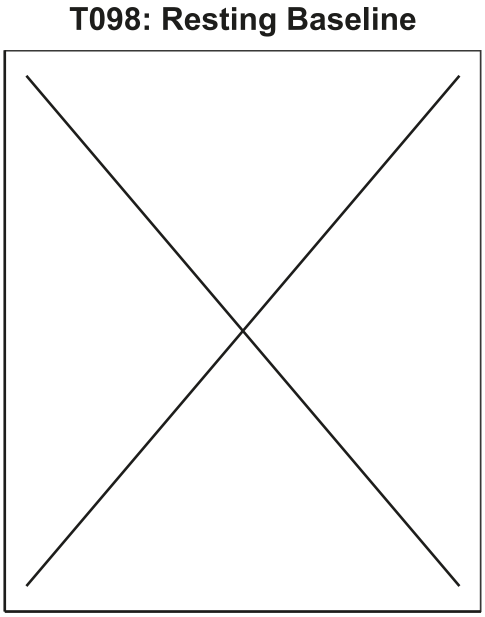
T098: Resting Baseline

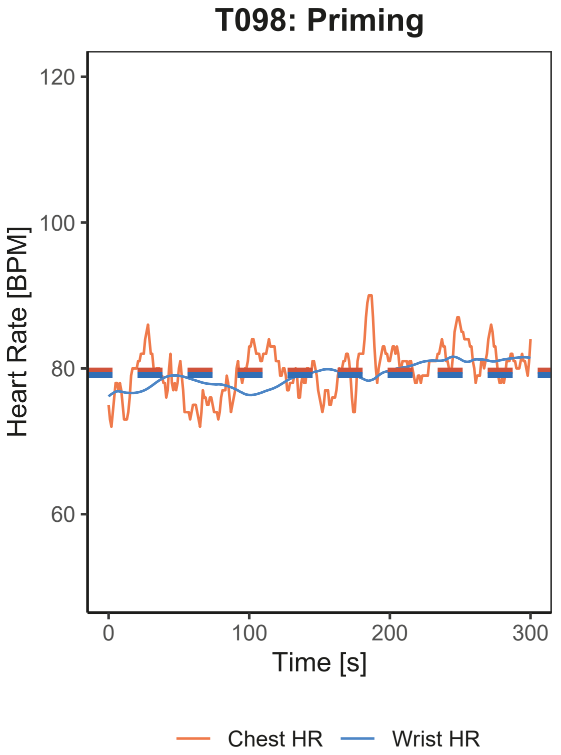
T098: Priming
Heart Rate [BPM]
120
100
80
60
0
100
200
300
Time [s]
Chest HR
Wrist HR

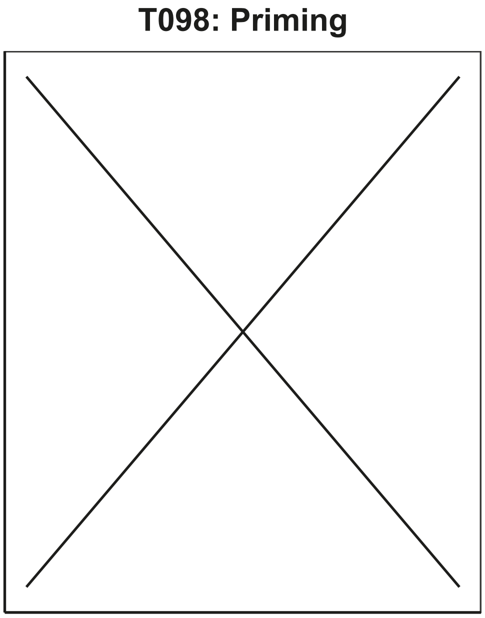
T098: Priming

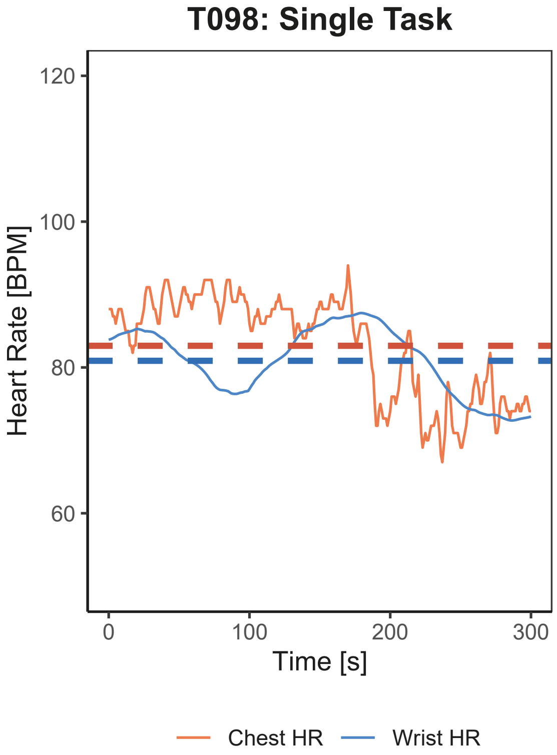
T098: Single Task
Heart Rate [BPM]
120
100
80
60
0
100
200
300
Time [s]
Chest HR
Wrist HR

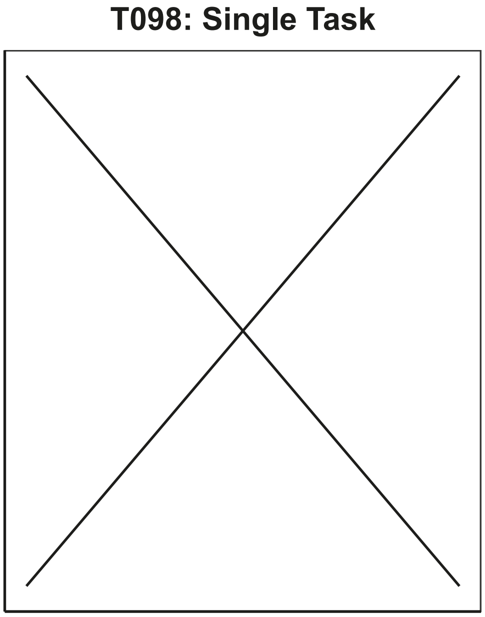
T098: Single Task

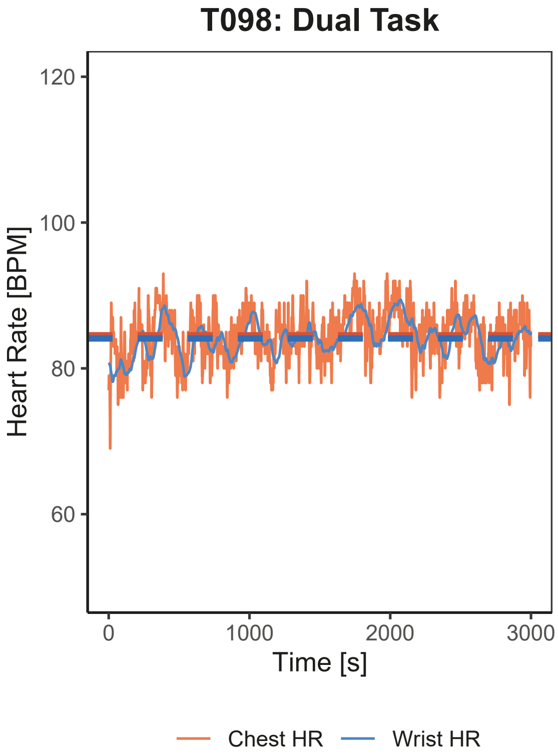
T098: Dual Task
Heart Rate [BPM]
120
100
80
60
0
1000
2000
3000
Time [s]
Chest HR
Wrist HR

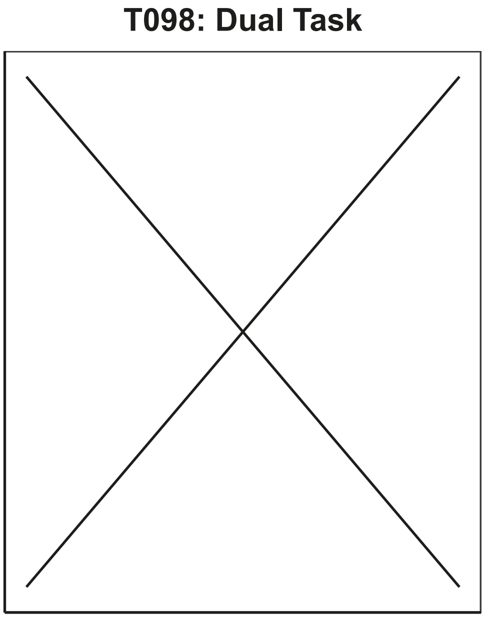
T098: Dual Task

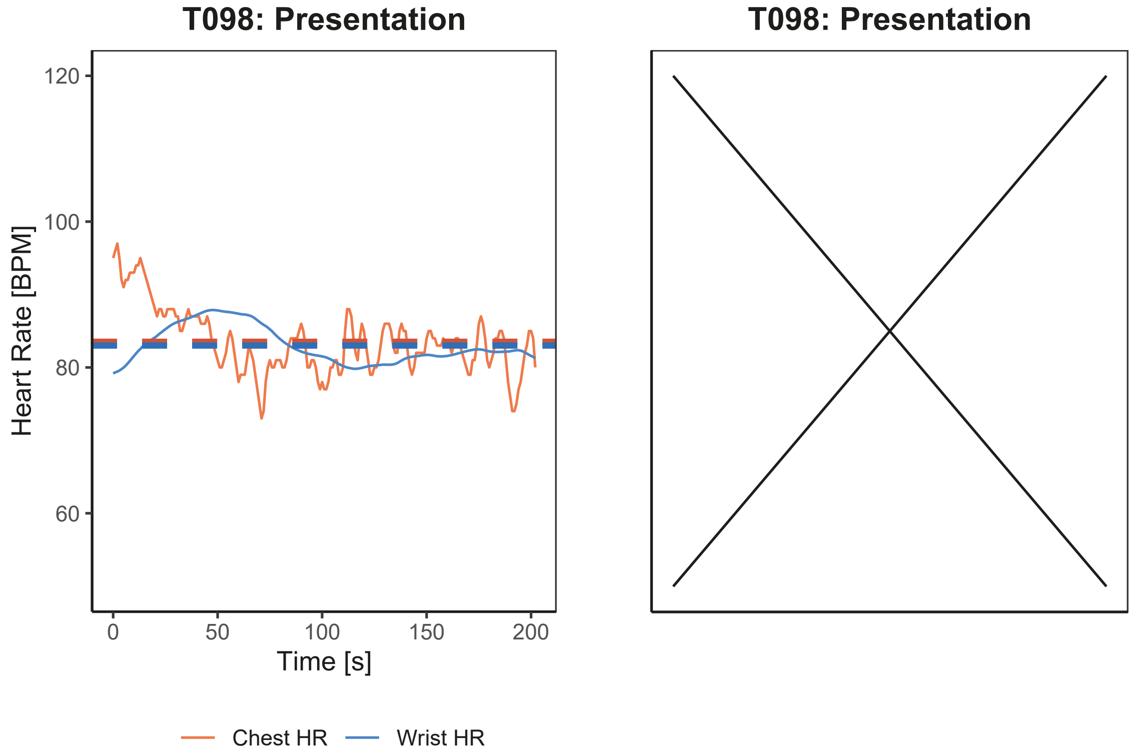
T098: Presentation
T098: Presentation
Heart Rate [BPM]
120
100
80
60
0
50
100
150
200
Time [s]
Chest HR
Wrist HR

```
## ---------------------------------------------------------------------------- ##
```

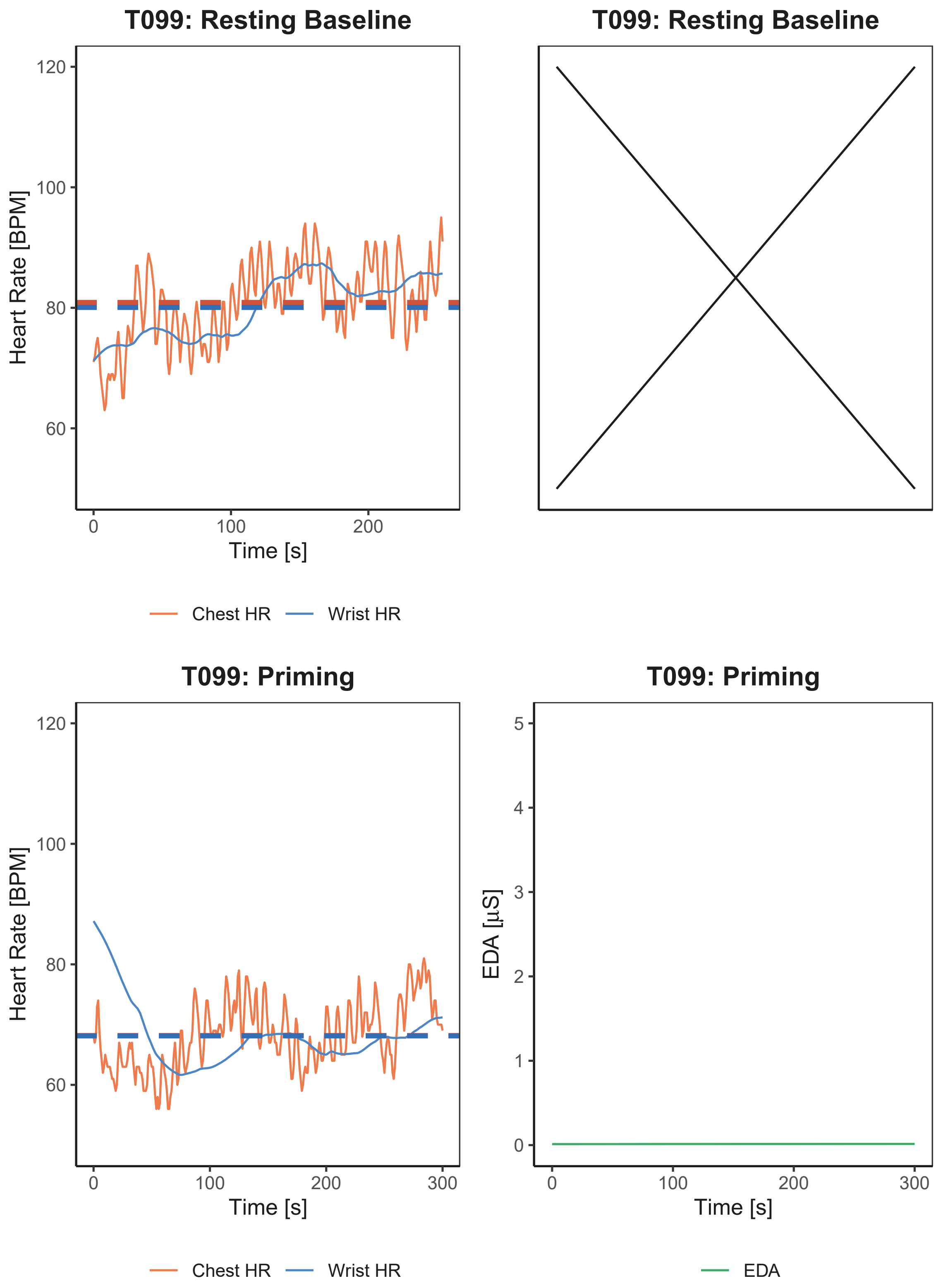
T099: Resting Baseline
Heart Rate [BPM]
120
100
80
60
0
100
200
Time [s]
Chest HR
Wrist HR
T099: Resting Baseline
T099: Priming
Heart Rate [BPM]
120
100
80
60
0
100
200
300
Time [s]
Chest HR
Wrist HR
T099: Priming
EDA [µS]
5
4
3
2
1
0
0
100
200
300
Time [s]
EDA

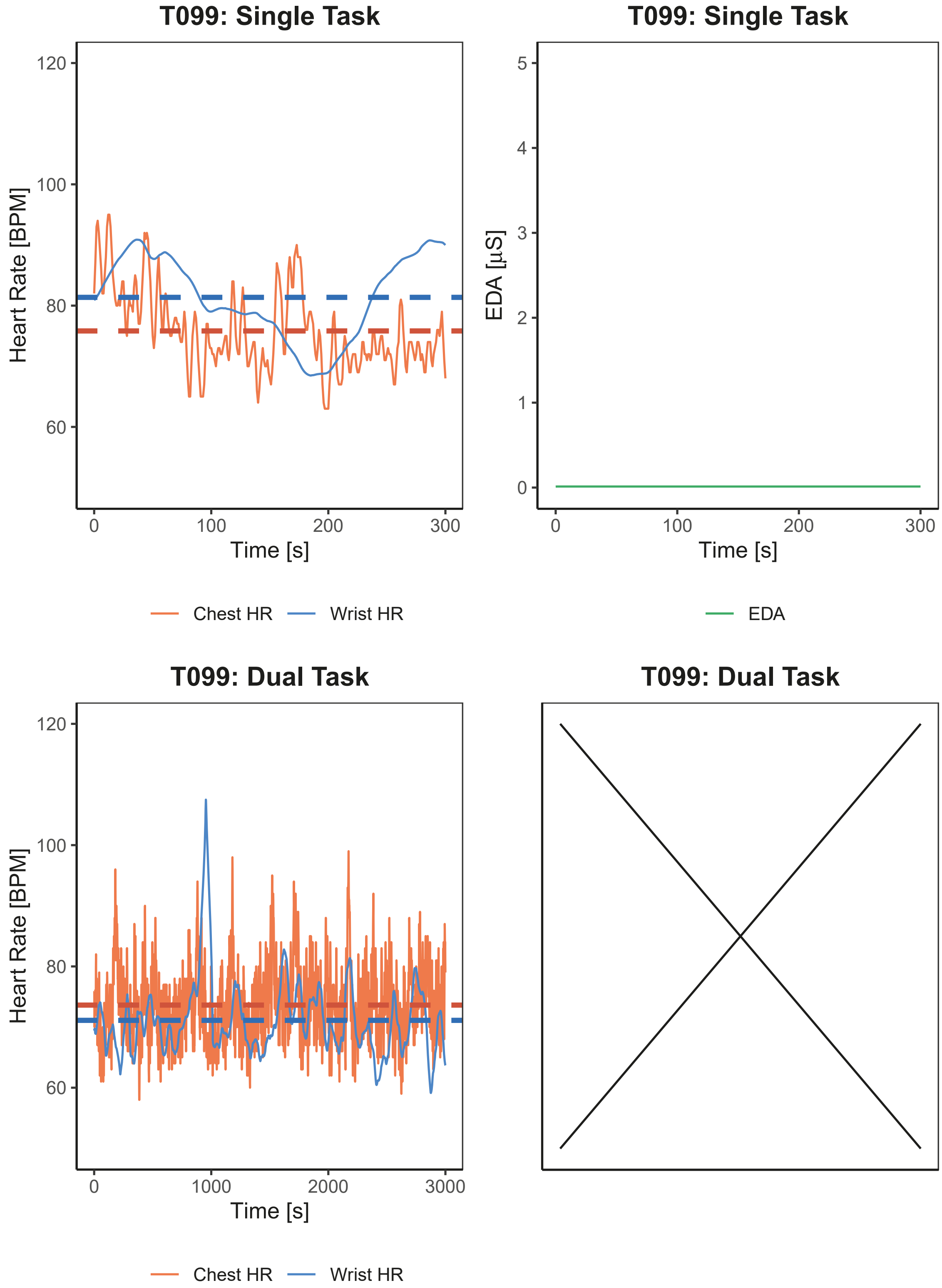
T099: Single Task
Heart Rate [BPM]
120
100
80
60
0
100
200
300
Time [s]
Chest HR
Wrist HR
T099: Single Task
EDA [µS]
5
4
3
2
1
0
0
100
200
300
Time [s]
EDA
T099: Dual Task
Heart Rate [BPM]
120
100
80
60
0
1000
2000
3000
Time [s]
Chest HR
Wrist HR
T099: Dual Task

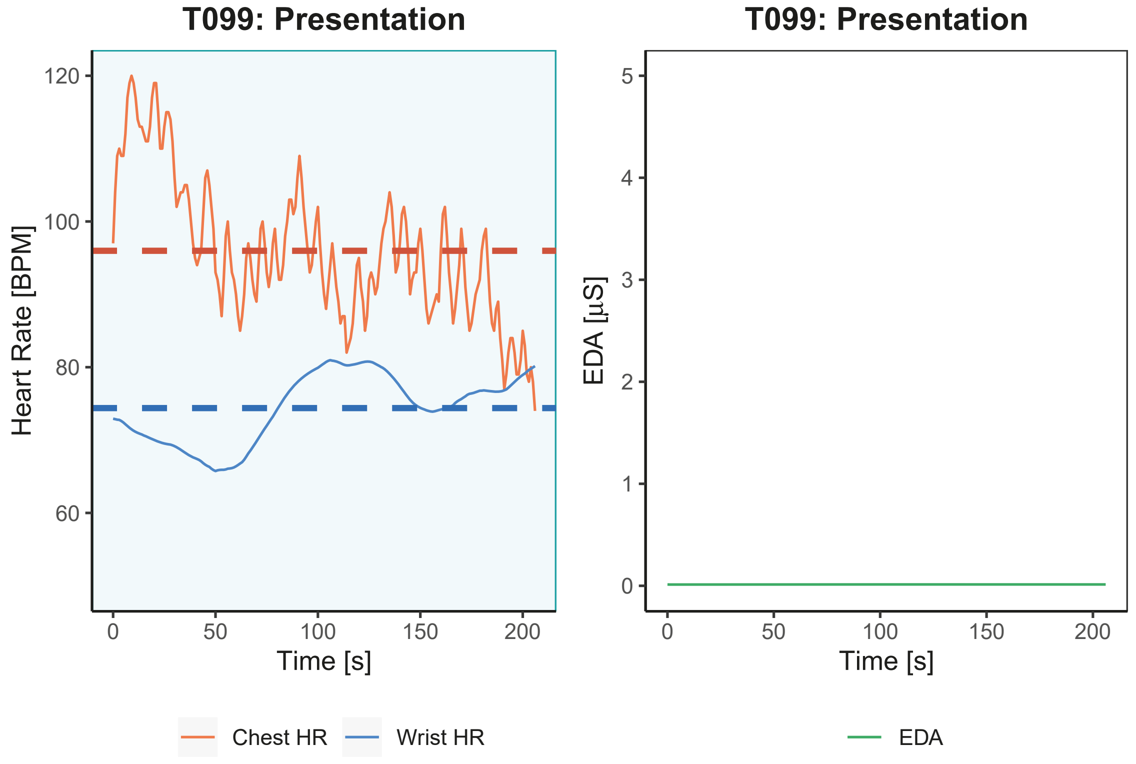
T099: Presentation
Heart Rate [BPM]
120
100
80
60
0
50
100
150
200
Time [s]
T099: Presentation
EDA [µS]
5
4
3
2
1
0
0
50
100
150
200
Time [s]
Chest HR
Wrist HR
EDA

--

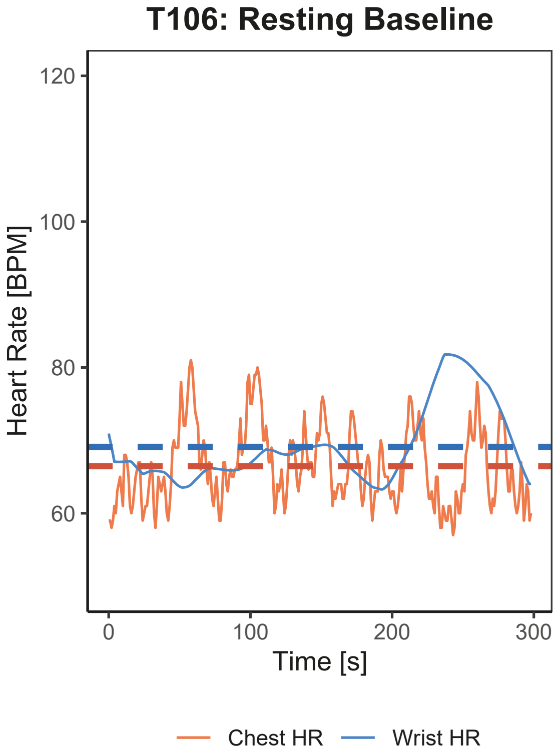
T106: Resting Baseline
Heart Rate [BPM]
120
100
80
60
0
100
200
300
Time [s]
Chest HR
Wrist HR

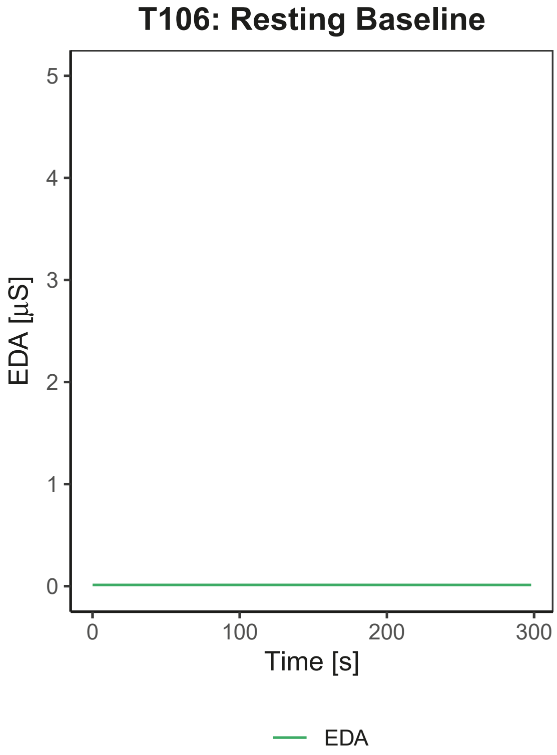
T106: Resting Baseline
EDA [µS]
5
4
3
2
1
0
0
100
200
300
Time [s]
EDA

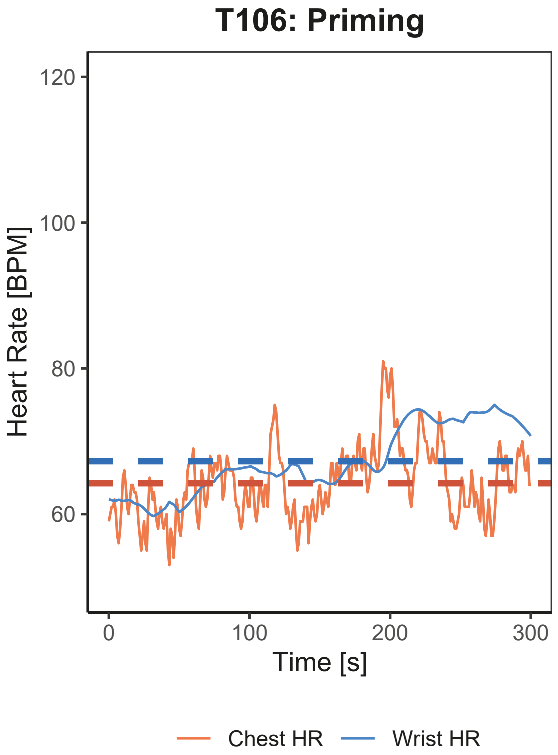
T106: Priming
Heart Rate [BPM]
120
100
80
60
0
100
200
300
Time [s]
Chest HR
Wrist HR

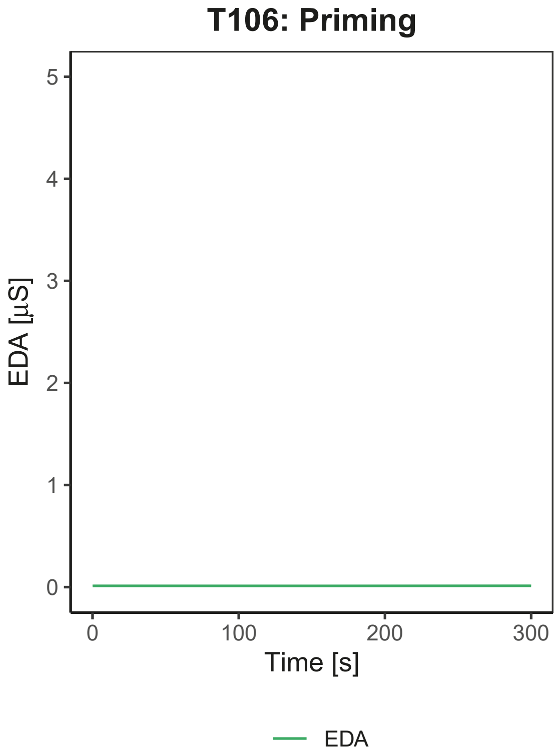
T106: Priming
EDA [µS]
5
4
3
2
1
0
0
100
200
300
Time [s]
EDA

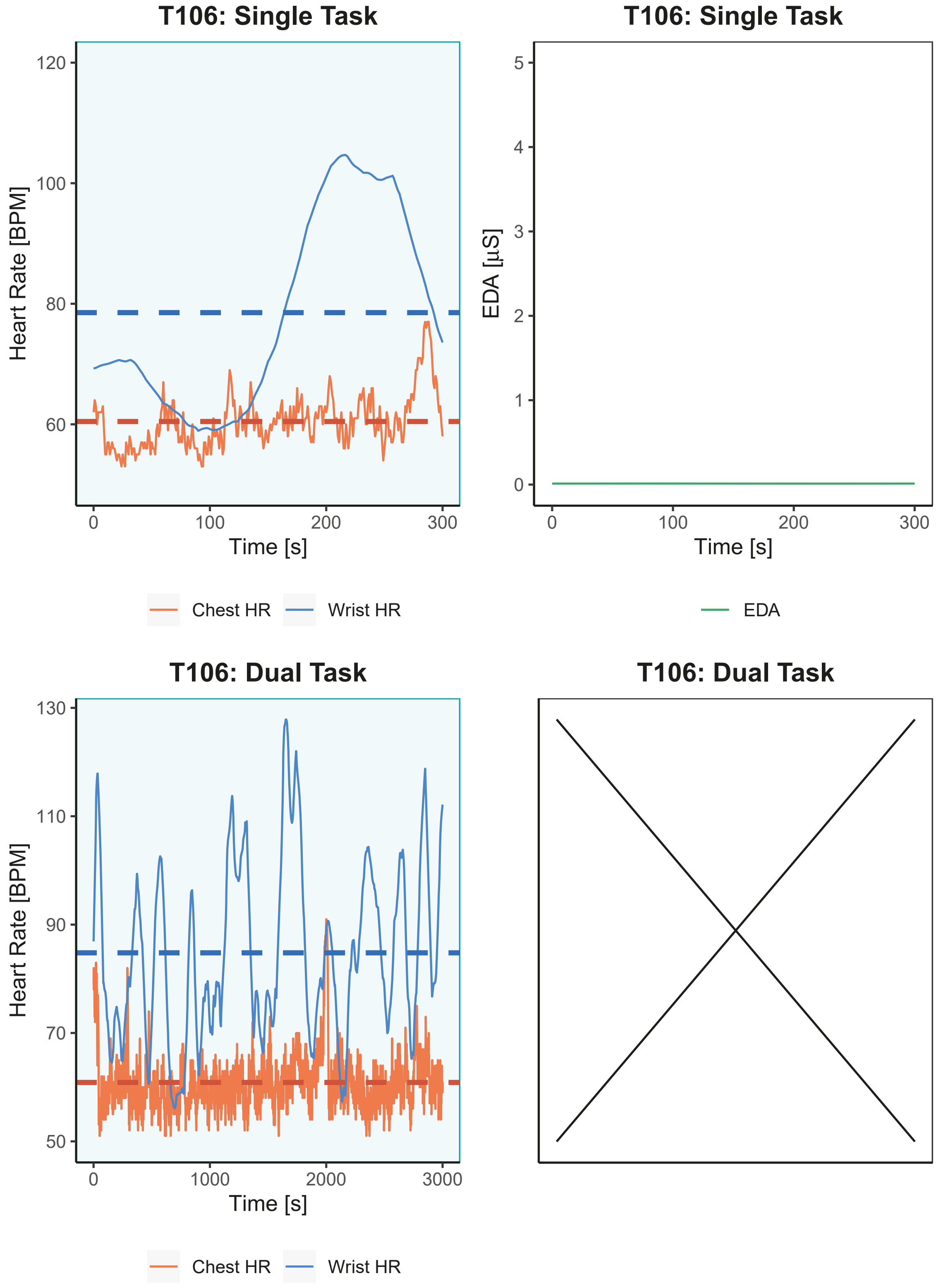
T106: Single Task
Heart Rate [BPM]
120
100
80
60
0
100
200
300
Time [s]
Chest HR
Wrist HR
T106: Single Task
EDA [µS]
5
4
3
2
1
0
0
100
200
300
Time [s]
EDA
T106: Dual Task
Heart Rate [BPM]
130
110
90
70
50
0
1000
2000
3000
Time [s]
Chest HR
Wrist HR
T106: Dual Task

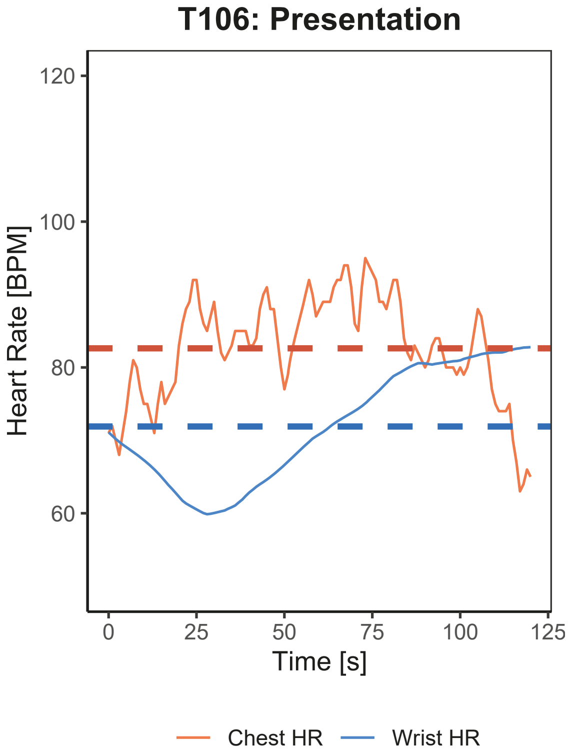
T106: Presentation
120
100
80
60
Heart Rate [BPM]
0
25
50
75
100
125
Time [s]
Chest HR
Wrist HR

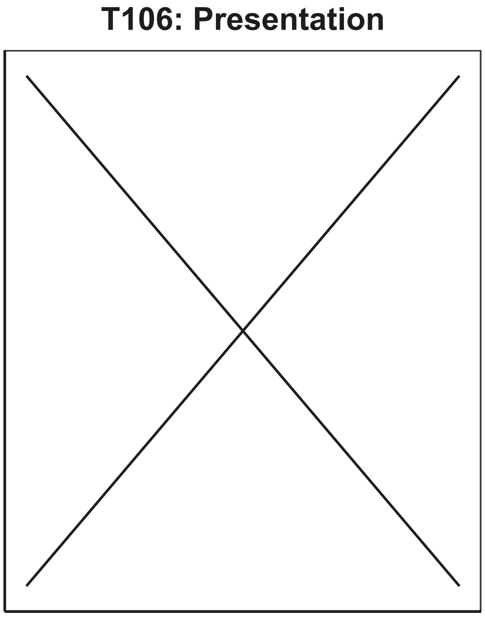
T106: Presentation

--

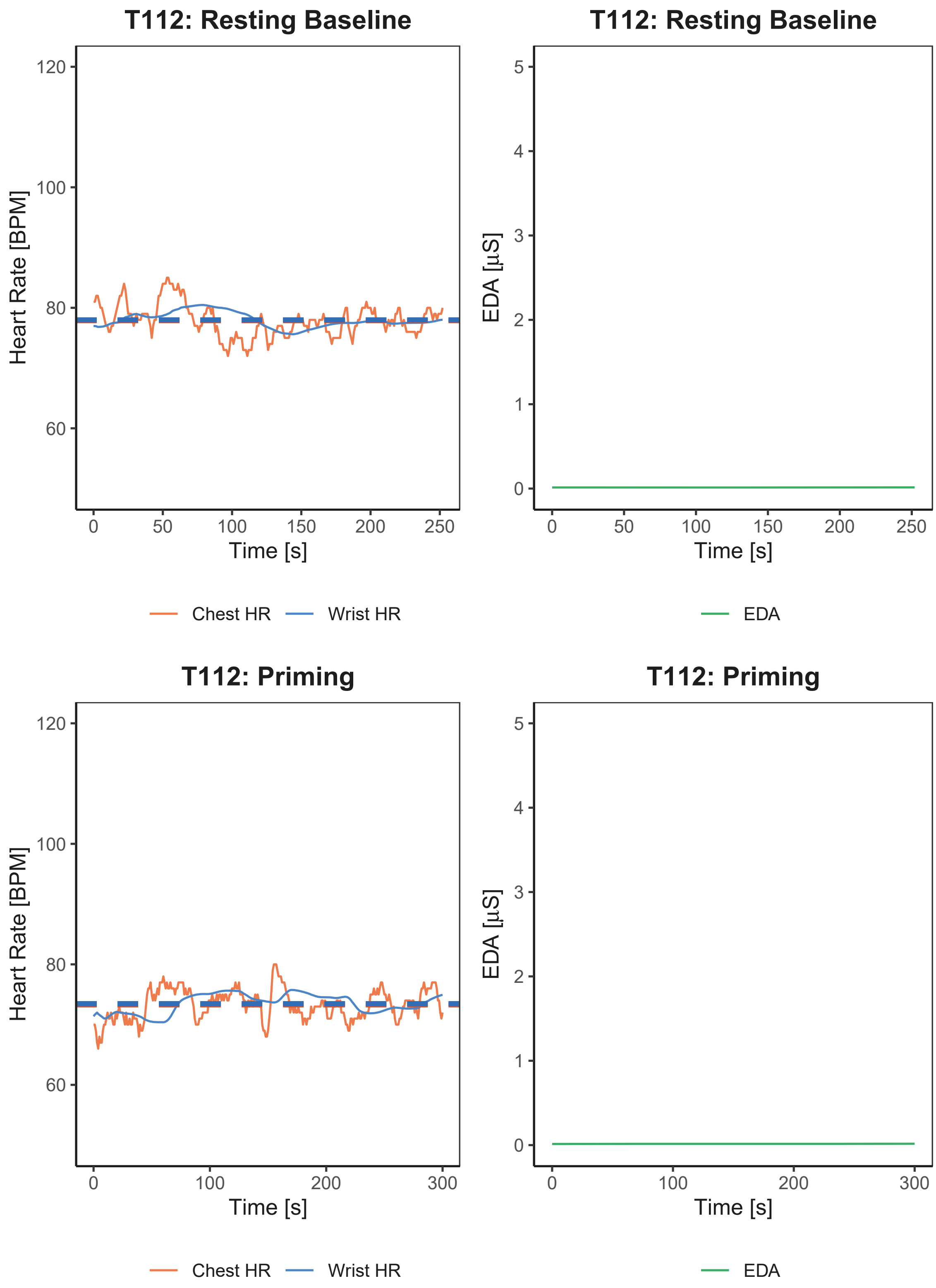
T112: Resting Baseline
Heart Rate [BPM]
120
100
80
60
0
50
100
150
200
250
Time [s]
Chest HR
Wrist HR
T112: Resting Baseline
EDA [µS]
5
4
3
2
1
0
0
50
100
150
200
250
Time [s]
EDA
T112: Priming
Heart Rate [BPM]
120
100
80
60
0
100
200
300
Time [s]
Chest HR
Wrist HR
T112: Priming
EDA [µS]
5
4
3
2
1
0
0
100
200
300
Time [s]
EDA

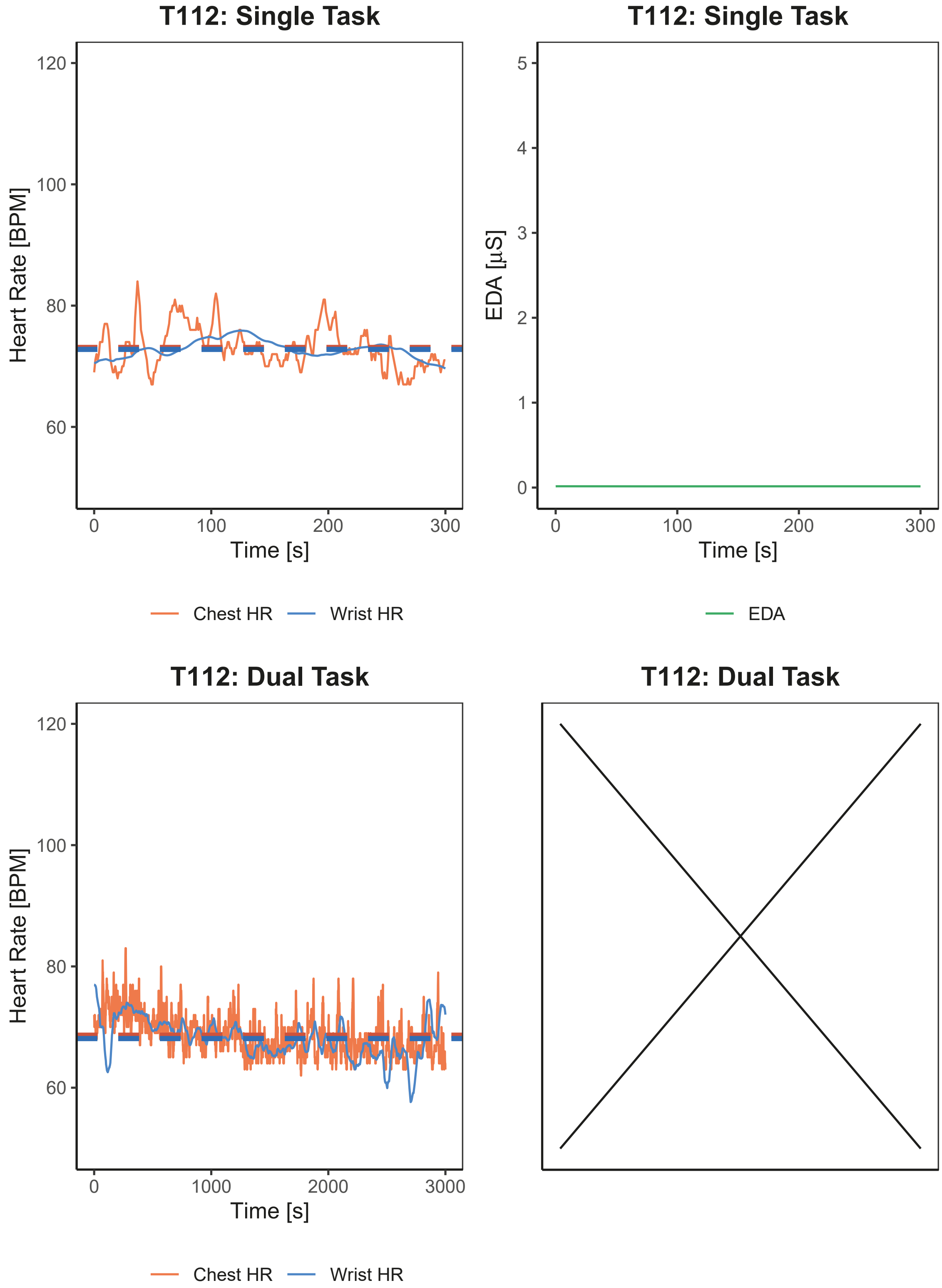
T112: Single Task
Heart Rate [BPM]
120
100
80
60
0
100
200
300
Time [s]
Chest HR
Wrist HR
T112: Single Task
EDA [µS]
5
4
3
2
1
0
0
100
200
300
Time [s]
EDA
T112: Dual Task
Heart Rate [BPM]
120
100
80
60
0
1000
2000
3000
Time [s]
Chest HR
Wrist HR
T112: Dual Task

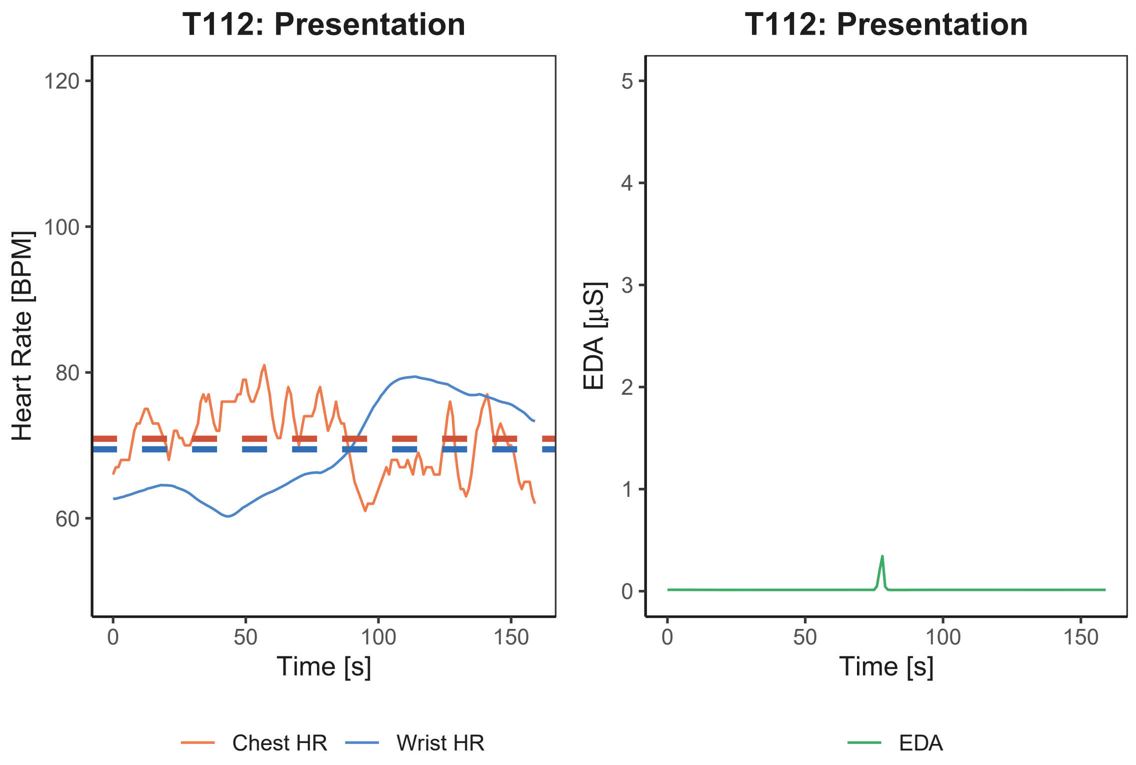
T112: Presentation
Heart Rate [BPM]
120
100
80
60
0
50
100
150
Time [s]
Chest HR
Wrist HR
T112: Presentation
EDA [µS]
5
4
3
2
1
0
0
50
100
150
Time [s]
EDA

--

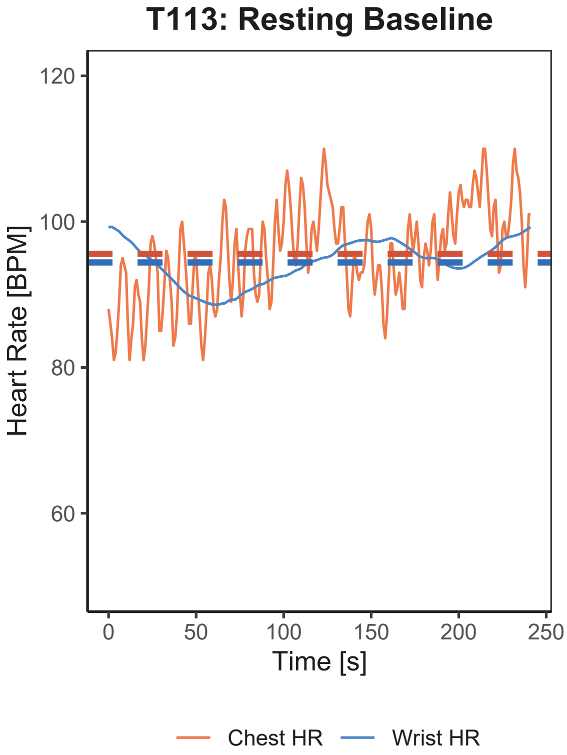
T113: Resting Baseline
Heart Rate [BPM]
120
100
80
60
0
50
100
150
200
250
Time [s]
Chest HR
Wrist HR

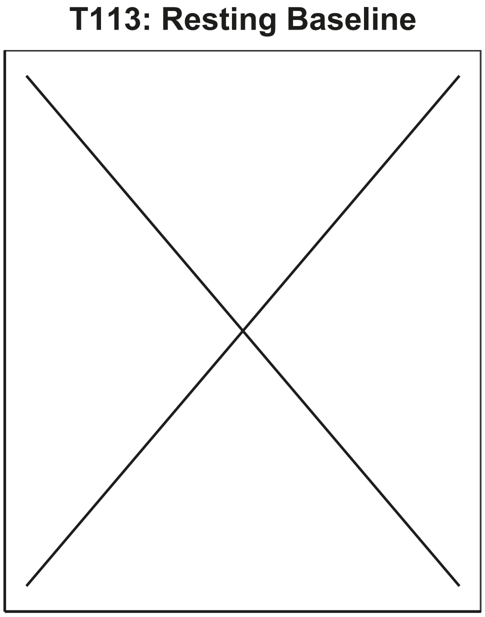
T113: Resting Baseline

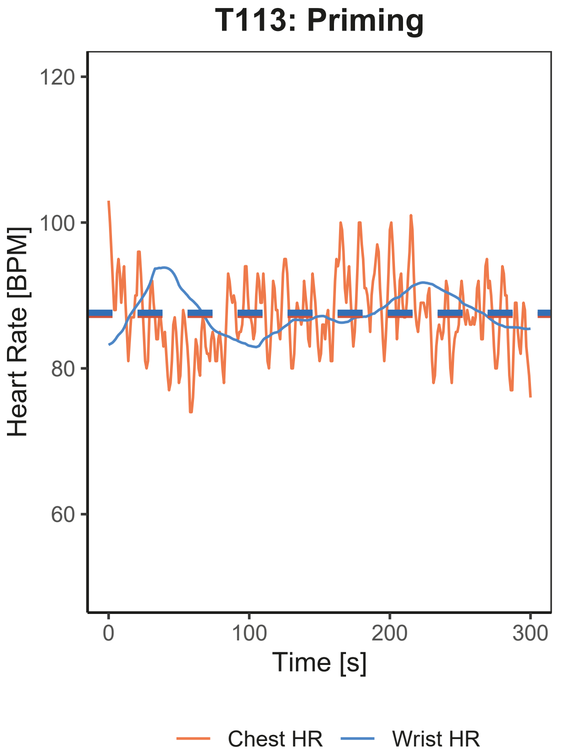
T113: Priming
Heart Rate [BPM]
120
100
80
60
0
100
200
300
Time [s]
Chest HR
Wrist HR

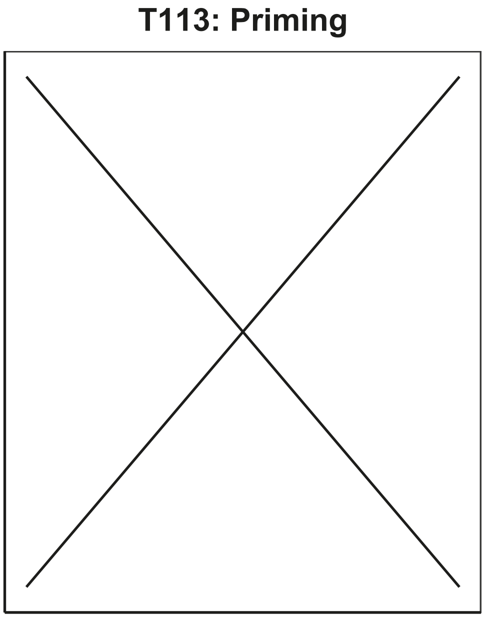
T113: Priming

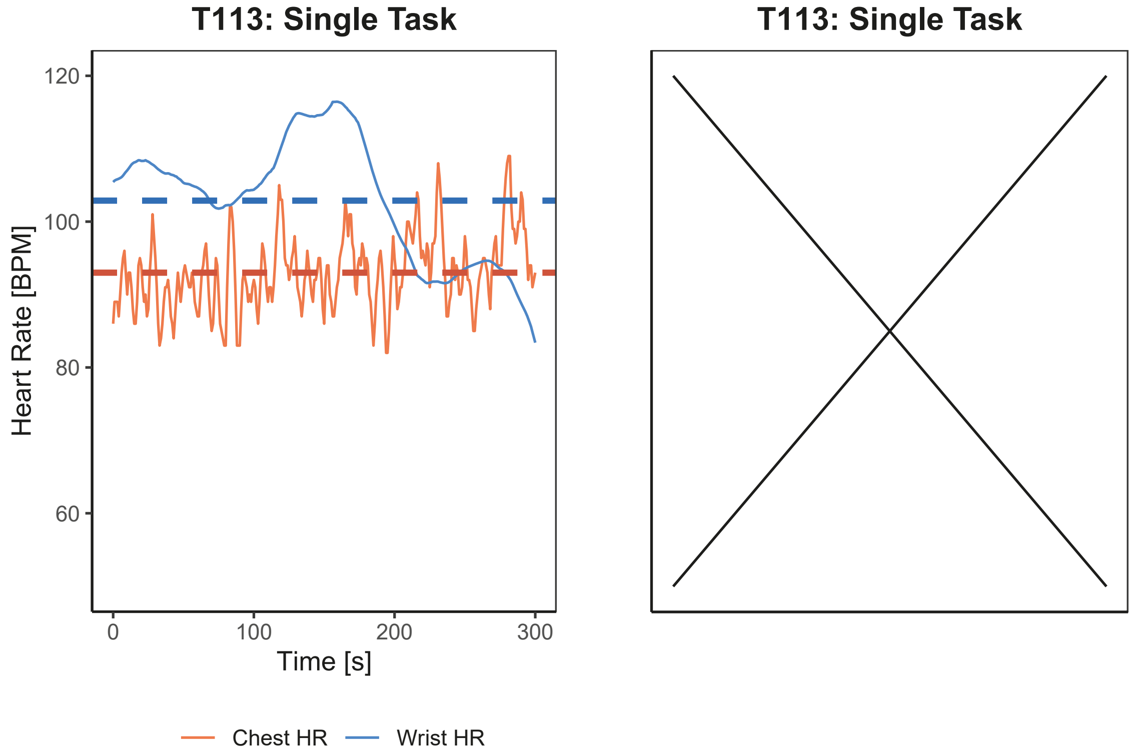

T113: Single Task
T113: Single Task
Heart Rate [BPM]
120
100
80
60
0
100
200
300
Time [s]
Chest HR
Wrist HR

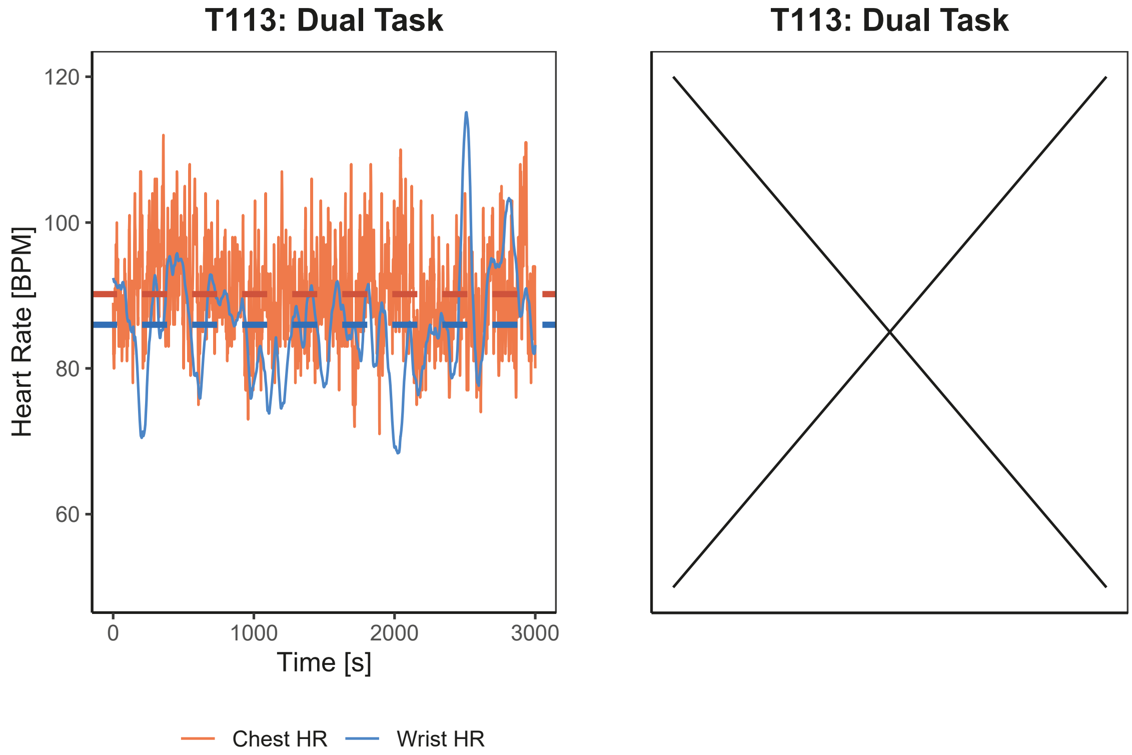

T113: Dual Task
T113: Dual Task
Heart Rate [BPM]
120
100
80
60
0
1000
2000
3000
Time [s]
Chest HR
Wrist HR

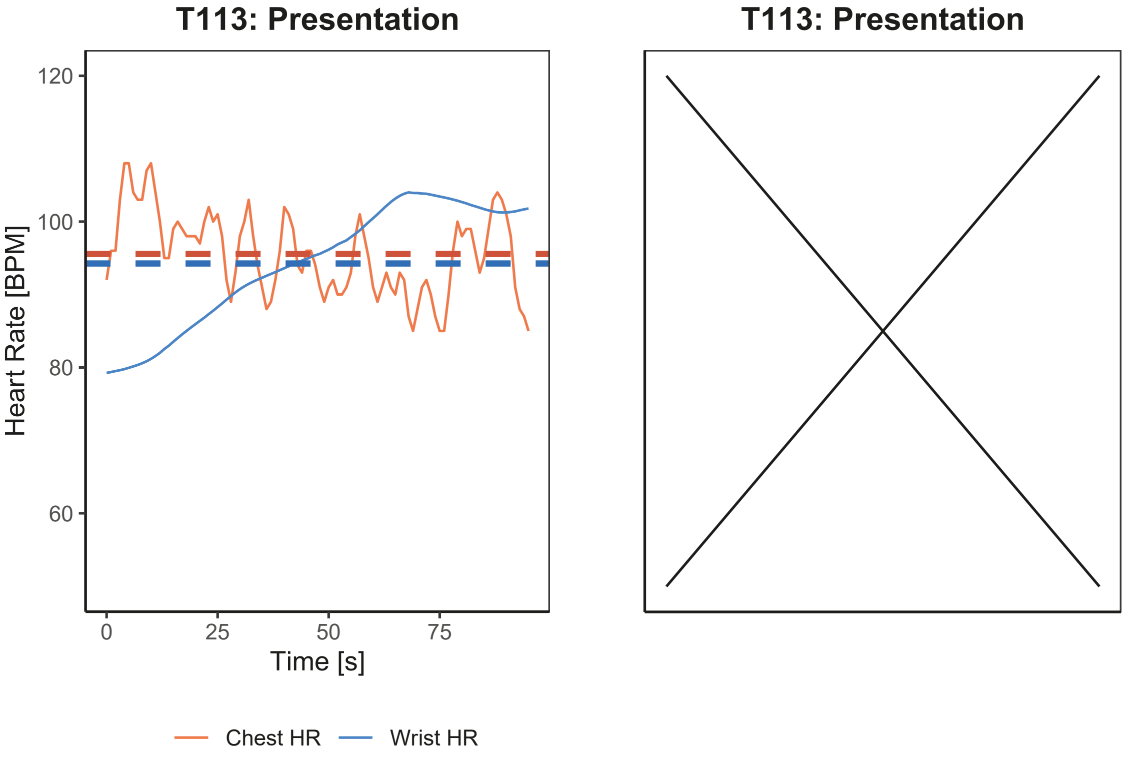
T113: Presentation
T113: Presentation
Heart Rate [BPM]
120
100
80
60
0
25
50
75
Time [s]
Chest HR
Wrist HR

```
## -------------------------------------------------------------------------------- ##
```

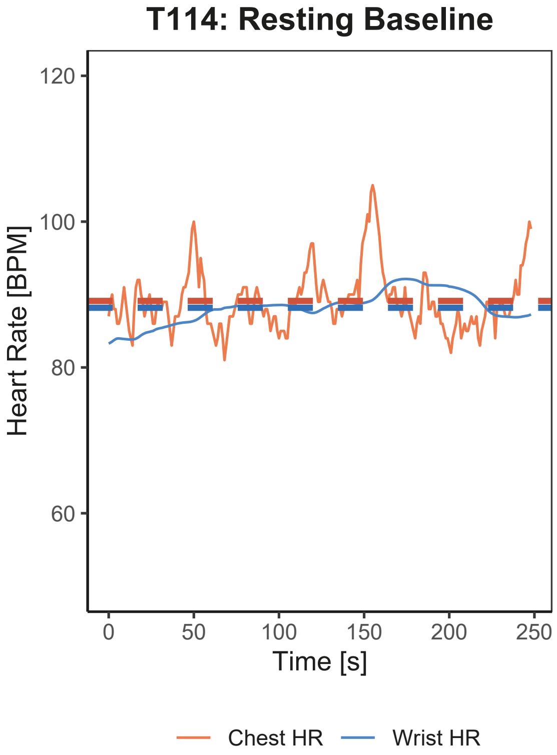
T114: Resting Baseline
Heart Rate [BPM]
120
100
80
60
0
50
100
150
200
250
Time [s]
Chest HR
Wrist HR

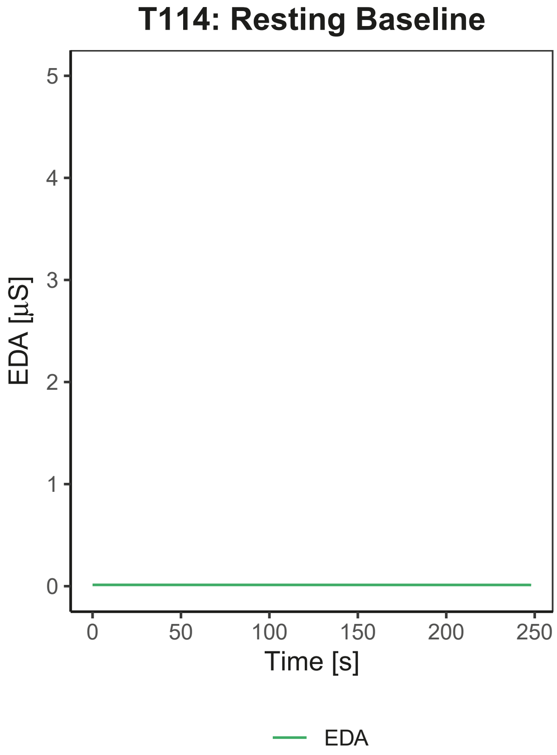
T114: Resting Baseline
EDA [µS]
5
4
3
2
1
0
0
50
100
150
200
250
Time [s]
EDA

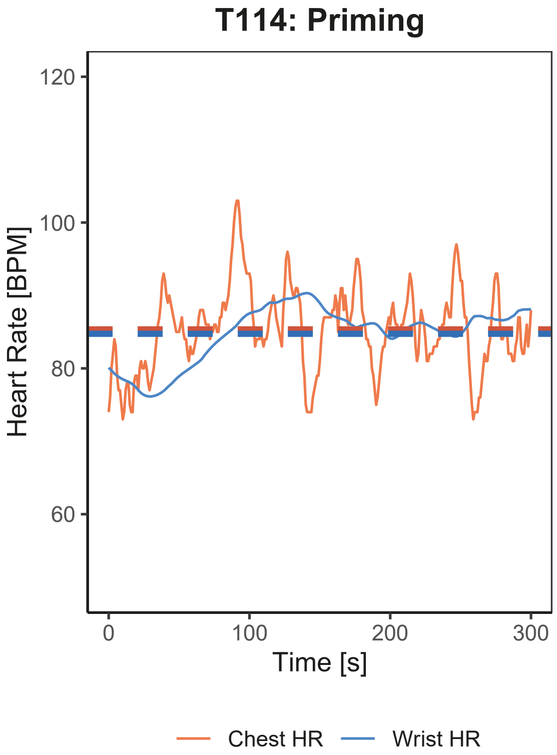
T114: Priming
Heart Rate [BPM]
120
100
80
60
0
100
200
300
Time [s]
Chest HR
Wrist HR

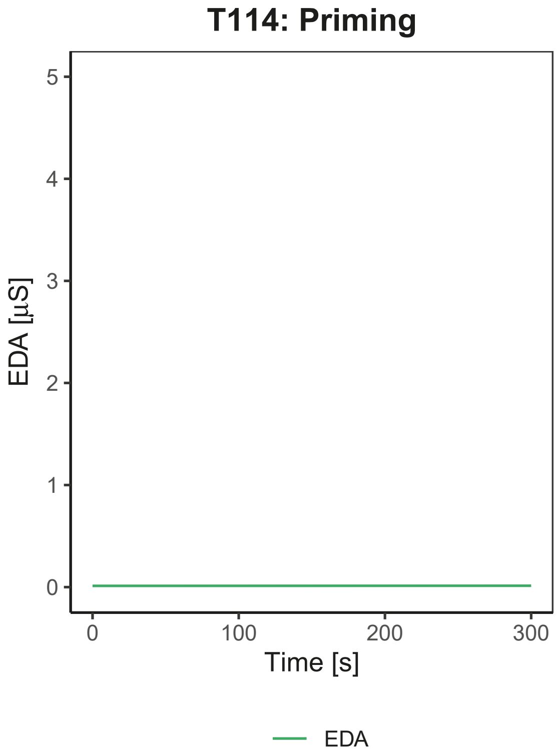
T114: Priming
EDA [µS]
5
4
3
2
1
0
0
100
200
300
Time [s]
EDA

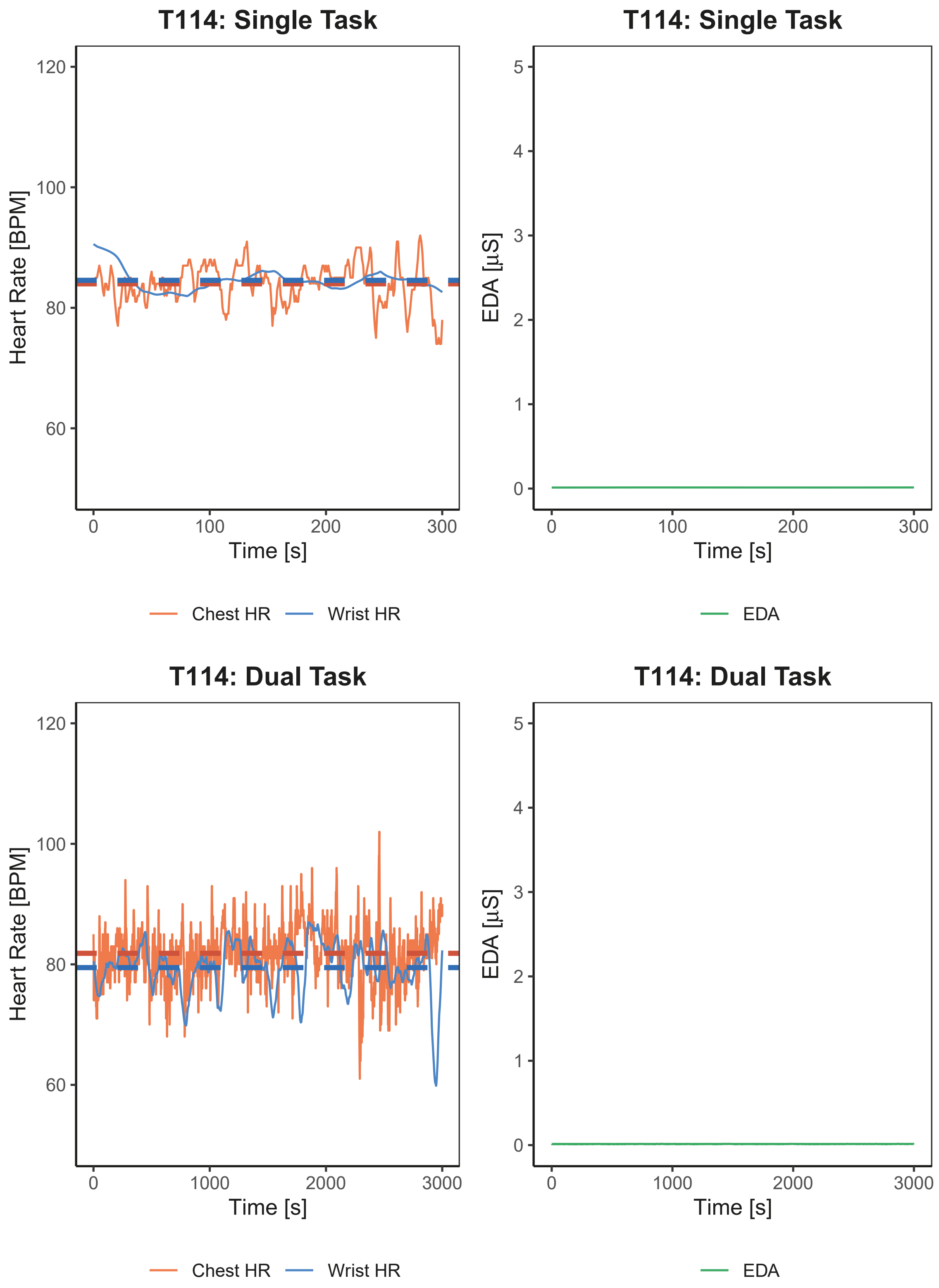
T114: Single Task
Heart Rate [BPM]
Time [s]
Chest HR
Wrist HR
T114: Single Task
EDA [µS]
Time [s]
EDA
T114: Dual Task
Heart Rate [BPM]
Time [s]
Chest HR
Wrist HR
T114: Dual Task
EDA [µS]
Time [s]
EDA

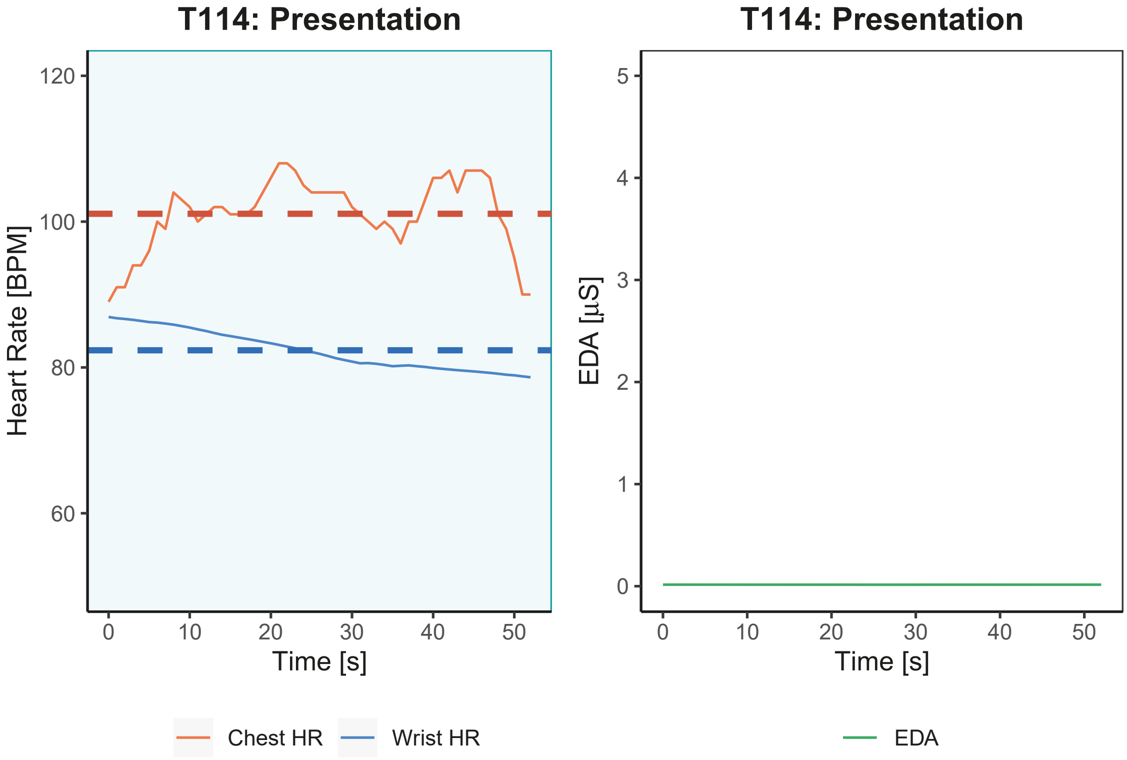

T114: Presentation
Heart Rate [BPM]
120
100
80
60
0
10
20
30
40
50
Time [s]
T114: Presentation
EDA [µS]
5
4
3
2
1
0
0
10
20
30
40
50
Time [s]
Chest HR
Wrist HR
EDA

--

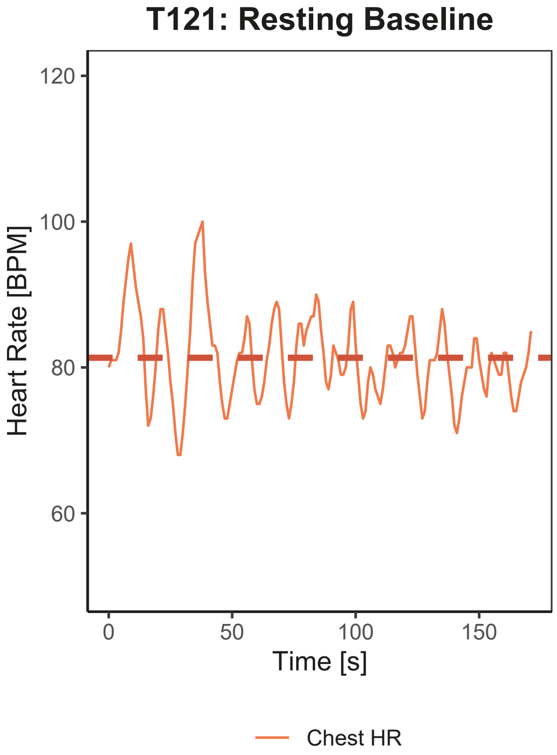
T121: Resting Baseline
120
100
80
60
Heart Rate [BPM]
0
50
100
150
Time [s]
Chest HR

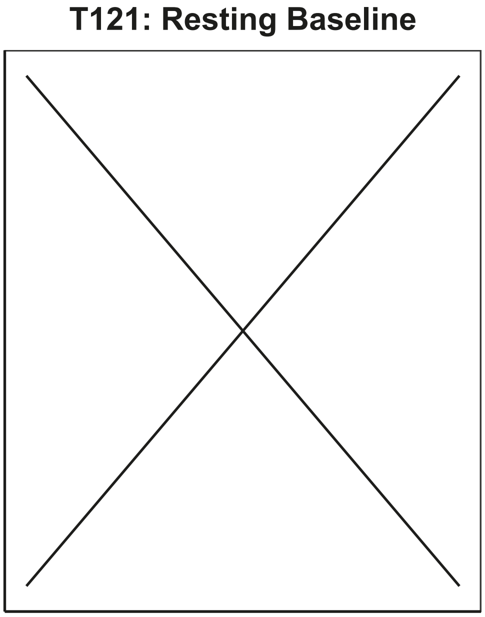
T121: Resting Baseline

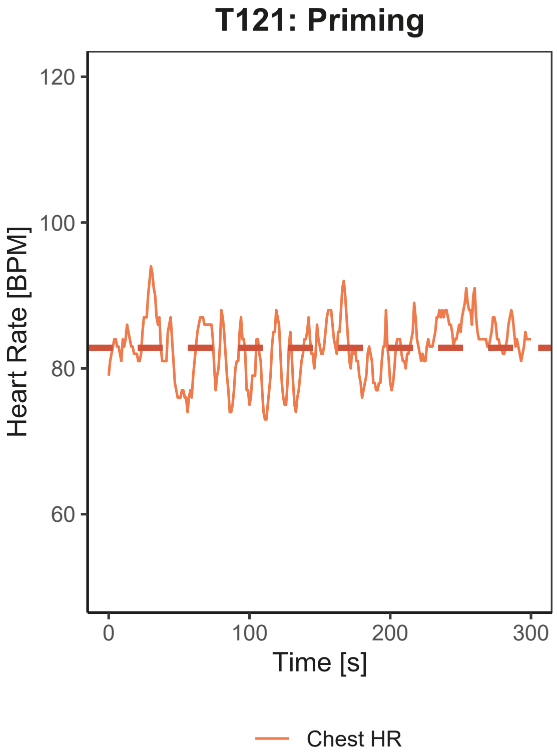
T121: Priming
120
100
80
60
Heart Rate [BPM]
0
100
200
300
Time [s]
Chest HR

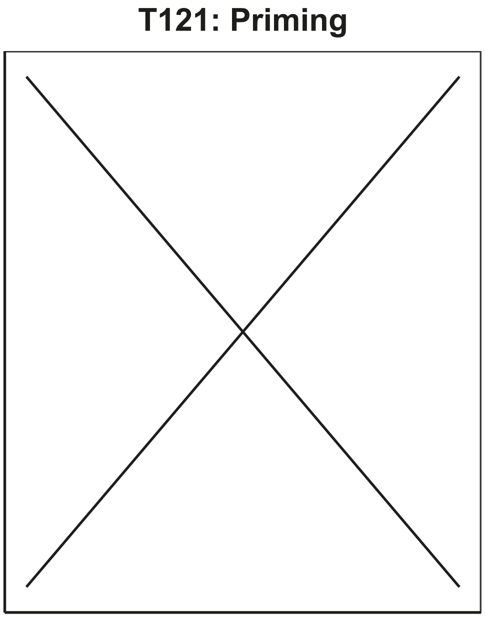
T121: Priming

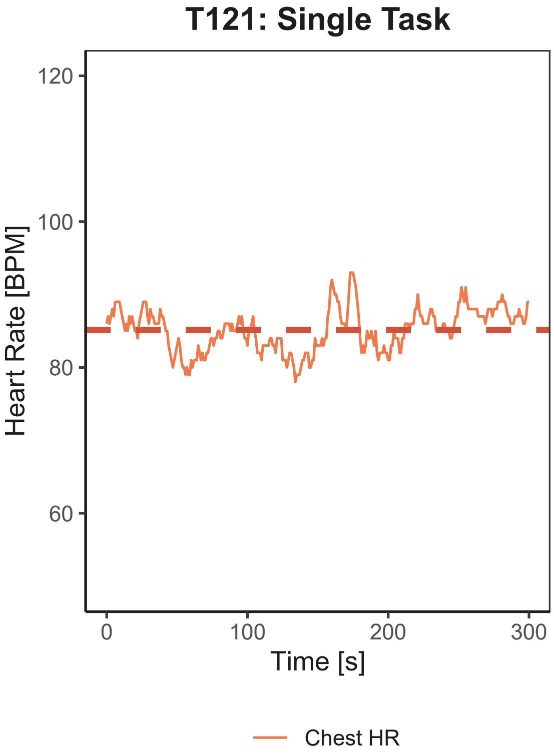
T121: Single Task
Heart Rate [BPM]
120
100
80
60
0
100
200
300
Time [s]
Chest HR

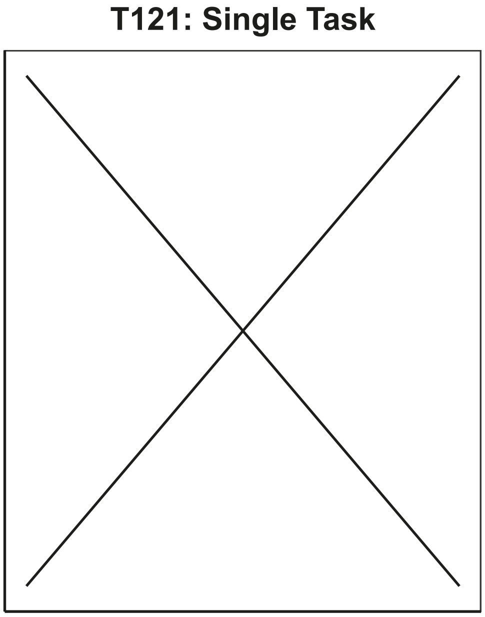
T121: Single Task

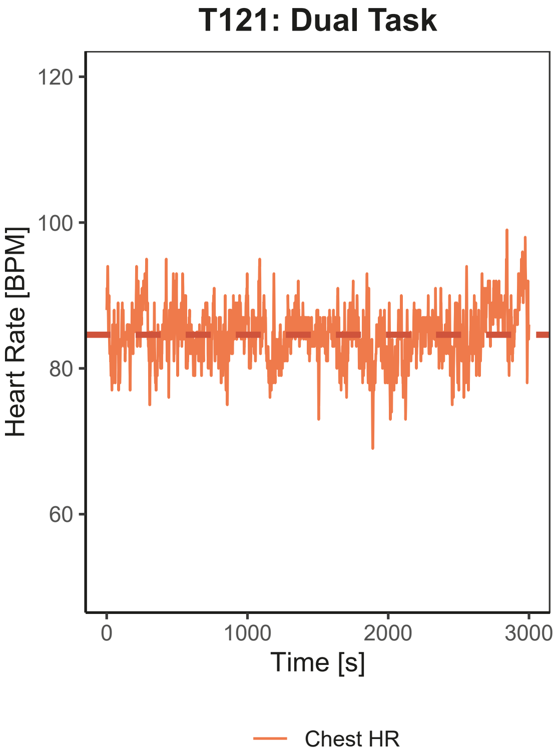
T121: Dual Task
Heart Rate [BPM]
120
100
80
60
0
1000
2000
3000
Time [s]
Chest HR

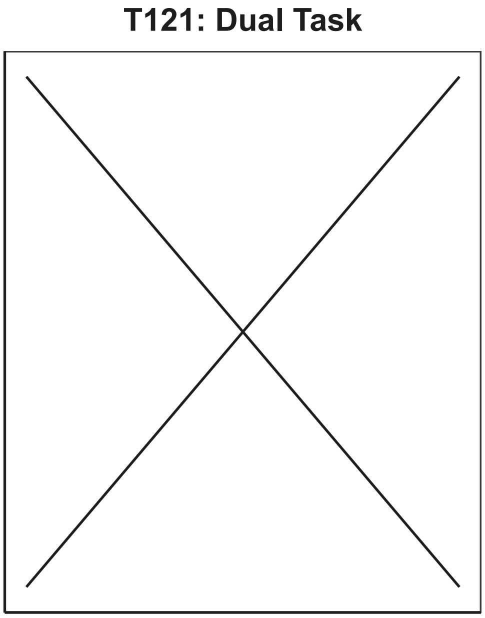
T121: Dual Task

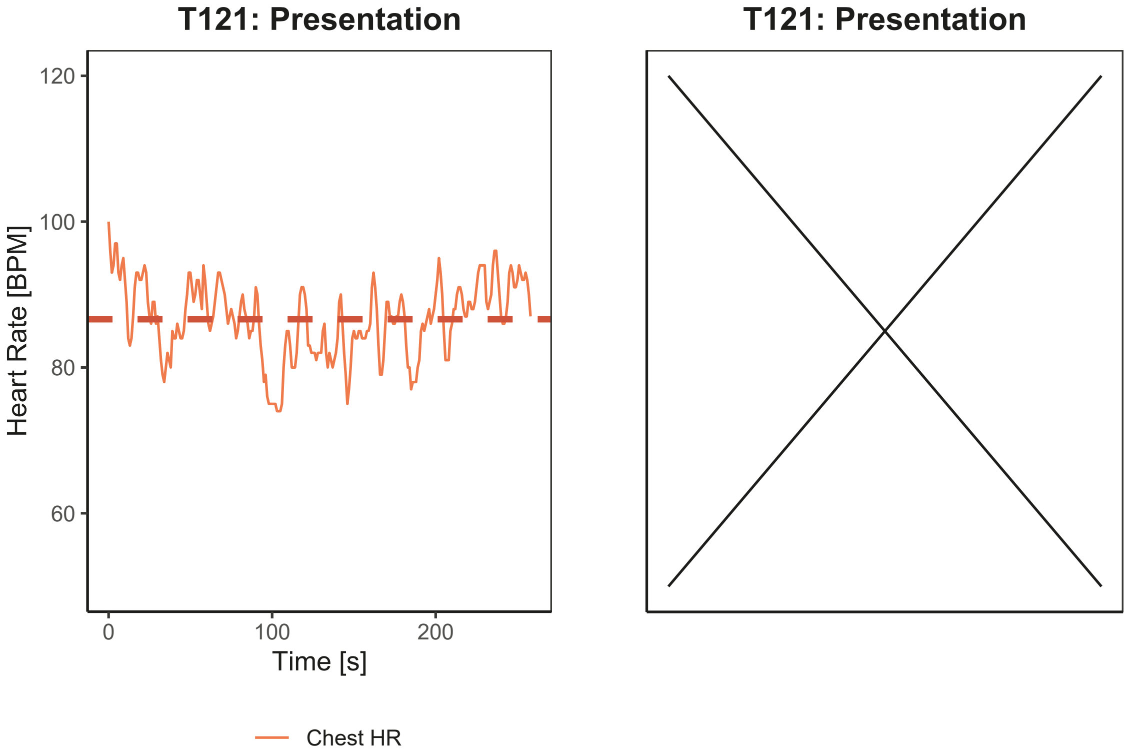
T121: Presentation
T121: Presentation
Heart Rate [BPM]
120
100
80
60
0
100
200
Time [s]
Chest HR

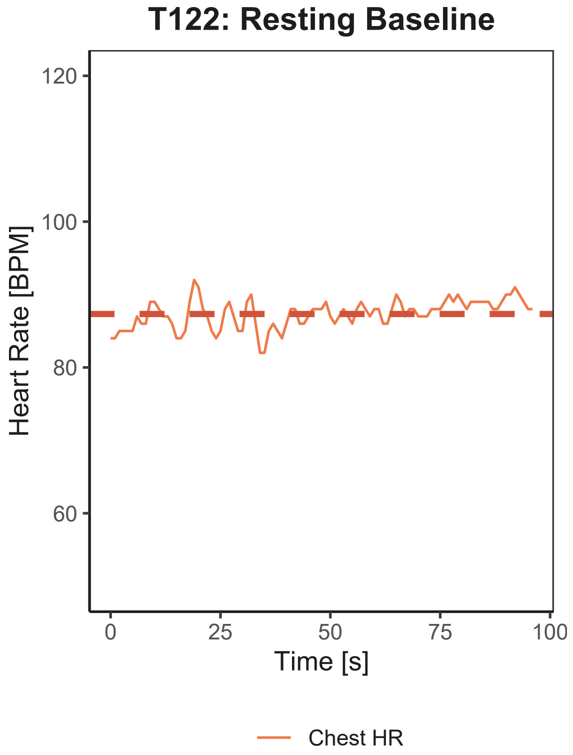
T122: Resting Baseline
Heart Rate [BPM]
120
100
80
60
0
25
50
75
100
Time [s]
Chest HR

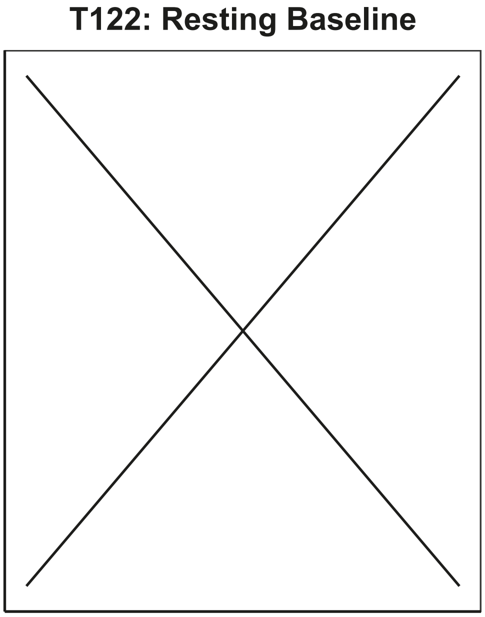
T122: Resting Baseline

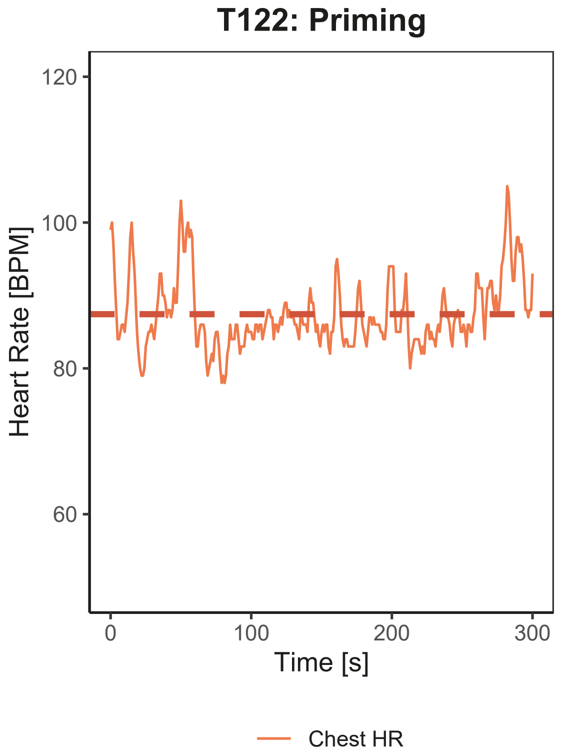
T122: Priming
Heart Rate [BPM]
120
100
80
60
0
100
200
300
Time [s]
Chest HR

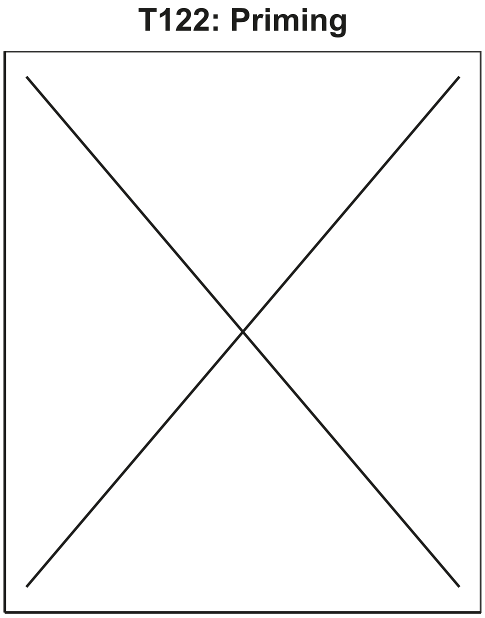
T122: Priming

T122: Single Task

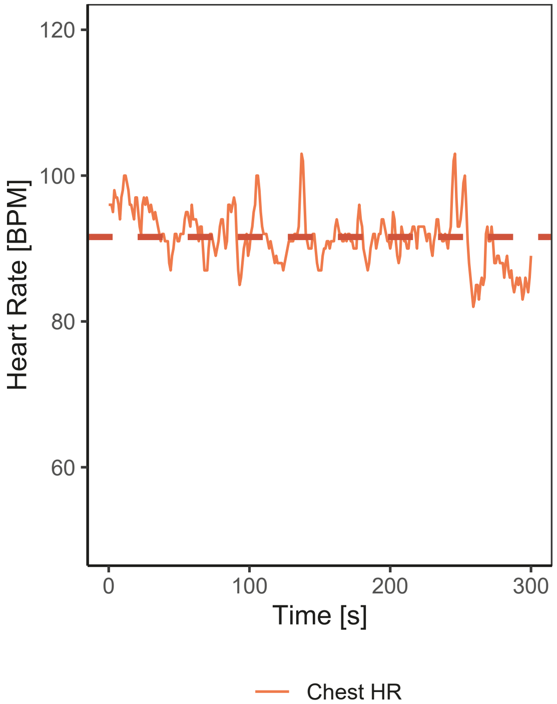

T122: Single Task

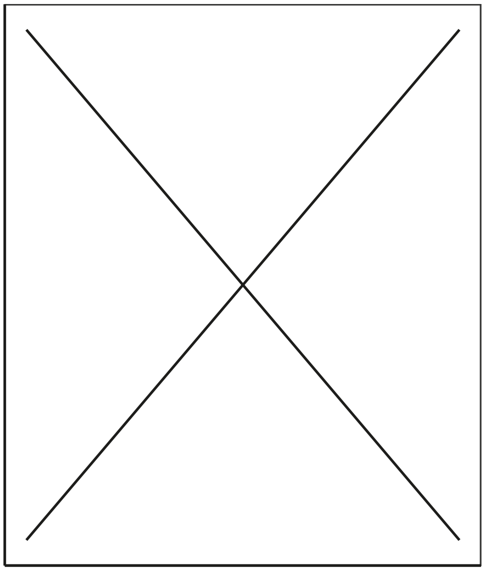

T122: Dual Task

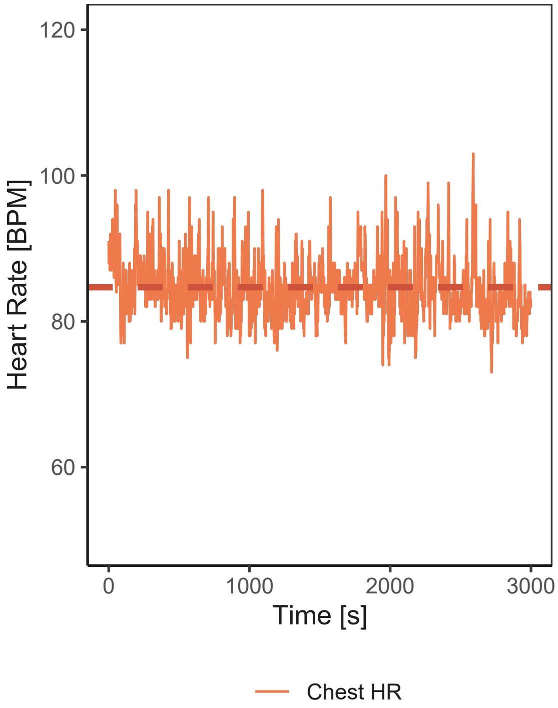

T122: Dual Task

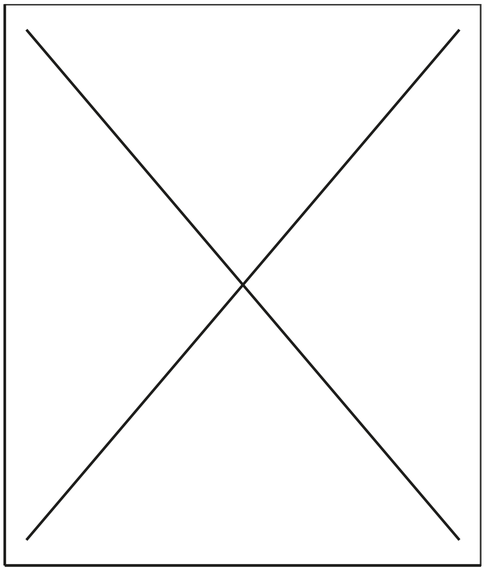

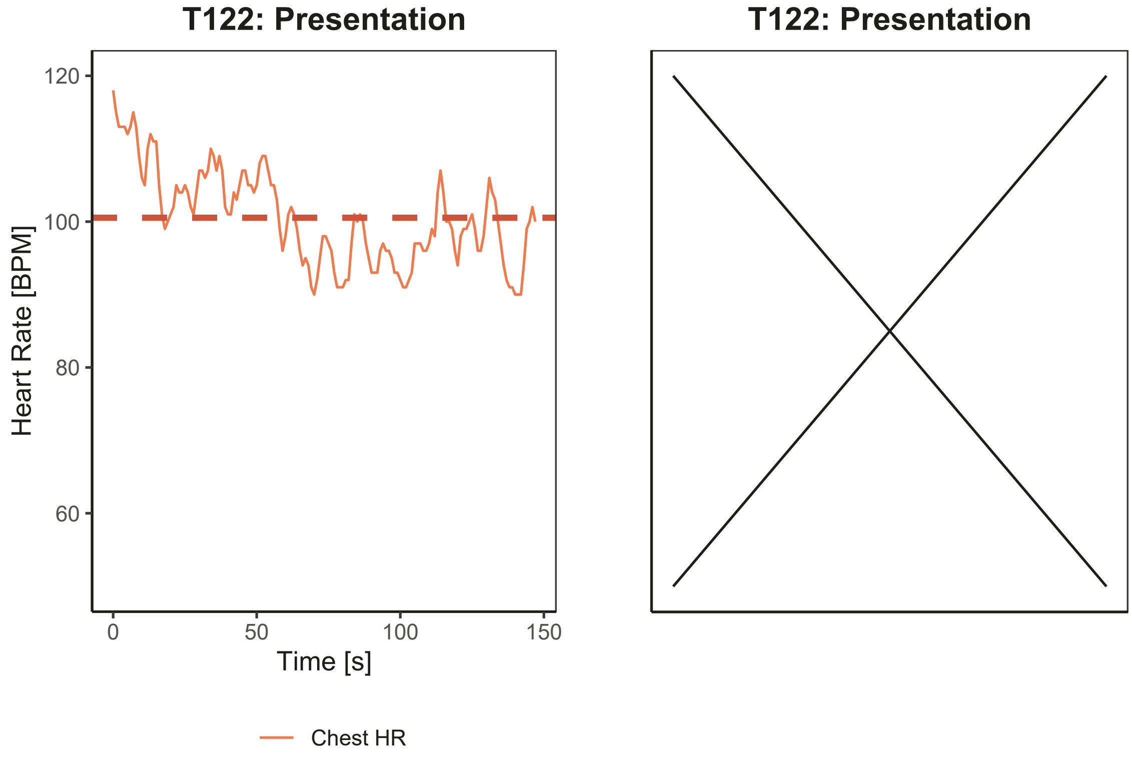
T122: Presentation
Heart Rate [BPM]
120
100
80
60
0
50
100
150
Time [s]
T122: Presentation
Chest HR

--

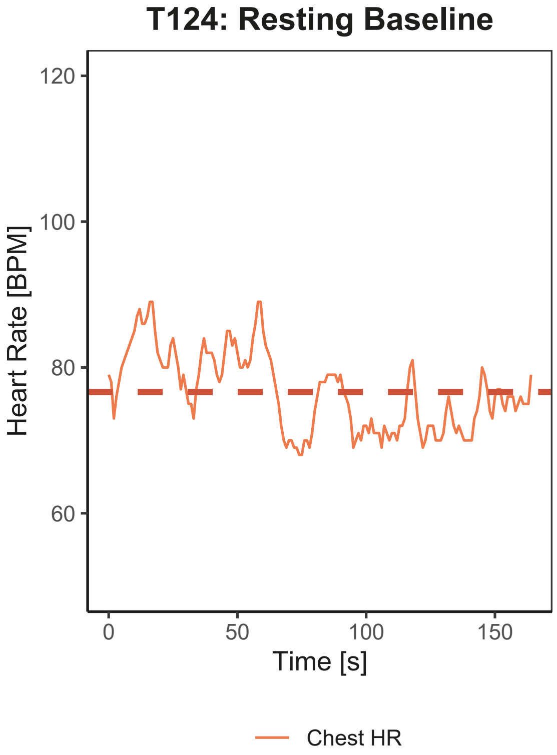
T124: Resting Baseline
Heart Rate [BPM]
120
100
80
60
0
50
100
150
Time [s]
Chest HR

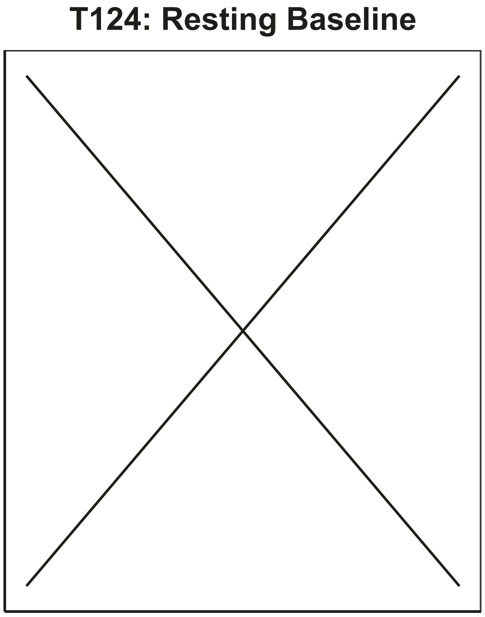
T124: Resting Baseline

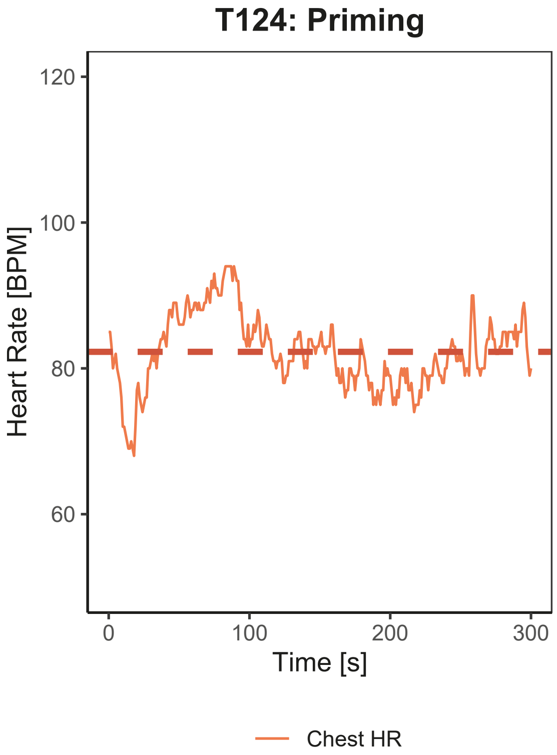
T124: Priming
Heart Rate [BPM]
120
100
80
60
0
100
200
300
Time [s]
Chest HR

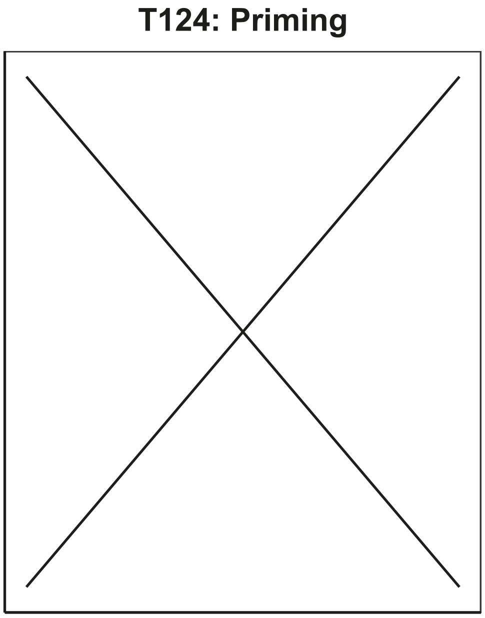
T124: Priming

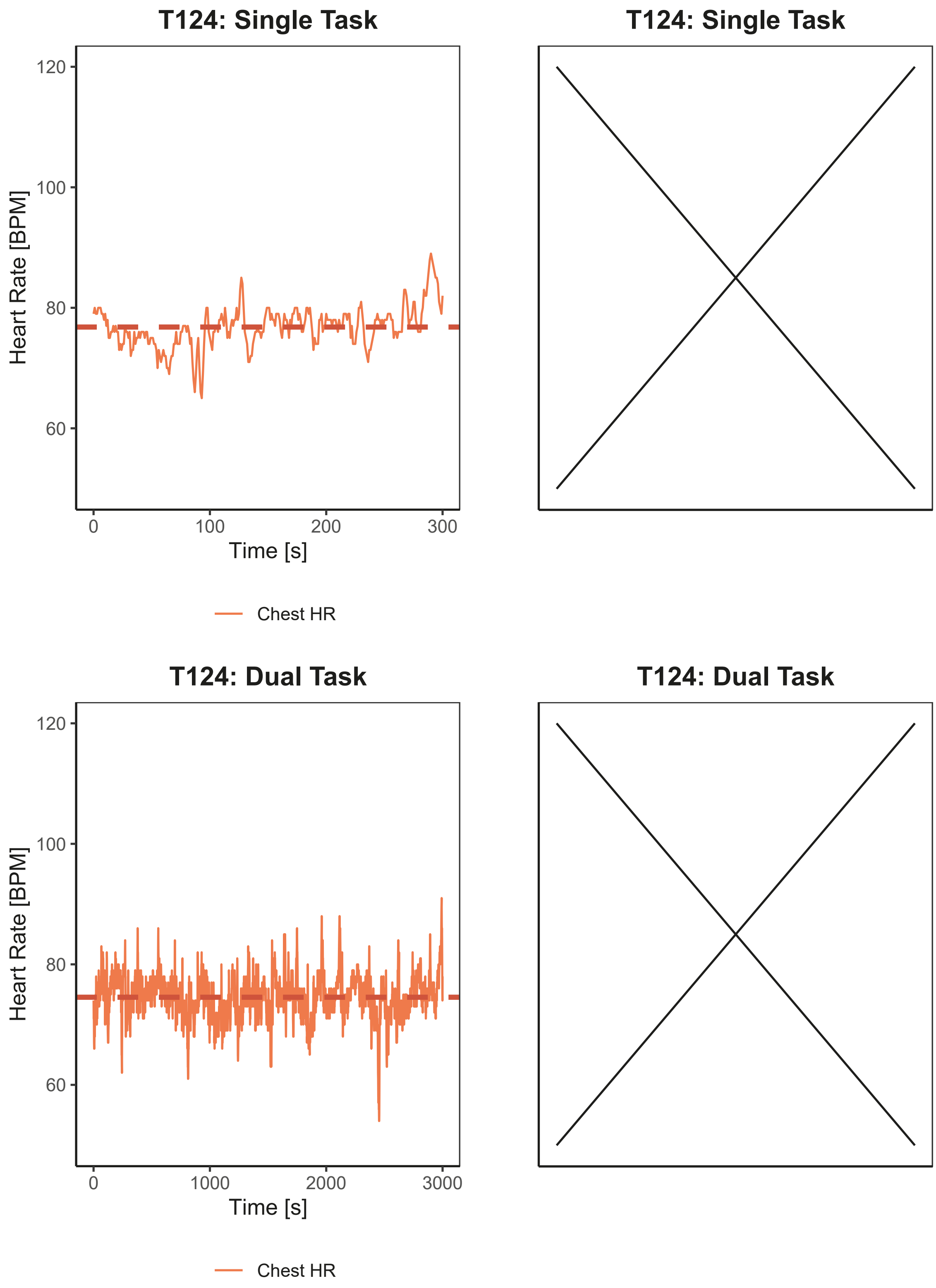
T124: Single Task
Heart Rate [BPM]
120
100
80
60
0
100
200
300
Time [s]
Chest HR
T124: Single Task
T124: Dual Task
Heart Rate [BPM]
120
100
80
60
0
1000
2000
3000
Time [s]
Chest HR
T124: Dual Task

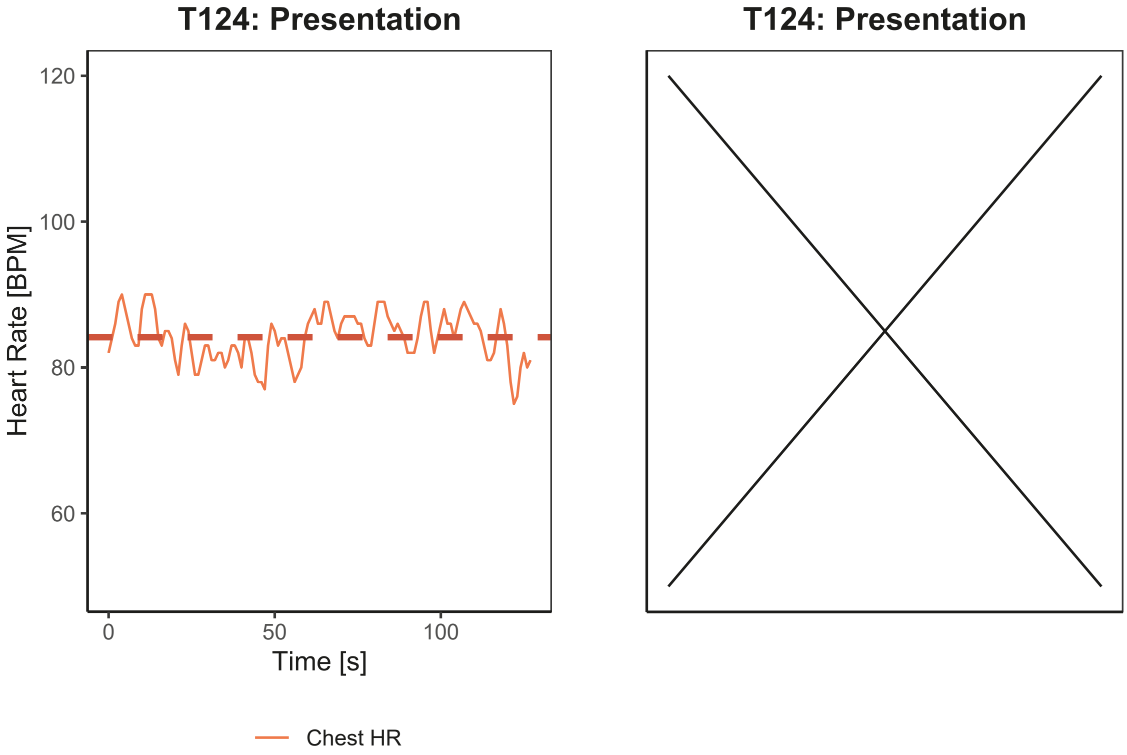
T124: Presentation
T124: Presentation
Heart Rate [BPM]
120
100
80
60
0
50
100
Time [s]
Chest HR

```
## ----------------------------------------------------------------------------- ##
```

T126: Resting Baseline

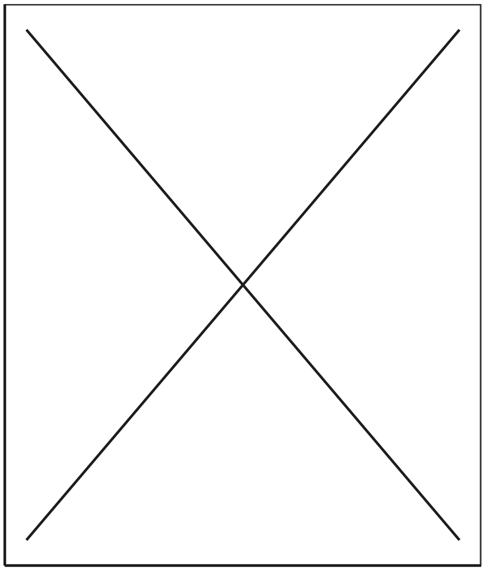

T126: Resting Baseline

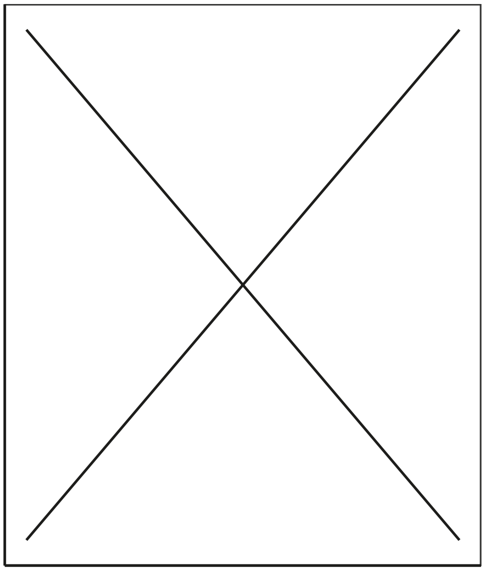

T126: Priming

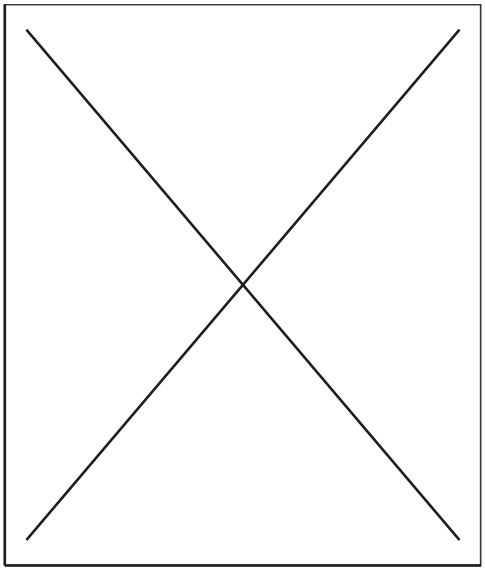

T126: Priming

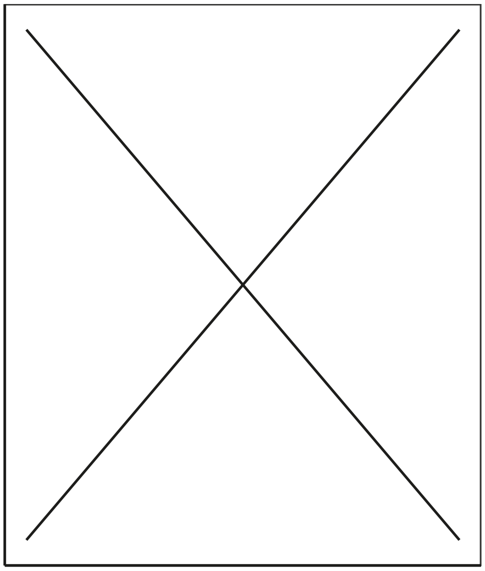

T126: Single Task

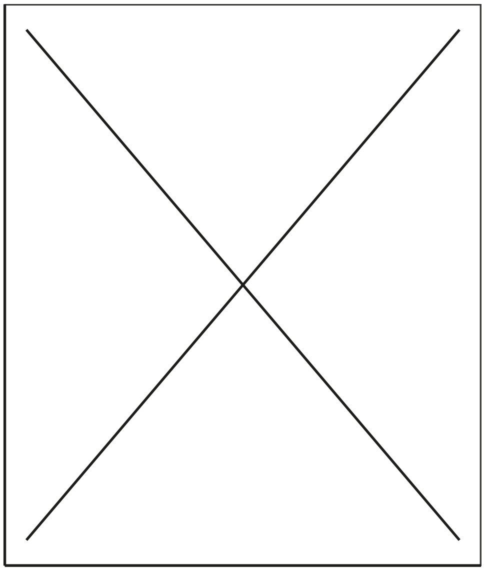

T126: Single Task

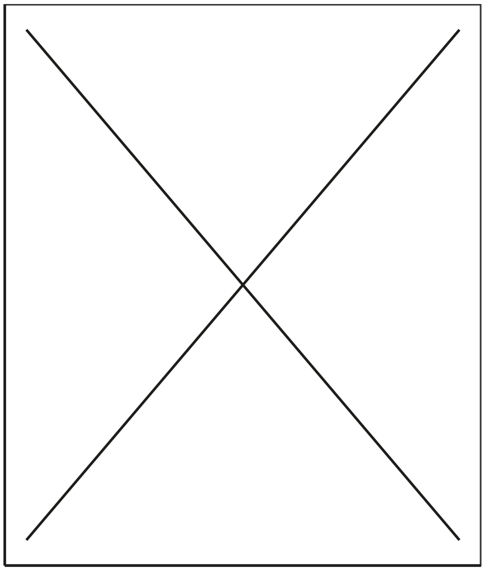

T126: Dual Task

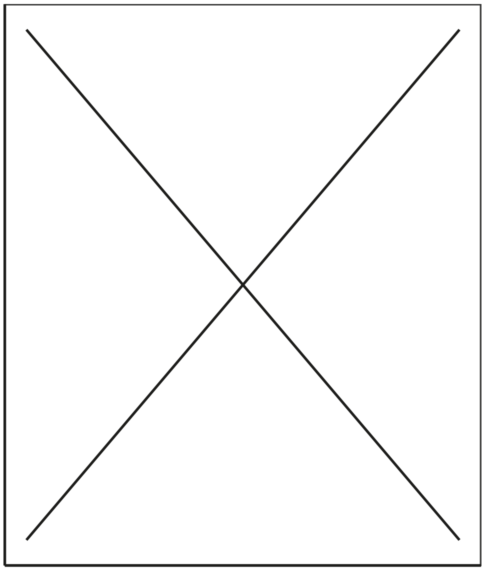

T126: Dual Task

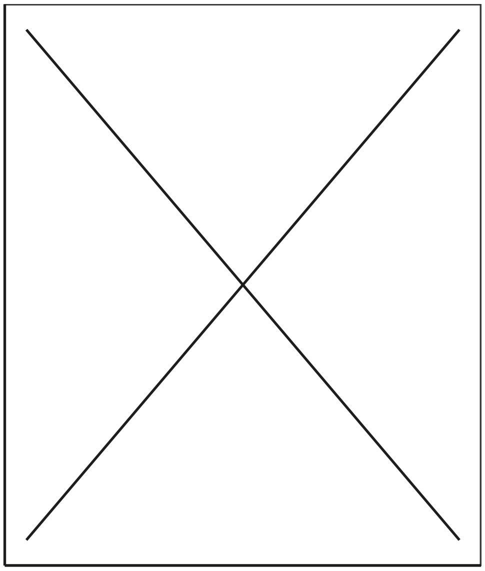

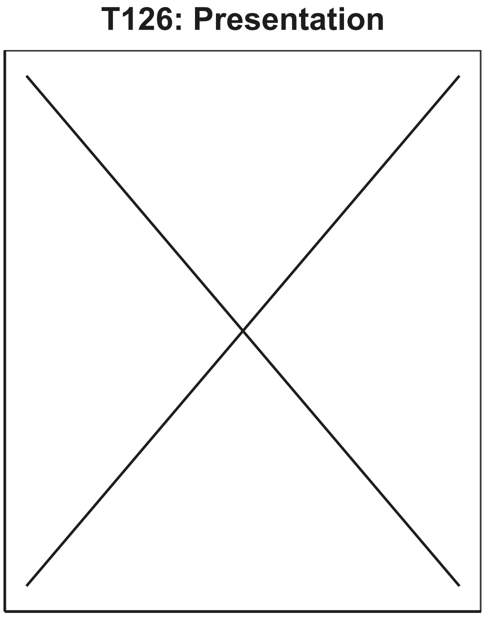
T126: Presentation

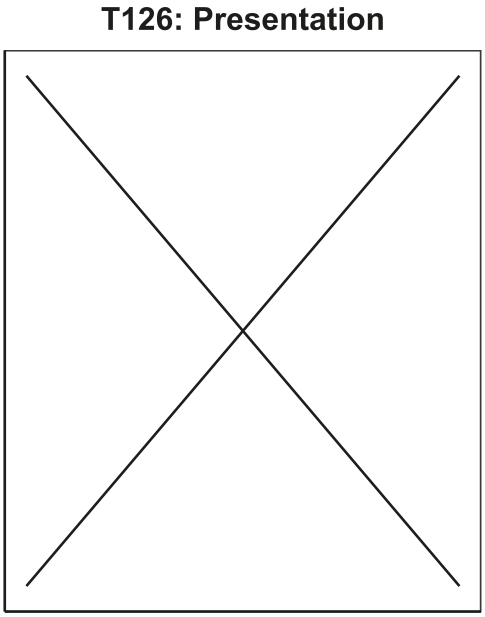
T126: Presentation

```
## ------------------------------------------------------------------------------ ##
```

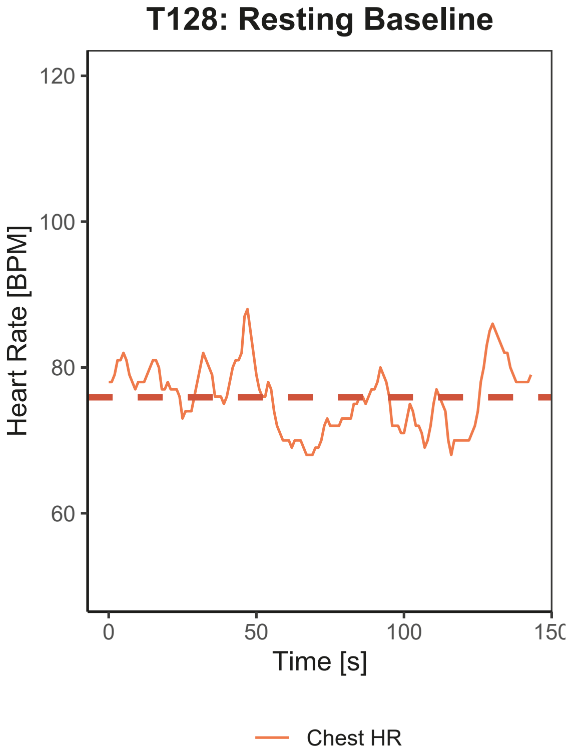
T128: Resting Baseline
Heart Rate [BPM]
120
100
80
60
0
50
100
150
Time [s]
Chest HR

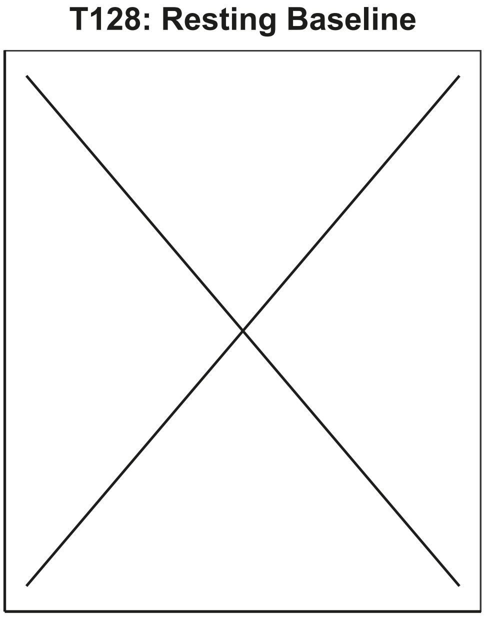
T128: Resting Baseline

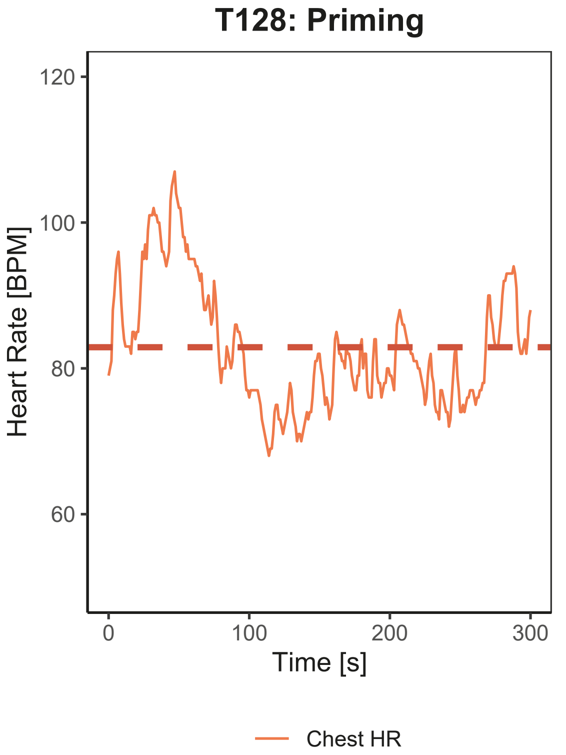
T128: Priming
Heart Rate [BPM]
120
100
80
60
0
100
200
300
Time [s]
Chest HR

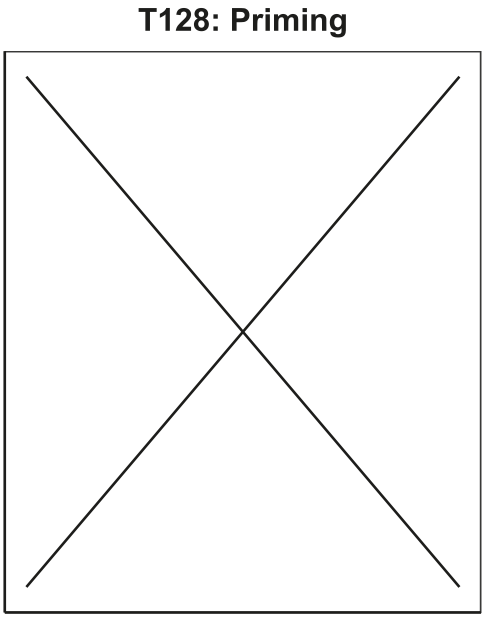
T128: Priming

T128: Single Task

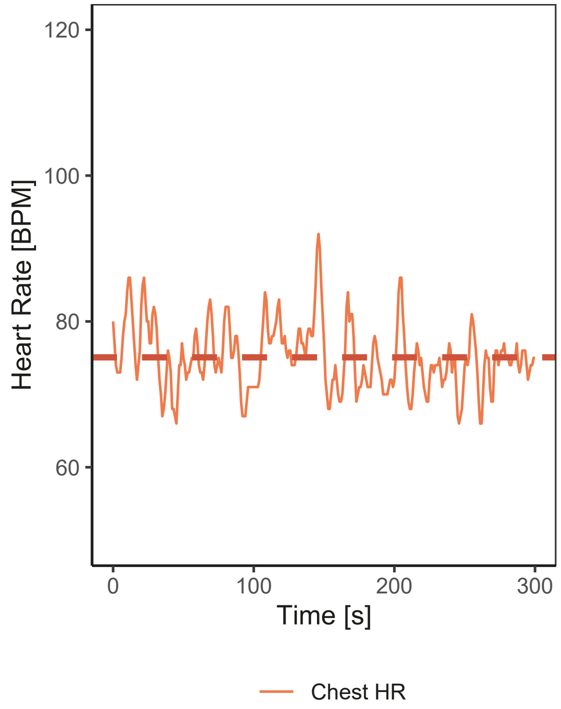

T128: Single Task

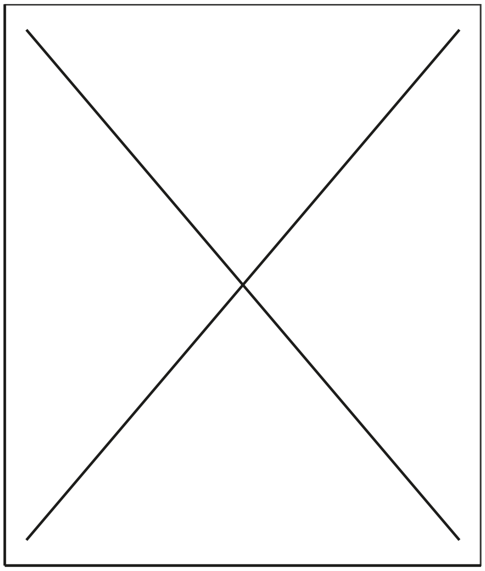

T128: Dual Task

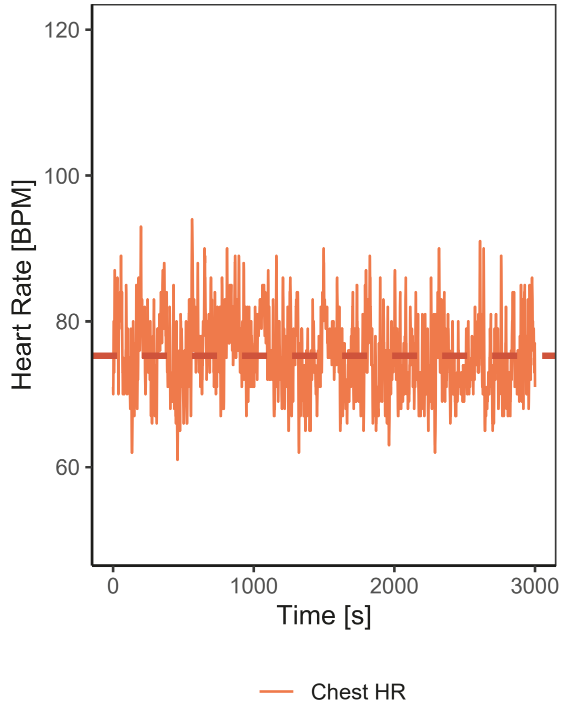

T128: Dual Task

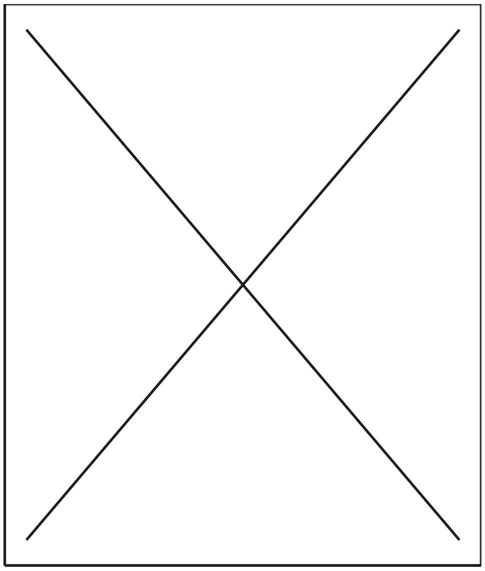

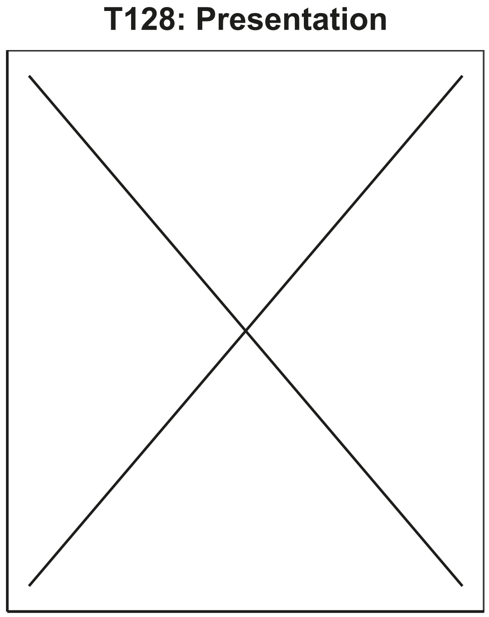
T128: Presentation

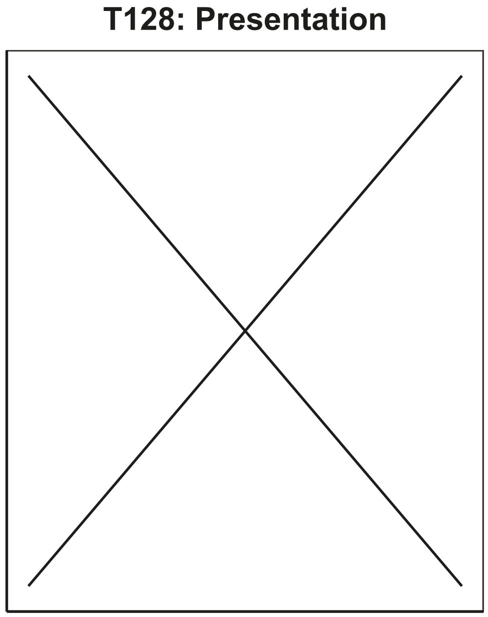
T128: Presentation

```
## ---------------------------------------------------------------------------------- ##
```

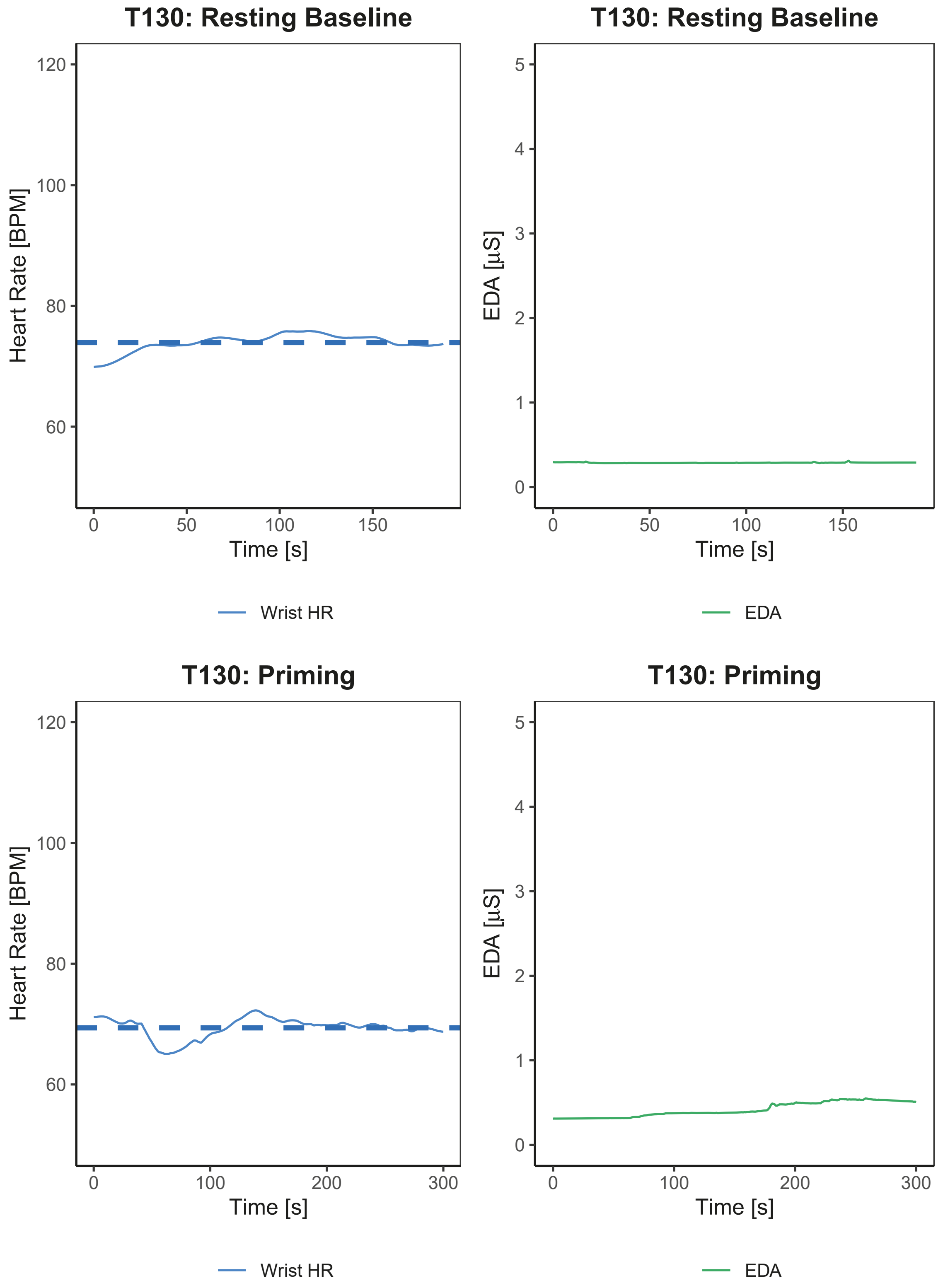
T130: Resting Baseline
Heart Rate [BPM]
Time [s]
Wrist HR
T130: Resting Baseline
EDA [µS]
Time [s]
EDA
T130: Priming
Heart Rate [BPM]
Time [s]
Wrist HR
T130: Priming
EDA [µS]
Time [s]
EDA

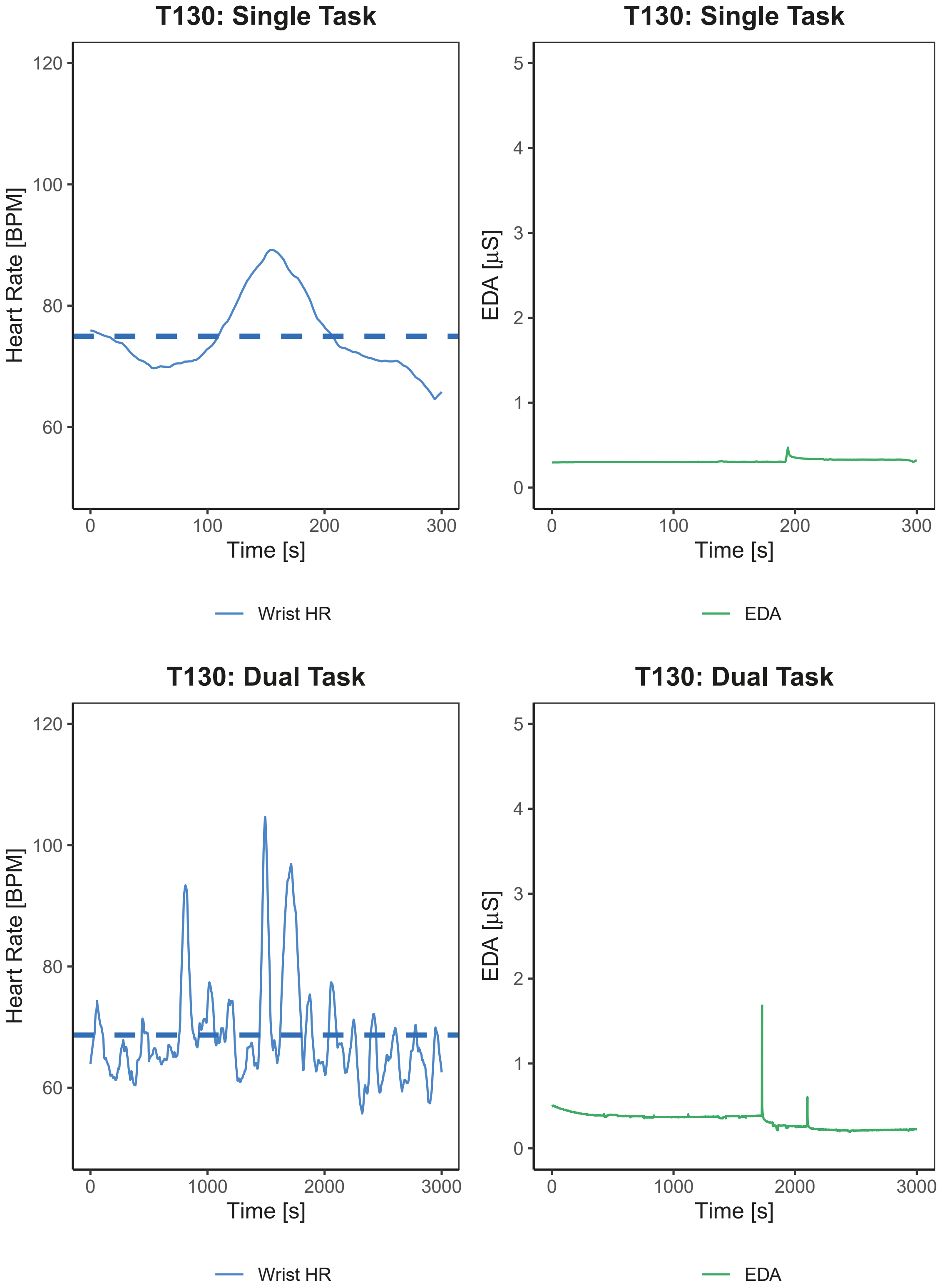
T130: Single Task
Heart Rate [BPM]
Time [s]
Wrist HR
T130: Single Task
EDA [µS]
Time [s]
EDA
T130: Dual Task
Heart Rate [BPM]
Time [s]
Wrist HR
T130: Dual Task
EDA [µS]
Time [s]
EDA

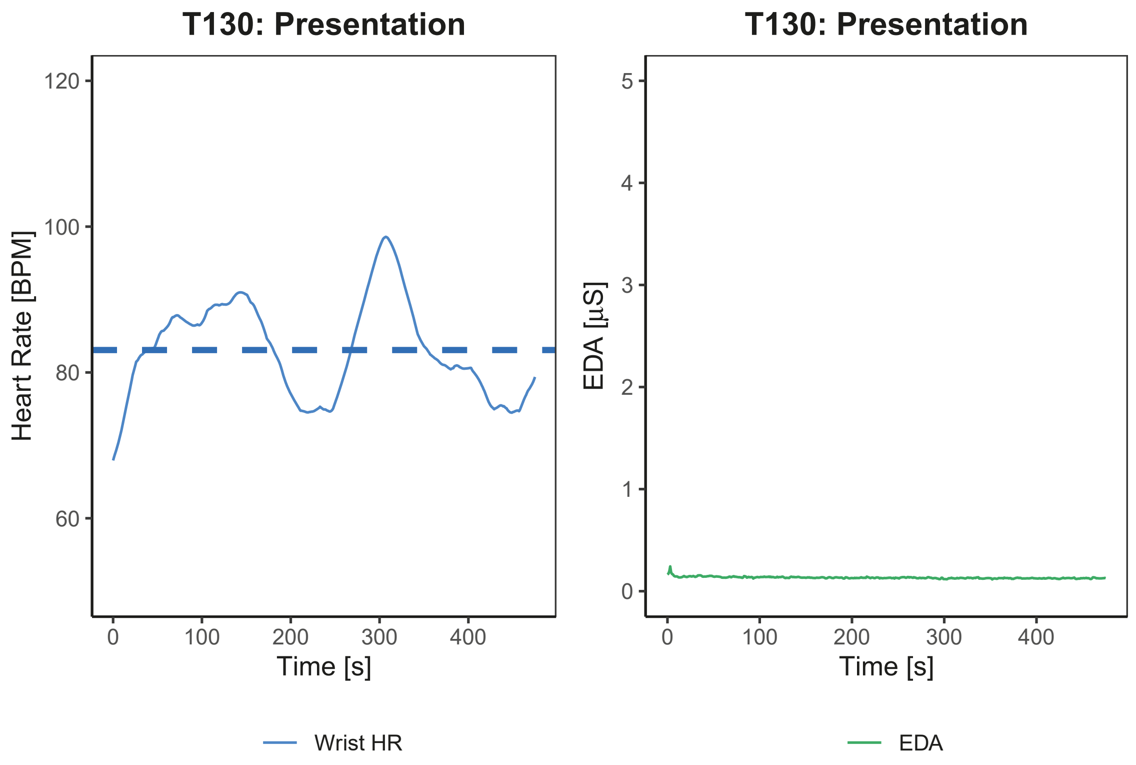
T130: Presentation
Heart Rate [BPM]
120
100
80
60
0
100
200
300
400
Time [s]
Wrist HR
T130: Presentation
EDA [µS]
5
4
3
2
1
0
0
100
200
300
400
Time [s]
EDA

--

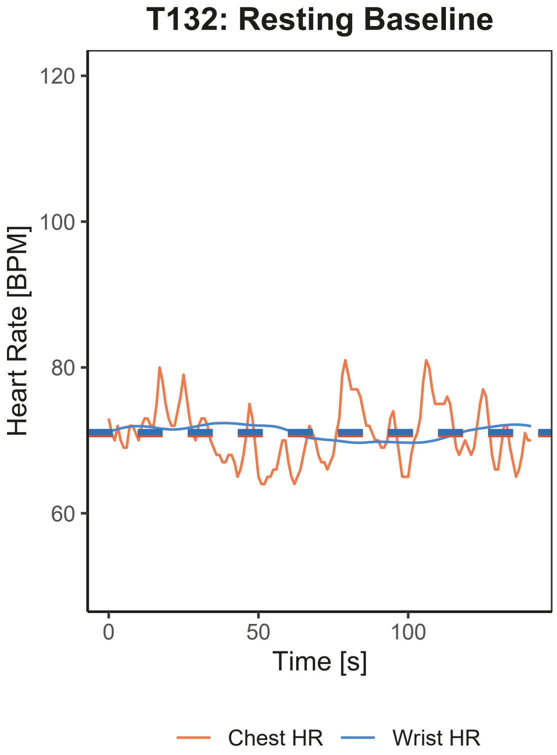
T132: Resting Baseline
Heart Rate [BPM]
Time [s]
Chest HR
Wrist HR

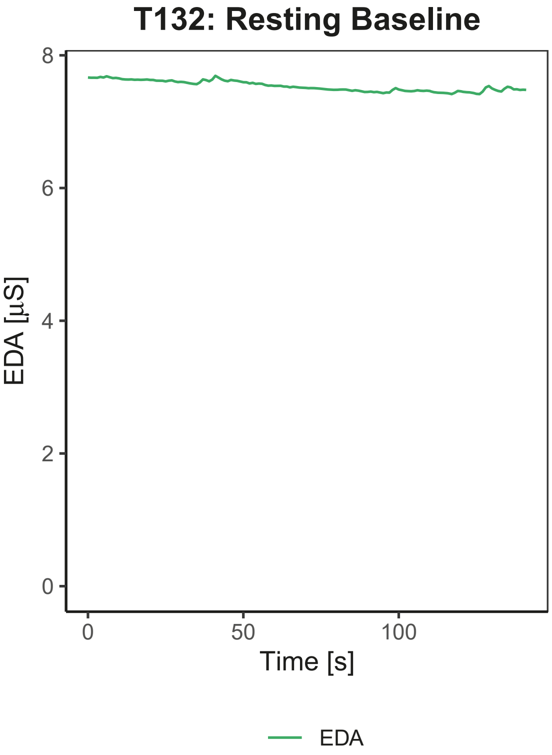
T132: Resting Baseline
EDA [µS]
Time [s]
EDA

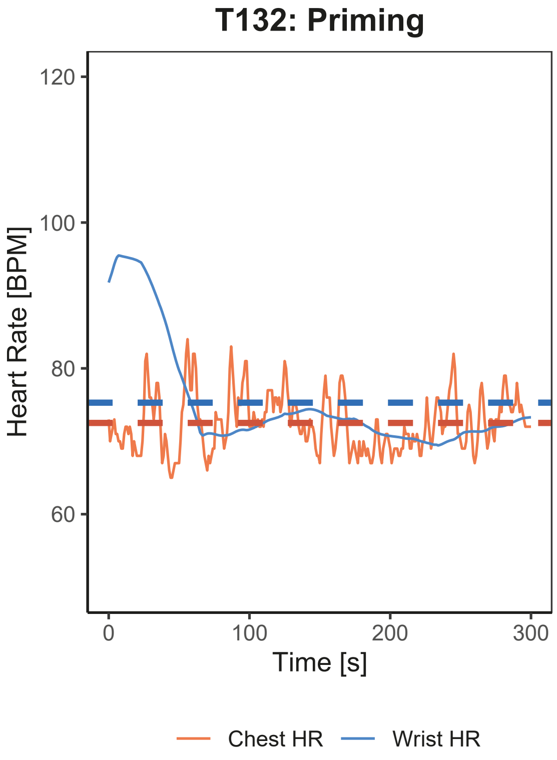
T132: Priming
Heart Rate [BPM]
Time [s]
Chest HR
Wrist HR

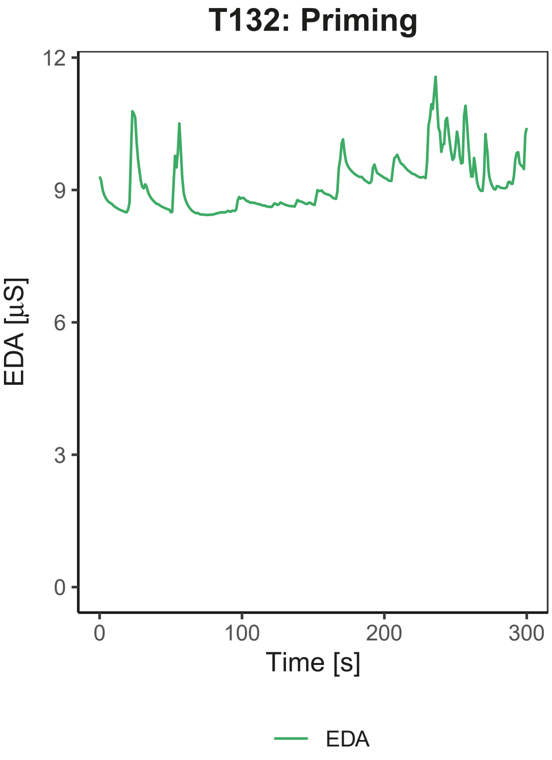
T132: Priming
EDA [µS]
Time [s]
EDA

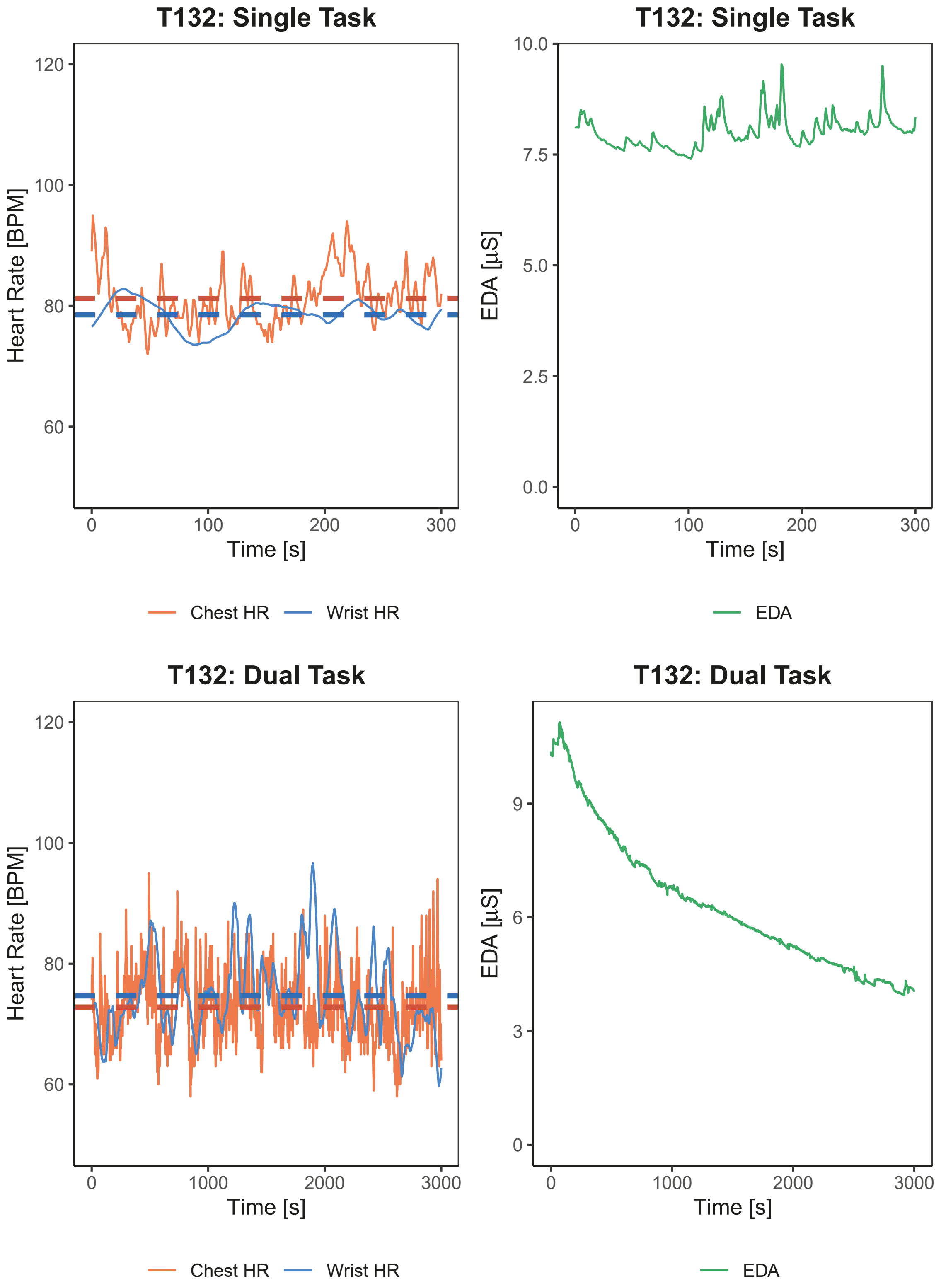
T132: Single Task
Heart Rate [BPM]
120
100
80
60
0
100
200
300
Time [s]
Chest HR
Wrist HR
T132: Single Task
EDA [µS]
10.0
7.5
5.0
2.5
0.0
0
100
200
300
Time [s]
EDA
T132: Dual Task
Heart Rate [BPM]
120
100
80
60
0
1000
2000
3000
Time [s]
Chest HR
Wrist HR
T132: Dual Task
EDA [µS]
9
6
3
0
0
1000
2000
3000
Time [s]
EDA

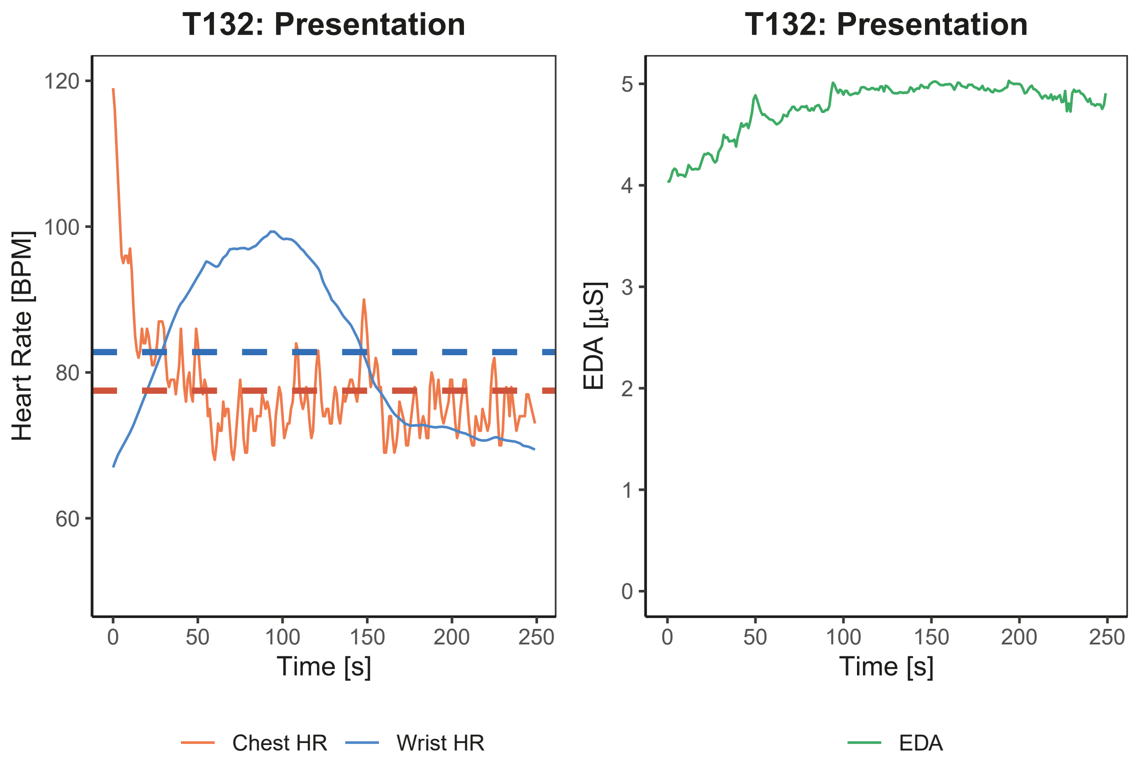
T132: Presentation
Heart Rate [BPM]
120
100
80
60
0
50
100
150
200
250
Time [s]
Chest HR
Wrist HR
T132: Presentation
EDA [µS]
5
4
3
2
1
0
0
50
100
150
200
250
Time [s]
EDA

--

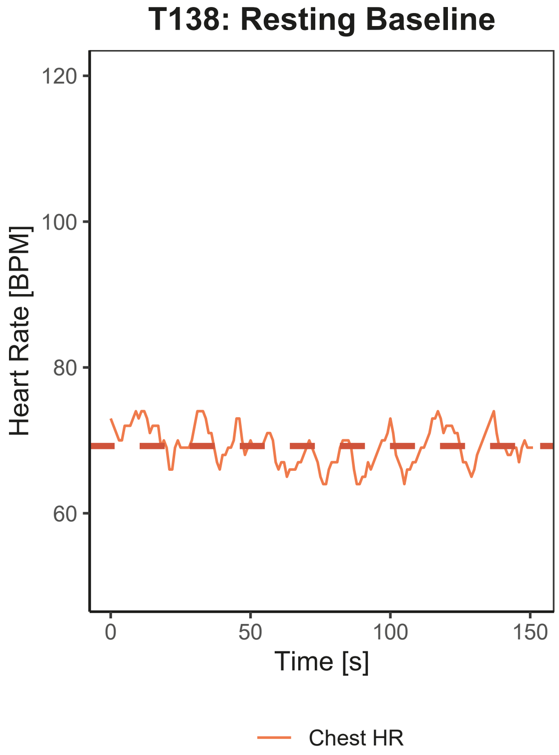
T138: Resting Baseline
Heart Rate [BPM]
120
100
80
60
0
50
100
150
Time [s]
Chest HR

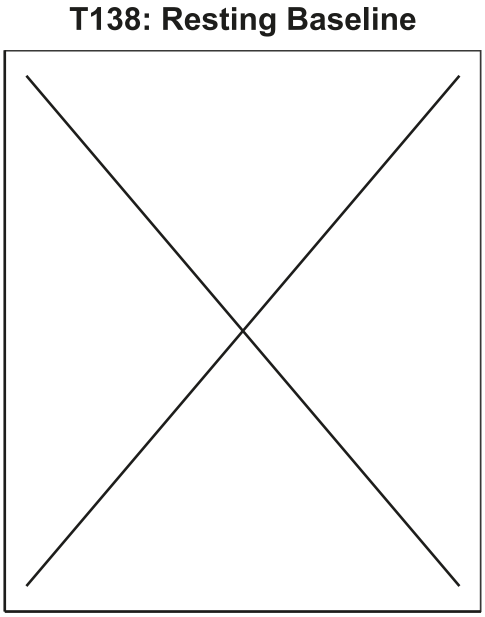
T138: Resting Baseline

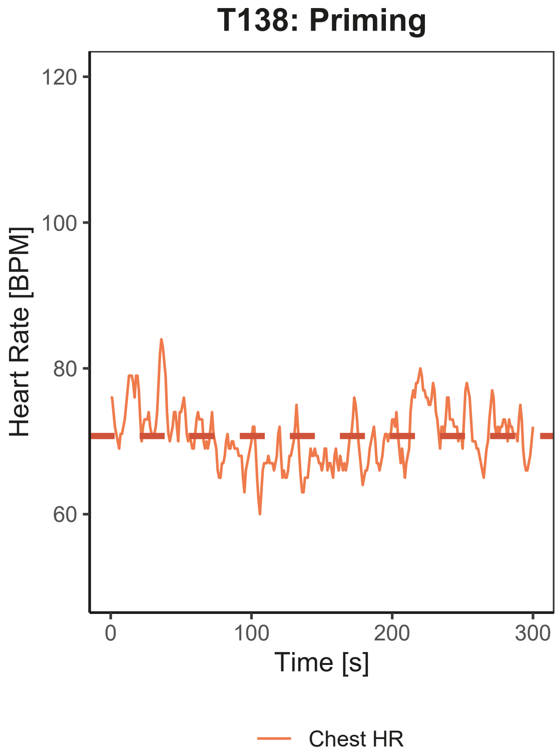
T138: Priming
Heart Rate [BPM]
120
100
80
60
0
100
200
300
Time [s]
Chest HR

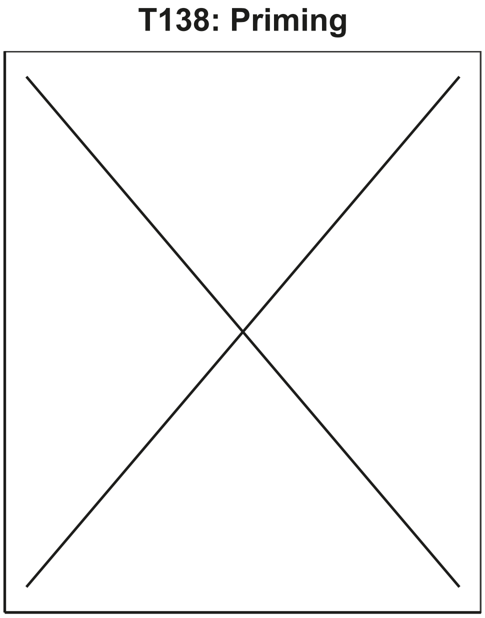
T138: Priming

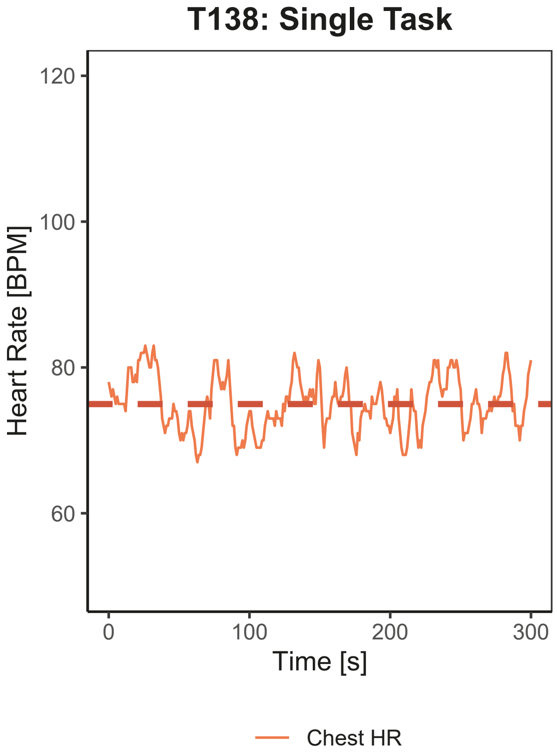
T138: Single Task
Heart Rate [BPM]
120
100
80
60
0
100
200
300
Time [s]
Chest HR

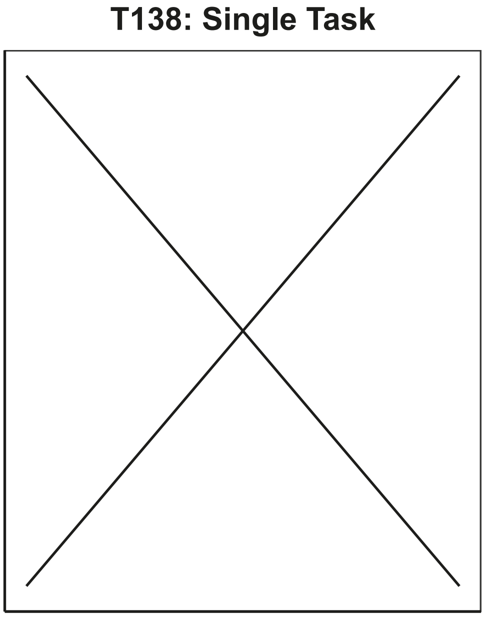
T138: Single Task

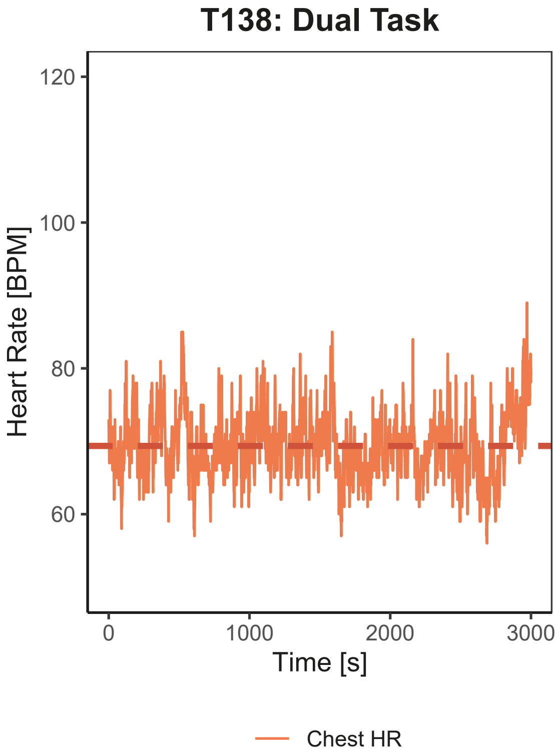
T138: Dual Task
Heart Rate [BPM]
120
100
80
60
0
1000
2000
3000
Time [s]
Chest HR

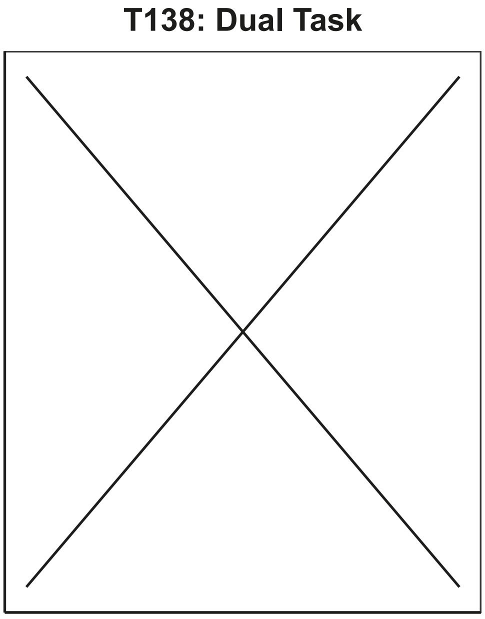
T138: Dual Task

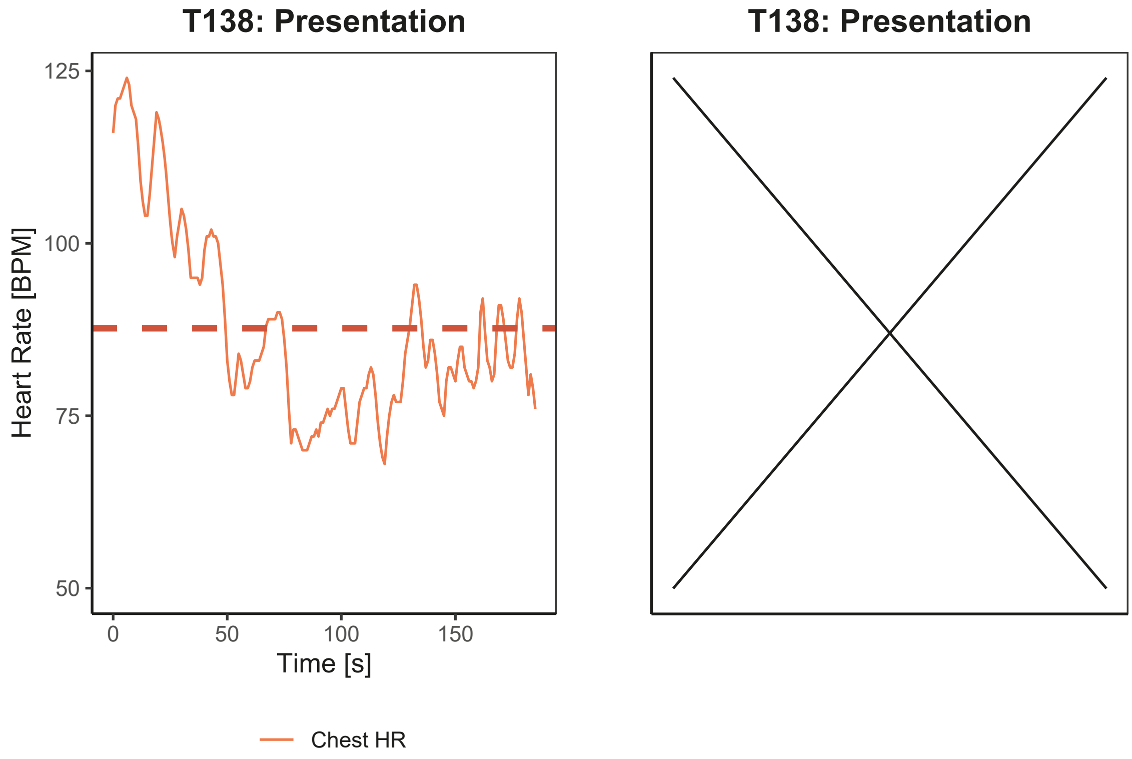
T138: Presentation
Heart Rate [BPM]
125
100
75
50
0
50
100
150
Time [s]
Chest HR
T138: Presentation

--

T139: Resting Baseline

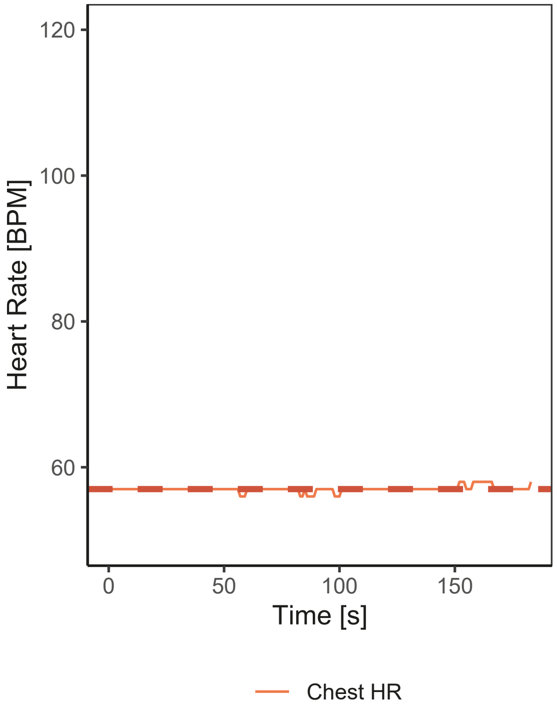

T139: Resting Baseline

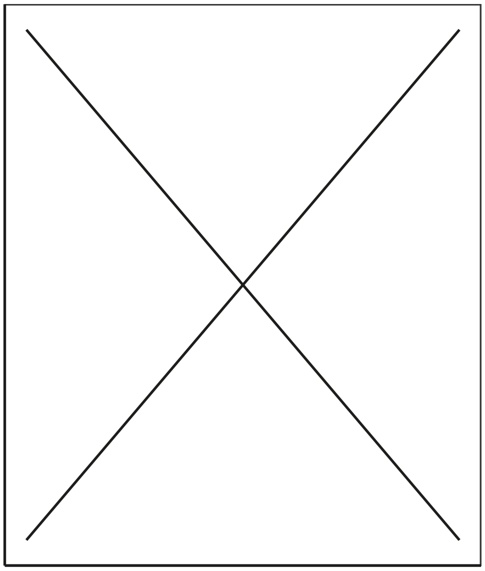

T139: Priming

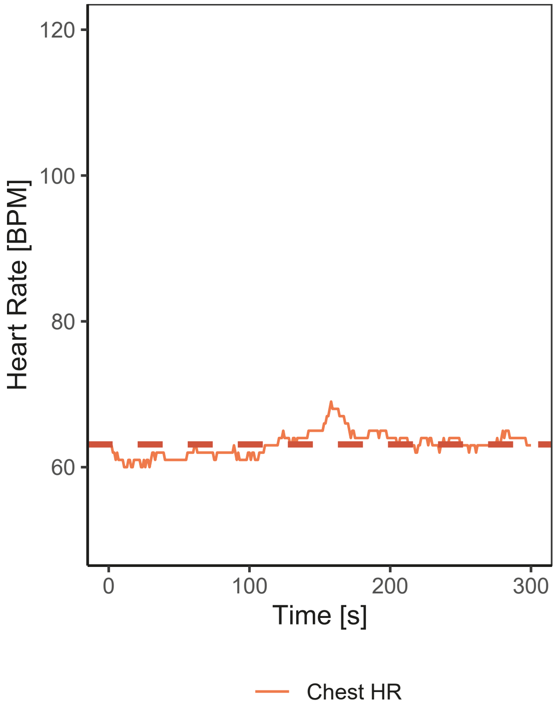

T139: Priming

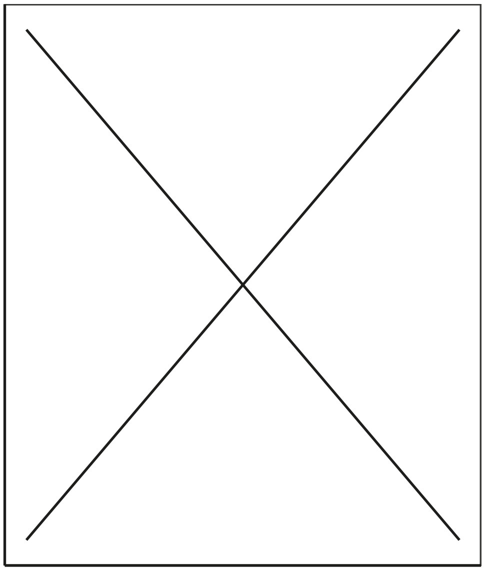

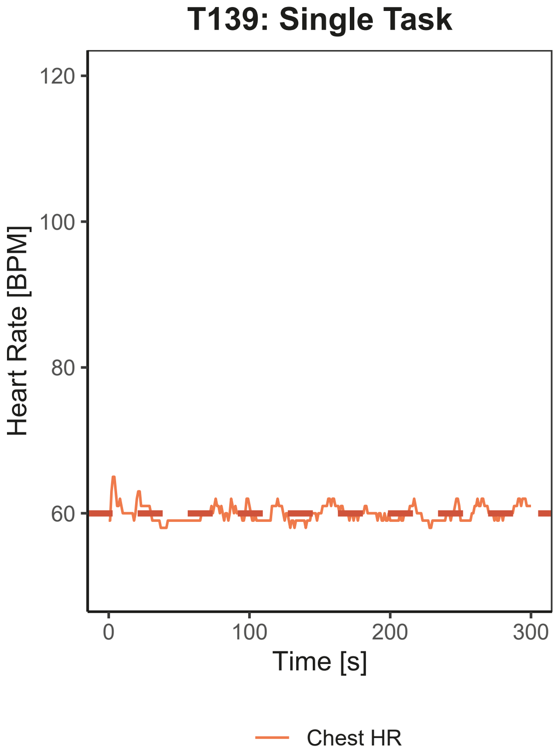
T139: Single Task
Heart Rate [BPM]
120
100
80
60
0
100
200
300
Time [s]
Chest HR

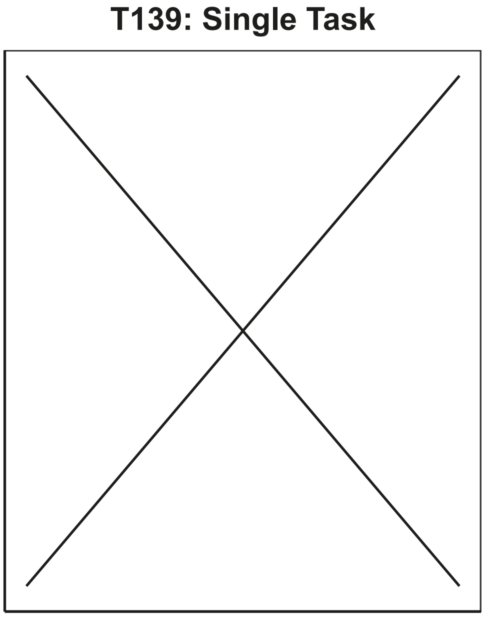
T139: Single Task

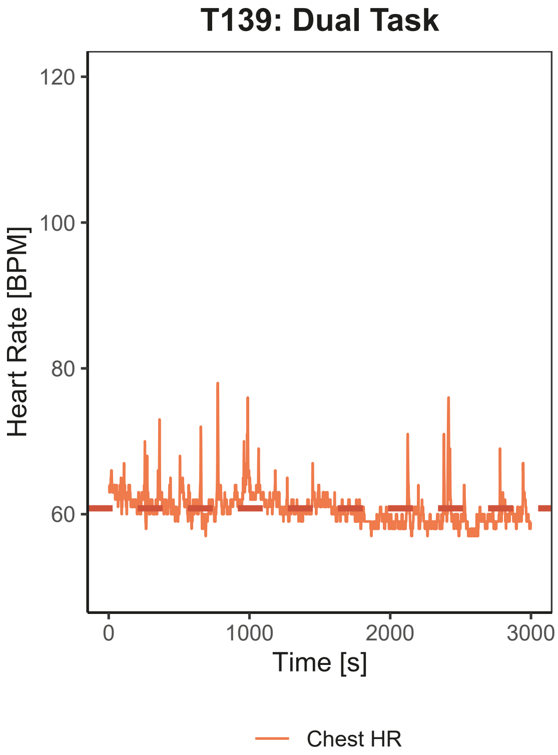
T139: Dual Task
Heart Rate [BPM]
120
100
80
60
0
1000
2000
3000
Time [s]
Chest HR

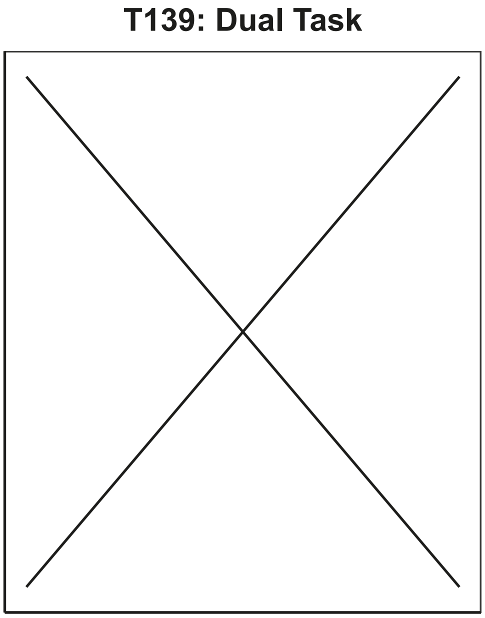
T139: Dual Task

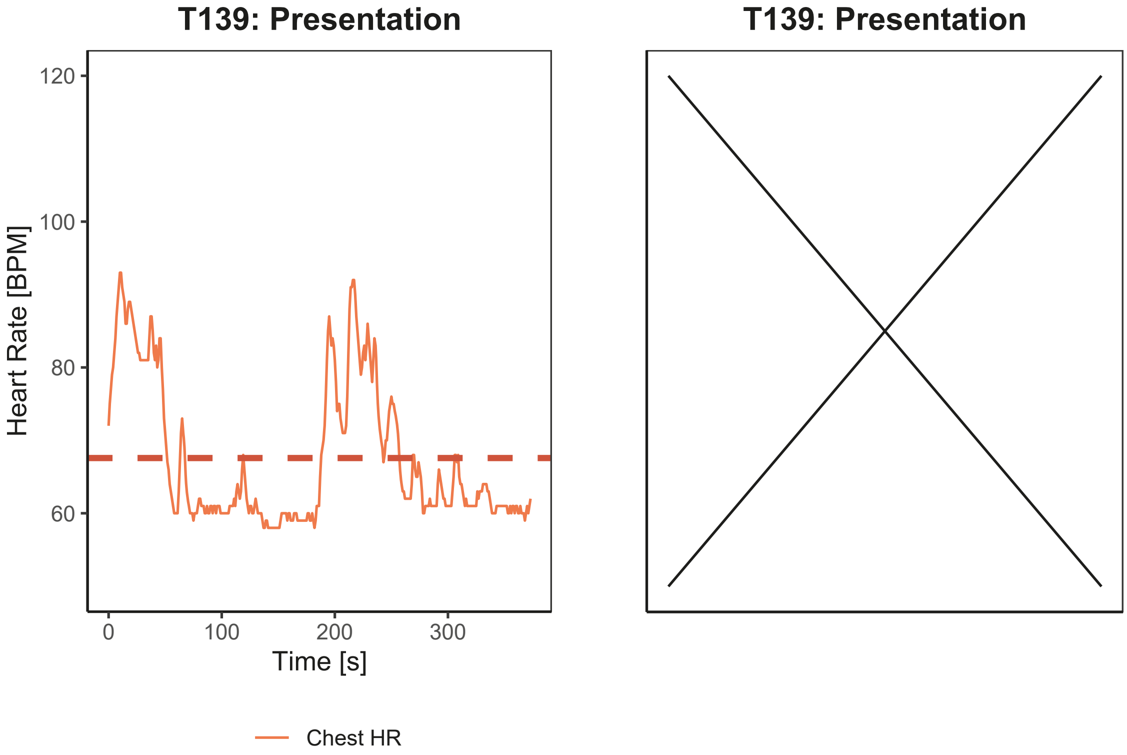
T139: Presentation
Heart Rate [BPM]
120
100
80
60
0
100
200
300
Time [s]
Chest HR
T139: Presentation

--

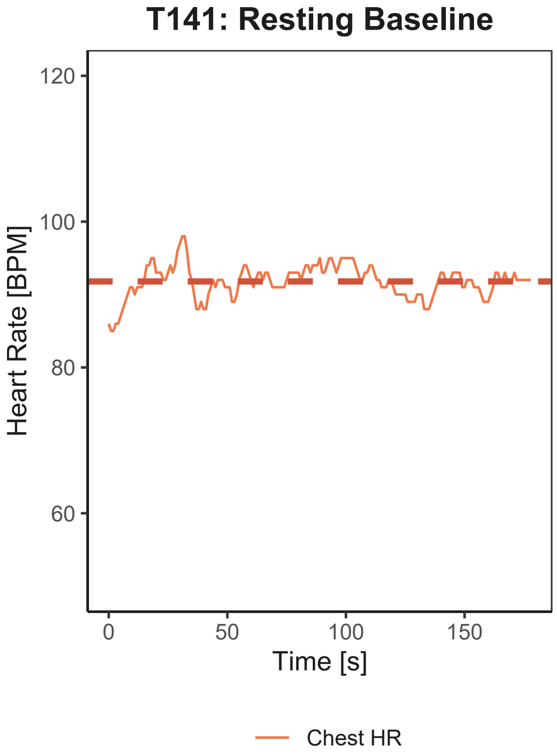
T141: Resting Baseline
Heart Rate [BPM]
120
100
80
60
0
50
100
150
Time [s]
Chest HR

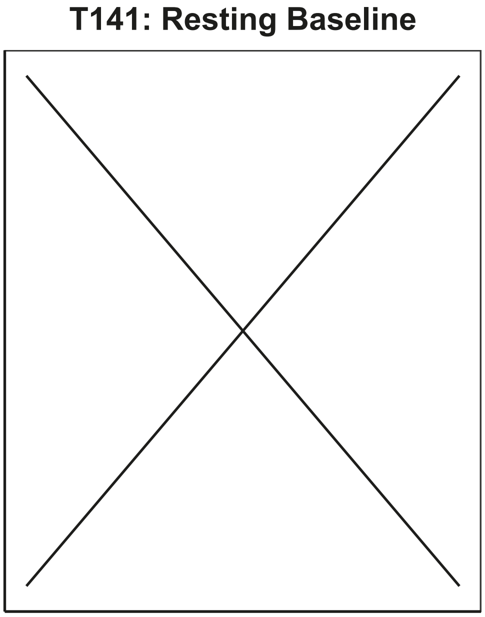
T141: Resting Baseline

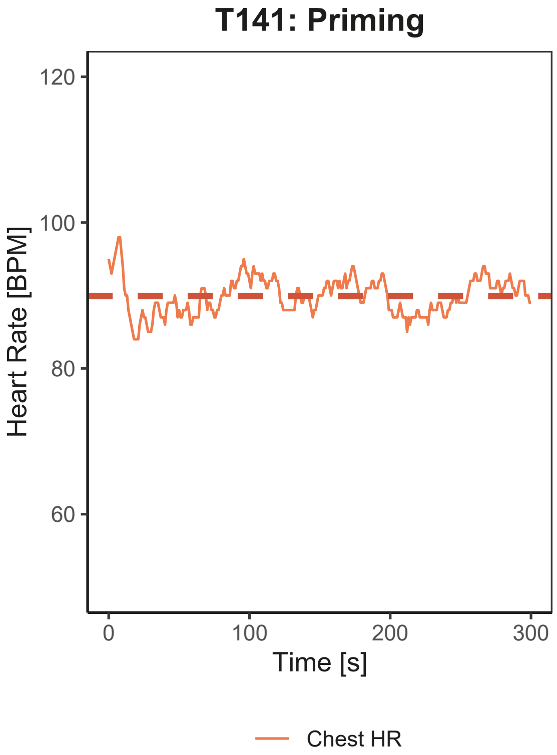
T141: Priming
Heart Rate [BPM]
120
100
80
60
0
100
200
300
Time [s]
Chest HR

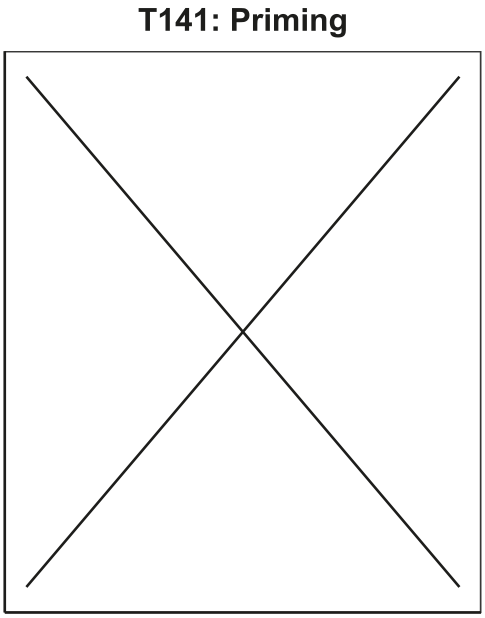
T141: Priming

T141: Single Task

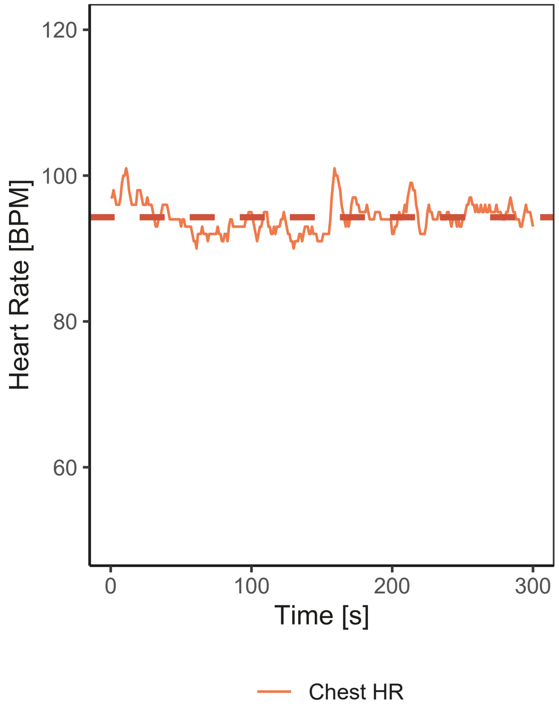

T141: Single Task

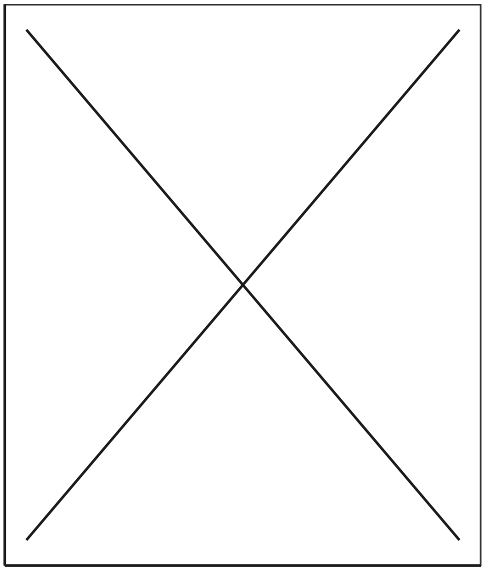

T141: Dual Task

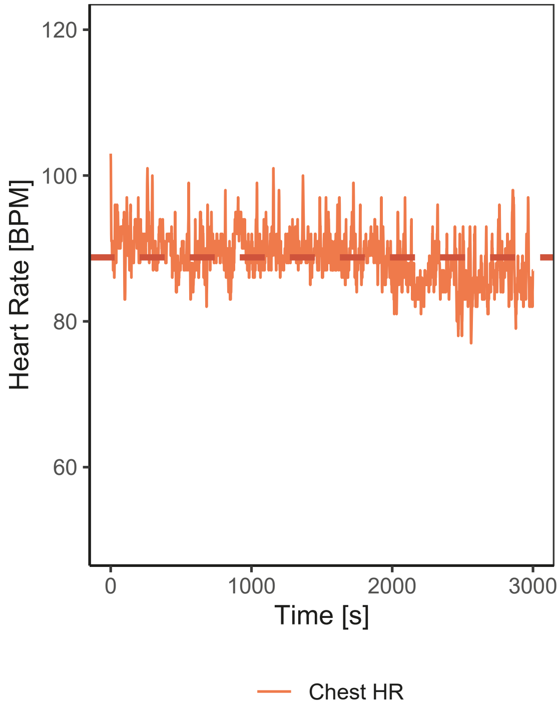

T141: Dual Task

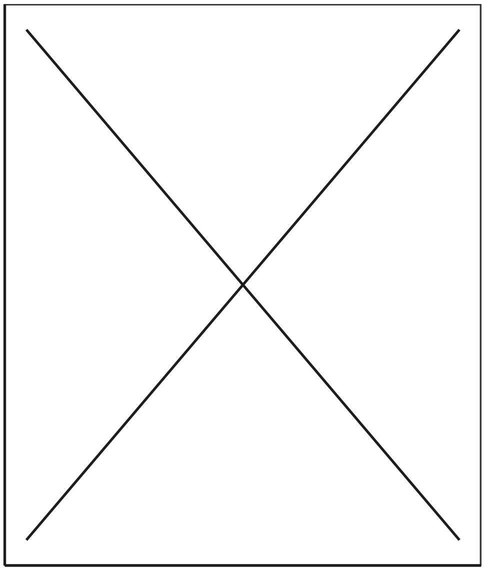

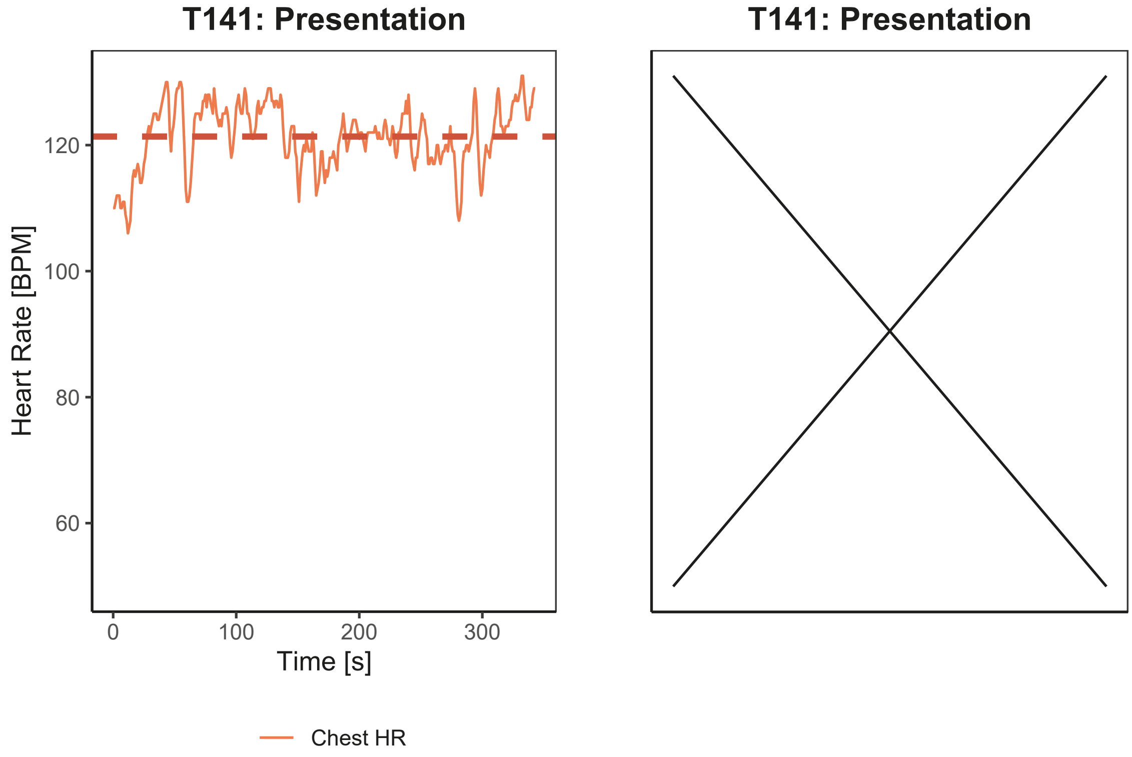
T141: Presentation
Heart Rate [BPM]
120
100
80
60
0
100
200
300
Time [s]
Chest HR
T141: Presentation

--

T144: Resting Baseline

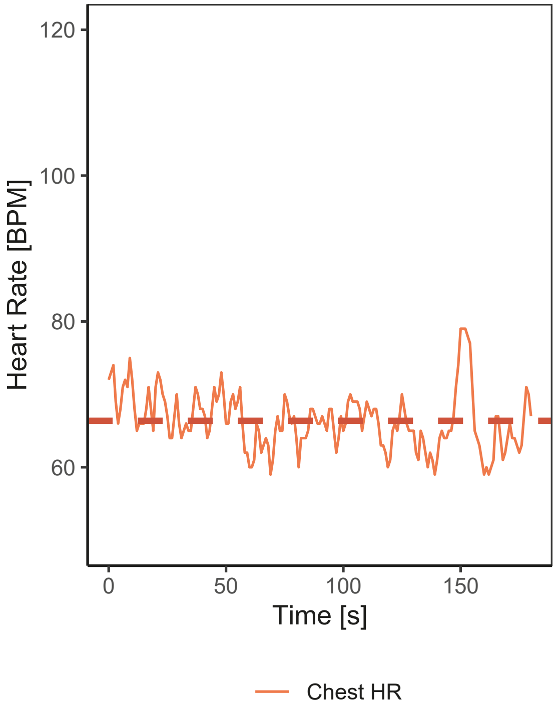

T144: Resting Baseline

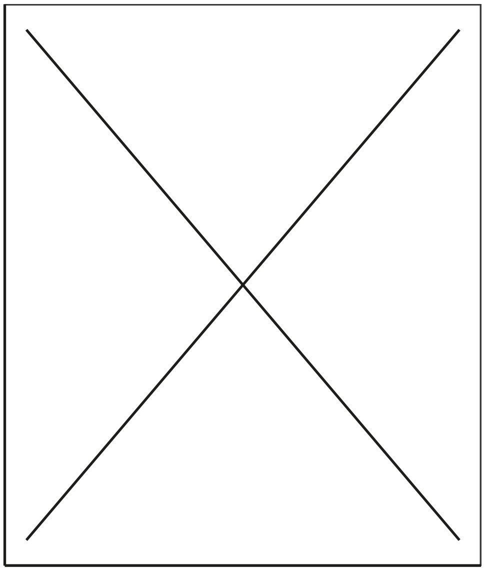

T144: Priming

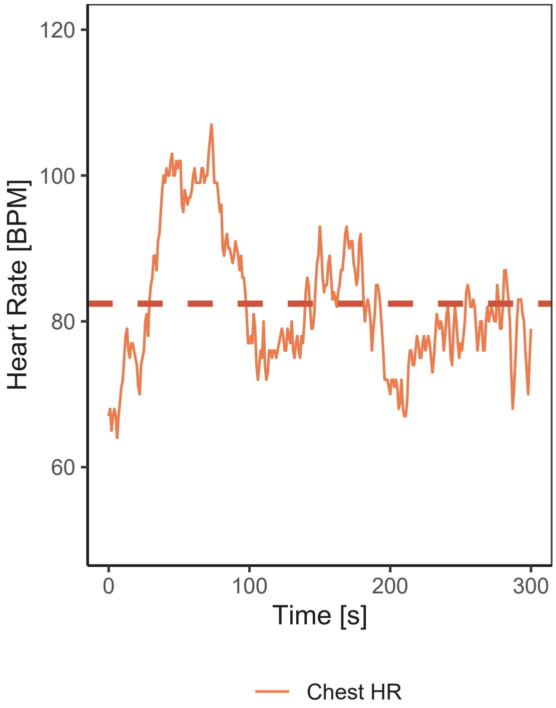

T144: Priming

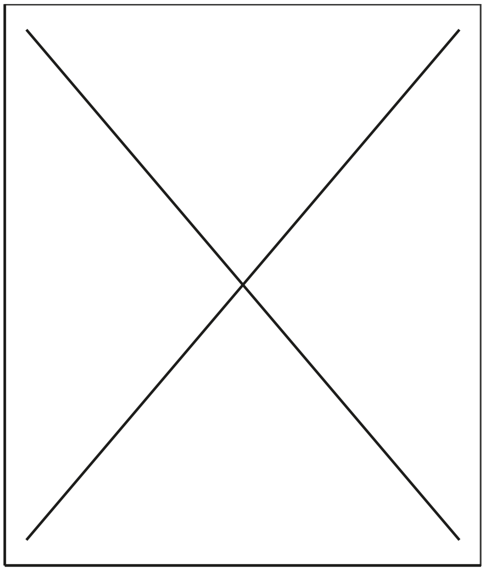

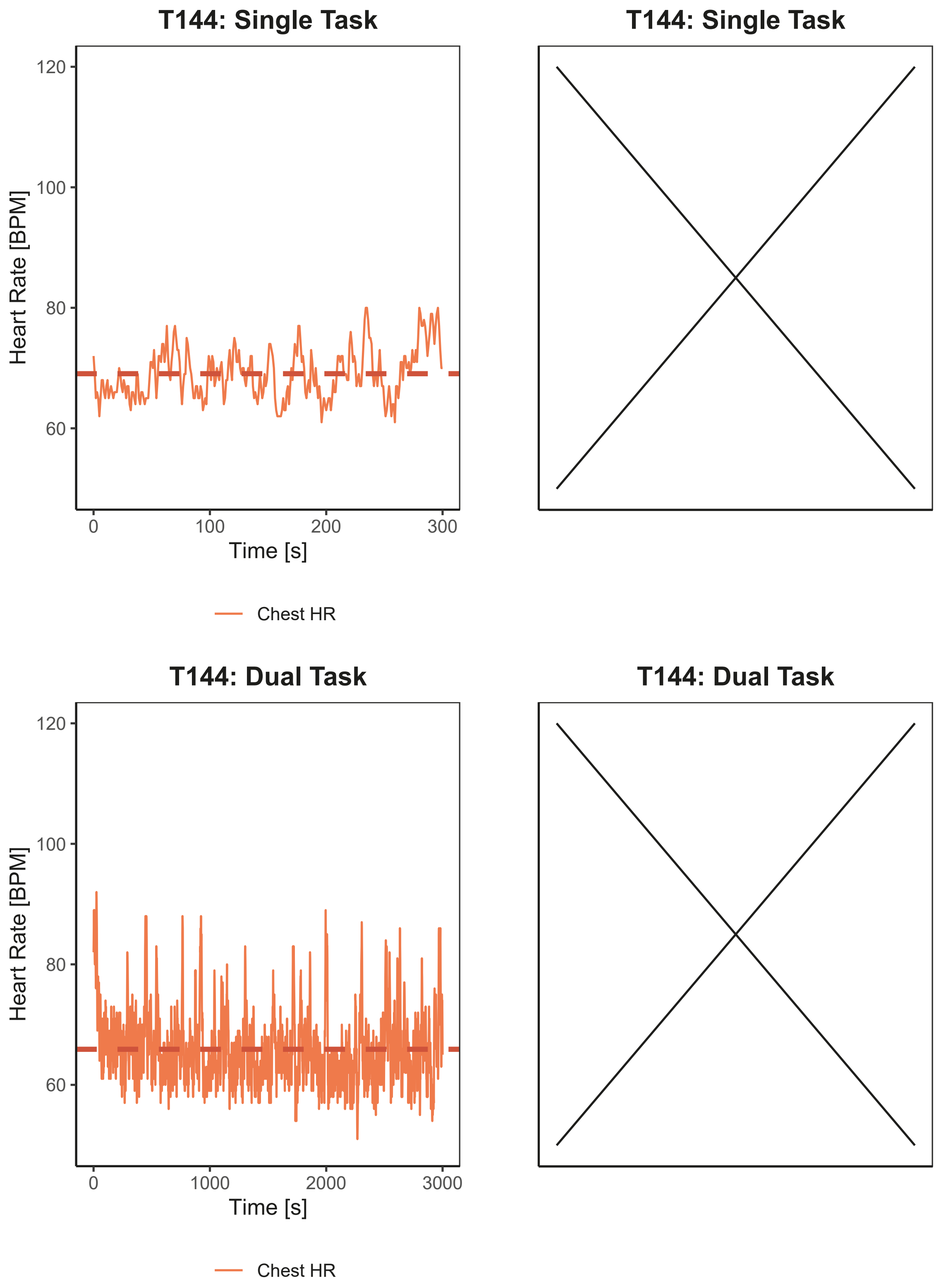
T144: Single Task
Heart Rate [BPM]
120
100
80
60
0
100
200
300
Time [s]
Chest HR
T144: Single Task
T144: Dual Task
Heart Rate [BPM]
120
100
80
60
0
1000
2000
3000
Time [s]
Chest HR
T144: Dual Task

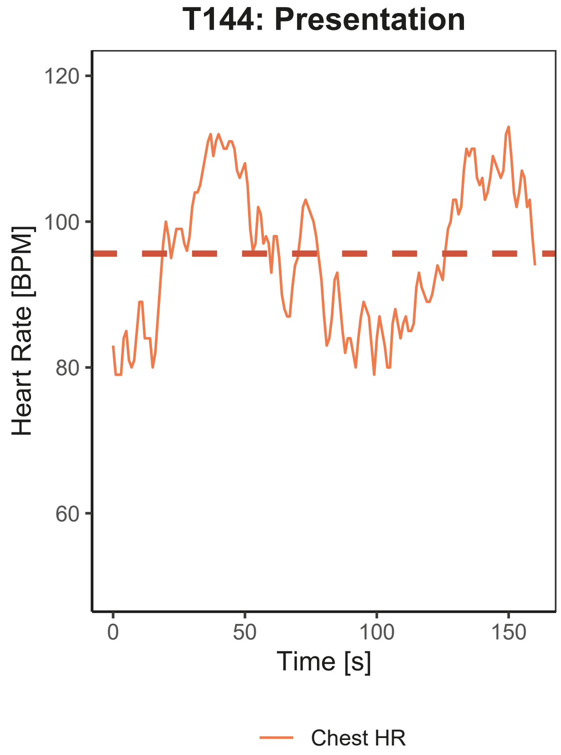
T144: Presentation
Heart Rate [BPM]
120
100
80
60
0
50
100
150
Time [s]
Chest HR

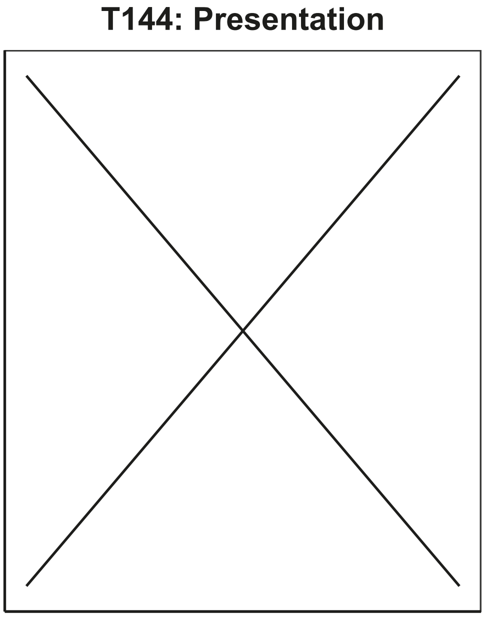
T144: Presentation

```
## ------------------------------------------------------------------------------- ##
```

T145: Resting Baseline

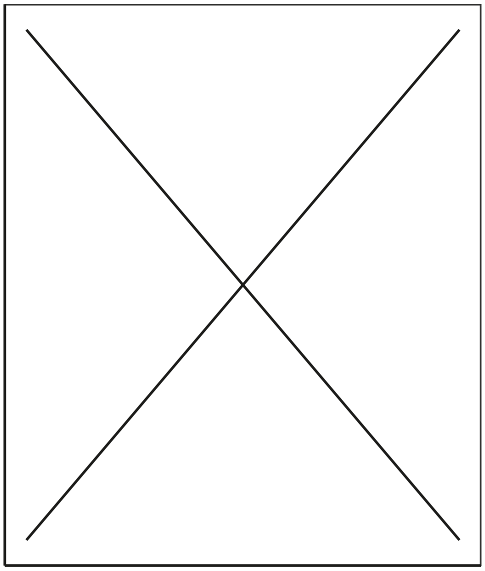

T145: Resting Baseline

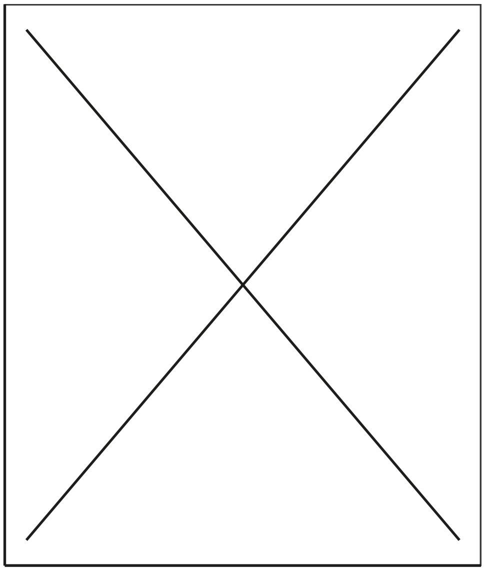

T145: Priming

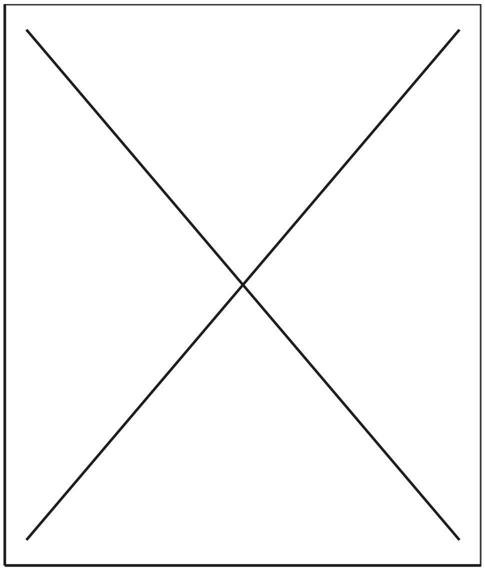

T145: Priming

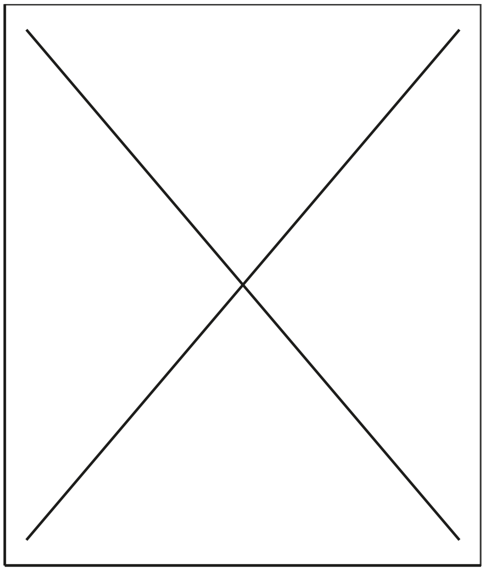

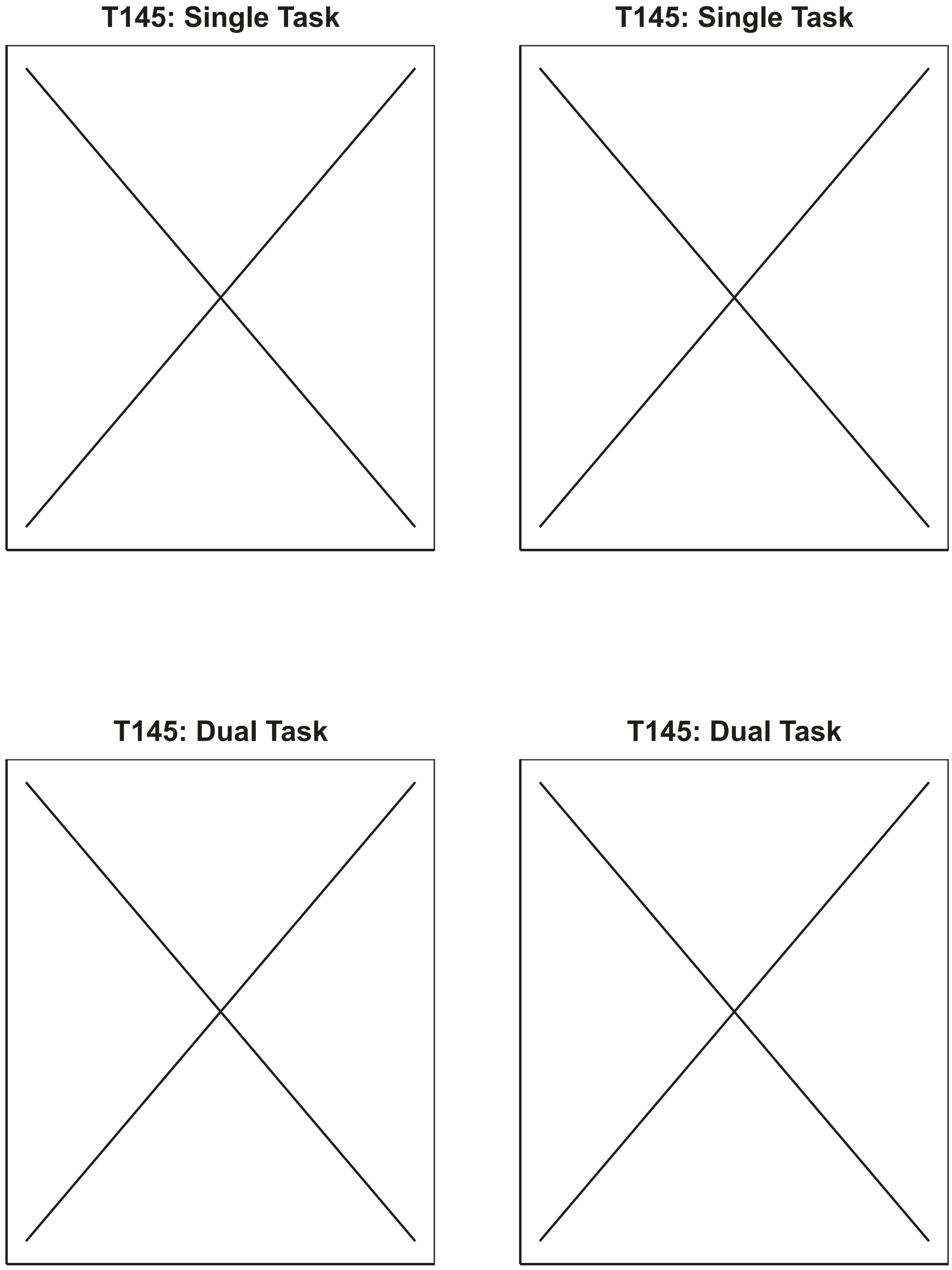
T145: Single Task
T145: Single Task
T145: Dual Task
T145: Dual Task

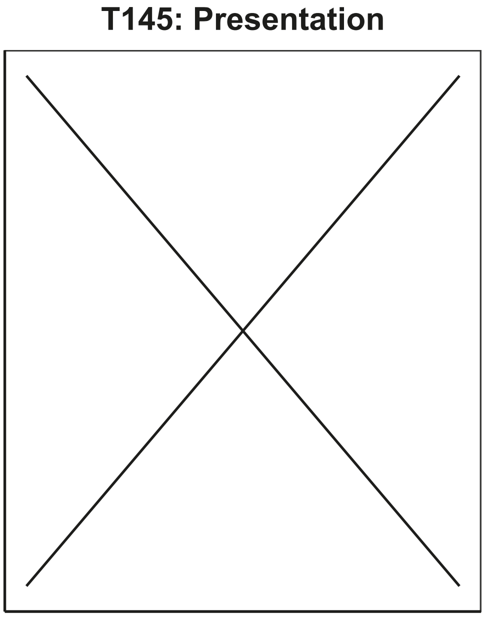
T145: Presentation

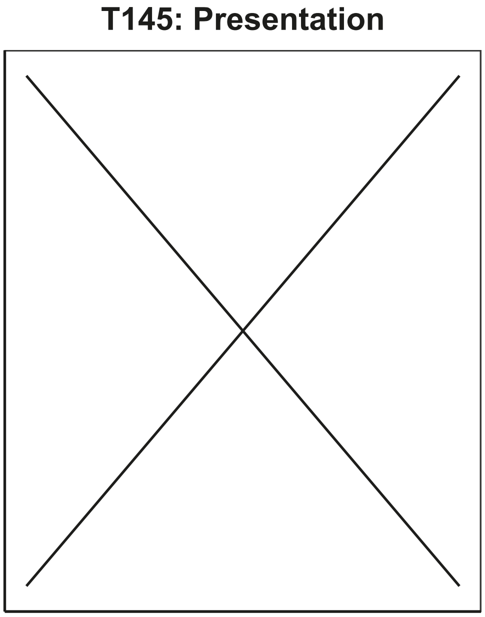
T145: Presentation

```
## -------------------------------------------------------------------------------- ##
```

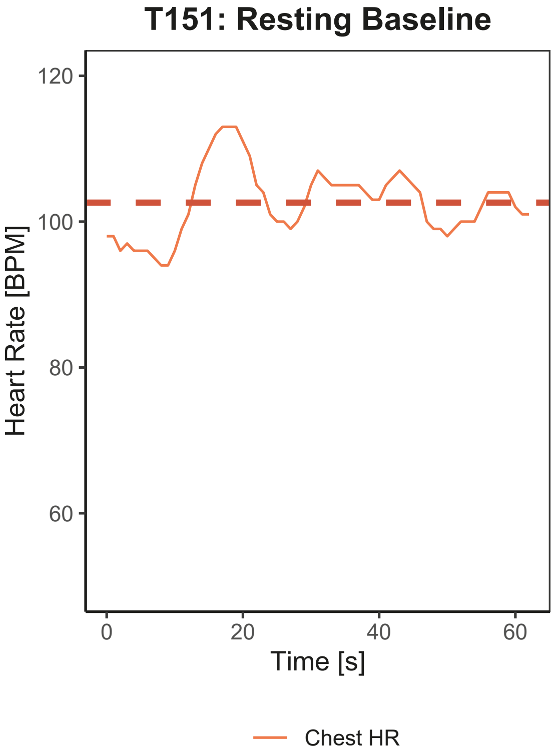
T151: Resting Baseline
Heart Rate [BPM]
120
100
80
60
0
20
40
60
Time [s]
Chest HR

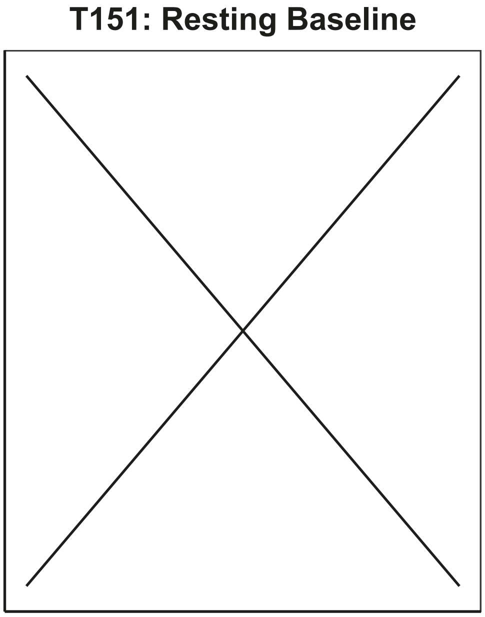
T151: Resting Baseline

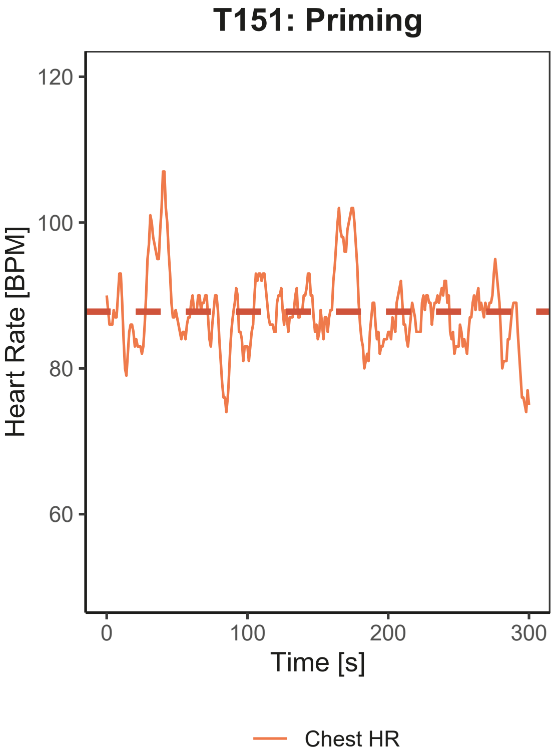
T151: Priming
Heart Rate [BPM]
120
100
80
60
0
100
200
300
Time [s]
Chest HR

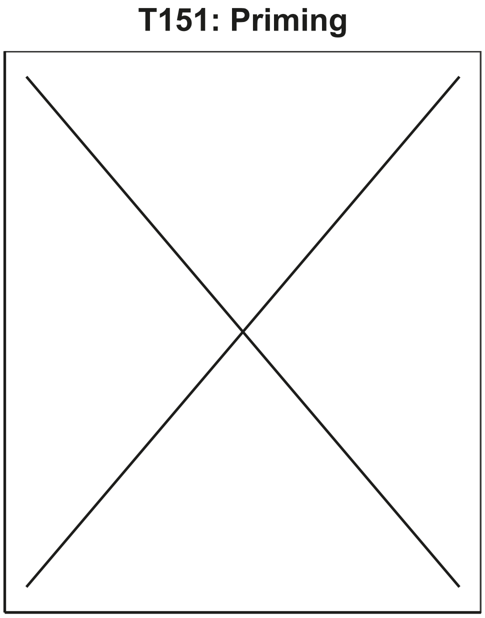
T151: Priming

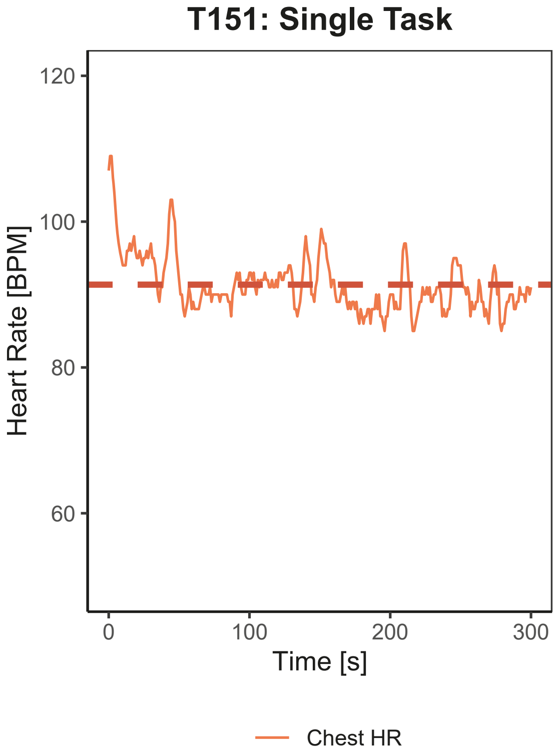
T151: Single Task
Heart Rate [BPM]
120
100
80
60
0
100
200
300
Time [s]
Chest HR

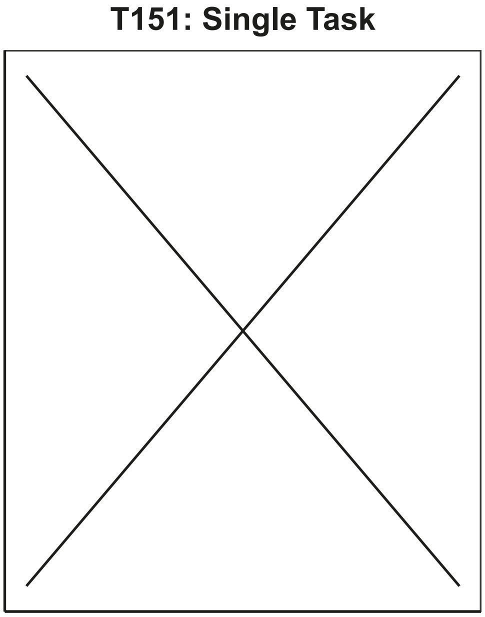
T151: Single Task

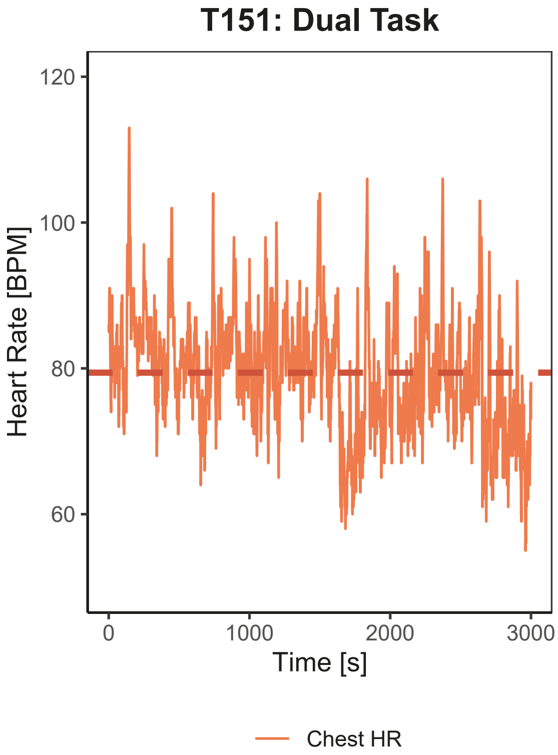
T151: Dual Task
Heart Rate [BPM]
120
100
80
60
0
1000
2000
3000
Time [s]
Chest HR

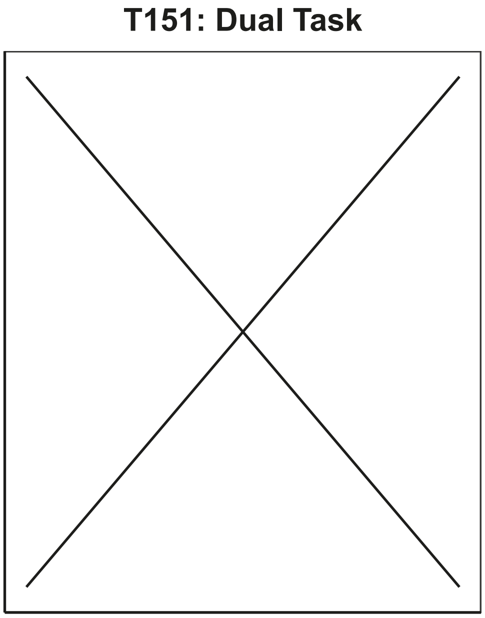
T151: Dual Task

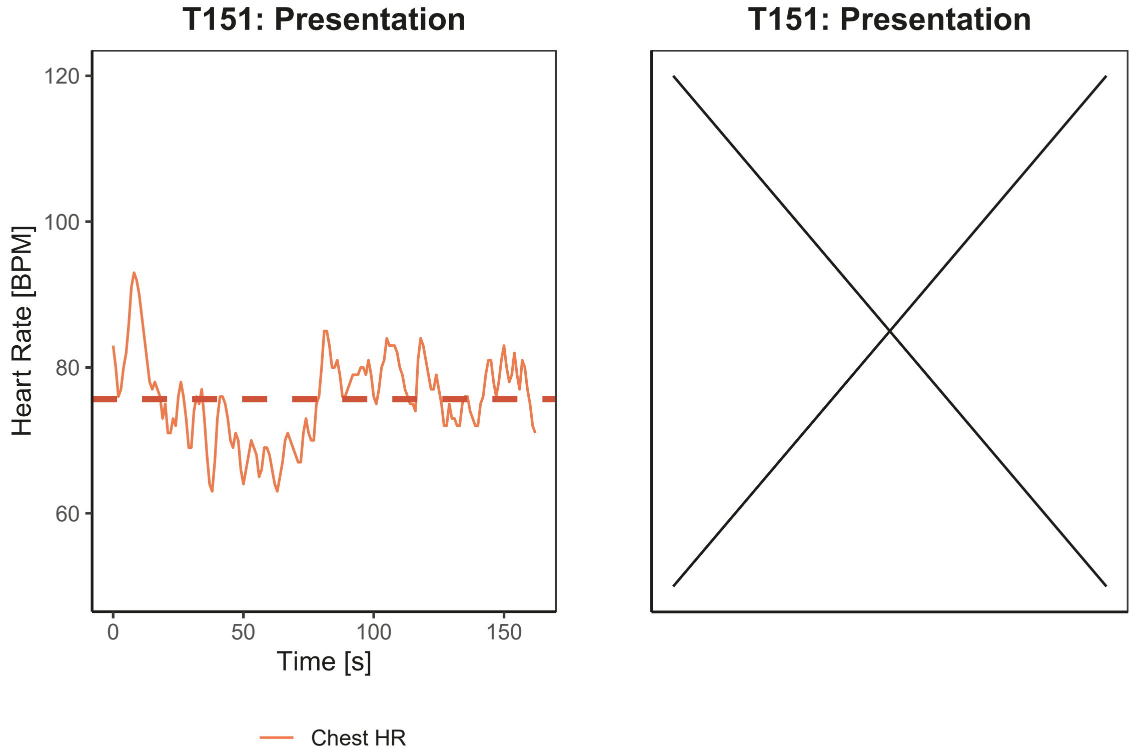
T151: Presentation
T151: Presentation
Heart Rate [BPM]
120
100
80
60
0
50
100
150
Time [s]
Chest HR

```
## ------------------------------------------------------------------------------ ##
```

T152: Resting Baseline

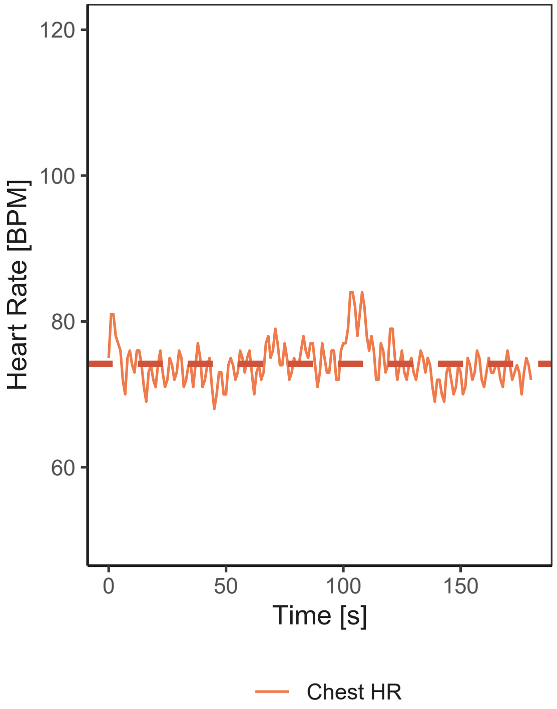

T152: Resting Baseline

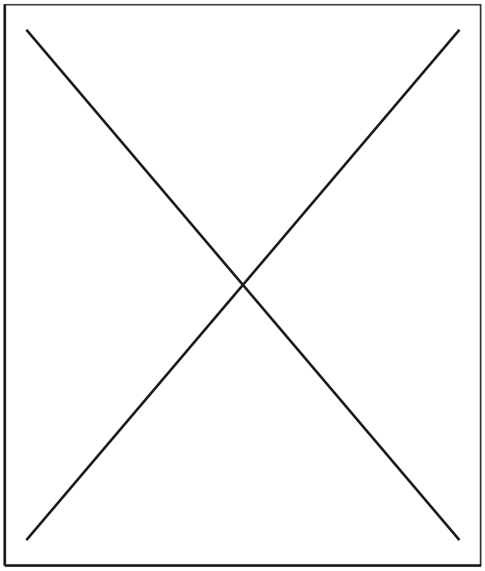

T152: Priming

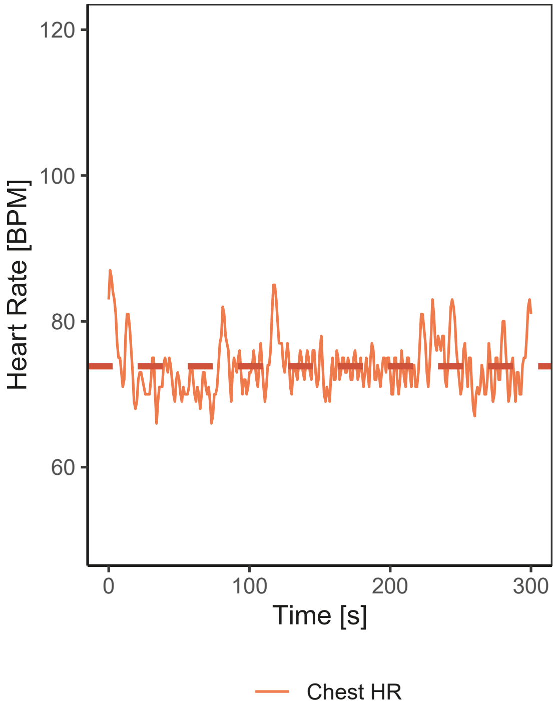

T152: Priming

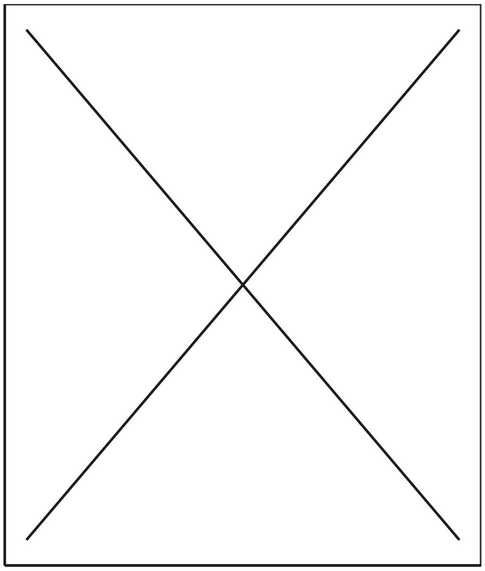

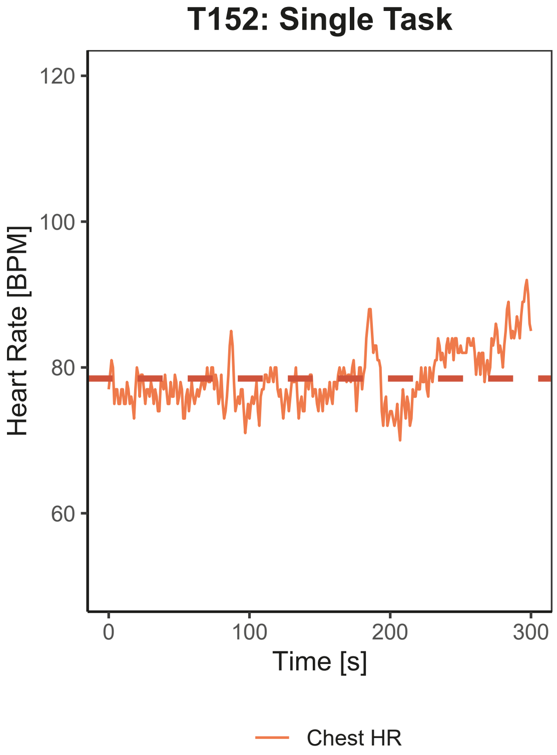
T152: Single Task
Heart Rate [BPM]
120
100
80
60
0
100
200
300
Time [s]
Chest HR

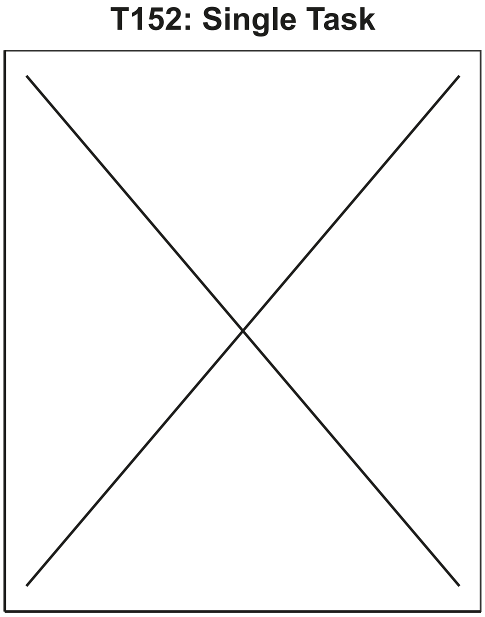
T152: Single Task

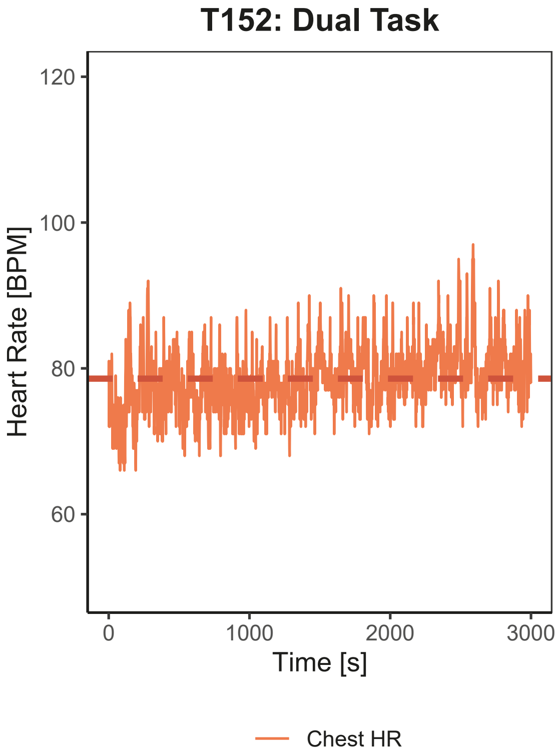
T152: Dual Task
Heart Rate [BPM]
120
100
80
60
0
1000
2000
3000
Time [s]
Chest HR

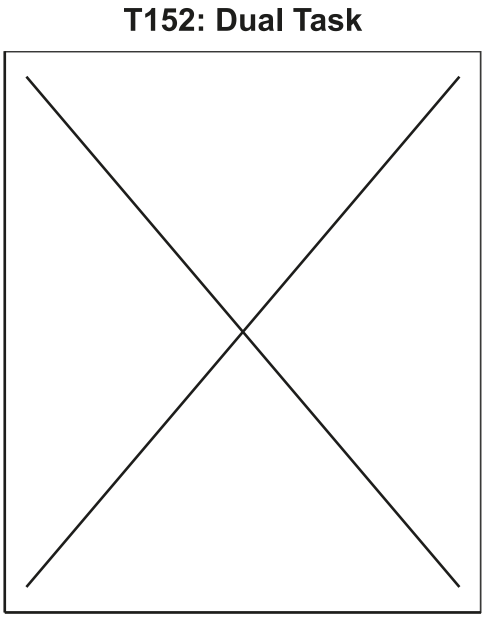
T152: Dual Task

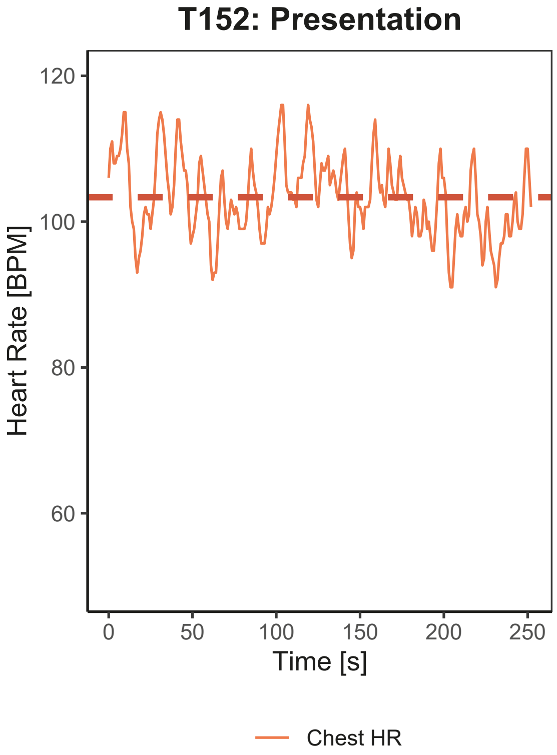
T152: Presentation
Heart Rate [BPM]
120
100
80
60
0
50
100
150
200
250
Time [s]
Chest HR

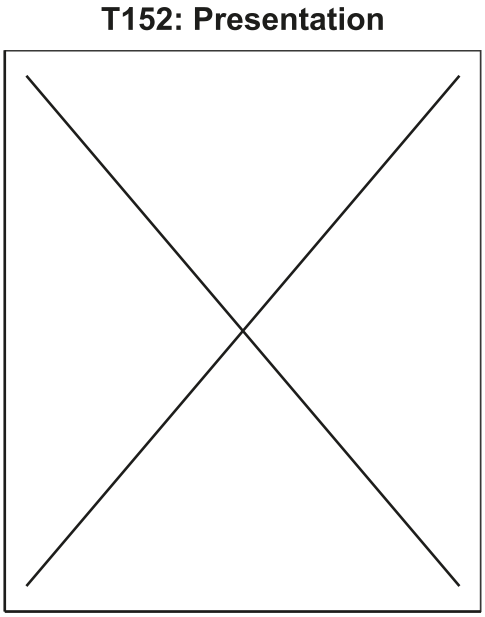
T152: Presentation

```
## ---------------------------------------------------------------------------- ##
```

T154: Resting Baseline

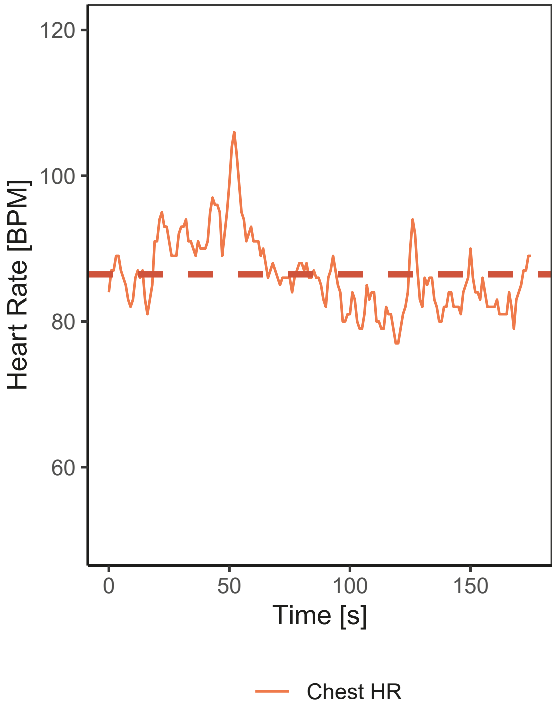

T154: Resting Baseline

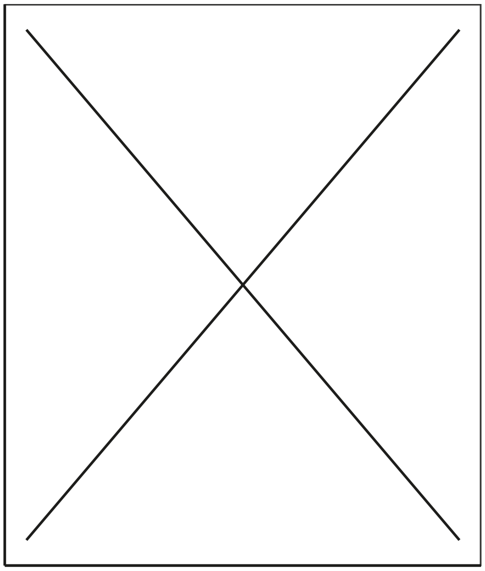

T154: Priming

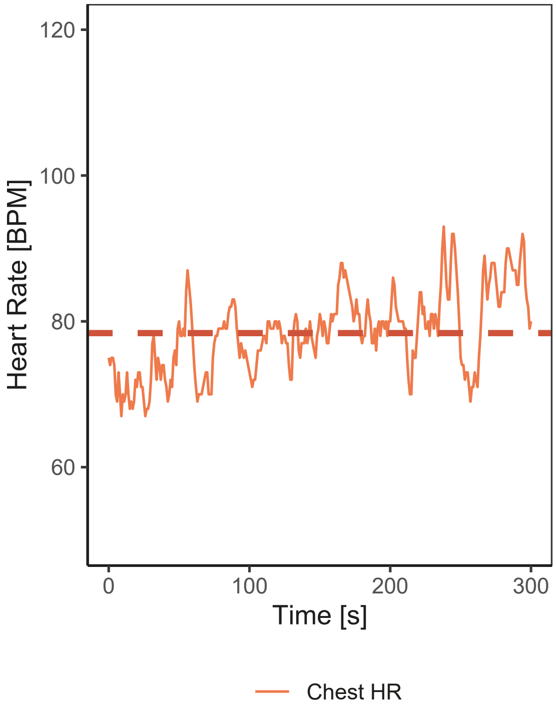

T154: Priming

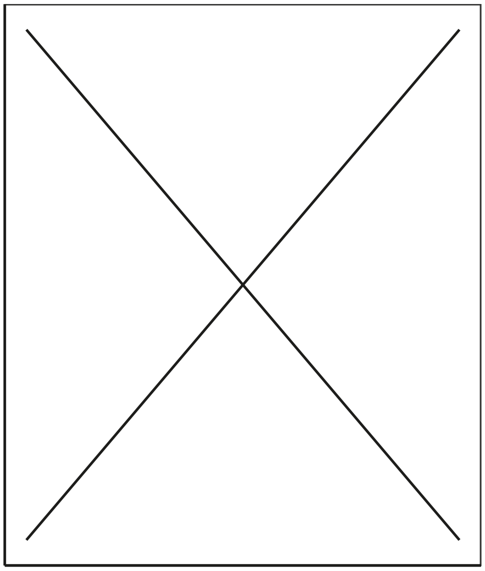

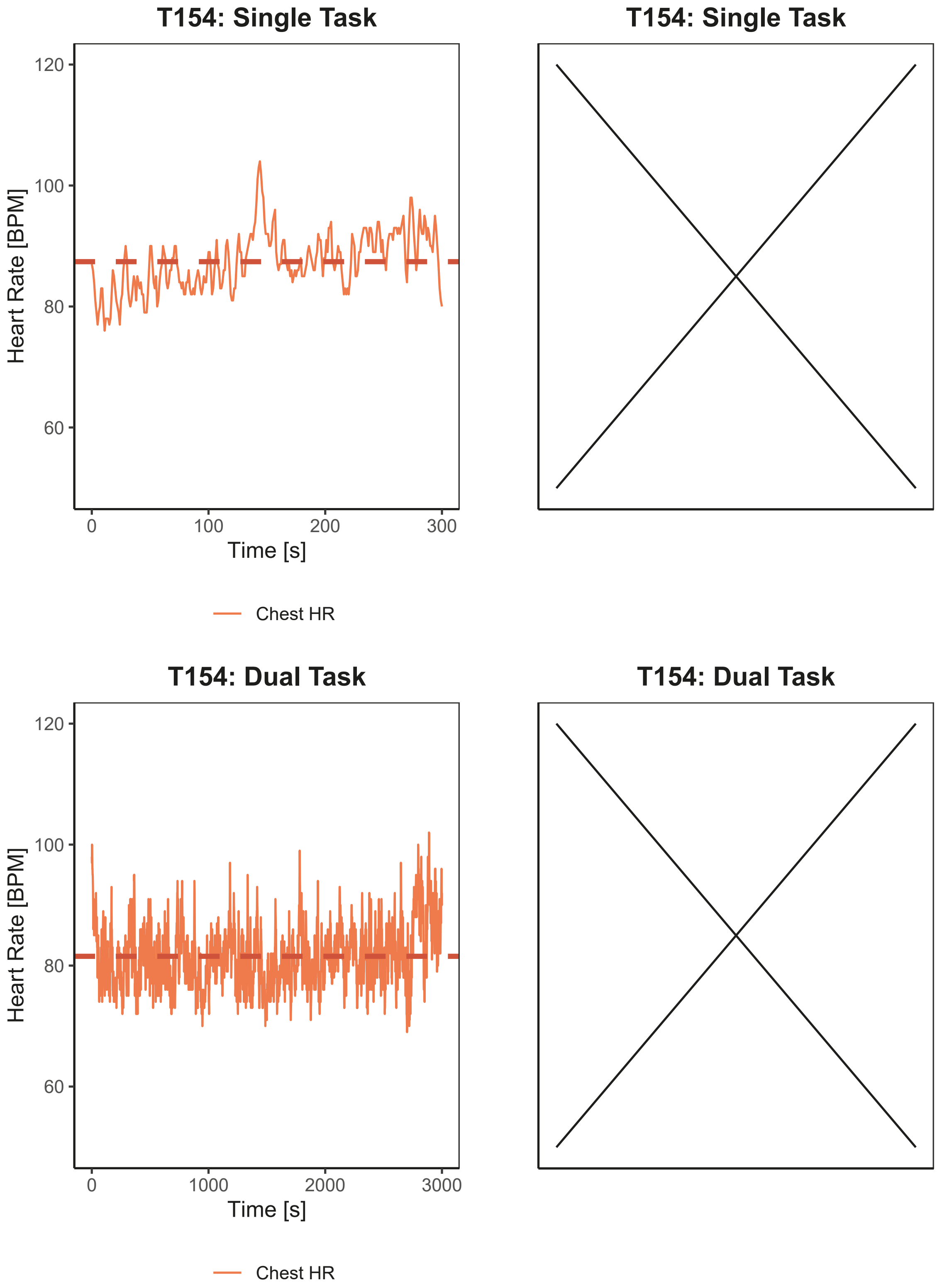
T154: Single Task
Heart Rate [BPM]
120
100
80
60
0
100
200
300
Time [s]
Chest HR
T154: Single Task
T154: Dual Task
Heart Rate [BPM]
120
100
80
60
0
1000
2000
3000
Time [s]
Chest HR
T154: Dual Task

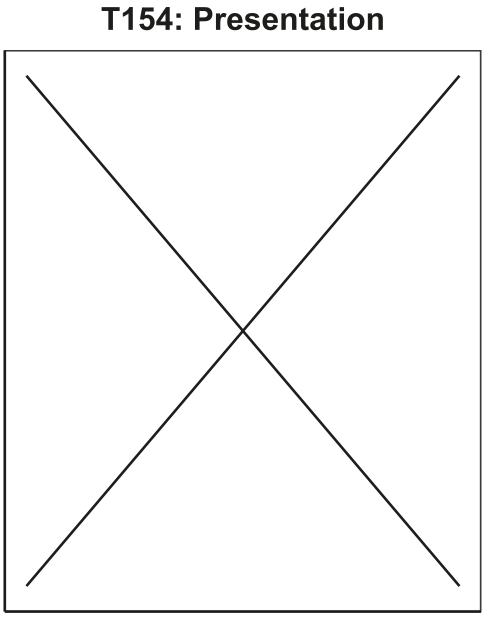
T154: Presentation

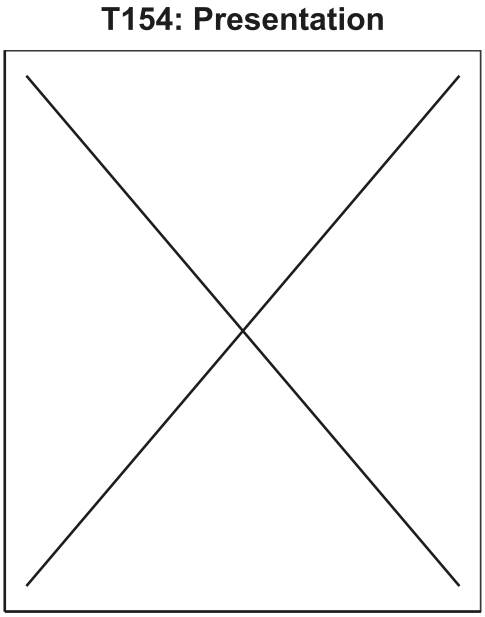
T154: Presentation

```
## ------------------------------------------------------------------------------- ##
```

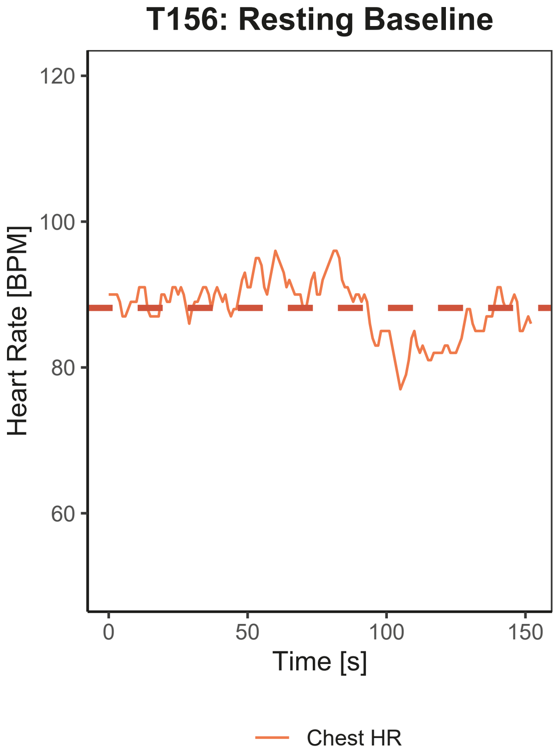
T156: Resting Baseline
Heart Rate [BPM]
120
100
80
60
0
50
100
150
Time [s]
Chest HR

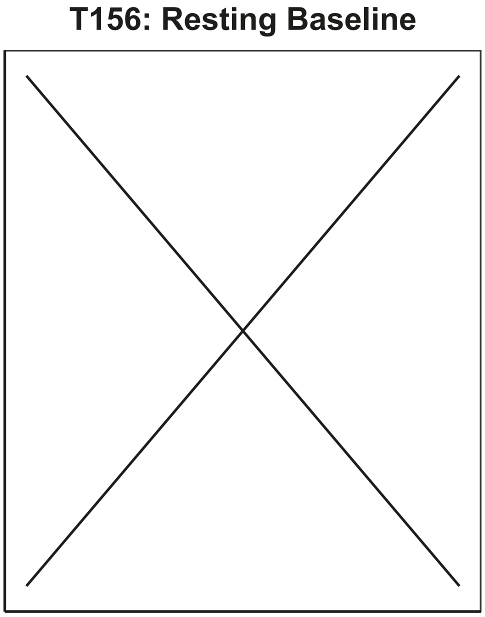
T156: Resting Baseline

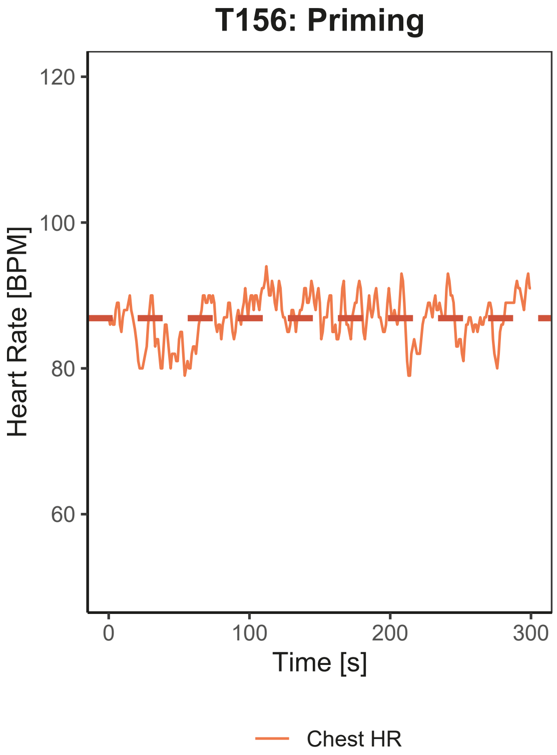
T156: Priming
Heart Rate [BPM]
120
100
80
60
0
100
200
300
Time [s]
Chest HR

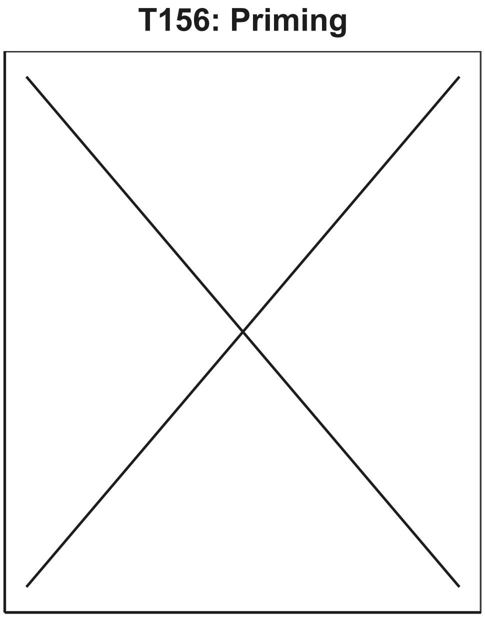
T156: Priming

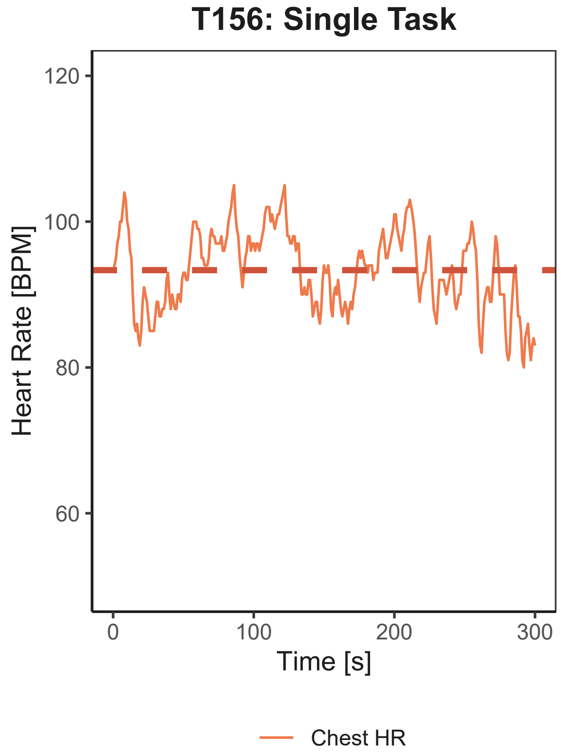
T156: Single Task
Heart Rate [BPM]
120
100
80
60
0
100
200
300
Time [s]
Chest HR

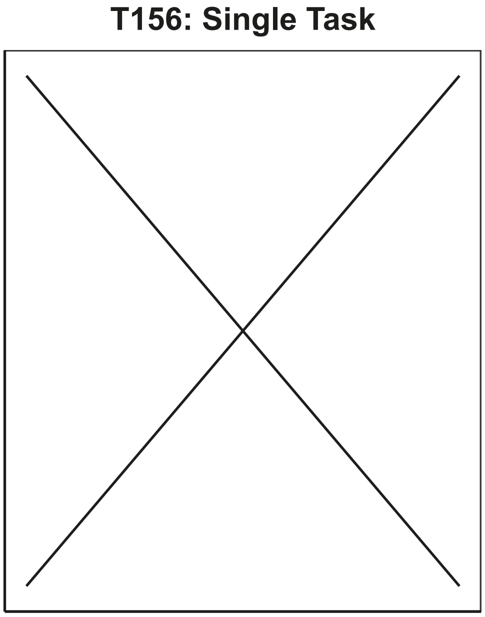
T156: Single Task

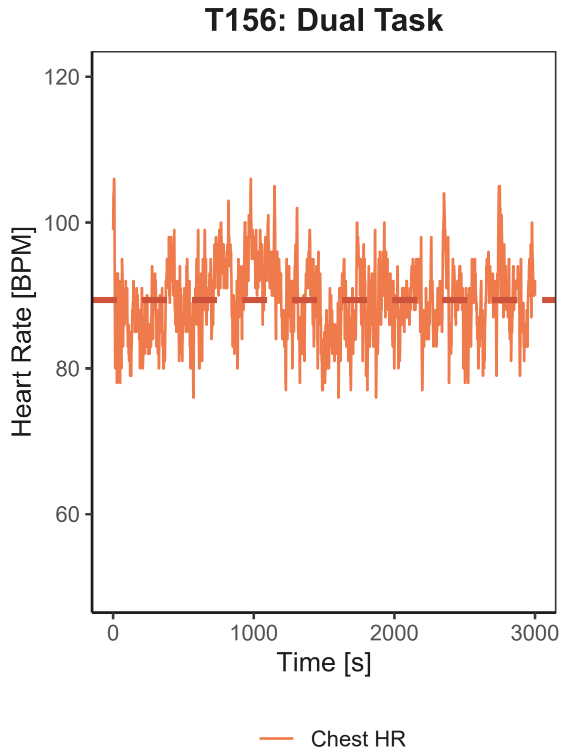
T156: Dual Task
Heart Rate [BPM]
120
100
80
60
0
1000
2000
3000
Time [s]
Chest HR

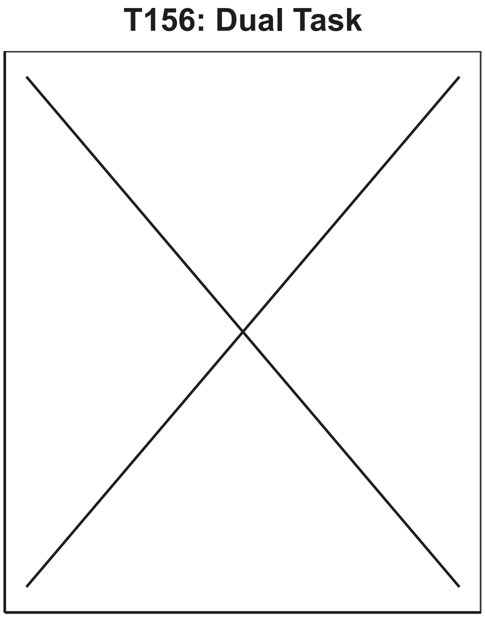
T156: Dual Task

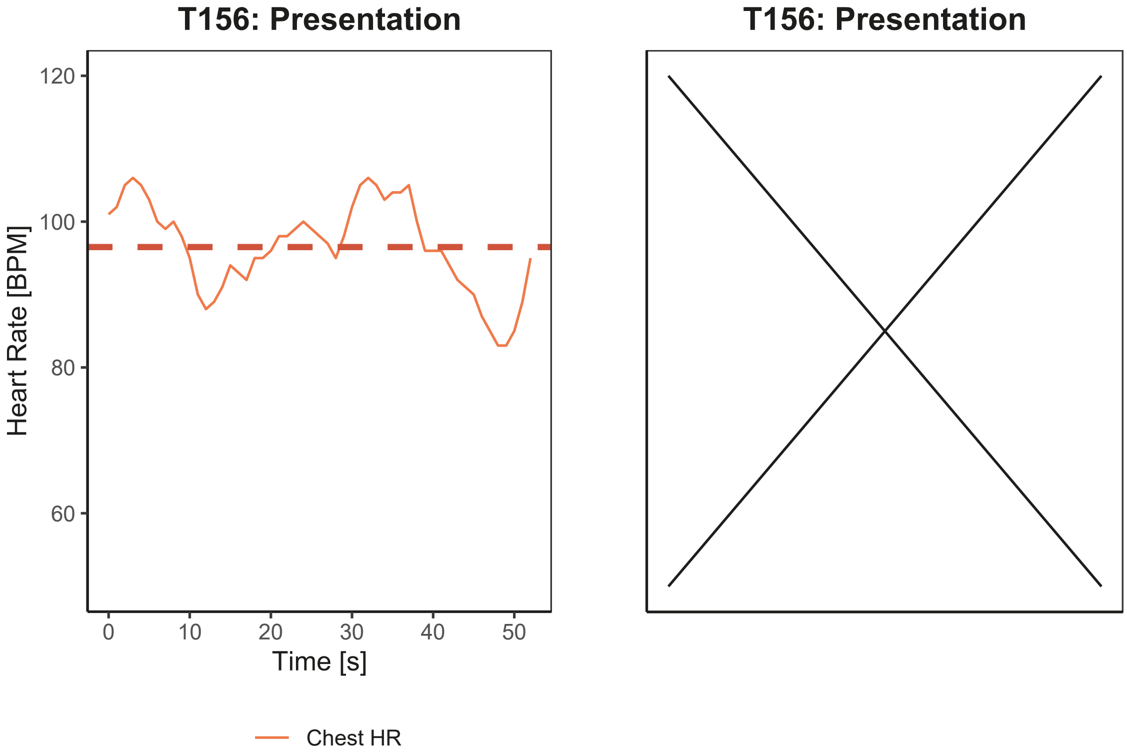
T156: Presentation
Heart Rate [BPM]
120
100
80
60
0
10
20
30
40
50
Time [s]
Chest HR
T156: Presentation

--

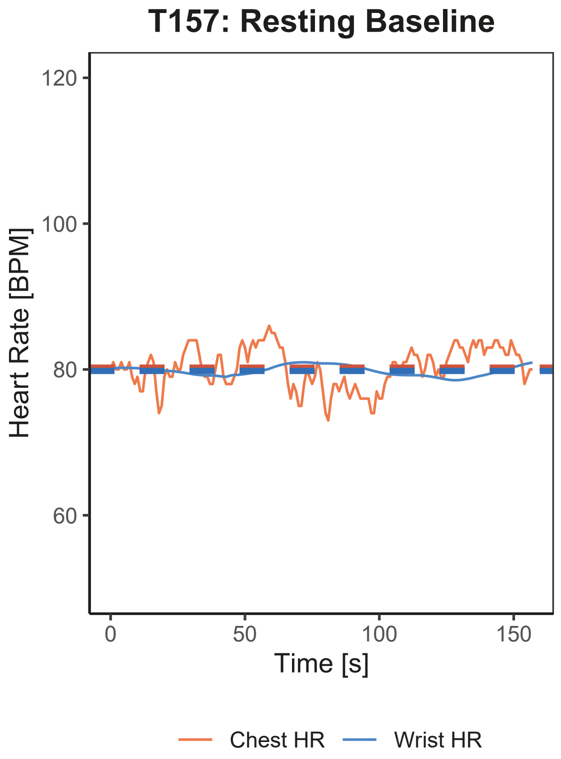
T157: Resting Baseline
Heart Rate [BPM]
120
100
80
60
0
50
100
150
Time [s]
Chest HR
Wrist HR

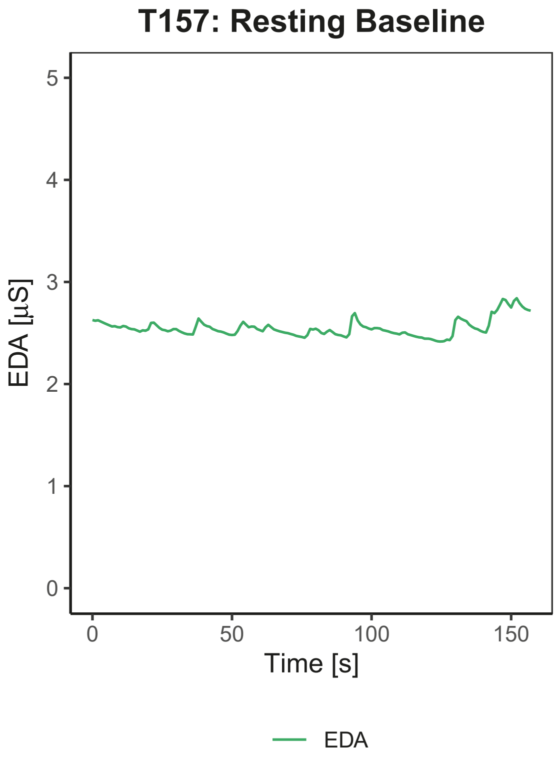
T157: Resting Baseline
EDA [µS]
5
4
3
2
1
0
0
50
100
150
Time [s]
EDA

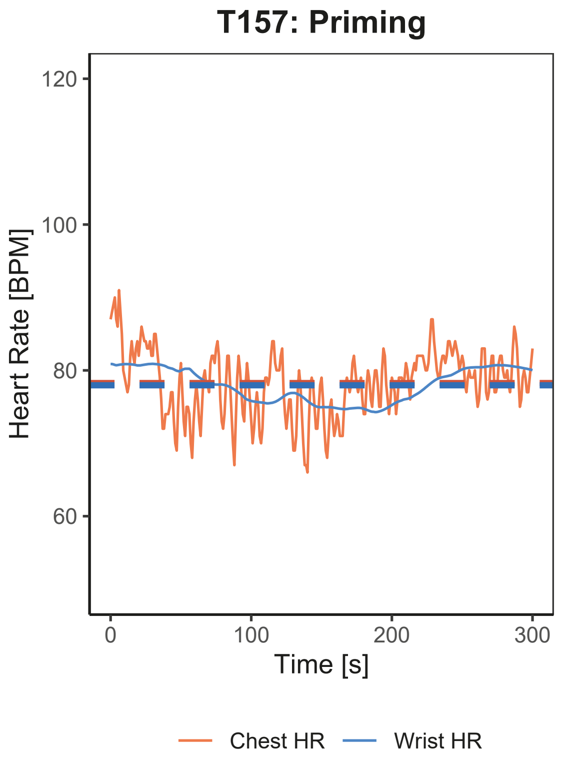
T157: Priming
Heart Rate [BPM]
120
100
80
60
0
100
200
300
Time [s]
Chest HR
Wrist HR

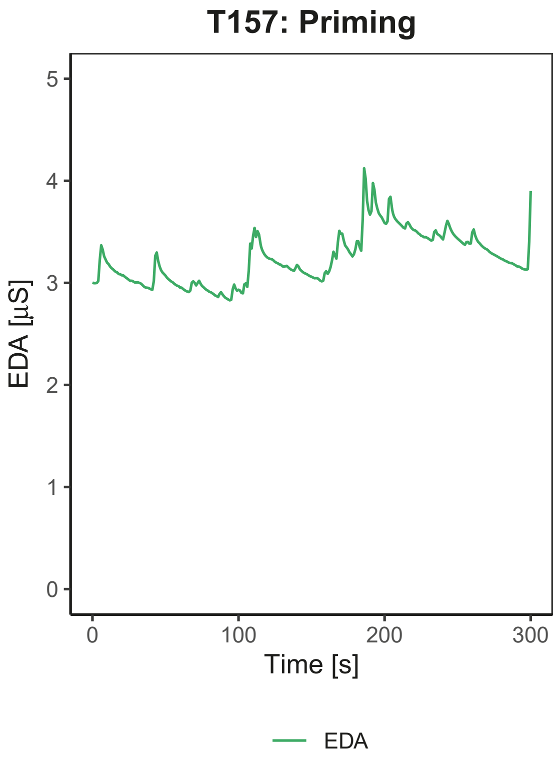
T157: Priming
EDA [µS]
5
4
3
2
1
0
0
100
200
300
Time [s]
EDA

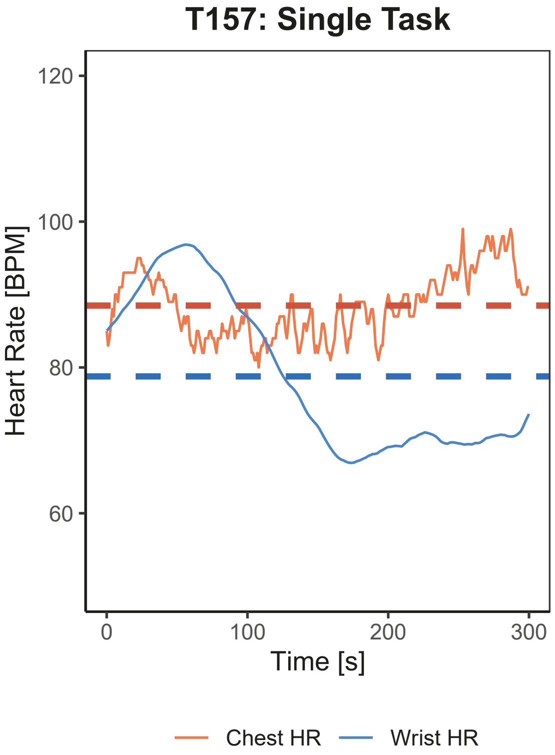
T157: Single Task
Heart Rate [BPM]
120
100
80
60
0
100
200
300
Time [s]
Chest HR
Wrist HR

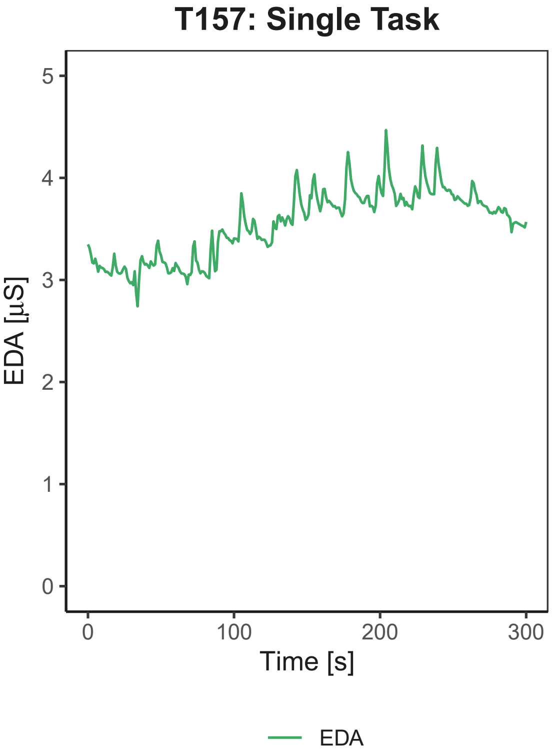
T157: Single Task
EDA [µS]
5
4
3
2
1
0
0
100
200
300
Time [s]
EDA

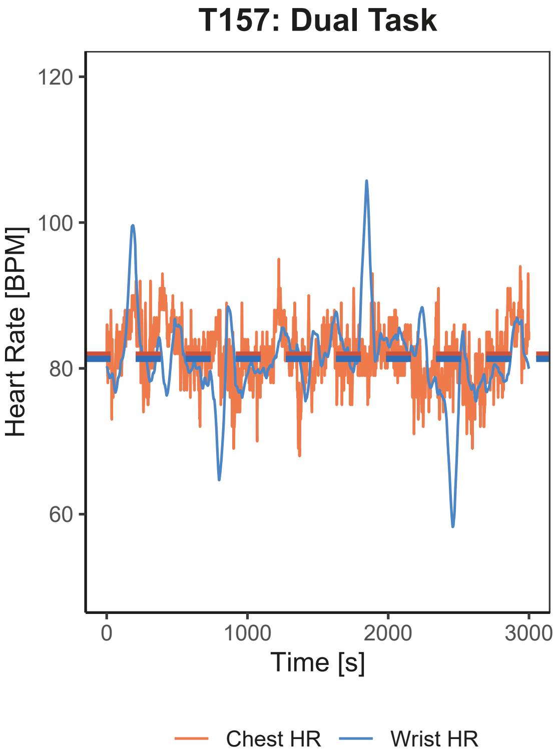
T157: Dual Task
Heart Rate [BPM]
120
100
80
60
0
1000
2000
3000
Time [s]
Chest HR
Wrist HR

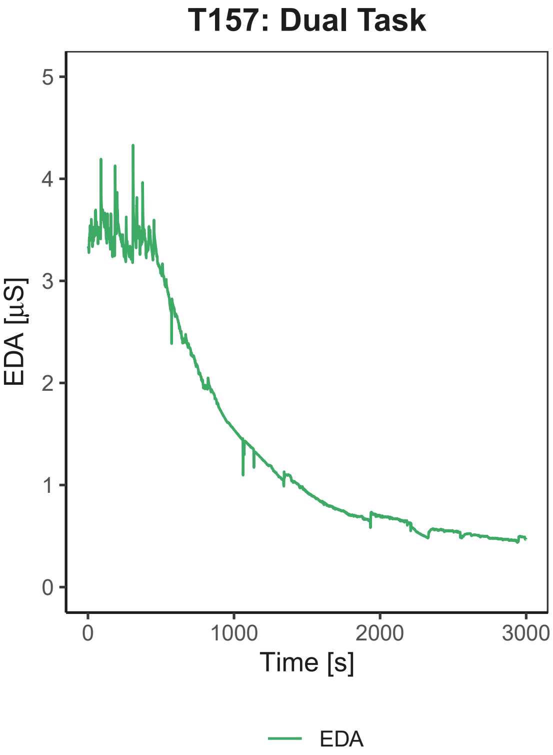
T157: Dual Task
EDA [µS]
5
4
3
2
1
0
0
1000
2000
3000
Time [s]
EDA

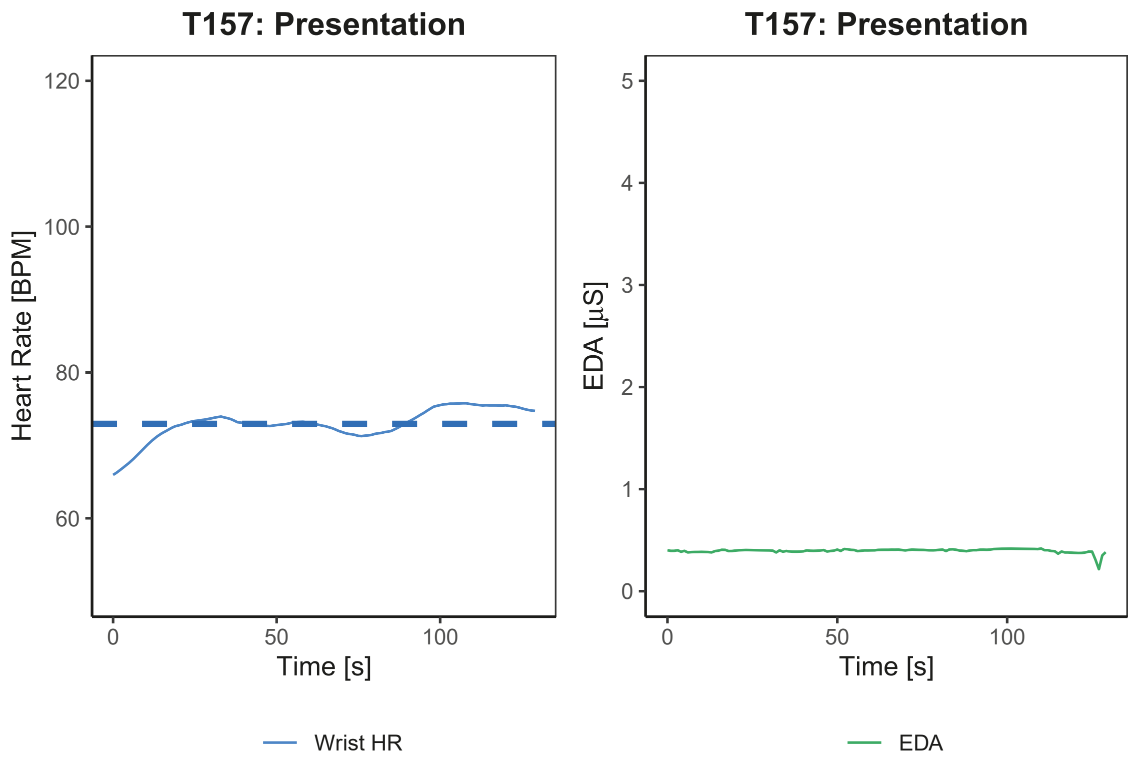
T157: Presentation
Heart Rate [BPM]
120
100
80
60
0
50
100
Time [s]
Wrist HR
T157: Presentation
EDA [µS]
5
4
3
2
1
0
0
50
100
Time [s]
EDA

--

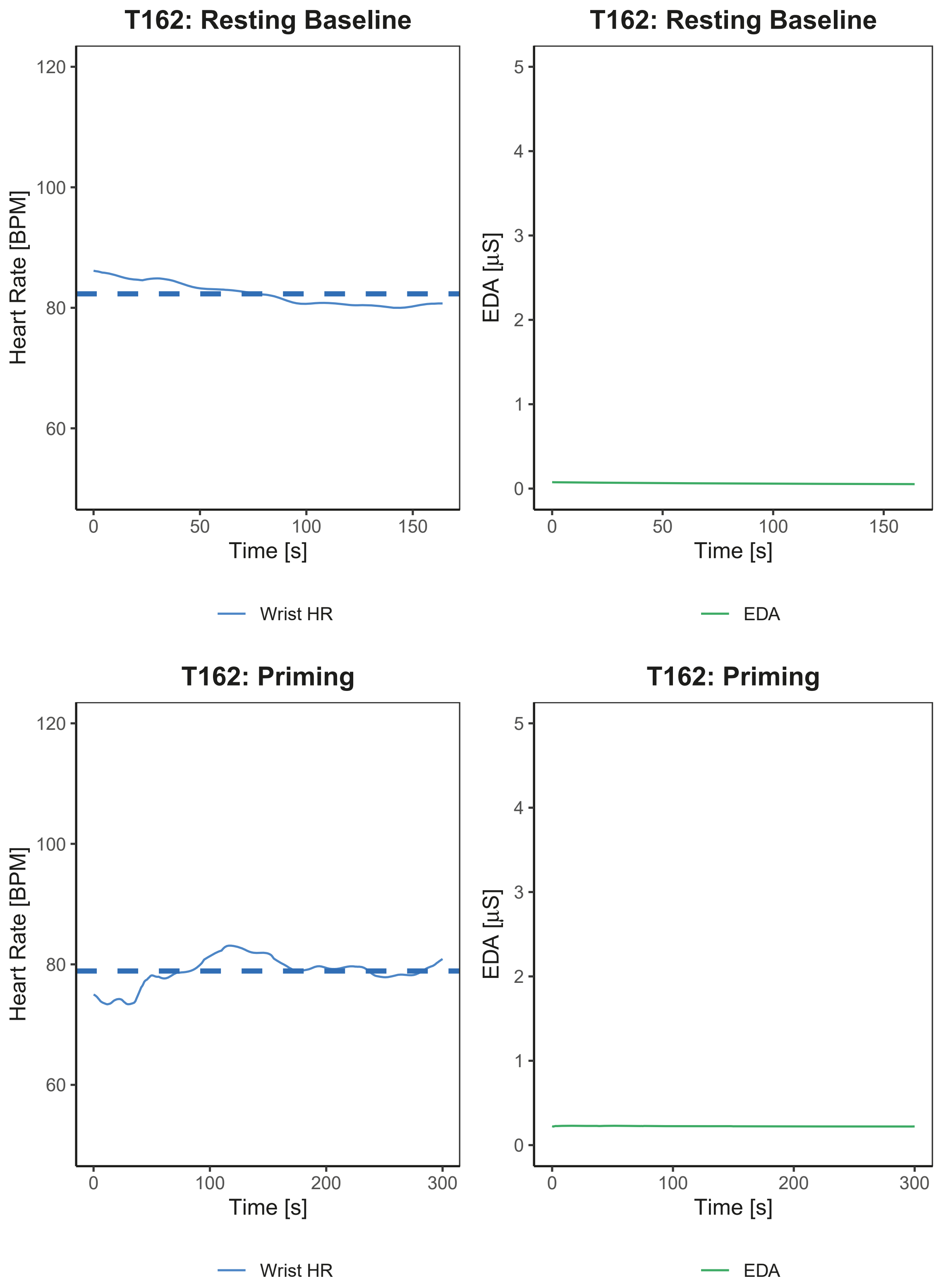
T162: Resting Baseline
Heart Rate [BPM]
120
100
80
60
0
50
100
150
Time [s]
Wrist HR
T162: Resting Baseline
EDA [µS]
5
4
3
2
1
0
0
50
100
150
Time [s]
EDA
T162: Priming
Heart Rate [BPM]
120
100
80
60
0
100
200
300
Time [s]
Wrist HR
T162: Priming
EDA [µS]
5
4
3
2
1
0
0
100
200
300
Time [s]
EDA

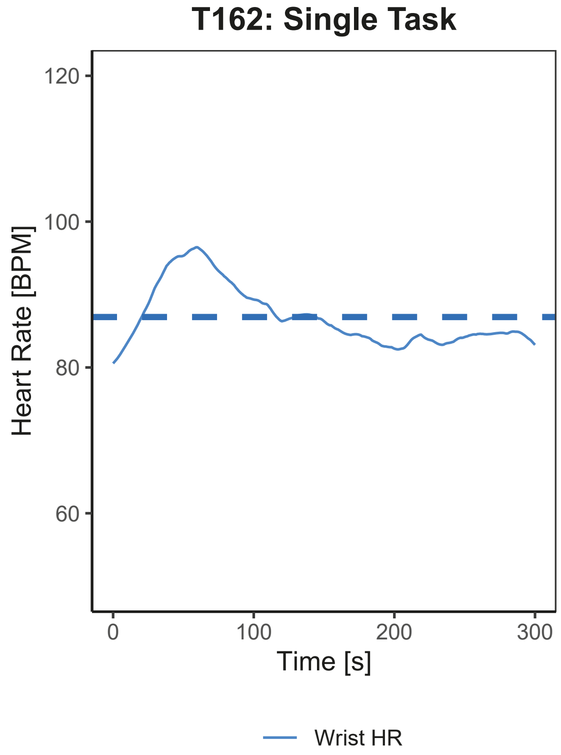
T162: Single Task
Heart Rate [BPM]
120
100
80
60
0
100
200
300
Time [s]
Wrist HR

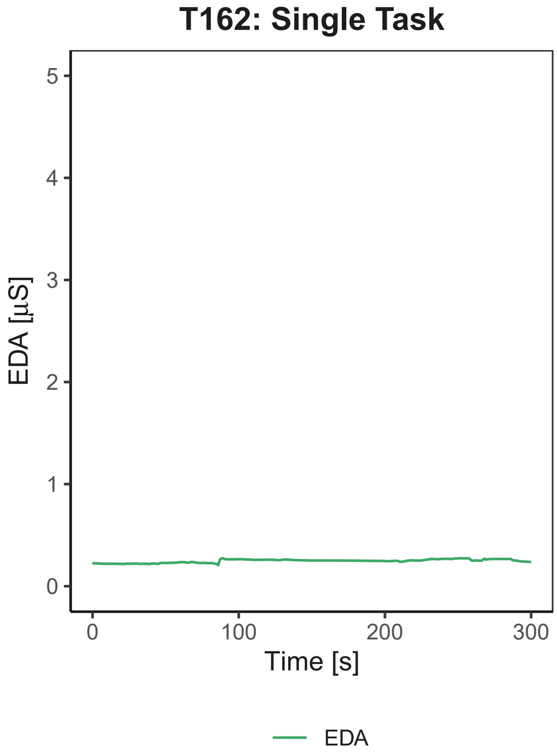
T162: Single Task
EDA [µS]
5
4
3
2
1
0
0
100
200
300
Time [s]
EDA

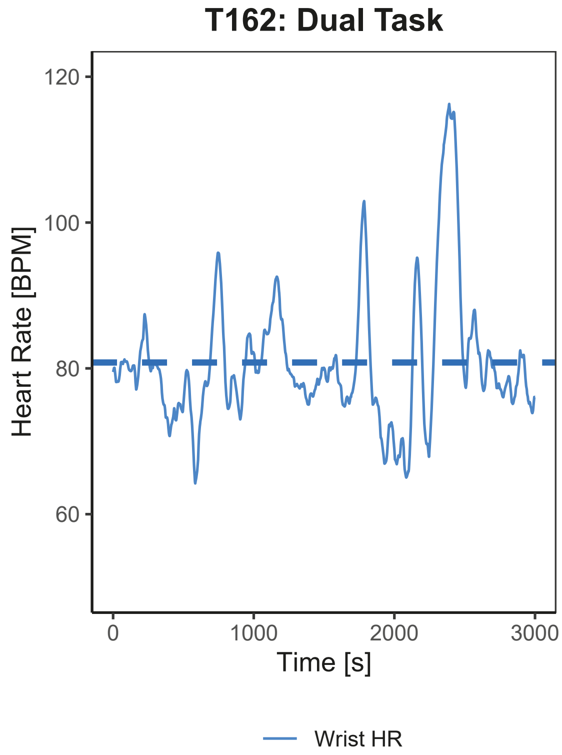
T162: Dual Task
Heart Rate [BPM]
120
100
80
60
0
1000
2000
3000
Time [s]
Wrist HR

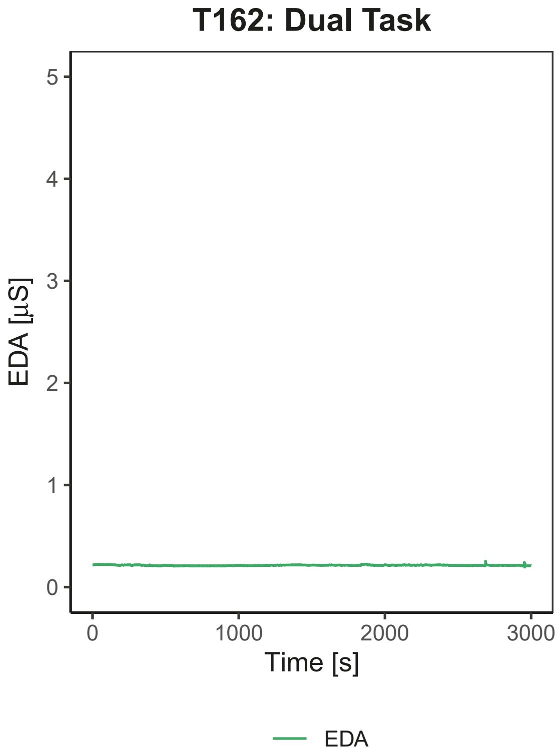
T162: Dual Task
EDA [µS]
5
4
3
2
1
0
0
1000
2000
3000
Time [s]
EDA

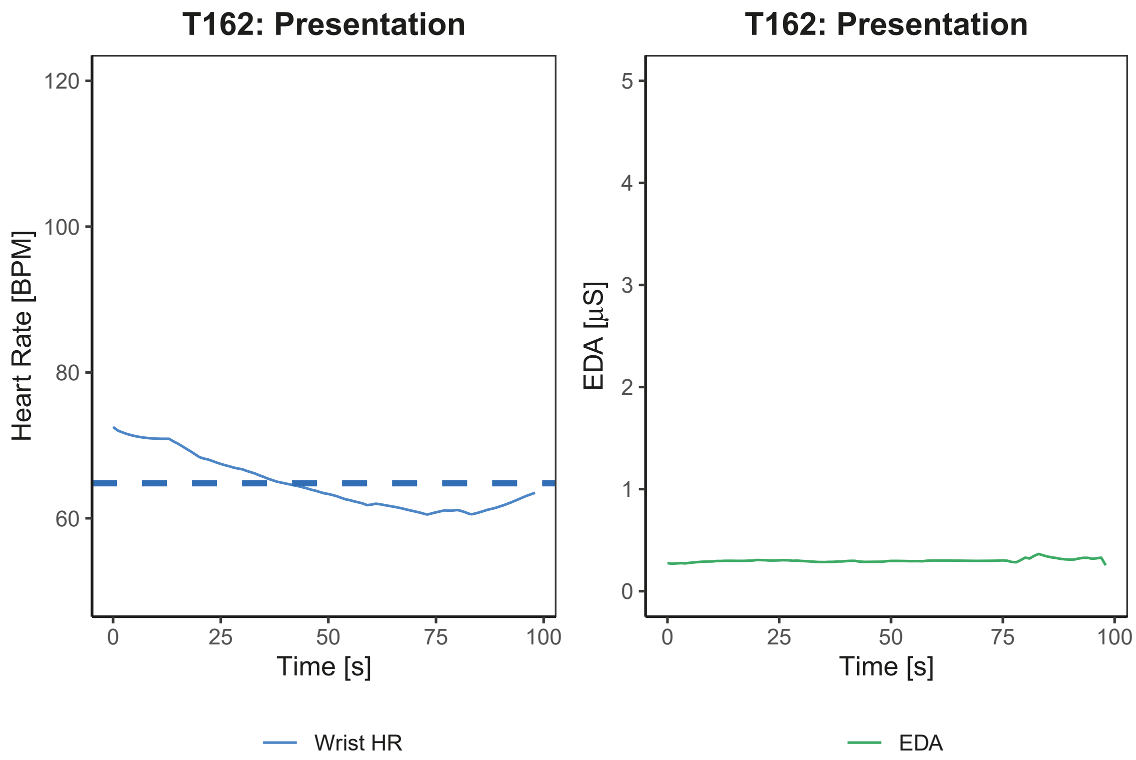
T162: Presentation
Heart Rate [BPM]
120
100
80
60
0
25
50
75
100
Time [s]
Wrist HR
T162: Presentation
EDA [µS]
5
4
3
2
1
0
0
25
50
75
100
Time [s]
EDA

--

T166: Resting Baseline

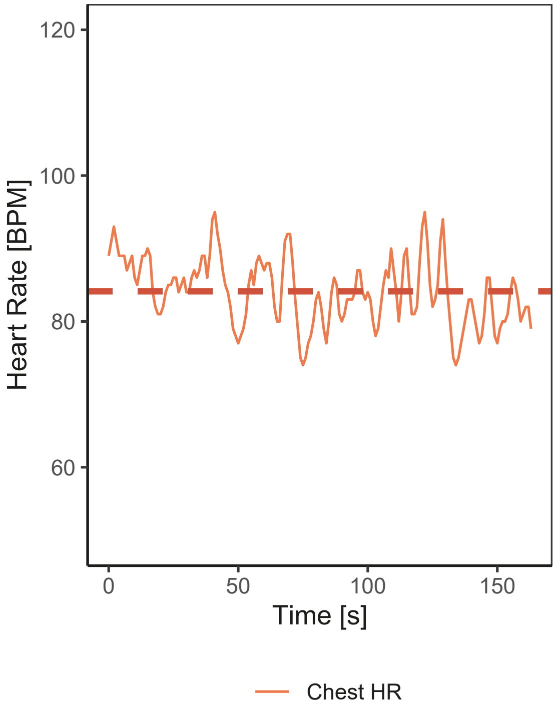

T166: Resting Baseline

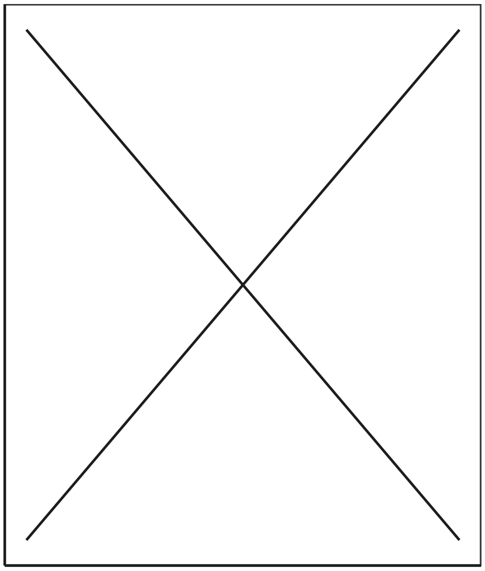

T166: Priming

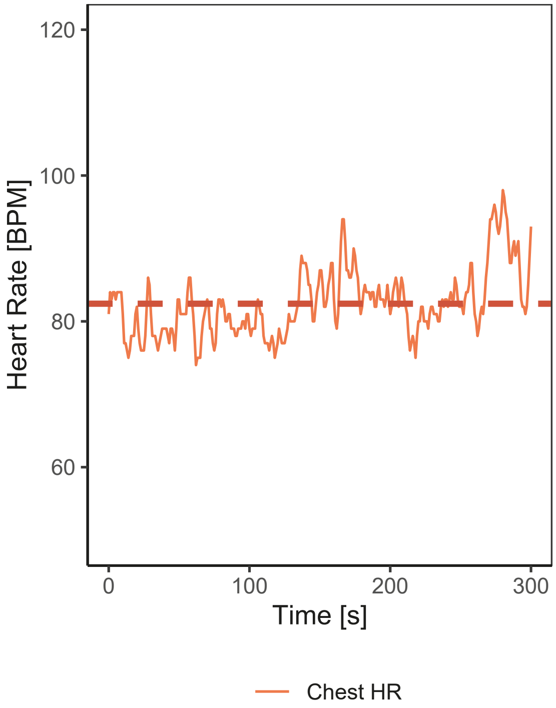

T166: Priming

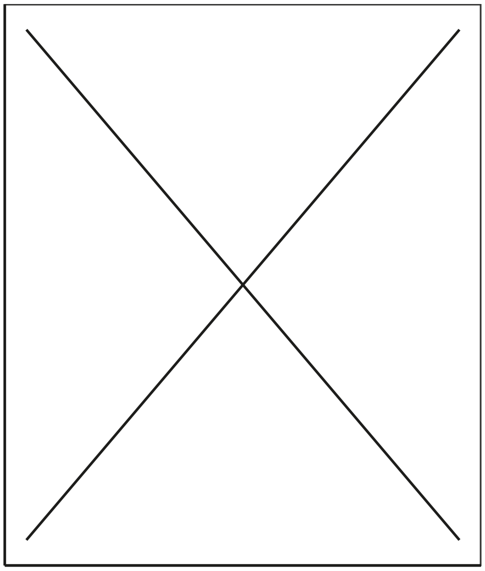

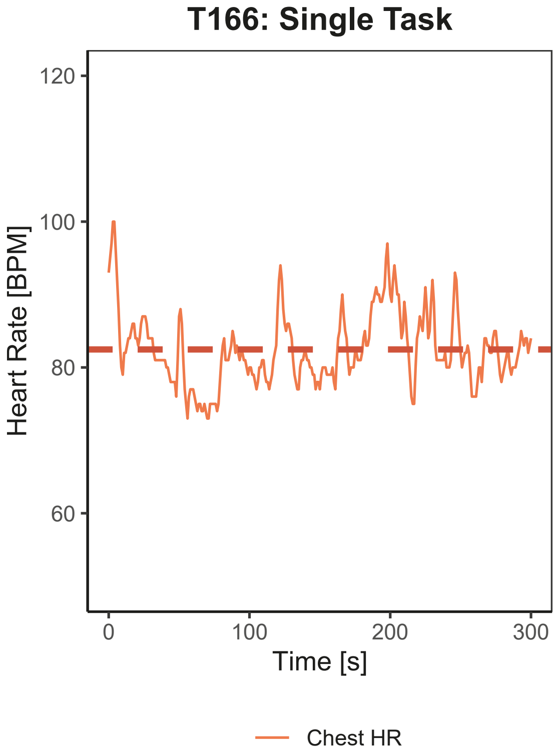
T166: Single Task
Heart Rate [BPM]
120
100
80
60
0
100
200
300
Time [s]
Chest HR

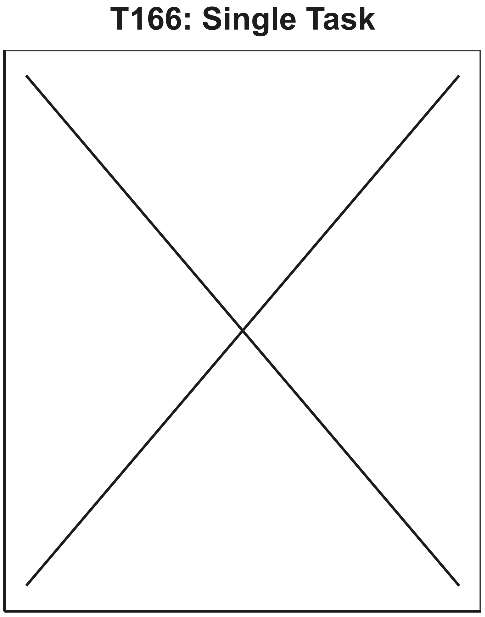
T166: Single Task

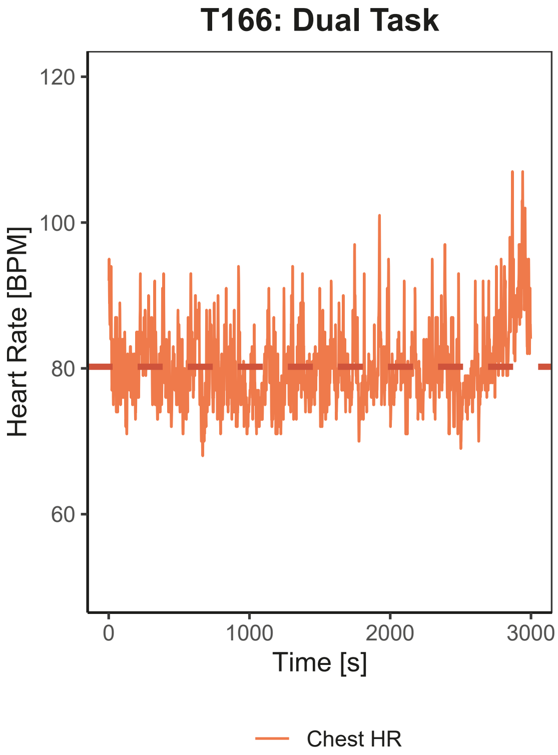
T166: Dual Task
Heart Rate [BPM]
120
100
80
60
0
1000
2000
3000
Time [s]
Chest HR

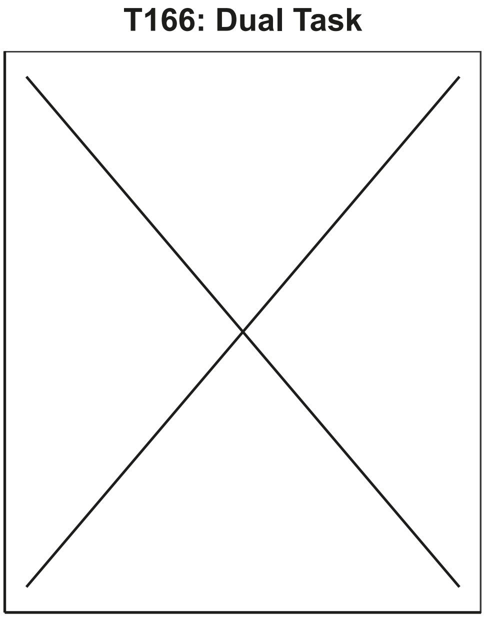
T166: Dual Task

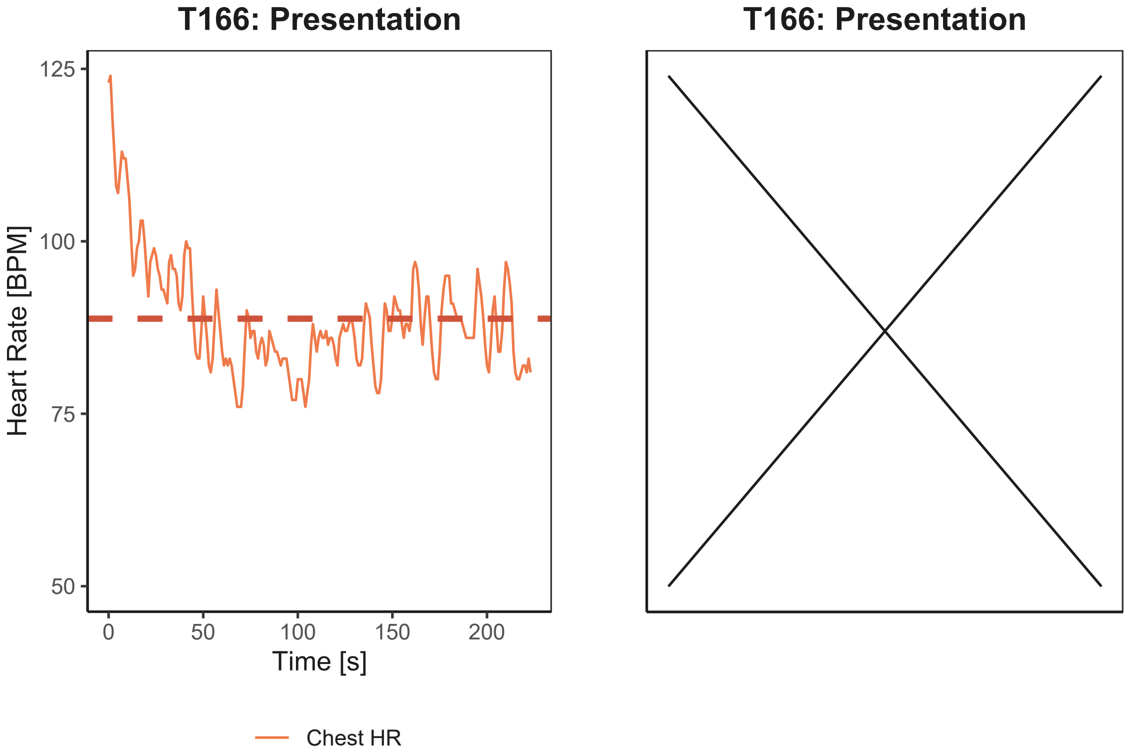
T166: Presentation
T166: Presentation
Heart Rate [BPM]
125
100
75
50
0
50
100
150
200
Time [s]
Chest HR

--

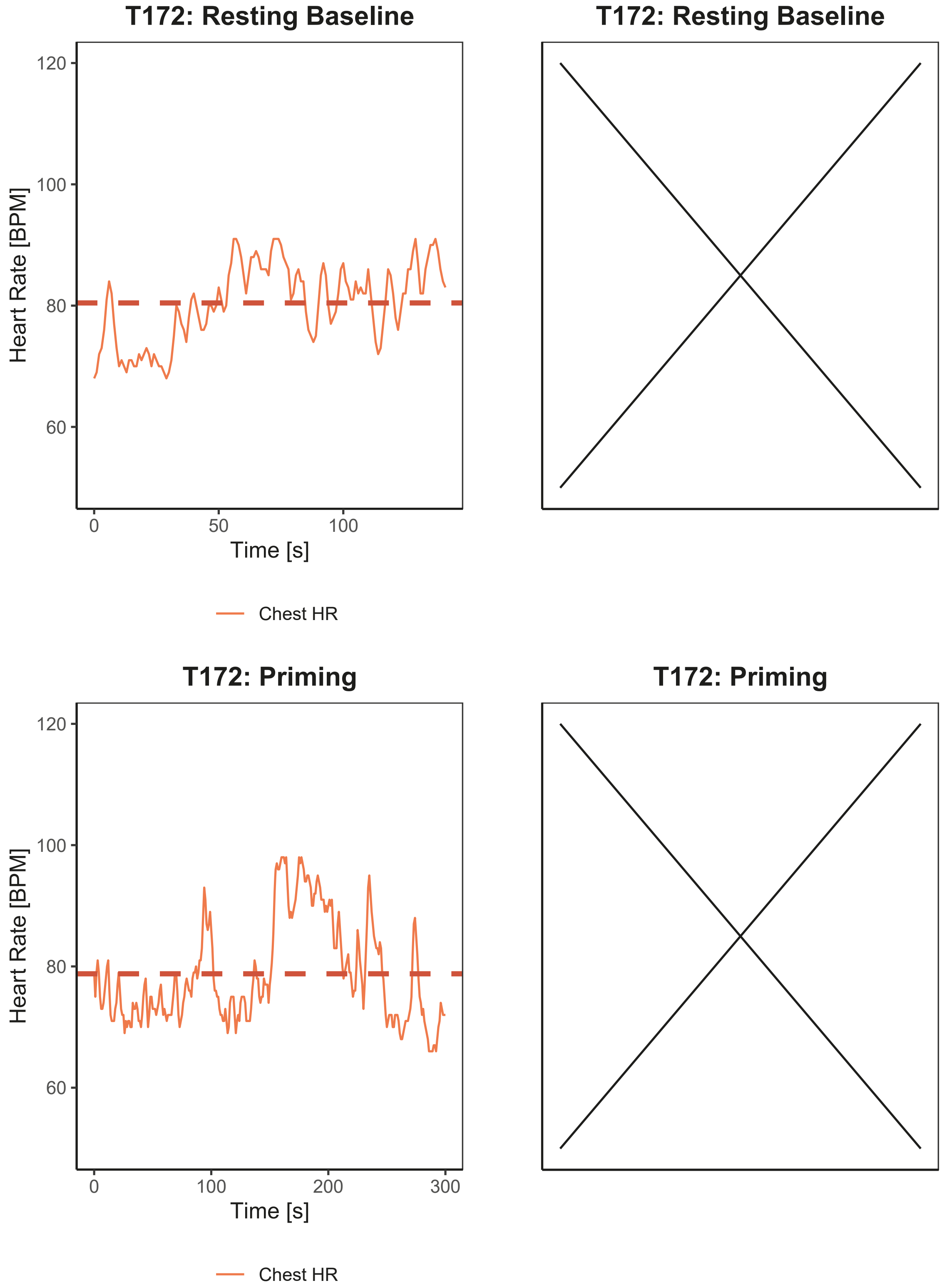
T172: Resting Baseline
Heart Rate [BPM]
120
100
80
60
0
50
100
Time [s]
Chest HR
T172: Resting Baseline
T172: Priming
Heart Rate [BPM]
120
100
80
60
0
100
200
300
Time [s]
Chest HR
T172: Priming

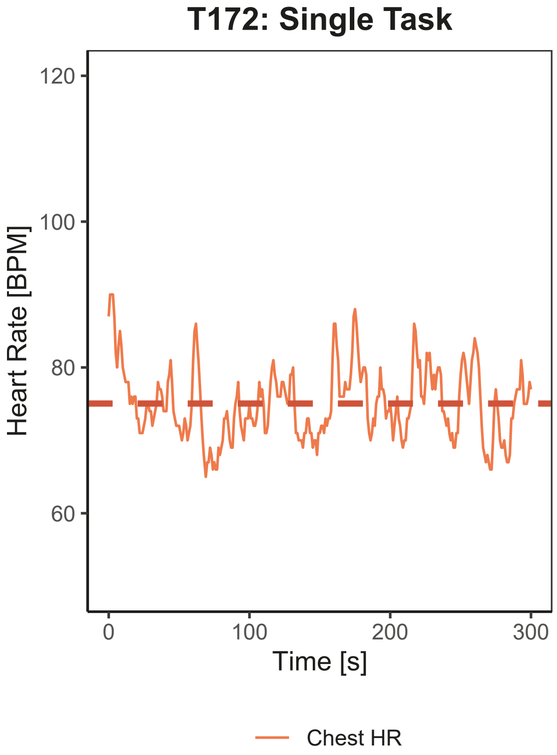
T172: Single Task
Heart Rate [BPM]
120
100
80
60
0
100
200
300
Time [s]
Chest HR

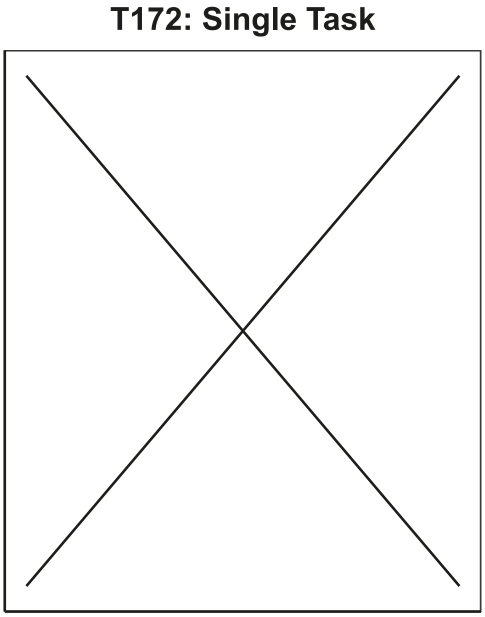
T172: Single Task

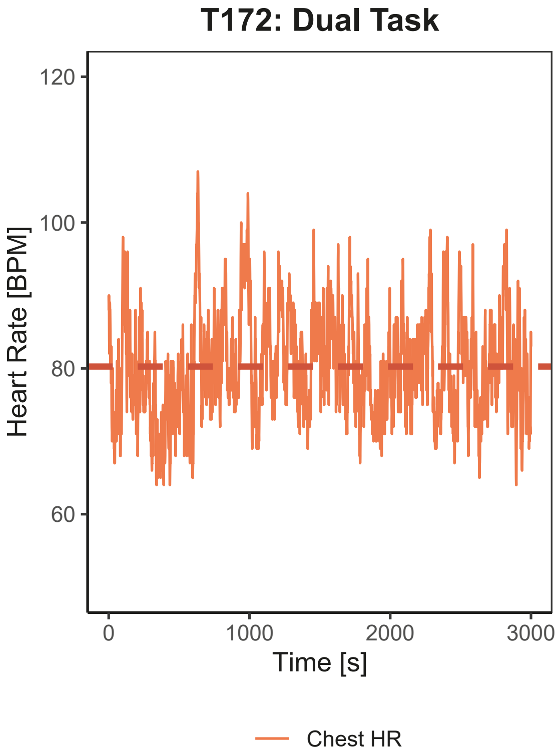
T172: Dual Task
Heart Rate [BPM]
120
100
80
60
0
1000
2000
3000
Time [s]
Chest HR

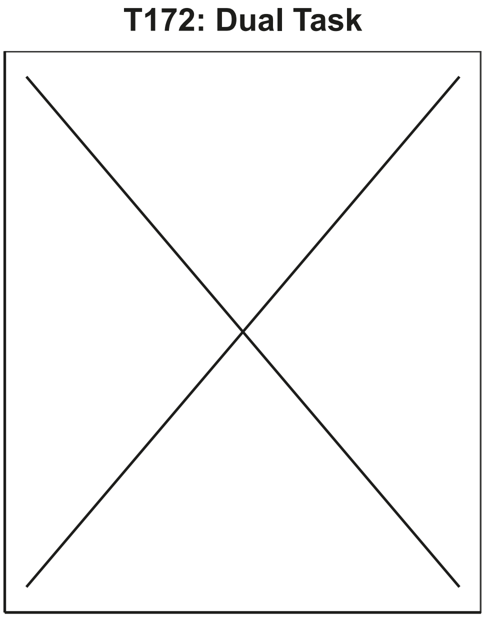
T172: Dual Task

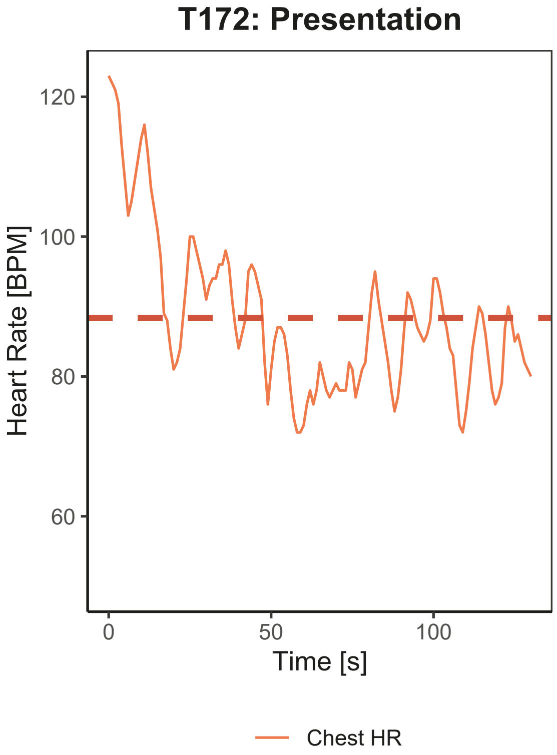

T172: Presentation
Heart Rate [BPM]
120
100
80
60
0
50
100
Time [s]
Chest HR

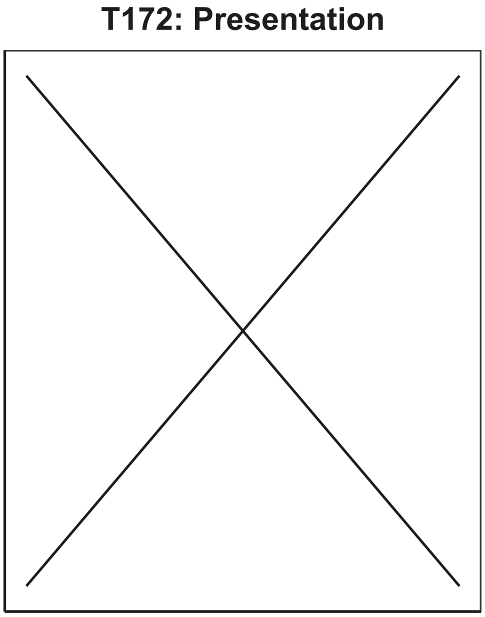

T172: Presentation

--

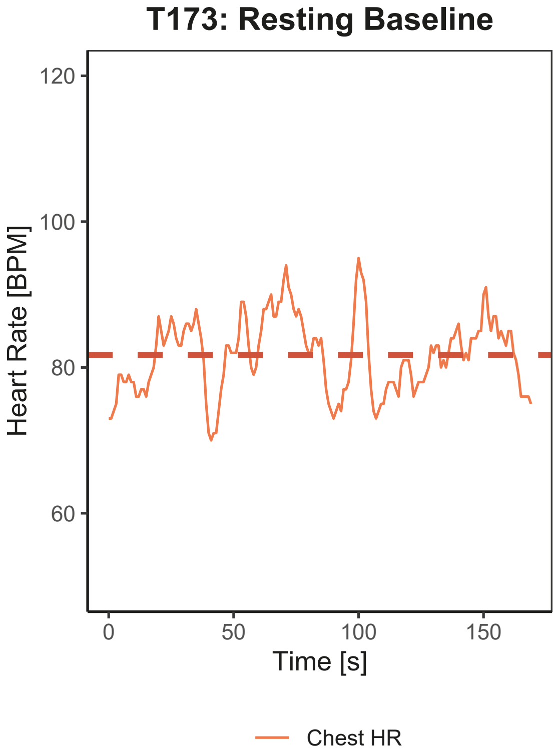
T173: Resting Baseline
Heart Rate [BPM]
120
100
80
60
0
50
100
150
Time [s]
Chest HR

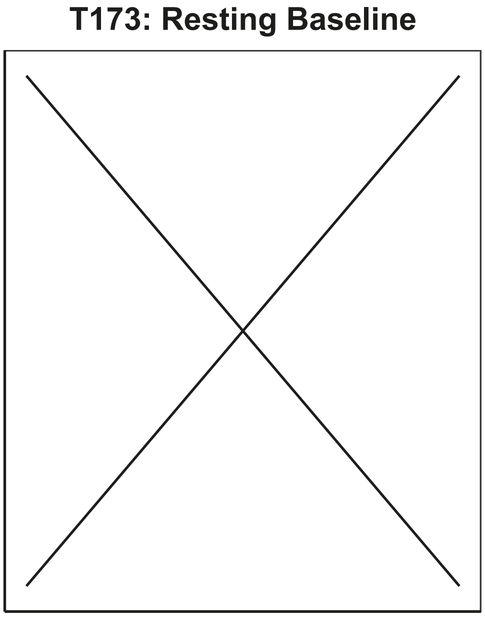
T173: Resting Baseline

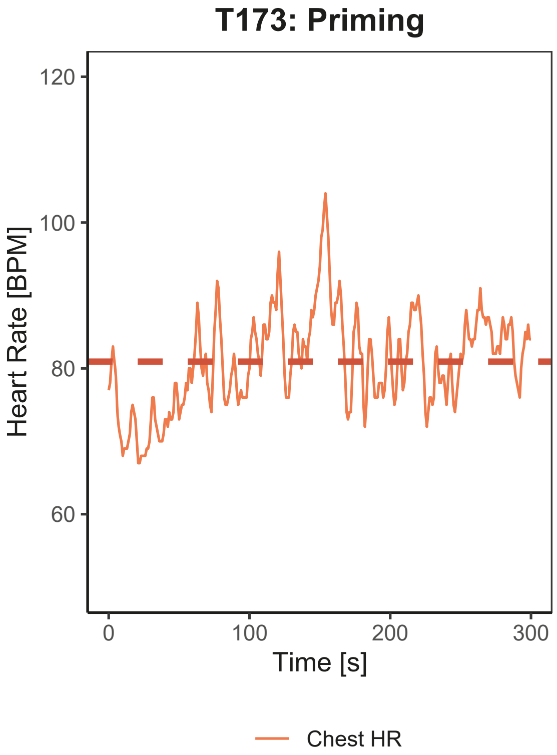
T173: Priming
Heart Rate [BPM]
120
100
80
60
0
100
200
300
Time [s]
Chest HR

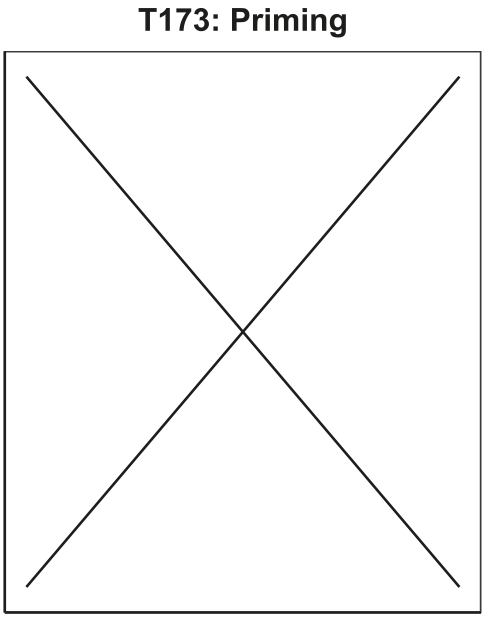
T173: Priming

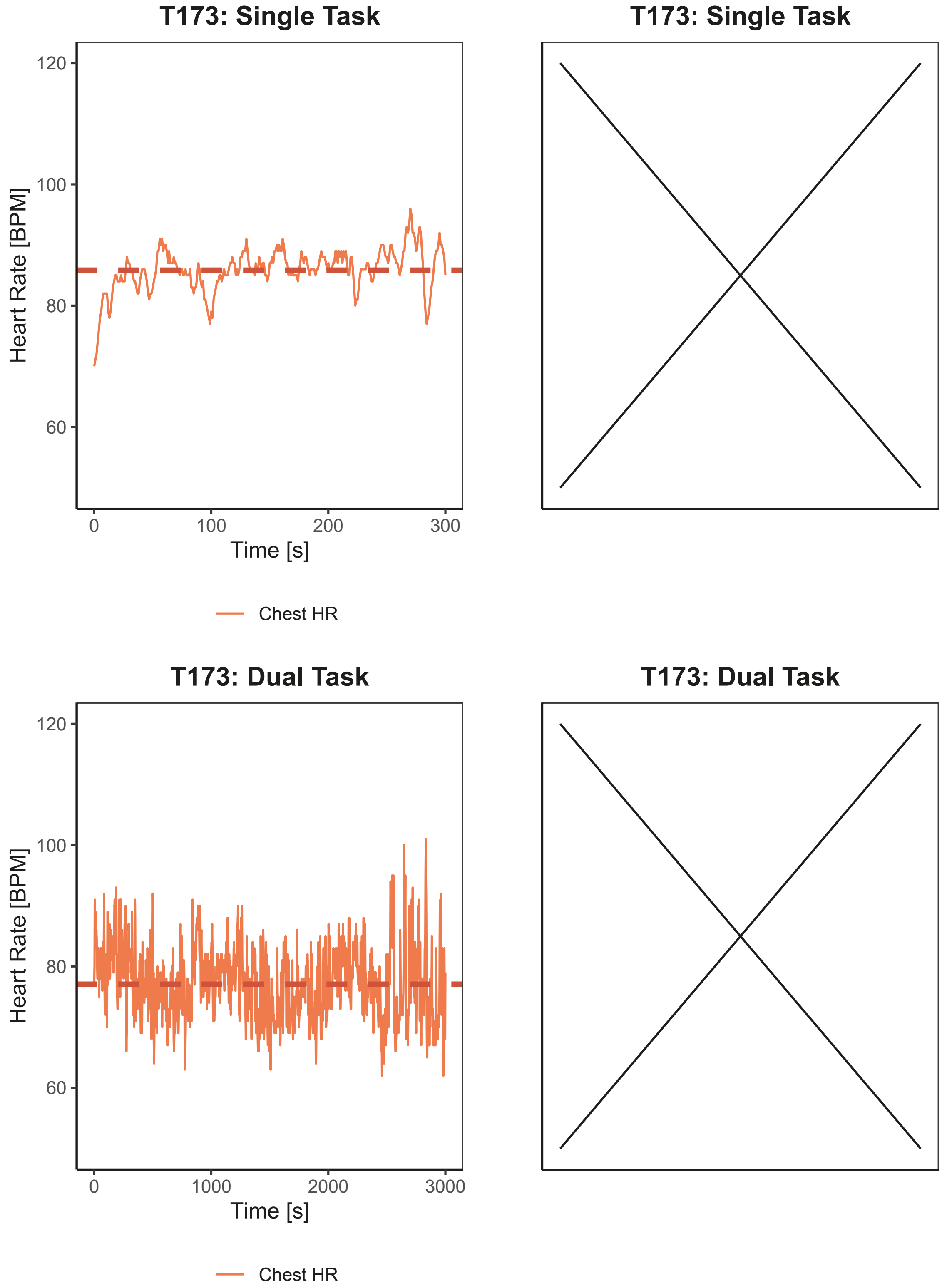

T173: Single Task
Heart Rate [BPM]
120
100
80
60
0
100
200
300
Time [s]
Chest HR
T173: Single Task
T173: Dual Task
Heart Rate [BPM]
120
100
80
60
0
1000
2000
3000
Time [s]
Chest HR
T173: Dual Task

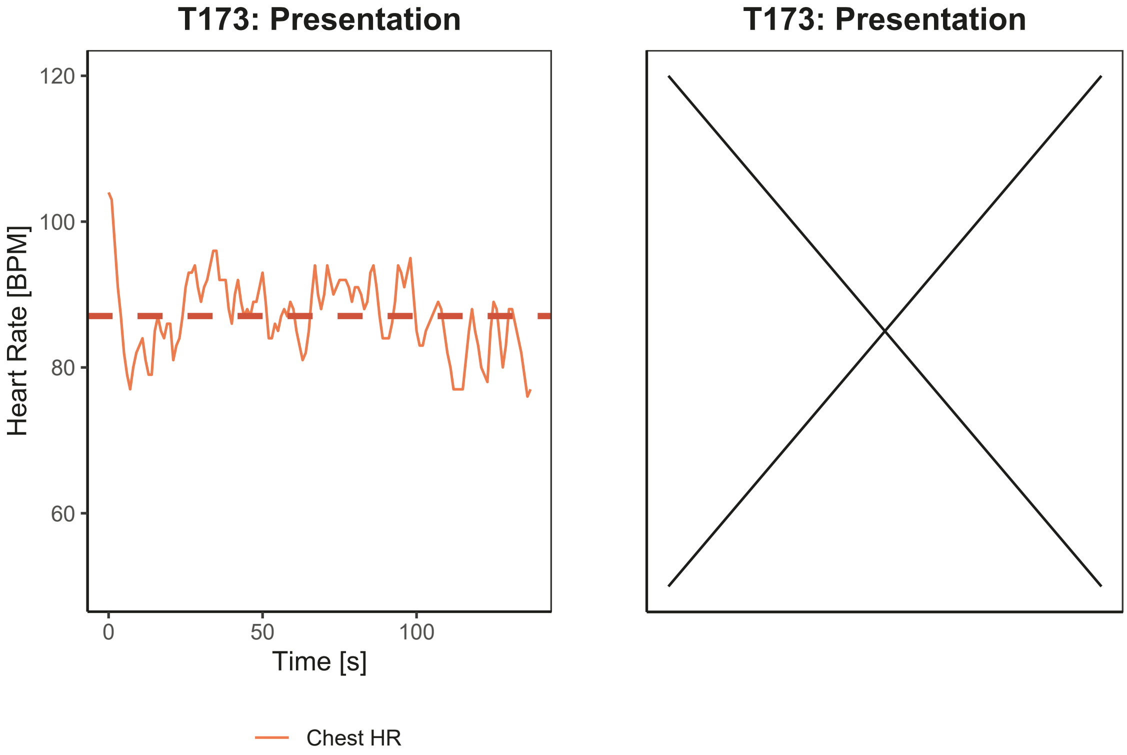
T173: Presentation
Heart Rate [BPM]
120
100
80
60
0
50
100
Time [s]
Chest HR
T173: Presentation

--

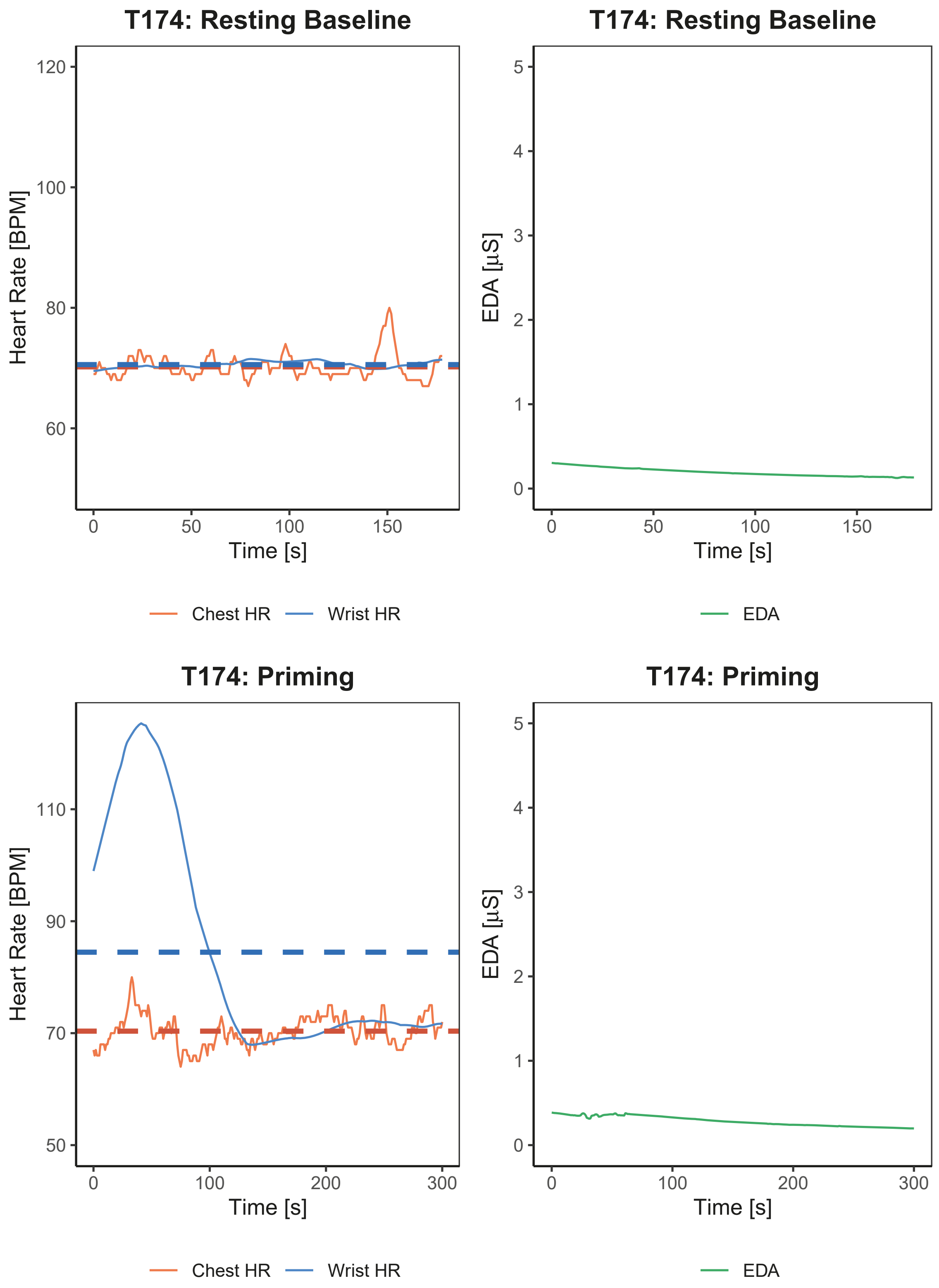
T174: Resting Baseline
Heart Rate [BPM]
120
100
80
60
0
50
100
150
Time [s]
Chest HR
Wrist HR
T174: Resting Baseline
EDA [µS]
5
4
3
2
1
0
0
50
100
150
Time [s]
EDA
T174: Priming
Heart Rate [BPM]
110
90
70
50
0
100
200
300
Time [s]
Chest HR
Wrist HR
T174: Priming
EDA [µS]
5
4
3
2
1
0
0
100
200
300
Time [s]
EDA

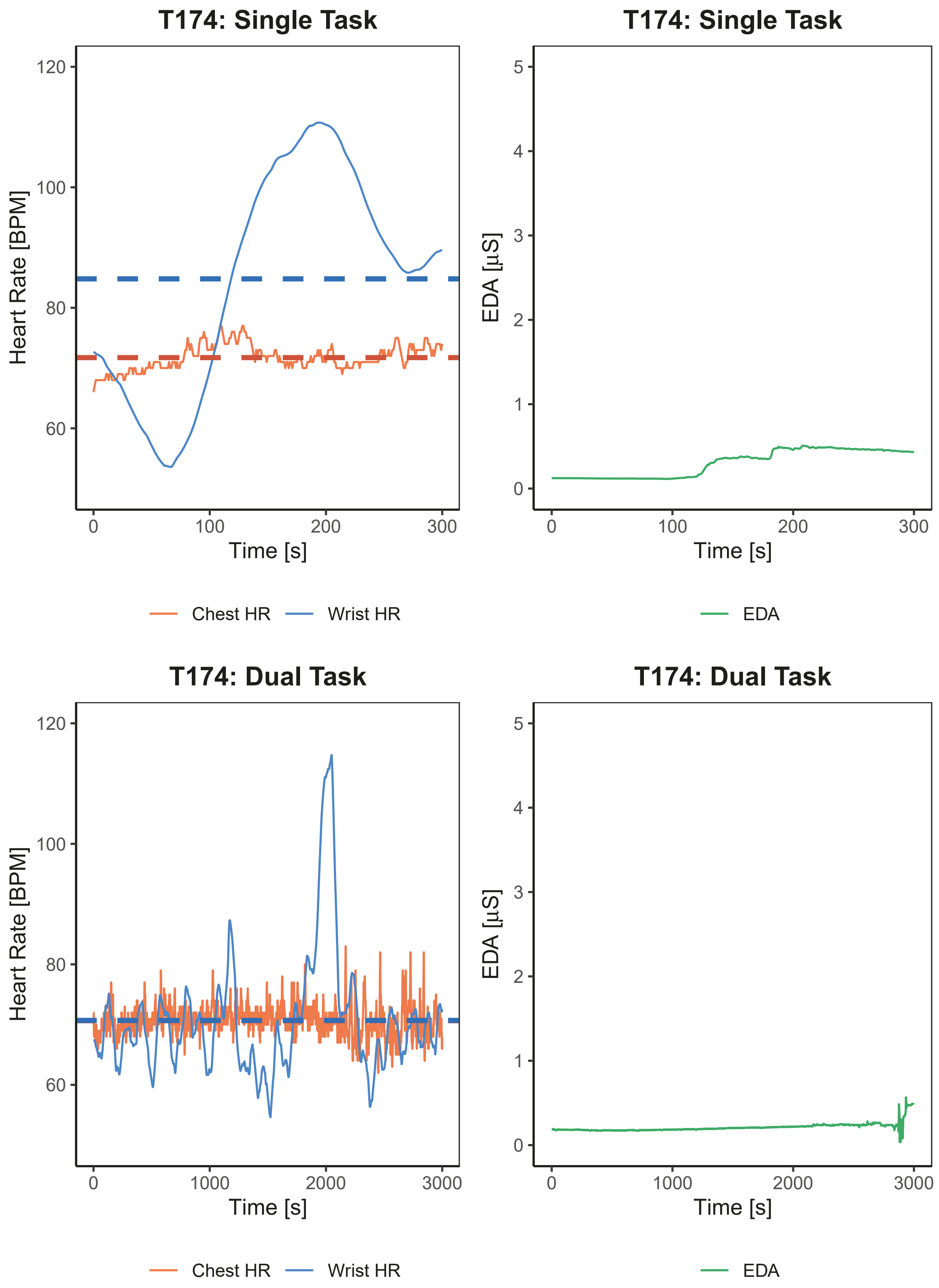
T174: Single Task
Heart Rate [BPM]
120
100
80
60
0
100
200
300
Time [s]
Chest HR
Wrist HR
T174: Single Task
EDA [µS]
5
4
3
2
1
0
0
100
200
300
Time [s]
EDA
T174: Dual Task
Heart Rate [BPM]
120
100
80
60
0
1000
2000
3000
Time [s]
Chest HR
Wrist HR
T174: Dual Task
EDA [µS]
5
4
3
2
1
0
0
1000
2000
3000
Time [s]
EDA

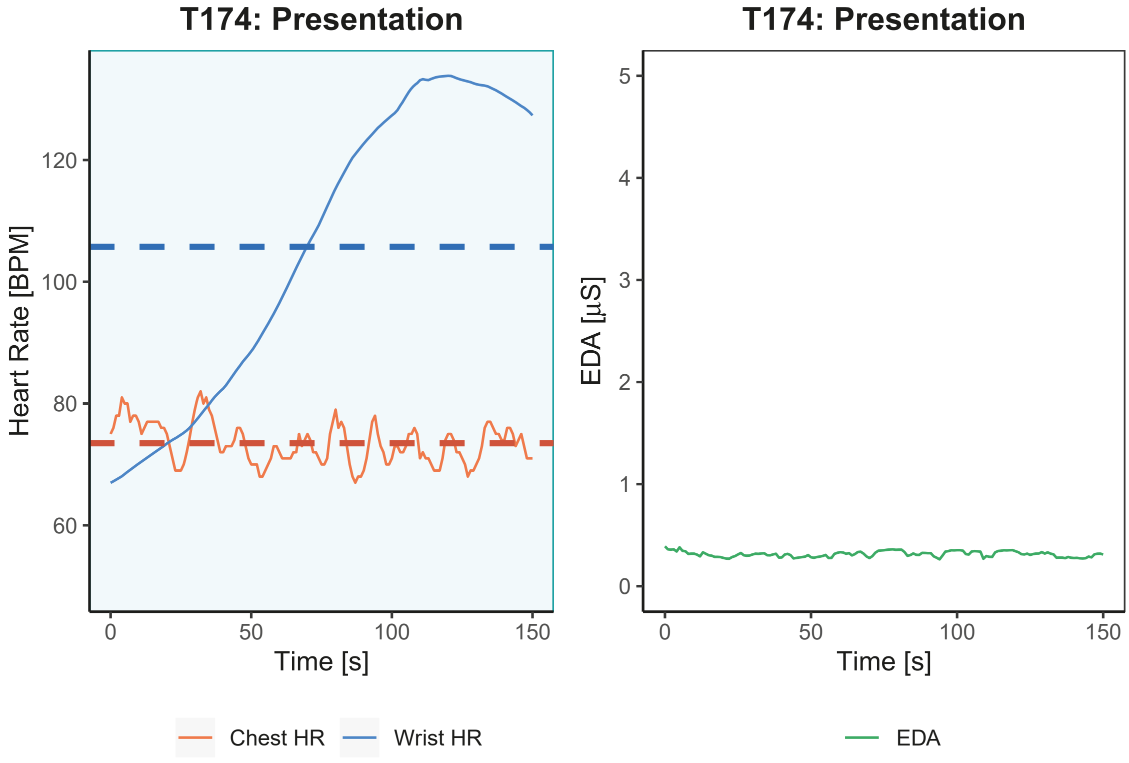
T174: Presentation
Heart Rate [BPM]
120
100
80
60
0
50
100
150
Time [s]
T174: Presentation
EDA [µS]
5
4
3
2
1
0
0
50
100
150
Time [s]
Chest HR
Wrist HR
EDA

--

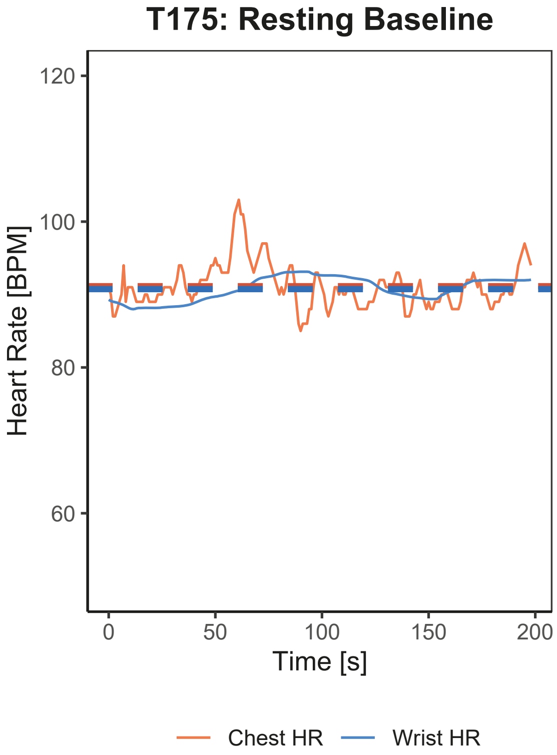
T175: Resting Baseline
Heart Rate [BPM]
120
100
80
60
0
50
100
150
200
Time [s]
Chest HR
Wrist HR

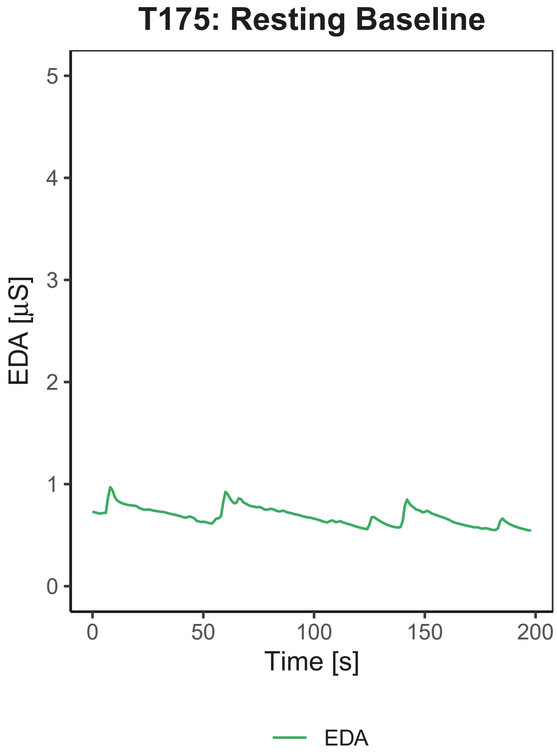
T175: Resting Baseline
EDA [µS]
5
4
3
2
1
0
0
50
100
150
200
Time [s]
EDA

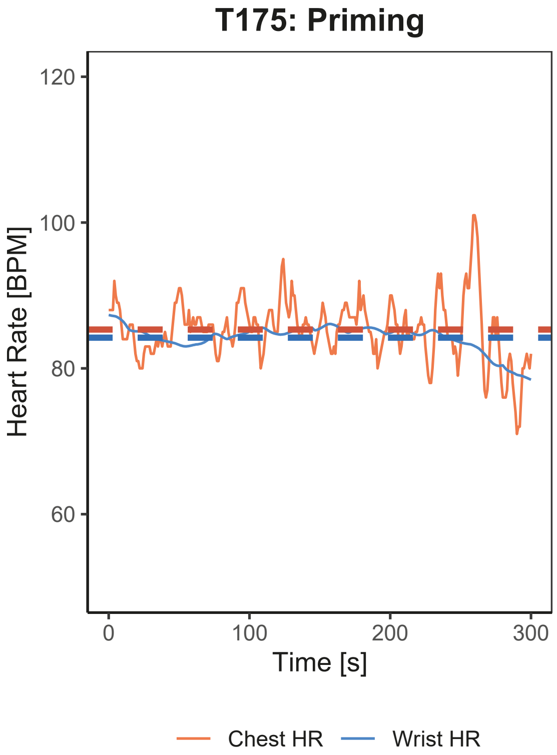
T175: Priming
Heart Rate [BPM]
120
100
80
60
0
100
200
300
Time [s]
Chest HR
Wrist HR

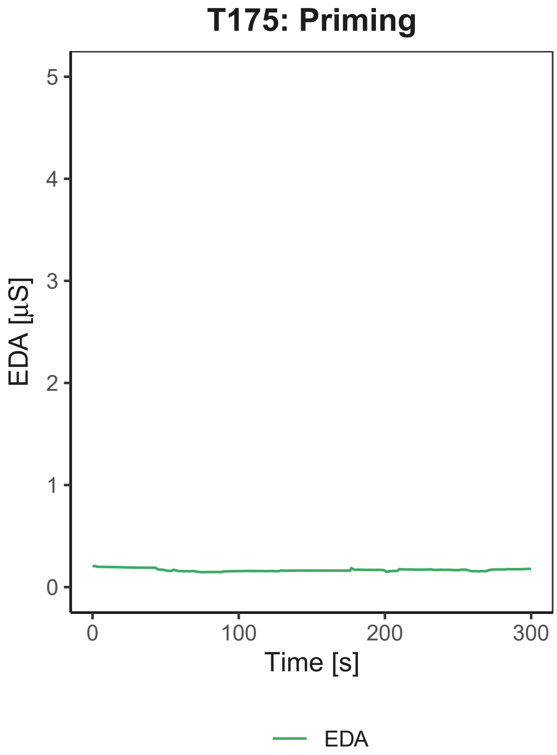
T175: Priming
EDA [µS]
5
4
3
2
1
0
0
100
200
300
Time [s]
EDA

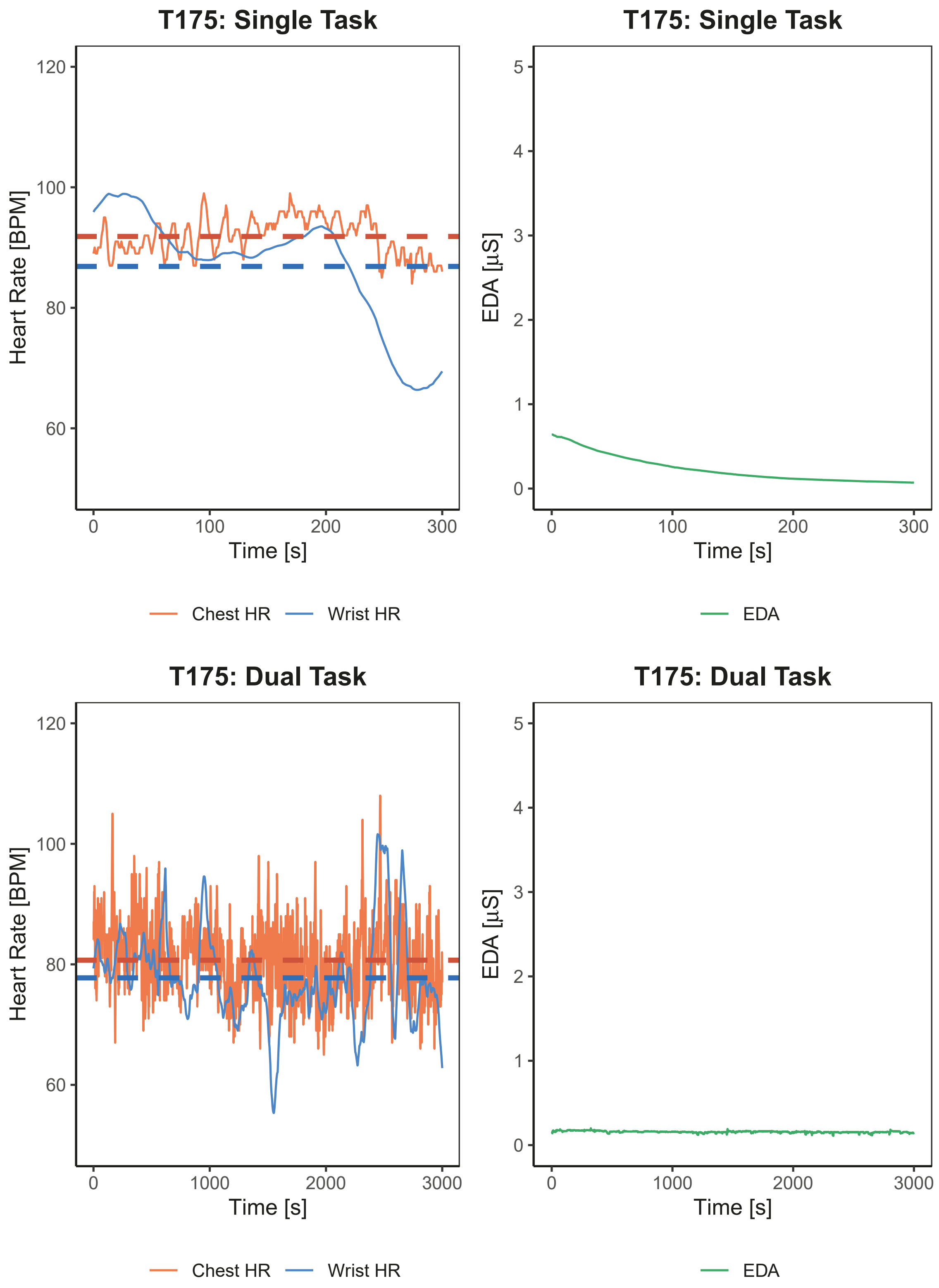
T175: Single Task
Heart Rate [BPM]
120
100
80
60
0
100
200
300
Time [s]
Chest HR
Wrist HR
T175: Single Task
EDA [µS]
5
4
3
2
1
0
0
100
200
300
Time [s]
EDA
T175: Dual Task
Heart Rate [BPM]
120
100
80
60
0
1000
2000
3000
Time [s]
Chest HR
Wrist HR
T175: Dual Task
EDA [µS]
5
4
3
2
1
0
0
1000
2000
3000
Time [s]
EDA

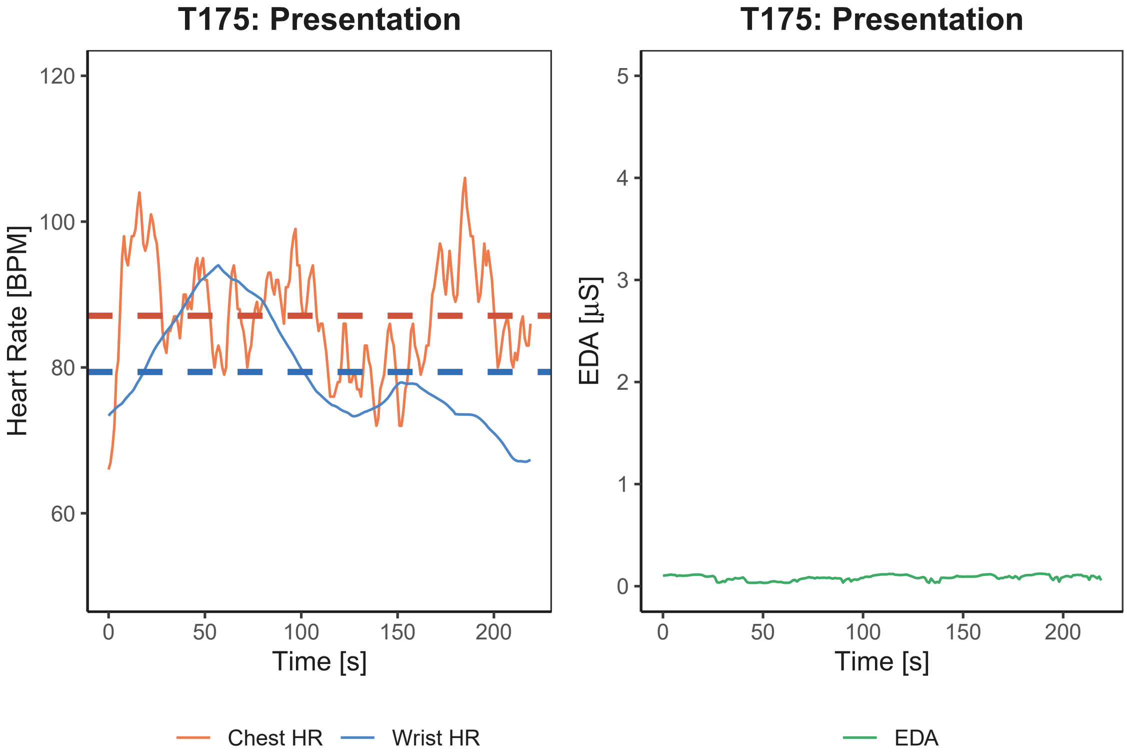
T175: Presentation
Heart Rate [BPM]
120
100
80
60
0
50
100
150
200
Time [s]
T175: Presentation
EDA [µS]
5
4
3
2
1
0
0
50
100
150
200
Time [s]
Chest HR
Wrist HR
EDA

--

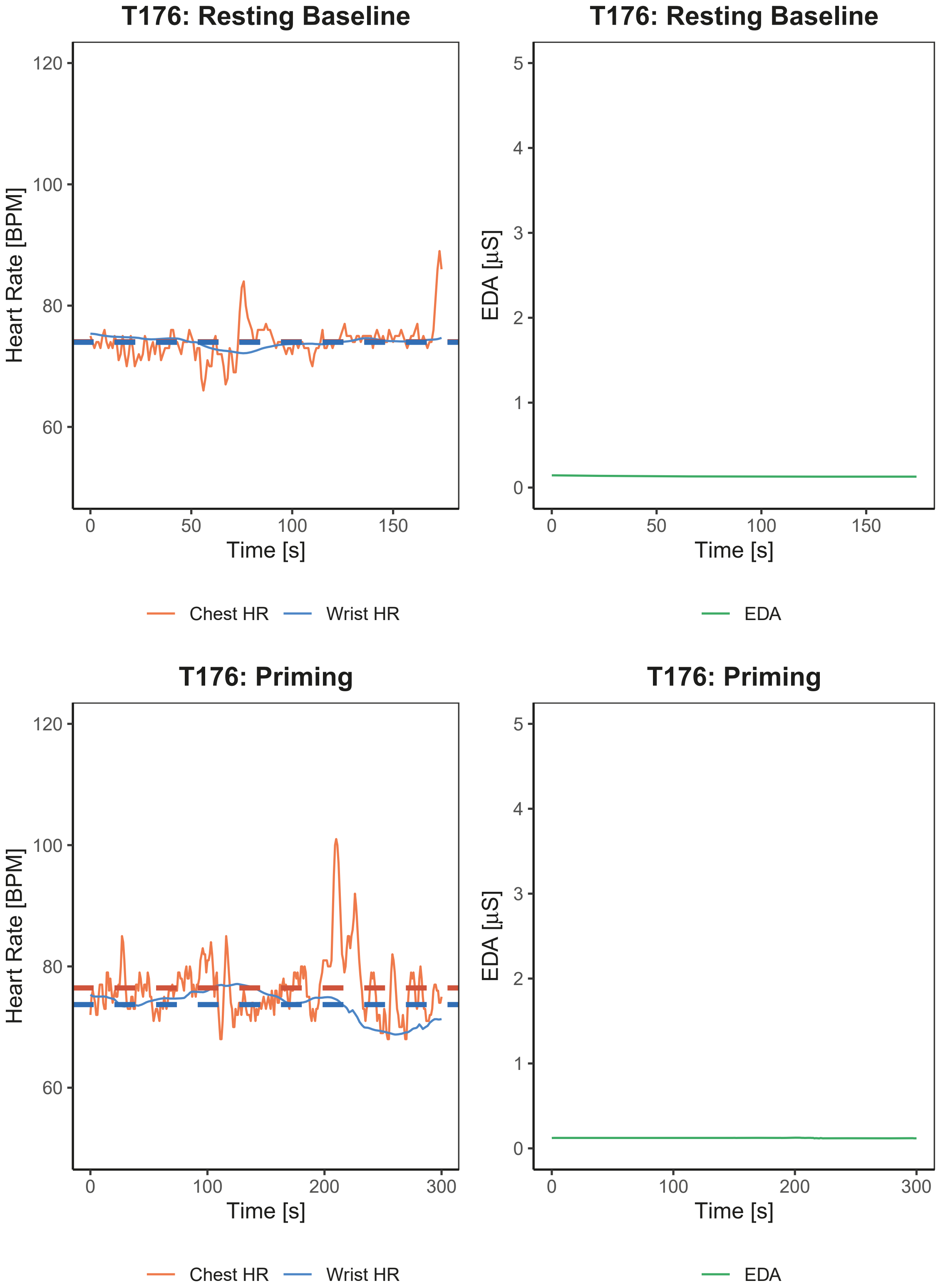
T176: Resting Baseline
Heart Rate [BPM]
Time [s]
Chest HR
Wrist HR
T176: Resting Baseline
EDA [µS]
Time [s]
EDA
T176: Priming
Heart Rate [BPM]
Time [s]
Chest HR
Wrist HR
T176: Priming
EDA [µS]
Time [s]
EDA

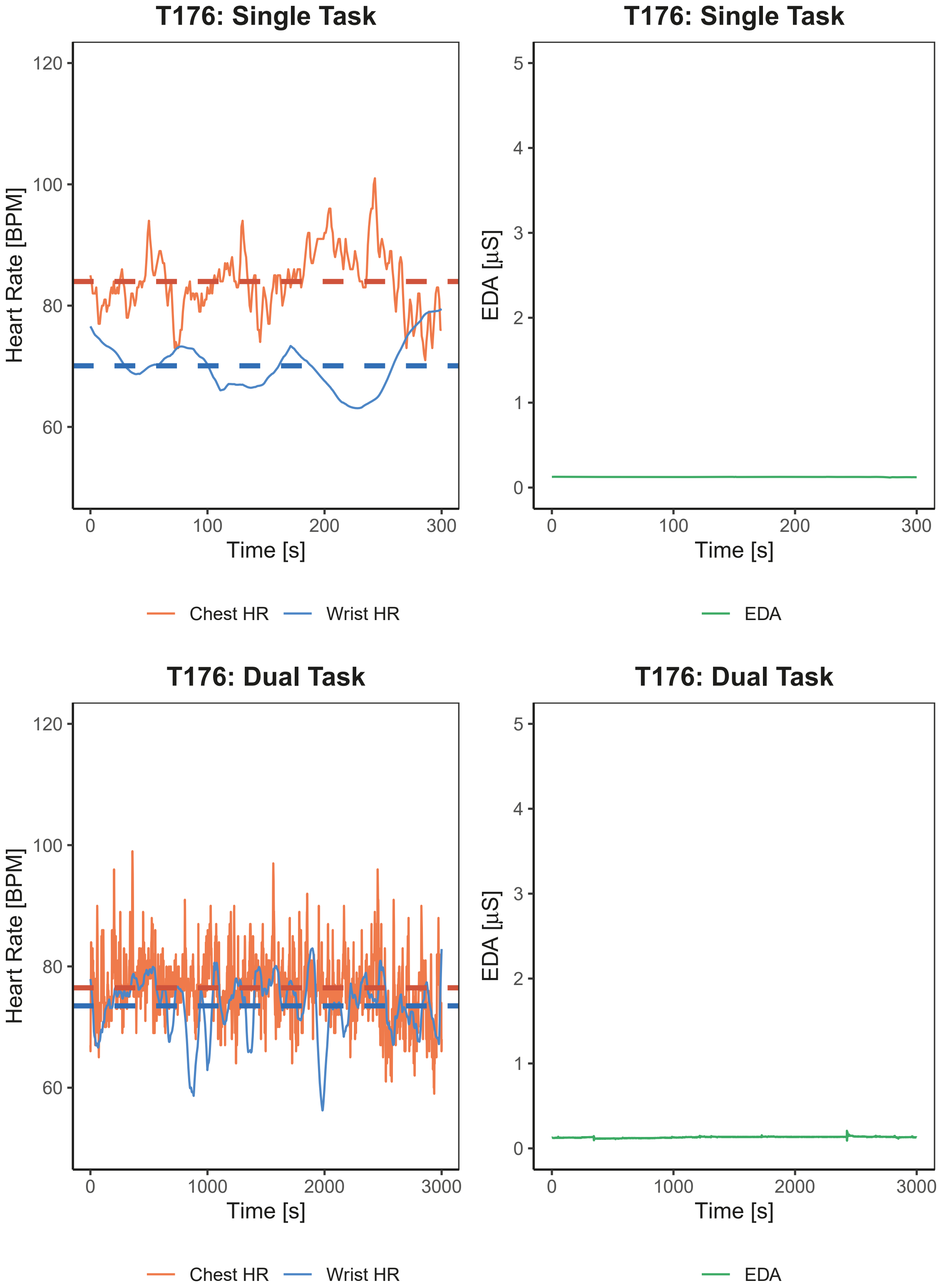
T176: Single Task
Heart Rate [BPM]
120
100
80
60
0
100
200
300
Time [s]
Chest HR
Wrist HR
T176: Single Task
EDA [µS]
5
4
3
2
1
0
0
100
200
300
Time [s]
EDA
T176: Dual Task
Heart Rate [BPM]
120
100
80
60
0
1000
2000
3000
Time [s]
Chest HR
Wrist HR
T176: Dual Task
EDA [µS]
5
4
3
2
1
0
0
1000
2000
3000
Time [s]
EDA

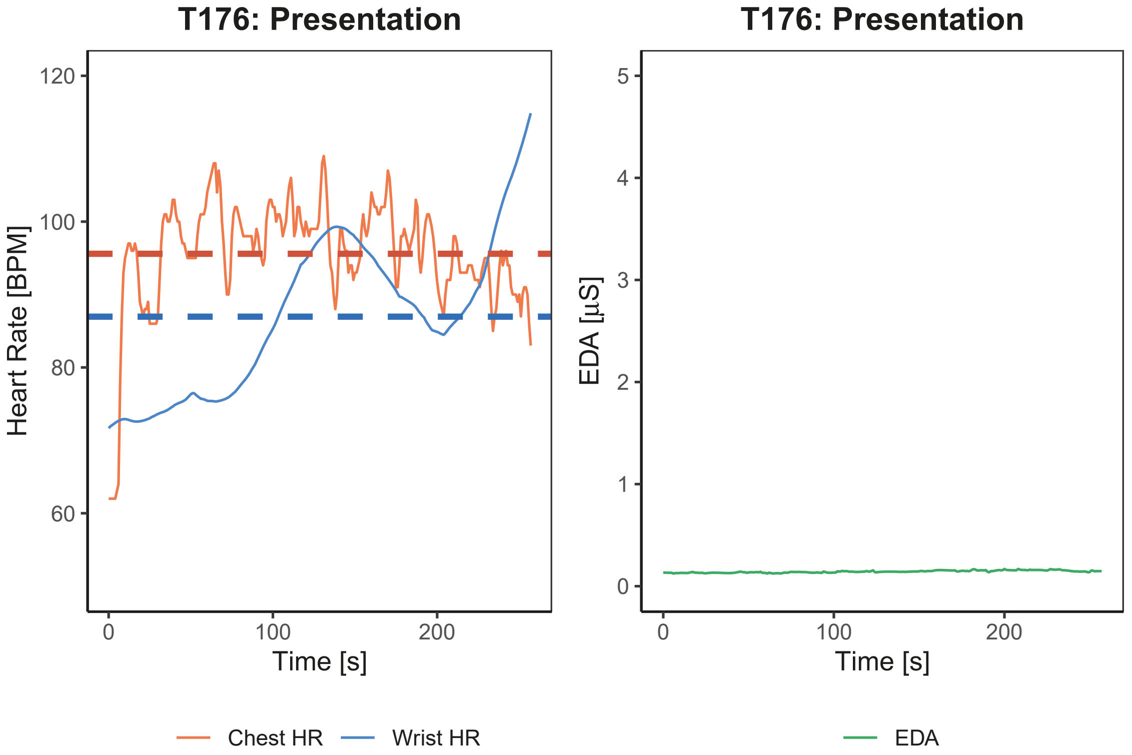

T176: Presentation
Heart Rate [BPM]
120
100
80
60
0
100
200
Time [s]
Chest HR
Wrist HR
T176: Presentation
EDA [μS]
5
4
3
2
1
0
0
100
200
Time [s]
EDA

--

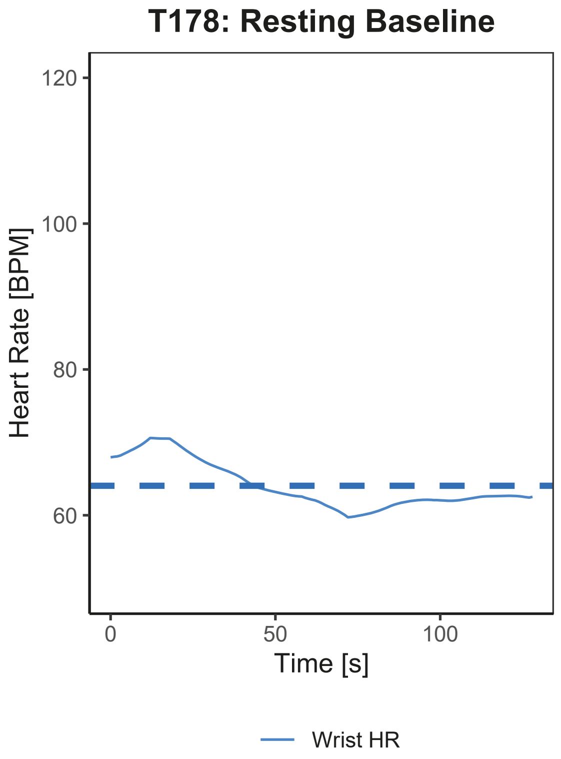
T178: Resting Baseline
Heart Rate [BPM]
120
100
80
60
0
50
100
Time [s]
Wrist HR

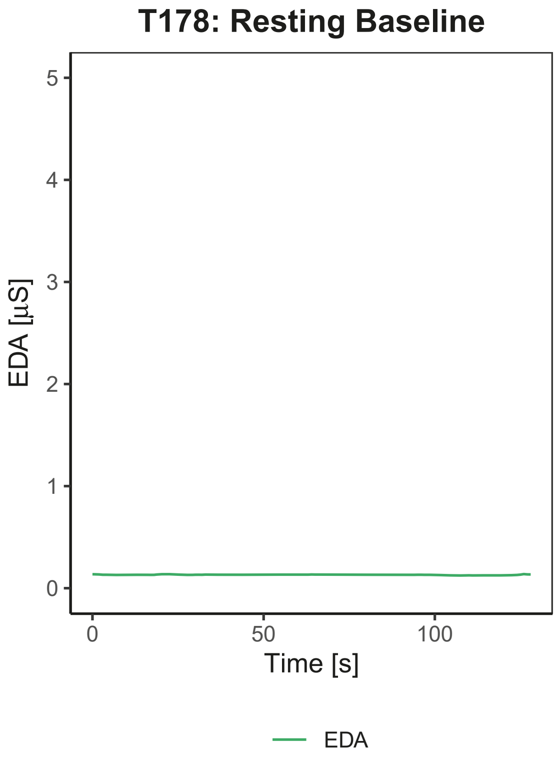
T178: Resting Baseline
EDA [µS]
5
4
3
2
1
0
0
50
100
Time [s]
EDA

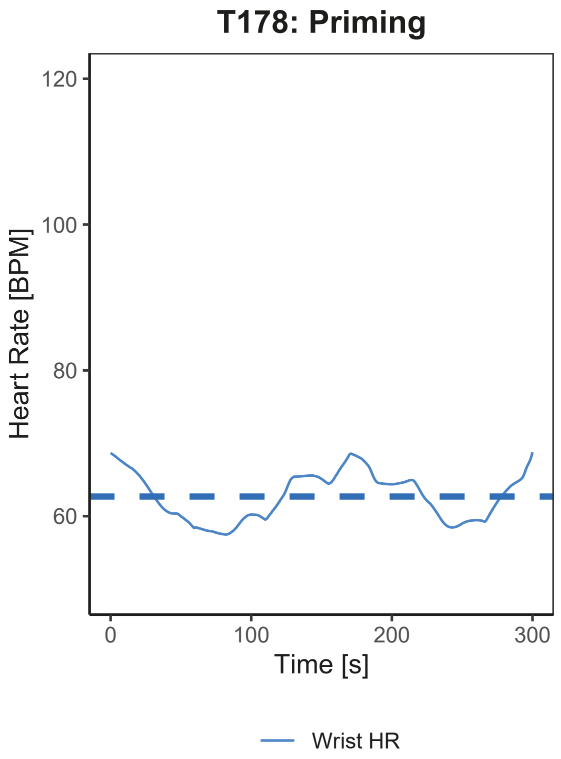
T178: Priming
Heart Rate [BPM]
120
100
80
60
0
100
200
300
Time [s]
Wrist HR

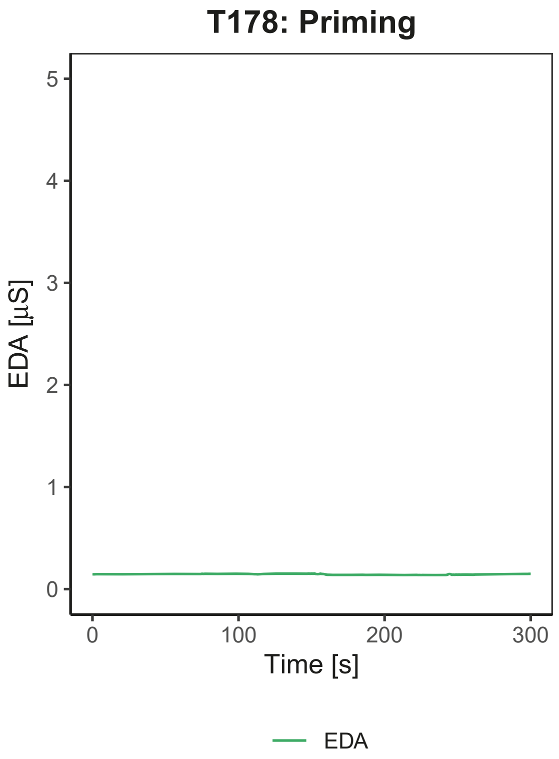
T178: Priming
EDA [µS]
5
4
3
2
1
0
0
100
200
300
Time [s]
EDA

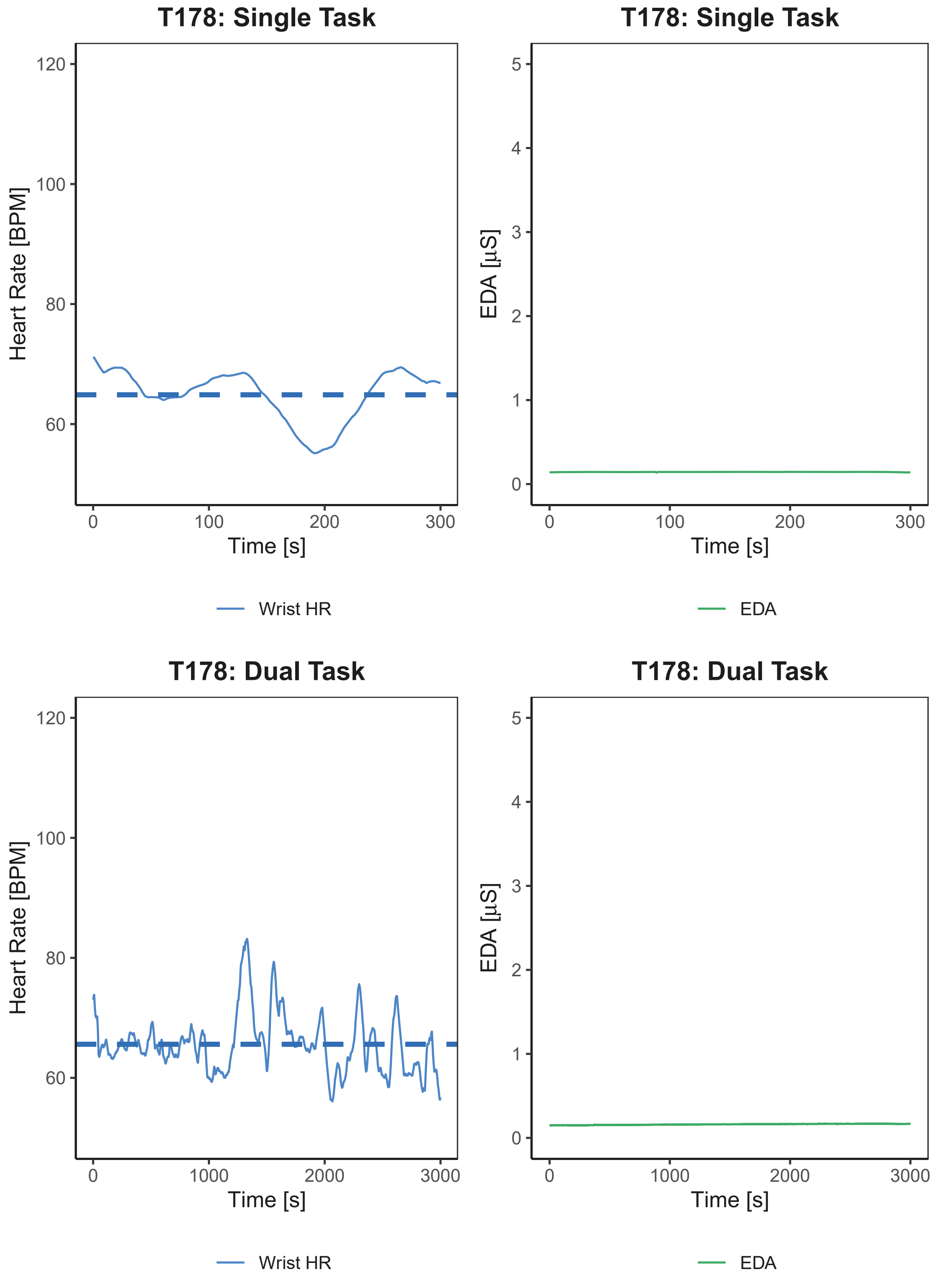
T178: Single Task
Heart Rate [BPM]
Time [s]
Wrist HR
T178: Single Task
EDA [µS]
Time [s]
EDA
T178: Dual Task
Heart Rate [BPM]
Time [s]
Wrist HR
T178: Dual Task
EDA [µS]
Time [s]
EDA

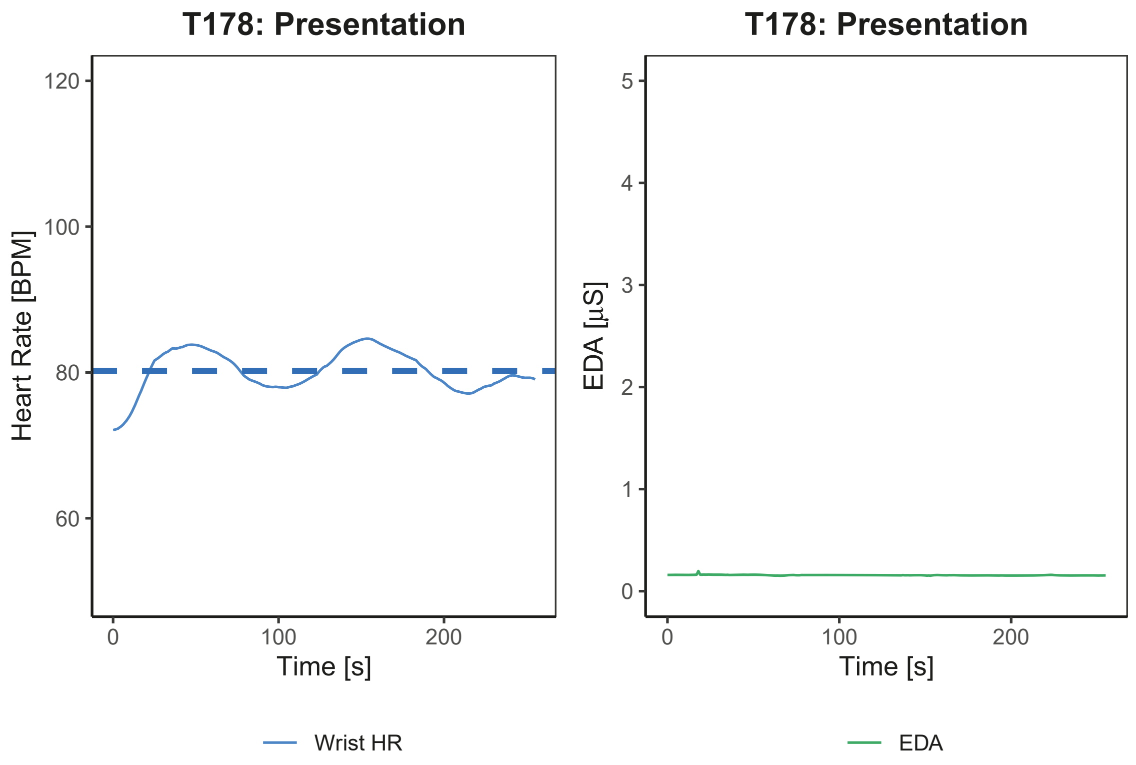

T178: Presentation
Heart Rate [BPM]
120
100
80
60
0
100
200
Time [s]
Wrist HR
T178: Presentation
EDA [µS]
5
4
3
2
1
0
0
100
200
Time [s]
EDA
